# Update of the Genetic Variability of Monkeypox Virus Clade IIb Lineage B.1

**DOI:** 10.3390/microorganisms12091874

**Published:** 2024-09-11

**Authors:** Fabio Scarpa, Ilenia Azzena, Alessandra Ciccozzi, Francesco Branda, Chiara Locci, Maria Perra, Noemi Pascale, Chiara Romano, Giancarlo Ceccarelli, Giuseppe Terrazzano, Pier Luigi Fiori, Massimo Ciccozzi, Marco Casu, Daria Sanna

**Affiliations:** 1 Department of Biomedical Sciences, University of Sassari, 07100 Sassari, Italy; aciccozzi@uniss.it (A.C.); c.locci3@phd.uniss.it (C.L.); m.perra9@studenti.uniss.it (M.P.); fioripl@uniss.it (P.L.F.); darsanna@uniss.it (D.S.); 2 Department of Veterinary Medicine, University of Sassari, 07100 Sassari, Italy; npascale@uniss.it (N.P.); marcasu@uniss.it (M.C.); 3 Unit of Medical Statistics and Molecular Epidemiology, Università Campus Bio-Medico di Roma, 00128 Roma, Italy; f.branda@unicampus.it (F.B.); chiara.romano@unicampus.it (C.R.); m.ciccozzi@unicampus.it (M.C.); 4 Department of Chemical Physical Mathematical and Natural Sciences, University of Sassari, 07100 Sassari, Italy; 5 Department of Public Health and Infectious Diseases, University Hospital Policlinico Umberto I, Sapienza University of Rome, 00161 Rome, Italy; giancarlo.ceccarelli@uniroma1.it; 6 Department of Sciences, University of Basilicata,85100 Potenza, Italy; giuseppe.terrazzano@unibas.it; 7 Azienza Ospedaliera Universitaria (AOU) Sassari, 07100 Sassari, Italy

**Keywords:** monkeypox, clade IIb, lineage B.1, genome-based surveillance, zoonotic disease, phylodynamic, genetics, phylogeny, molecular epidemiology

## Abstract

From 1 January 2022 to 31 May 2024, the World Health Organization (WHO) reported 97,745 laboratory-confirmed Mpox cases, including 203 deaths, across 116 countries. Despite a 2.3% decrease in new cases in May 2024 compared to April 2024, significant regional variations persist. The African Region reported the highest proportion of new cases, while other regions experienced mixed trends. Phylogenomic analyses of the Mpox virus Clade IIb lineage B.1 reveal stable genetic variability with minimal diversification. The Bayesian Skyline Plot indicates a generally stable viral population size with a modest peak in late 2023, followed by a decline. In general, the data indicate that the MPXV outbreak is primarily localized within a few consistent geographic clusters. The virus’s evolution is relatively slow, as indicated by its stable genetic variability, and Clade IIb lineage B.1 does not currently show signs of rapid genetic changes or population growth. The current low level of genetic diversity should not lead to complacency. Ongoing genomic surveillance is essential for effective outbreak management and understanding. This monitoring is crucial for identifying any shifts in the virus’s behavior or transmission, allowing for prompt public health responses and adjustments. In addition, continued vigilance is necessary to detect any new variants that might influence the outbreak’s trajectory.

## 1. Introduction

Monkeypox (Mpox) is a zoonotic viral disease caused by the monkeypox virus (MPXV), a member of the *Orthopoxvirus* genus in the family Poxviridae. The disease was first identified in humans in 1970 in the Democratic Republic of Congo, and since then, sporadic cases and outbreaks have been reported in several African countries, primarily in Central and West Africa. Recently, there has been an increase in reported cases outside of Africa, raising concerns about the potential for global spread [1]. The clinical presentation of monkeypox resembles that of smallpox, although it is generally less severe. Key symptoms include fever, headache, muscle aches, back pain, lymphadenopathy, and a distinctive rash that often begins on the face and spreads to other parts of the body. The rash progresses through several stages—from macules to papules, vesicles, pustules, and finally crusts—which eventually fall off [2]. Transmission of MPXV occurs through close contact with infected animals or through exposure to contaminated materials [3]. Human-to-human transmission, although less common, can occur via respiratory droplets, direct contact with bodily fluids, lesion materials, or contaminated fomites [4]. Despite the availability of vaccines for smallpox that offer cross-protection against Mpox, the cessation of routine smallpox vaccination has contributed to an increase in human susceptibility [5].

MPXV is characterized by double-stranded DNA [6] and is split into two taxonomic groups: Clade I, formerly referred to as the Central African (Congo Basin) clades, and Clade II, previously known as the West African clades [7]. Clade I is linked with more severe disease and higher transmissibility, featuring a case/fatality ratio (CFR) greater than 10%, while Clade II has a CFR of less than 1% [8,9]. Animal hosts for the virus include rodents and non-human primates [10]. Clade II was identified during outbreaks in Nigeria, Singapore, the United Kingdom, and the United States between 2017 and 2019 and is responsible for the rapid global spread observed in 2022 [11,12,13]. Clade II was further subdivided into Clade IIa and Clade IIb. Clade IIa represents a smaller group of older strains, whereas Clade IIb includes more recent strains and has been the primary focus in studies of recent outbreaks. Within Clade IIb, there is a specific lineage known as Lineage B.1, which has been particularly significant in the context of recent global outbreaks. This lineage is responsible for the majority of cases reported outside historically endemic regions, drawing considerable attention due to its widespread transmission and public health impact. Recent research has shed light on the distinct characteristics and molecular composition of this new clade [14], confirming its division into three distinct lineages and identifying the one that emerged in Europe at the end of 2021 and the beginning of 2022 as B.1, which has been responsible for most of the infections. There is currently no specific antiviral treatment for Mpox; however, supportive care and management of complications are critical. Recent developments have included the approval of a specific vaccine for Mpox and the use of antiviral agents, such as tecovirimat, under investigational protocols [15].

In recent years, starting from 2022, there have been several outbreaks of Mpox, causing some concern. Indeed, on 13 May 2022, the WHO was notified of two confirmed and one unconfirmed case of Mpox (from the same household) in the United Kingdom (https://www.who.int/emergencies/disease-outbreak-news/item/2022-DON383, accessed on 31 July 2024). As of 2 October 2022, a total of 68,900 laboratory-confirmed cases of Mpox have been reported to the WHO from 106 countries across all WHO regions (https://www.who.int/publications/m/item/multi-country-outbreak-of-monkeypox--external-situation-report--7---5-october-2022, accessed on 31 July 2024). On 23 July 2022, Tedros Adhanom Ghebreyesus, the Director-General of WHO, declared the outbreak a Public Health Emergency of International Concern (PHEIC), noting that “…*the outbreak has spread rapidly worldwide through new transmission methods that we do not yet fully comprehend*” [16]. The global response included initiatives to raise public awareness to mitigate the disease’s spread and the adaptation of smallpox vaccines for this purpose [17,18]. In May 2023, the World Health Organization announced the conclusion of the PHEIC, attributing the decision to consistent advancements in managing the disease’s spread [19]. As of 31 December 2023, 2803, cases and 22 deaths from 10 counties were reported within the region. Although cases remained relatively low, by 28 July 2024, there had been 99,388 confirmed cases and 208 deaths reported across 116 countries [20,21,22].

This multi-country outbreak is attributed to the Mpox virus belonging to Clade II, which is a less virulent and severe lineage [14].

In this context, given that genetic variability is a crucial indicator providing fundamental information on both the current state and potential expansion of a virus, we present an update on the genome-based genetic variability of MPXV Clade IIb B.1.

## 2. Materials and Methods

The initial genomic evaluation of the Mpox IIb B.1 clade was performed using a subsampling method that considered data from around the world, with a sampling range June 2022–July 2024. The study utilized 3822 high-coverage complete genomes sourced from the GISAID database (available at https://www.epicov.org/epi3/frontend#28ba39), with data access on 31 July 2024, as detailed in the Supplementary Materials (Tables S1 and S2). The analysis utilized the Nextstrain/ncov tool available at the GitHub repository (https://github.com/nextstrain/ncov, accessed on 5 August 2024) and the global phylodynamics section of the GISAID platform (https://gisaid.org/phylodynamics/global/nextstrain/, accessed on 31 July 2024). In this analysis, all genomes belonging to all clades and lineages sampled from June 2022 have been included. The resulting phylogenetic tree was then edited using GIMP 2.8, which can be downloaded from https://www.gimp.org/downloads/oldstable/ (accessed on 7 August 2024).

To infer expansion capabilities and variations in effective population size, we constructed a subset of all available genomes belonging to Clade IIb B.1, based on samples collected between 5 May 2022, and 26 June 2024 (n = 1797).

This dataset, devoted to the population dynamics of the MPXV Clade IIb lineage B.1, was first aligned using the L-INS-I algorithm implemented in the software Mafft version 7.471 [23], and manually checked and cleaned using Unipro UGENE v.35 [24]. After filtering out misaligned sites and sites with more than 75% gaps, the final dataset consisted of 199,944 base pairs. To verify the temporal signal of molecular phylogenies, a tip-to-root regression was performed using the software TempEst 1.5.3 [25]. The tree used for this analysis in TempEst was generated using IQ-Tree 2.3.2 [26] by setting the parameters with default options. The analysis was performed using the software Beast 1.10.4 [27] with simulations of 200 × 10^6^ generations using the Coalescent Bayesian Skyline Model under the lognormal uncorrelated relaxed clock model, following the methods of Scarpa et al. [28].

Due to the lack of a temporal signal (see the Results section for details), which prevented the estimation of the evolutionary rate, it was not possible to use a rate specific to clade B.1 to infer population dynamics. Instead, a higher generic rate was used. Specifically, time calibration of the variation in viral population size was performed using an evolutionary rate of 5 × 10^−5^ [4 × 10^−5^–6 × 10^−5^], as suggested by Firth et al. [29] for variola viruses.

## 3. Results

**Figure 1:**
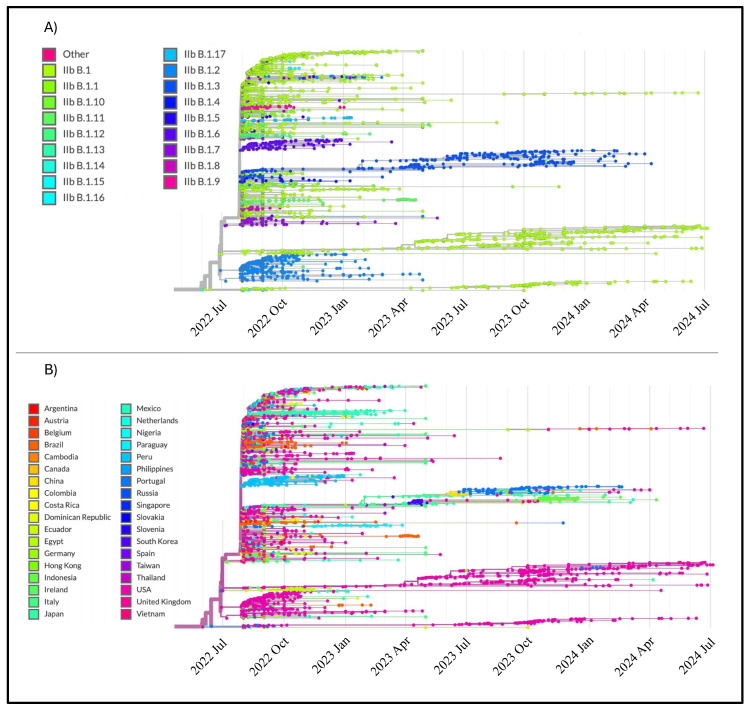
Highlights of all clades and lineages of MPXV collected between June 22 and July 2024 in the time-scaled phylogenetic tree constructed with 3822 complete genomes (last updated 31 July 2024. (**A**) Terminals labeled according to the GISAID Clade of belonging. (**B**) Terminals labeled according to the country of origin. The figure has been edited using the software GIMP 2.8 (available at https://www.gimp.org/downloads/oldstable/, accessed on 7 August 2024).

The phylogenomic reconstruction reported in Figure 1, based on all available genomes from various clades and subtypes, indicates the evolutionary path of the lineages. It is interesting to note that the clusters are formed according to lineage (Figure 1A) but do not show consistency with regard to the country of origin (Figure 1B). In general, the length of the branches indicates a relatively slow rate of evolution. More specifically, older nodes are on shorter branches, while more recent nodes and terminals are on longer branches, indicating a further slowdown in the rate of evolution over time.

The test for the temporal signal performed using a tip-to-root regression shows no positive correlation between sequence divergence and sampling dates (correlation coefficient: −0.2754; R-squared: 0.0759). The correlation coefficient indicates a weak negative correlation between the two variables. This value suggests that there is a slight tendency toward an inverse relationship, but the strength of this relationship is relatively low. Likewise, the R-squared value indicates that only 7.59% of the variability in the dependent variable can be explained by the independent variable. This suggests that the model explains very little of the variability in the data, and there are likely other influencing factors that are not included in the model. Accordingly, the evolutionary rate and divergence time were not estimated for Clade IIb lineage B.1.

**Figure 2:**
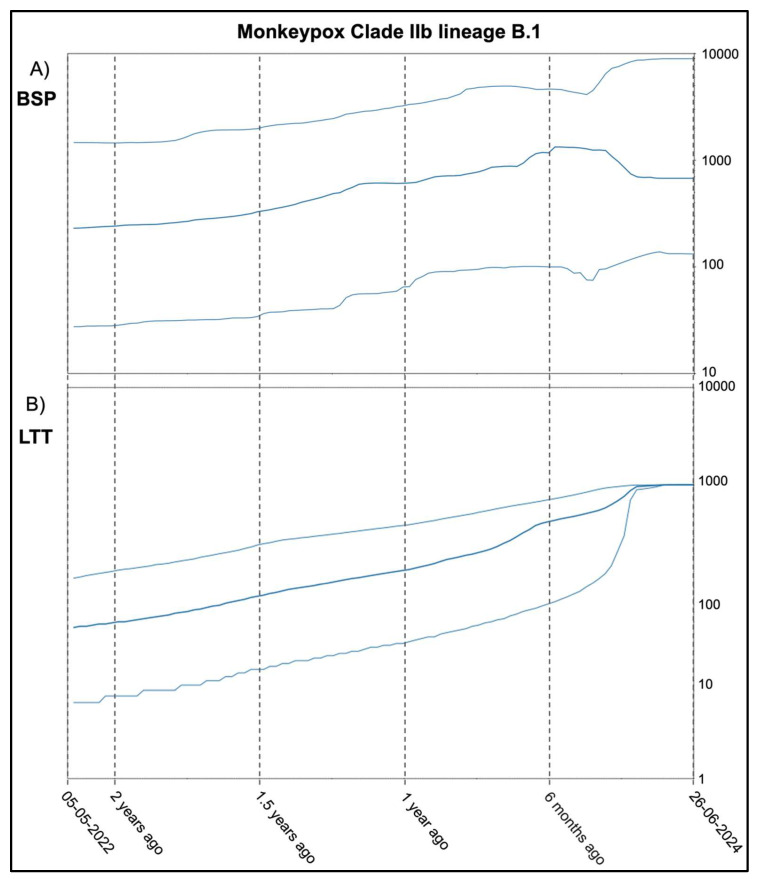
(**A**) Bayesian Skyline Plot (BSP) for the MPXV Clade IIb lineages B.1. This plot shows the genetic variability and thus the effective viral population size (*y*-axis) over time (*x*-axis). (**B**) Lineages Through Times (LTT). The number of lineages (*y*-axis) is displayed as a function of time (*x*-axis). Thin lines represent the 95% high posterior density (HPD) interval.

The Bayesian Skyline Plot (BSP), showed in Figure 2A, depicts the lack of many oscillations over time. The curve shows a slight upward trend, reaching its highest point approximately six months before the most recent sample, dated 26 June 2024. This point does not represent a true peak, but rather the culmination of an increase in genetic variability and, consequently, in the viral population size. After that point, there is a relatively rapid decline until about three months before 26 June, followed by a plateau where no further oscillations in variability are observed.

The Lineages Through Times (LTT) graph (Figure 2B) depicts a similar pattern, showing a slight but steady increase in the number of haplotypes up until approximately three months before 26 June. From this point onward, the graph shows no further growth or variation.

## 4. Discussion

**Figure 3:**
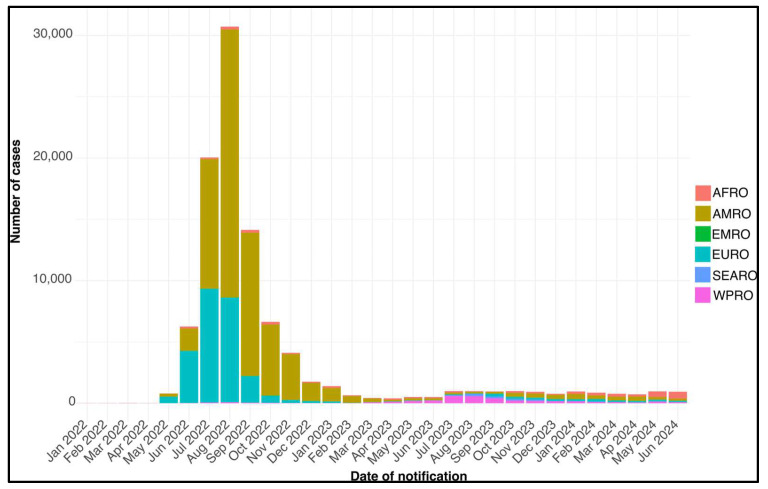
Trend of confirmed cases of Mpox in the six WHO regions from January 2022 to June 2024. For details on the exact number of cases, see Table 1.

The WHO reported that from 1 January 2022 to 31 May 2024, over 99,176 laboratory-confirmed Mpox cases, including 208 deaths, were recorded in 116 countries across all six WHO regions (see Figure 3 and Table 1 for details). In May 2024, 646 new cases were reported, which represents a 2.3% decrease from April 2024, including some cases retrospectively added for previous months (https://cdn.who.int/media/docs/default-source/health-emergency-information-risk-assessment/20240628_mpox_external-sitrep_34.pdf?sfvrsn=7a4abfce_1&download=true, accessed on 31 July 2024). Most May 2024 cases were reported from the African Region (43.5%), followed by the European Region (21.8%), and the Region of the Americas (20.4%). All regions except the Region of the Americas reported an increase in cases in May compared to the previous month (132 cases vs. 316 cases), while no cases were reported in the Eastern Mediterranean Region. In May 2024, 15 out of 26 reporting countries (58%) saw an increase in cases compared to April 2024. The Democratic Republic of Congo had the highest relative increase in the African Region (277 cases vs. 157), France reported the largest increase in the European Region (31 cases vs. 12), Mexico had the highest rise in the Region of the Americas (12 cases vs. 9), Australia reported the most significant increase in the Western Pacific Region (33 cases vs. 5), and Thailand saw the highest increase in the South-East Asia Region (21 cases vs. 19). The WHO continues to urge all countries to ensure that Mpox is classified as a notifiable disease and to report cases, including zero reporting (reporting when no cases have been detected), as per the Standing Recommendations on mpox issued by the WHO Director-General. This report does not address countries with no reported cases. Thus, the lack of reported cases from a country could be due to non-reporting rather than an actual absence of cases. Therefore, the overall decline in reporting to WHO should be interpreted with caution.

From an evolutionary point of view, molecular genome-based analyses of MPXV Clade IIb lineage B.1 suggest a general evolutionary trend with relatively stable genetic variability. Indeed, phylogenomic analysis (Figure 1) supports this view by revealing several small, nearly independent clusters. Occasionally, some specimens from one cluster seem to merge with others, forming a broader epidemic-type cluster with a closed community, similar to seasonal flu patterns (e.g., see Mugosa et al. [30]), and very similar to what was found in the previous survey in 2022 [31]. Furthermore, the geographic distribution of genetic clusters allows them to be linked to their regions of origin. Localized clusters appear as evolutionary dead ends with few or no descendants, and branch lengths suggest minimal diversification. This condition is particularly pronounced in recent terminals that are represented by long branches.

The population dynamics depicted by the Bayesian Skyline Plot (BSP) show that from the earliest sample collected in early May 2022 through late June 2024, the genetic variability was relatively stable, along with, consequently, the viral population size. The plot reveals minimal fluctuations over time. The curve displays a modest upward trend, reaching the higher point about six months prior to the most recent sample dated (i.e., 26 June 2024), that corresponds to late December 2023. This period marks the time when genetic variability and viral population size peaked, maintaining these high levels with minimal fluctuation for about three months. Afterward, there was a rapid decrease, reaching the lowest values around late April. Following this decline, the graph stabilized, showing no further variability and forming a plateau that persisted until the most recent sample date of 26 June 2024. In accordance with this trend, the growth in the number of lineages over time shows a light but constant rise that peaked in late April, starting the plateau phase that corresponds to the plateau phase of the viral population size. The lack of significant fluctuations over time aligns with a scenario based on a very slow growth rate in population size.

At the current status, this condition is not indicative of a viral population with great expansion potential but of a population that struggles to explode due to its biological characteristics. Indeed, this pattern contrasts sharply with the trend observed, for instance, in SARS-CoV-2, which underwent significant mutations throughout the pandemic [32], leading to the emergence of numerous lineages and sub-lineages. Although these new lineages expanded more slowly compared to the initial ones, they exhibited greater potential for expansion [33]. This observation is also supported by the lack of a temporal signal. This condition might be due to minimal differentiation and very low genetic variability within the dataset, a characteristic often observed in DNA viruses [27]. With high levels of variation and a temporal range spanning over two years, we would expect a strong temporal signal. DNA viruses generally evolve more slowly than RNA viruses, resulting in weak or absent correlations between divergence and sampling dates. MPXV does not have the high mutation rate typical of RNA viruses, which facilitates rapid evolution. The dataset, which comprises genomes collected globally over approximately 30 months, shows a maximum genetic distance of 0.007 (±0.0002). This is very similar to the maximum genetic distance reported by Scarpa et al. [31] in a dataset of genomes collected over approximately four months, further highlighting the low capabilities.

Viruses exhibit varying levels of genetic variability due to differences in their genome composition (RNA or DNA), size, replication mechanisms, and the presence or absence of proofreading activities by their polymerases. For instance, RNA viruses often have higher mutation rates because their polymerases typically lack proofreading capabilities, resulting in frequent errors during replication. In contrast, DNA viruses generally have more accurate polymerases with proofreading functions that correct replication errors, leading to lower mutation rates and genetic variability. In general, RNA viruses have higher mutation rates and genetic diversity compared to DNA viruses. RNA viruses produce 10^−6^ to 10^−4^ new base substitutions per nucleotide per cell, while DNA viruses range from 10^−8^ to 10^−6^ [34,35]. In addition, single-stranded viruses tend to mutate faster than double-stranded viruses, and there is an inverse correlation between genome size and mutation rate. Some DNA viruses, such as herpesviruses, exhibit genetic variability comparable to RNA viruses [36]. For instance, cytomegalovirus is known to exhibit significant genetic divergence across different hosts and infection stages [37].

In terms of human health, the biological characteristics of the MPXV are advantageous because they prevent the virus from spreading quickly, and despite more than two years passing, the MPXV has not seen a significant increase in population size or genetic variability.

During the multi-country outbreak, in terms of cases, the epidemic peak occurred in the third quarter of 2022, with the Region of the Americas (AMRO) having the most cases (44,134), followed by the European Region (EURO) with 19,964 cases. This peak represents the rapidly expanding phase of the epidemic, likely due to a combination of factors, such as the novelty of the pathogen in these populations, lack of preexisting immunity, and potential delays in implementing effective control measures. Subsequently, a significant decline in cases was observed in almost all regions. For example, in the AMRO region, the number of cases declined from 44,134 in the third quarter of 2022 to 11,023 in the fourth quarter of 2022, and further to 1941 in the first quarter of 2023. However, this trend has not been consistent across all regions. The African Region (AFRO) has maintained a relatively constant number of cases, fluctuating between 183 and 591 cases per quarter in 2022, with a slight increase to 1207 cases in the second quarter of 2024. This could suggest an endemic situation in the region, possibly due to the presence of animal reservoirs of the virus. The Western Pacific Region (WPRO) showed an interesting trend, with a significant increase in cases in the third quarter of 2023 (1568 cases) compared to previous quarters. This could indicate a delayed spread of the epidemic in this region or possible localized clusters. The Eastern Mediterranean Region (EMRO) and South-East Asia Region (SEARO) generally reported lower numbers of cases, but with some notable increases, such as the 409 cases in SEARO in the third quarter of 2023. Nevertheless, the multi-country Mpox outbreak should not be underestimated. Continuous molecular monitoring at the genomic level is crucial for a comprehensive understanding. Persistent surveillance is necessary due to the potential for asymptomatic infections, which could lead to an underestimation of total infections [38]. The virus may have emerged in March 2022, and the distinct clusters in the phylogenomic analysis suggest an early and subtle spread of the virus [14,39]. It is important to note that, although slow, the evolutionary rate of lineage B.1 is faster than that of other lineages [39]. In addition, it seems that accelerated evolution of human MPXV may be influenced by APOBEC3 activity [8]. APOBEC3 proteins are a family of cytidine deaminases that play crucial roles in the innate immune response against viral infections. These proteins have the ability to introduce mutations into viral DNA or RNA, inhibiting viral replication and spread [40]. When APOBEC3 proteins are active against a virus like MPXV, they can induce a higher rate of mutations in the virus’s genetic material [41]. This increased mutagenesis could potentially lead to accelerated evolution of the virus, as it adapts more quickly to the host environment [42]. However, it is important to note that while APOBEC3 proteins are part of the innate immune system and can contribute to viral control, their role is complex and not fully understood. Many viruses have evolved mechanisms to counteract APOBEC3 effects, and the interplay between APOBEC3 activity and viral evolution is an area of ongoing research [41].

In general, in a constantly evolving environment, infectious diseases like Mpox continue to emerge and re-emerge, presenting significant threats to global health. To effectively manage Mpox outbreaks, prompt response and efficient surveillance are essential. Enhancing global surveillance systems is crucial for early detection and response, necessitating cooperation between governments and international organizations to build robust monitoring networks, establish reporting methods, and improve diagnostic capabilities. Integrating Mpox surveillance into existing disease monitoring platforms can enhance readiness and response. However, detection alone is not enough; rapid and accurate diagnostic technologies are vital for timely case identification and epidemic control. Investments in developing point-of-care diagnostic tools, such as lateral flow assays or portable PCR equipment, are crucial. These tests should be rapid, easy to use, affordable, and capable of distinguishing Mpox from other infections. Understanding Mpox transmission dynamics is also critical. More research is needed to understand how the disease spreads between humans, identify potential reservoirs and vectors, and evaluate the impact of environmental factors on transmission. Such research will help refine targeted interventions, epidemic response strategies, and preventive measures. Animals are crucial for sustaining these infections in the environment through the zoonotic cycle. It is believed that zoonotic transmission is the main method by which infectious agents are introduced into human populations [43]. This recent Mpox outbreak across various countries underscores the danger of new viruses originating from animals, which could lead to epidemics or pandemics and present a serious threat to global health. It is essential to comprehend how zoonotic transmission occurs and to keep track of outbreaks in wildlife populations. Identifying and analyzing animal viruses that pose the greatest risk of spilling over to humans is crucial.

### Strengths and Weaknesses of the Research

The comprehensive dataset of Mpox cases reported by the WHO from January 2022 to May 2024 provides valuable insights into the global trajectory of the outbreak. One of the key strengths of this data is its extensive geographic coverage, which captures reports from 116 countries across all the WHO regions. This broad scope enhances the understanding of the epidemic’s spread and helps identify regional variations in case numbers and trends. The detailed analysis of genetic variability, including phylogenomic and Bayesian Skyline Plot (BSP) evaluations, offers a nuanced view of the virus’s evolutionary dynamics and stability over time. These strengths allow for a robust assessment of the epidemic’s behavior and the virus’s molecular characteristics. However, there are potential weaknesses in the data. The underreporting from certain countries, potentially due to a lack of effective reporting mechanisms, introduces uncertainty regarding the true scale of the outbreak. Additionally, the relatively stable genetic variability of the Mpox virus, compared to more rapidly evolving pathogens like SARS-CoV-2, may obscure potential evolutionary shifts that could influence future outbreaks. The dataset’s emphasis on genetic and epidemiological trends without accounting for non-reported cases highlights areas where further research and improved data collection could enhance the overall understanding of Mpox dynamics.

## 5. Conclusions

The data presented indicate that the Mpox virus outbreak appears to be confined to several geographically consistent epidemic clusters. The evolution of this virus remains slow, as evidenced by its stable genetic variability. Currently, Clade IIb lineage B.1 does not exhibit characteristics typical of a lineage poised for a rapid increase in genetic variability or population size.

However, the current low level of genetic differentiation should not lead to complacency. Continued genome-based surveillance is crucial for effectively managing and understanding the outbreak. This ongoing monitoring will help in detecting any potential changes in the virus’s behavior or spread, allowing for timely interventions and adjustments to public health strategies. Additionally, vigilance is needed to assess any emerging variants that could impact the course of the outbreak.

## Figures and Tables

**Table 1:** Total Mpox cases by WHO region. Data from 22 January as of June 2024.

WHO Region	Total Cases/Deaths	Total Cases/Deaths in 2022	Total Cases/Deaths in 2023	Total Cases/Deaths in 2024
Region of the Americas	62,904/141	57,236/104	3856/34	1812/3
European Region	27,529/10	25,705/5	998/2	826/3
African Region	4232/35	1224/15	1154/7	1854/13
Western Pacific Region	3491/10	229/0	2690/7	572/3
South-East Asia Region	925/11	35/1	755/1	135/9
Eastern Mediterranean Region	95/1	80/1	15/0	0/0

## Data Availability

Genomes analyzed in the present study were taken from the GSAID database and are available at https://gisaid.org/, accessed on 31 July 2024.

## Supplementary Materials

The following supporting information can be downloaded at: https://www.mdpi.com/article/10.3390/microorganisms12091874/s1, Table S1: Acknowledgment Table generated from GISAID for the first used dataset composed by genomes belonging to all clades and all lineages of Mpox virus (n = 3822); Table S2: Acknowledgment Table generated form GISAID for the second used dataset composed by all genomes belonging to Mpox virus Clade IIb lineage B.1 of (n = 1797).
