# Supplementary material for: Update of the Genetic Variability of Monkeypox Virus Clade IIb Lineage B.1
Source: Microorganisms. 2024 Sep 11;12(9):1874. doi: 10.3390/microorganisms12091874 (PMC11434445; doi:10.3390/microorganisms12091874)
Supplement: Supplementary file 1 [file microorganisms-12-01874-s001.zip › Table_S1.pdf]

We gratefully acknowledge the following Authors from the Originating laboratories responsible for obtaining the specimens, as well as the Submitting laboratories where the genome data were generated and shared via GISAID, on which this research is based.

All Submitters of data may be contacted directly via www.gisaid.org

Authors are sorted alphabetically.

Accession ID	Originating Laboratory	Submitting Laboratory	Authors
EPI_ISL_13052263	Microbiol Genomics and Bioinformatics, Bundeswehr Institute of Microbiology	Microbiol Genomics and Bioinformatics, Bundeswehr Institute of Microbiology	Antwerpen,M.H., Lang,D., Zange,S., Walter,M.C. and Woelfel,R.
EPI_ISL_13052266, EPI_ISL_13052267, EPI_ISL_13052268, EPI_ISL_13052269, EPI_ISL_13052270, EPI_ISL_13052272, EPI_ISL_13052273	Instituto Nacional de Saude Doutor Ricardo Jorge (INSA)	Instituto Nacional de Saude Doutor Ricardo Jorge (INSA)	Joana Isidro, Vítor Borges, Miguel Pinto, Daniel Sobral, João Dourado Santos, Alexandra Nunes, Verónica Mixão, Rita Ferreira, Daniela Santos, Silvia Duarte, Luís Vieira, Maria José Borrego, Sofia Núncio, Isabel Lopes de Carvalho, Ana Pelerito, Rita Cordeiro, João Paulo Gomes
EPI_ISL_13052274	Laboratory of Virology, University Hospitals of Geneva	Laboratory of Virology, University Hospitals of Geneva	Laubscher,F., Chudzinski,V., Schibler,M., Kaiser,L. and Renzoni,A.
EPI_ISL_13052282	Microbiology, Immunology and Transplantation, KU Leuven, Rega Institute	Microbiology, Immunology and Transplantation, KU Leuven, Rega Institute	Vanmechelen,B., Wawina-Bokalanga,T., Logist,A.-S., Sinnesael,R., Ysebaert,L., Verlinden,J., Bloemen,M. and Maes,P.
EPI_ISL_13052283	Microbiology, Immunology and Transplantation, KU Leuven, Rega Institute	Microbiology, Immunology and Transplantation, KU Leuven, Rega Institute	Wawina-Bokalanga,T., Vanmechelen,B., Logist,A.-S., Sinnesael,R., Ysebaert,L., Verlinden,J., Bloemen,M. and Maes,P.
EPI_ISL_13052284	Microbiology, Hospital Universitari Germans Trias i Pujol	Microbiology, Hospital Universitari Germans Trias i Pujol	Martinez-Puchol,S., Coello,A., Bordoy,A.E., Soler,L., Panisello,D., Gonzalez-Gomez,S., Clara,G., Paris de Leon,A., Not,A., Hernandez,A., Bofill-Mas,S., Saludes,V., Blanco,I., Martro,E. and Cardona,P.-J.
EPI_ISL_13052285	Laboratory of Virology, University Hospitals of Geneva	Laboratory of Virology, University Hospitals of Geneva	Laubscher,F., Schibler,M., Kaiser,L. and Renzoni,A.
EPI_ISL_13052287	Virology, GENomique EPIdemiologique des maladies Infectieuses	Virology, GENomique EPIdemiologique des maladies Infectieuses	unknown
EPI_ISL_13052288	Department of Health, Utah Public Health Laboratory	Department of Health, Utah Public Health Laboratory	Young,E.L., Hergert,J. and Oakeson,K.F.
EPI_ISL_13052290	Laboratory for Diagnostics of Zoonoses and WHO Centre, Institute of Microbiology and Immunology, Faculty of Medicine, University of Ljubljana	Laboratory for Diagnostics of Zoonoses and WHO Centre, Institute of Microbiology and Immunology, Faculty of Medicine, University of Ljubljana	Zakotnik,S., Vlaj,D., Suljic,A., Zorec,T.M., Korva,M., Poljak,M. and Avsic Zupanc,T.
EPI_ISL_13052291	Laboratory for Diagnostics of Zoonoses and WHO Centre, Institute of Microbiology and Immunology, Faculty of Medicine, University of Ljubljana	Laboratory for Diagnostics of Zoonoses and WHO Centre, Institute of Microbiology and Immunology, Faculty of Medicine, University of Ljubljana	Zakotnik,S., Vlaj,D., Suljic,A., Zorec,T.M., Skubic,C., Rozman,D., Korva,M., Poljak,M. and Avsic Zupanc,T.
EPI_ISL_13052295	SC (UCO) Igiene e Sanità Pubblica, ASUGI, Trieste	Genomics and Epigenomics, AREA Science Park	Licastro,D., DeGasperi,M., Negri,C., Piscianz,E., Koncan,R., Dal Monego,S., Segat,L. and D'Agaro,P.
EPI_ISL_13056233, EPI_ISL_13056234, EPI_ISL_13056235, EPI_ISL_13056236, EPI_ISL_13056237, EPI_ISL_13056238, EPI_ISL_13056239, EPI_ISL_13056240, EPI_ISL_13056241, EPI_ISL_13056242, EPI_ISL_13056243, EPI_ISL_13056244, EPI_ISL_13056245, EPI_ISL_13056246, EPI_ISL_13056247, EPI_ISL_13056248, EPI_ISL_13056249, EPI_ISL_13056250, EPI_ISL_13056251, EPI_ISL_13056252, EPI_ISL_13056253, EPI_ISL_13056254, EPI_ISL_13056255			
see above	USAMRIID, Center for Genome Sciences, United States Army Medical Research Institute of Infectious Diseases	USAMRIID, Center for Genome Sciences, United States Army Medical Research Institute of Infectious Diseases	Kugelman,J.R., Johnston,S.C., Mulembakani,P.M., Kisalu,N., Lee,M.S., Koroleva,G., McCarthy,S.E., Gestole,M.C., Wolfe,N.D., Fair,J.N., Schneider,B.S., Wright,L.L., Huggins,J., Whitehouse,C.A., Wemakoy,E.O., Muyembe-Tamfum,J.J., Hensley,L.E., Palacios,G.F. and Rimoin,A.W.
EPI_ISL_13056275, EPI_ISL_13056276, EPI_ISL_13056277, EPI_ISL_13056278, EPI_ISL_13056279, EPI_ISL_13056280, EPI_ISL_13056281	Centers for Disease Control and Prevention	Centers for Disease Control and Prevention	Yinka-Ogunleye,A., Aruna,O., Dalhat,M., Ogoina,D., McCollum,A., Disu,Y., Mamadu,I., Akinpelu,A., Ahmad,A., Burga,J., Ndoreraho,A., Nkunzimana,E., Manneh,L., Mohammed,A., Adeoye,O., Tom-Aba,D., Silenou,B., Ipadeola,O., Saleh,M., Adeyemo,A., Nwadiutor,I., Aworabhi,N., Uke,P., John,D., Wakama,P., Reynolds,M., Mauldin,M., Doty,J., Wilkins,K., Musa,J., Khalakdina,A., Adedeji,A., Mba,N., Ojo,O., Krause,G. and Ihekweazu,C.
EPI_ISL_13056289	Biochemistry and Molecular Biology, Israel Institute for Biological Research	Biochemistry and Molecular Biology, Israel Institute for Biological Research	Cohen Gihon,I., Israeli,O., Shifman,O., Erez,N., Melamed,S., Paran,N., Beth-Din,A. and Zvi,A.
EPI_ISL_13056892, EPI_ISL_13056893, EPI_ISL_13056894, EPI_ISL_13056895, EPI_ISL_13056896, EPI_ISL_13056897, EPI_ISL_13056899, EPI_ISL_13056900, EPI_ISL_13056901, EPI_ISL_13056902, EPI_ISL_13056903, EPI_ISL_13056904, EPI_ISL_13056905, EPI_ISL_13056906, EPI_ISL_13056907, EPI_ISL_13056908, EPI_ISL_13056909			
see above	Instituto Nacional de Saude Doutor Ricardo Jorge (INSA)	Instituto Nacional de Saude Doutor Ricardo Jorge (INSA)	Joana Isidro, Vítor Borges, Miguel Pinto, Daniel Sobral, João Dourado Santos, Alexandra Nunes, Verónica Mixão, Rita Ferreira, Daniela Santos, Silvia Duarte, Luís Vieira, Maria José Borrego, Sofia Núncio, Isabel Lopes de Carvalho, Ana Pelerito, Rita Cordeiro, João Paulo Gomes
EPI_ISL_13056910	Biochemistry and Molecular Genetics, Israel Institute for Biological Research	Biochemistry and Molecular Genetics, Israel Institute for Biological Research	Israeli,O., Guedj-Dana,Y., Lazar,S., Shifman,O., Erez,N., Weiss,S., Paran,N., Israely,T., Schuster,O., Zvi,A., Beth-Din,A. and Cohen Gihon,I.
EPI_ISL_13058475	National Public Health Laboratory, National Centre for Infectious Diseases	National Public Health Laboratory, National Centre for Infectious Diseases	Yong,S.E.F., Ng,O.T., Ho,Z.J.M., Mak,T.M., Marimuthu,K., Vasoo,S., Yeo,T.W., Ng,Y.K., Cui,L., Ferdous,Z., Chia,P.Y., Aw,B.J.W., Manauis,C.M., Low,C.K.K., Chan,G., Peh,X., Lim,P.L., Chow,L.P.A., Chan,M., Lee,V.J.M., Lin,R.T.P., Heng,M.K.D. and Leo,Y.S.
EPI_ISL_13089461	Hospital General Universitario Gregorio Marañón	Hospital General Universitario Gregorio Marañón	Sergio Buenestado Serrano, Rosalía Palomino Cabrera, Daniel Peñas Utrilla, Jorge Rodríguez-Grande, Laura Pérez-Lago, Cristina Rodriguez-Grande, Marta Herranz Martin, Julia Suárez, Pilar Catalán, Patricia Muñoz, Darío García de Viedma
EPI_ISL_13094227	Centers for Disease Control & Prevention (CDC), Division of High Consequence Pathogens and Pathology (DHCPP-PRB)	Centers for Disease Control & Prevention (CDC), Division of High Consequence Pathogens and Pathology (DHCPP-PRB)	Gigante,C.M., Lee,P., Seabolt,M.H., Wilkins,K., McCollum,A., Hutson,C., Davidson,W., Rao,A., Mendoza,R. and Li,Y.
EPI_ISL_13106454	Hospital General Universitario Gregorio Marañón	Hospital General Universitario Gregorio Marañón	Sergio Buenestado Serrano, Rosalía Palomino Cabrera, Daniel Peñas Utrilla, Jorge Rodríguez-Grande, Pedro Sola Campoy, Laura Pérez-Lago, Cristina Rodriguez-Grande, Marta Herranz Martin, Julia Suárez, Pilar Catalán, Patricia Muñoz, Darío García de Viedma
EPI_ISL_13191438	Instituto de Infectologia Emilio Ribas	Instituto Adolfo Lutz Strategic Laboratory	Claudio Tavares Sacchi, Karoline Rodrigues Campos, Marlon Benedito Nascimento Santos, Alex Domingos Reis, Ariadne Ferreira Amarante, Adriano Abbud, Adriana Bugno, Walkiria Delnoro Almeida Prado, Regiane Cardoso de Paula
EPI_ISL_13194516	Alberta Precision Laboratories	Alberta Precision Laboratories	Matthew Croxen, Ashwin Deo, Paul Dieu, Xiaoli Dong, Kara Gill, David Granger, Christina Ferrato, Vanipriyadarsini Ikkurti, Jamil Kanji, Petya Koleva, Vincent Li, Colin Lloyd, Tarah Lynch, Raymond Ma, Kanti Pabbaraju, Silas Rotich, Hilary Sergeant, Steven Shideler, Todd Skitsko, Sandy Shokopoles, Graham Tipples, Johanna Thayer, Anita Wong
EPI_ISL_13234112	Laboratório Central de Saúde Pública do Estado do Rio Grande do Sul	Instituto Adolfo Lutz Strategic Laboratory	Claudio Tavares Sacchi, Karoline Rodrigues Campos, Adriano Abbud, Adriana Bugno
EPI_ISL_13242738	Hospital General Universitario Gregorio Marañón	Hospital General Universitario Gregorio Marañón	Sergio Buenestado Serrano, Rosalía Palomino Cabrera, Daniel Peñas Utrilla, Jorge Rodríguez-Grande, Pedro Sola Campoy, Laura Pérez-Lago, Cristina Rodriguez-Grande, Marta Herranz Martin, Julia Suárez, Pilar Catalán, Patricia Muñoz, Darío García de Viedma
EPI_ISL_13244349	Erasmus Medical Center Department of Virology	Erasmus Medical Center Department of Virology	Bas Oude Munnink, Marjan Boter, Babette Weller, Richard Molenkamp, Janette Rahamat-Langendoen, Reina Sikkema, Marion Koopmans
EPI_ISL_13251120	Laboratory of Virology, INMI Lazzaro Spallanzani IRCCS	Laboratory of Virology, INMI Lazzaro Spallanzani IRCCS	Giombini,E., Gruber,C.E.M., Rueca,M., Gramigna,G., Vlta,S., Carletti,F., D'Abramo,A., Lapa,D., Puro,V., Fabeni,L., Butera,O., Colavita,F., Meschi,S., Matusali,G., Specchiarello,E., Vairo,F., Vaia,F., Garbuglia,A.R., Nicastri,E., Antinori,A., Glrardi,E. and Maggi,F.
EPI_ISL_13251157	checkin Zollhaus	Institute of Medical Virology, University of Zurich	Verena Kufner, Gabriela Ziltener, Maryam Zaheri, Stefan Schmutz, Annette Audigé, Odette Bernasconi, Kevin Steiner, Jon Huder, Cyril Shah, Riccarda Capaul, Guido Bloemberg, Jürg Böni, Michael Huber, Alexandra Trkola
EPI_ISL_13251584	Division of Infectious Diseases, University Hospital Zürich	Institute of Medical Virology, University of Zurich	Verena Kufner, Gabriela Ziltener, Maryam Zaheri, Stefan Schmutz, Annette Audigé, Odette Bernasconi, Kevin Steiner, Jon Huder, Cyril Shah, Riccarda Capaul, Guido Bloemberg, Jürg Böni, Michael Huber, Alexandra Trkola
EPI_ISL_13251723	checkin Zollhaus	Institute of Medical Virology, University of Zurich	Verena Kufner, Gabriela Ziltener, Maryam Zaheri, Stefan Schmutz, Annette Audigé, Odette Bernasconi, Kevin Steiner, Jon Huder, Cyril Shah, Riccarda Capaul, Guido Bloemberg, Jürg Böni, Michael Huber, Alexandra Trkola
EPI_ISL_13269478	Alberta Precision Laboratories	Alberta Precision Laboratories	Matthew Croxen, Ashwin Deo, Paul Dieu, Xiaoli Dong, Kara Gill, David Granger, Christina Ferrato, Vanipriyadarsini Ikkurti, Jamil Kanji, Petya Koleva, Vincent Li, Colin Lloyd, Tarah Lynch, Raymond Ma, Kanti Pabbaraju, Silas Rotich, Hilary Sergeant, Steven Shideler, Todd Skitsko, Sandy Shokopoles, Graham Tipples, Johanna Thayer, Anita Wong
EPI_ISL_13270980	Instituto de Infectologia Emilio Ribas	Imperial College London, School of Public Health	Claro,I.M., de Lima,E.L., Romano,C.M., Candido,D.S., Lindoso,J.A.L., Barra,L.A.C., Borges,L.M.S., Medeiros,L.A., Tomishige,M.Y.S., Ramundo,M.S., Moutinho,T., da Silva,A.J.D., Rodrigues,C.C.M., de Azevedo,L.C.F., Villas-Boas,L.S., da Silva,C.A.M., Coletti,T.M., O'Toole,A., Quick,J., Loman,N., Rambaut,A., Faria,N.R., Figueiredo-Mello,C. and Sabino,E.C.
EPI_ISL_13302316	Laboratory of Clinical Microbiology, Virology and Bioemergencies. ASST-Fatebenefratelli-Sacco, L.Sacco University Hospital	Army Medical and Veterinary Research Center	Silvia Fillo, Riccardo De Sanctis, Giovanni Faggioni, Andrea Ciammaruconi, Anna Anselmo, Vanessa Vera Fain, Simone Di Sabatino, Francesco Giordani, Antonella Fortunato, Rossella Brandi, Giulia Campoli, Marzia Cavalli, Anella Monte, Martina Lipari, Maria Di Spirito, Giorgia Grilli, Silvia Chimienti, Giandomenico Cerreto, Filippo Molinari, Giancarlo Petralito, Davide Mileto, Valeria Micheli, Maria Rita Gismondo, Florigio Lista
EPI_ISL_13304977	National Public Health Center, National Biosafety Laboratory	National Public Health Center, National Biosafety Laboratory	Judit Henczkó, Dániel Déri, Lili Jármi, Bernadett Pályi, Zoltán Kis,
EPI_ISL_13308158, EPI_ISL_13308160	IRBA Research Institute Biomédicale Des Armées	IRBA Research Institute Biomédicale Des Armées	Jarjaval,F., Nolent,F., Criqui,A., Chapus,C., Lamer,O., Ferraris,O. and Gorge,O.
EPI_ISL_13308167	Laboratory for Diagnostics of Zoonoses and WHO Centre, Institute of Microbiology and Immunology, Faculty of Medicine, University of Ljubljana	Laboratory for Diagnostics of Zoonoses and WHO Centre, Institute of Microbiology and Immunology, Faculty of Medicine, University of Ljubljana	Zakotnik,S., Vlaj,D., Suljic,A., Zorec,T.M., Korva,M., Poljak,M. and Avsic Zupanc,T.
EPI_ISL_13314740	Laboratorio de Vigilancia em Saude de Vinhedo	Instituto Adolfo Lutz Strategic Laboratory	Claudio Tavares Sacchi, Karoline Rodrigues Campos, Adriano Abbud, Adriana Bugno
EPI_ISL_13331598	Department for Virology, Molecular Biology and Genome Research, R. G. Lugar Center for Public Health Research, National Center for Disease Control and Public Health (NCDC) of Georgia	Department for Virology, Molecular Biology and Genome Research, R. G. Lugar Center for Public Health Research, National Center for Disease Control and Public Health (NCDC) of Georgia	Giorgi Tomashvili, Salome Javashvili, Meri Pantsulaia, Gvantsa Brachveli, Ana Papkiauri, Giorgi Gogoladze, Gvantsa Chanturia, Adam Kotorashvili, Maia Alkhazashvili, Khatuna Zakhashvili, Paata Imnadze, Amiran Gamkrelidze.
EPI_ISL_13331712	Laboratory of Virology, INMI Lazzaro Spallanzani IRCCS	Laboratory of Virology, INMI Lazzaro Spallanzani IRCCS	Rueca,M., Giombini,E., Gruber,C.E.M., Gramigna,G., Mazzotta,V., Carletti,F., Lapa,D., Pittalis,S., Puro,V., Fabeni,L., Butera,O., Colavita,F., Meschi,S., Matusali,G., Specchiarello,E., Vairo,F., Vaia,F., Nicastri,E., Antinori,A., Girardi,E. and

			Maggi,F.
EPI_ISL_13331713	Laboratory of Virology, INMI Lazzaro Spallanzani IRCCS	Laboratory of Virology, INMI Lazzaro Spallanzani IRCCS	Gramigna,G., Giombini,E., Gruber,C.E.M., Rueca,M., Carletti,F., Cicalini,S., Lapa,D., Puro,V., Marani,A., Fabeni,L., Butera,O., Colavita,F., Meschi,S., Matusali,G., Rivano Capparuccia,M., Specchiarello,E., Vairo,F., Vaia,F., Nicastri,E., Antinori,A., Girardi,E. and Maggi,F.
EPI_ISL_13331717	Genomics Division, Instituto Tecnologico y de Energias Renovables (ITER), Poligono Industrial de Granadilla	Genomics Division, Instituto Tecnologico y de Energias Renovables (ITER), Poligono Industrial de Granadilla,	Alcoba-Florez,J., Munoz-Barrera,A., Ciuffreda,L., Rodriguez-Perez,H., Rubio-Rodriguez,L.A., Gil-Campesino,H., Garcia-Martinez de Artola,D., Inigo-Campos,A., Diez-Gil,O., Gonzalez-Montelongo,R., Valenzuela-Fernandez,A., Lorenzo-Salazar,J.M. and Flores,C.
EPI_ISL_13338028	Clinical Virology Unit, Department of Clinical Sciences, Institute of Tropical Medicine of Antwerp	Clinical Virology Unit, Department of Clinical Sciences, Institute of Tropical Medicine of Antwerp	Antonio Mauro Rezende*, Tessa de Block*, Sandra Coppens, Eric Florence, Maartje van Frankenhuijsen, Stefanie Bracke, Isabel Brosius, Laurens Liesenborghs, Patrick Soentjens, Kevin Ariën, Marjan Van Esbroeck, Philippe Selhorst*, Koen Vercauteren* *equal contribution
EPI_ISL_13339105	Microbiology Service, Hospital Universitario Clinico San Cecilio, Granada	Microbiology Service, Hospital Universitario Clinico San Cecilio, Granada	Chueca N, de Salazar A, Viñuela L, Fuentes A, Casimiro-Soriguer CS, Perez-Florido J, Dopazo J, Garcia F
EPI_ISL_13342823	Clinical Virology Unit, Department of Clinical Sciences, Institute of Tropical Medicine of Antwerp	Clinical Virology Unit, Department of Clinical Sciences, Institute of Tropical Medicine of Antwerp	Philippe Selhorst, Antonio Mauro Rezende, Tessa de Block, Sandra Coppens, Eric Florence, Isabel Brosius, Laurens Liesenborghs, Kevin Ariën, Marjan Van Esbroeck, Chris Kenyon, Koen Vercauteren
EPI_ISL_13343634	Instituto de Infectologia Emilio Ribas	Instituto Adolfo Lutz Strategic Laboratory	Claudio Tavares Sacchi, Karoline Rodrigues Campos, Adriano Abbud, Adriana Bugno
EPI_ISL_13343697	Fleury Medicina Dignóstica	Instituto Adolfo Lutz Strategic Laboratory	Claudio Tavares Sacchi, Karoline Rodrigues Campos, Adriano Abbud, Adriana Bugno
EPI_ISL_13343718	Hospital Santa Ignes	Instituto Adolfo Lutz Strategic Laboratory	Claudio Tavares Sacchi, Karoline Rodrigues Campos, Adriano Abbud, Adriana Bugno
EPI_ISL_13362760, EPI_ISL_13362764	Laboratorio di Epidemiologia Molecolare e Sanità Pubblica-Policlinico Bari	Istituto Zooprofilattico Sperimentale della Puglia e della Basilicata	Parisi A, Simone D, Capozzi L, Del Sambro L, Bianco A, Chironna M, Loconsole D, Sallustio F, Galante D, Pace L, Manzulli V, Fasanella A.
EPI_ISL_13363142	Hospital Universitari Vall d'Hebron	Hospital Universitari Vall d'Hebron	Maria Piñana, Cristina Andrés, Alejandra González-Sánchez, Damir Garcia-Cehic, Ariadna Rando, Juliana Esperalba, Maria Gema Codina, Maria Carmen Martin, Carla Castillo, Karen García, Rodrigo Vásquez, Maria Piquer, Tomàs Pumarola, Josep Quer, Andrés Antón
EPI_ISL_13374487	National Public Health Center, National Biosafety Laboratory	National Public Health Center, National Biosafety Laboratory	Judit Henczkó, Dániel Déri, Fruzsina Petrovay, Lili Jármi, Bernadett Pályi, Eszter Balla, Zoltán Kis
EPI_ISL_13408799, EPI_ISL_13408801, EPI_ISL_13408803	Public Health Agency of Canada, National Microbiology Laboratory	Public Health Agency of Canada, National Microbiology Laboratory	Knox,N., Hole,D., Duggan,A., Yadav,C., Haidl,E., Chapel,M., Graham,M., Domselaar,G.V., Jolly,G., Audet,J., Fernando,L., Antonation,K., Hagan,M., Griffiths,E., Leung,A., Safronetz,D., Eshaghi,A., Gubbay,J.B., Hasso,M., Marchand-Austin,A., Olsha,R. and Patel,S.N.
EPI_ISL_13408805, EPI_ISL_13408807, EPI_ISL_13408809, EPI_ISL_13408811, EPI_ISL_13408813, EPI_ISL_13408815, EPI_ISL_13408817, EPI_ISL_13408819, EPI_ISL_13408821, EPI_ISL_13408823, EPI_ISL_13408825, EPI_ISL_13408827, EPI_ISL_13408829, EPI_ISL_13408831, EPI_ISL_13408833, EPI_ISL_13408835			
see above	Public Health Agency of Canada, National Microbiology Laboratory	Public Health Agency of Canada, National Microbiology Laboratory	ncknox
EPI_ISL_13408837, EPI_ISL_13408839, EPI_ISL_13408841, EPI_ISL_13408843, EPI_ISL_13408845, EPI_ISL_13408847, EPI_ISL_13408849, EPI_ISL_13408851, EPI_ISL_13408853, EPI_ISL_13408855, EPI_ISL_13408857, EPI_ISL_13408859, EPI_ISL_13408861			
see above	Public Health Agency of Canada, National Microbiology Laboratory	Public Health Agency of Canada, National Microbiology Laboratory	Knox,N., Duggan,A., Yadav,C., Hole,D., Haidl,E., Chapel,M., Jolly,G., Domselaar,G.V., Antonation,K., Leung,A., Fernando,L., Audet,J., Hagan,M., Graham,M., Griffiths,E., Safronetz,D., Charest,H., Levade,I. and Fafard,J.
EPI_ISL_13409177, EPI_ISL_13409178, EPI_ISL_13409179, EPI_ISL_13409180, EPI_ISL_13409181	Viral Genomics and Bioinformatics, MRC University of Glasgow Centre for Virus Research	Viral Genomics and Bioinformatics, MRC University of Glasgow Centre for Virus Research	Filipe,A., Tong,L., Vattipally,S.B., Maclean,A., Gunson,R., Holden,M.T.G., Barr,D., Ho,A., Palmarini,M., Rambaut,A., Robertson,D.L. and Thomson,E.C.
EPI_ISL_13436658	Coordenadoria de Vigilancia em Saude - Sao Paulo	Instituto Adolfo Lutz Strategic Laboratory	Claudio Tavares Sacchi, Karoline Rodrigues Campos, Ariadne Fereira Amarante, Adriano Abbud, Adriana Bugno
EPI_ISL_13436792	Hospital Santa Ignes	Instituto Adolfo Lutz Strategic Laboratory	Claudio Tavares Sacchi, Karoline Rodrigues Campos, Adriano Abbud, Adriana Bugno
EPI_ISL_13437056	Hosp. Alemao Oswaldo Cruz	Instituto Adolfo Lutz Strategic Laboratory	Claudio Tavares Sacchi, Karoline Rodrigues Campos, Ariadne Ferreira Amarante, Adriano Abbud, Adriana Bugno
EPI_ISL_13449965, EPI_ISL_13449966	Hospital Universitario La Paz, Microbiology	Hospital Universitario La Paz, Microbiology	de la Hoz-Sanchez,B., Lopez-Ortiz,M., Gutierrez-Arroyo,A., Roces-Alvarez,P., Lazaro-Peona,F., Dahdouh,E., Bloise,I., Garcia-Rodriguez,J. and Mingorance,J.
EPI_ISL_13459346	CRT-DST-AIDS	Instituto Adolfo Lutz Strategic Laboratory	Claudio Tavares Sacchi, Karoline Rodrigues Campos, Ariadne Ferreira Amarante, Adriano Abbud, Adriana Bugno
EPI_ISL_13459347, EPI_ISL_13459482, EPI_ISL_13459483	Instituto de Infectologia Emilio Ribas	Instituto Adolfo Lutz Strategic Laboratory	Claudio Tavares Sacchi, Karoline Rodrigues Campos, Ariadne Ferreira Amarante, Adriano Abbud, Adriana Bugno
EPI_ISL_13466447, EPI_ISL_13466448, EPI_ISL_13466449, EPI_ISL_13466450, EPI_ISL_13466451, EPI_ISL_13466452, EPI_ISL_13466453, EPI_ISL_13466455, EPI_ISL_13466456, EPI_ISL_13466457, EPI_ISL_13466458, EPI_ISL_13466459, EPI_ISL_13466460, EPI_ISL_13466461, EPI_ISL_13466462, EPI_ISL_13466463, EPI_ISL_13466464, EPI_ISL_13466465			
see above	Department of Infectious Diseases, National Institute of Health Doutor Ricardo Jorge, Portugal (INSA)	Department of Infectious Diseases, National Institute of Health Doutor Ricardo Jorge, Portugal (INSA)	Isidro,J., Borges,V., Pinto,M., Sobral,D., Santos,J., Nunes,A., Mixao,V., Ferreira,R., Santos,D., Duarte,S., Vieira,L., Borrego,M.J., Nuncio,S., Lopes de Carvalho,I., Pelerito,A., Cordeiro,R., Gomes,J.P.
EPI_ISL_13472080	National Institute of Public Health NIH - NRI	National Institute of Public Health NIH - NRI	Wołkowicz Tomasz, Zacharczuk Katarzyna, Gierczyński Rafał
EPI_ISL_13472250	Medical University of Vienna Center for Virology	Medical University of Vienna Center for Virology	Jeremy V. Camp, Monika Redlberger-Fritz, Stephan W. Aberle
EPI_ISL_13484458	Laboratorio de Enterovirus, Instituto Oswaldo Cruz, Fiocruz	Instituto Oswaldo Cruz FIOCRUZ - Laboratory of Respiratory Viruses and Measles (LVRS)	Paola Resende, Elisa Cavalcante Pereira, Bruna Mendonça da Silva, Jéssica Graça Macedo de Carvalho, Larissa Macedo Pinto, Victor Guimaraes, Marilda Siqueira, Renan da Silva Faustino, Marilia Santini, Edson Elias da Silva on behalf of the Fiocruz Genomic Surveillance Network
EPI_ISL_13498265	National Institute for Communicable Diseases of the National Health Laboratory Service	National Institute for Communicable Diseases of the National Health Laboratory Service	Chan WY, Mtshali PS, Grobbelaar A, Moolla N, Mohale T, Du Plessis MG, Ismail A, Weyer J
EPI_ISL_13502582	Laboratory of Microbiology and Virology, Ospedale Amedeo di Savoia, ASL "Città di Torino"	Laboratory of Microbiology and Virology, Ospedale Amedeo di Savoia, ASL "Città di Torino"	Francesco Cerutti, Antonella Bottoni, Marisa Cazzadore, Tiziano Allice, Maria Grazia Milia, Gabriella Gregori, Elisa Burdino, Valeria Ghisetti
EPI_ISL_13508393	Hosp. Itacolomy Butanta	Instituto Adolfo Lutz Strategic Laboratory	Claudio Tavares Sacchi, Karoline Rodrigues Campos, Ariadne Ferreira Amarante, Adriano Abbud, Adriana Bugno
EPI_ISL_13508471	Instituto de Infectologia Emilio Ribas	Instituto Adolfo Lutz Strategic Laboratory	Claudio Tavares Sacchi, Karoline Rodrigues Campos, Ariadne Ferreira Amarante, Adriano Abbud, Adriana Bugno
EPI_ISL_13511312	Laboratorio de Salud Pública de Antioquia	Instituto Nacional de Salud- Dirección de Investigación en Salud Pública	Katherine Laiton-Donato, Diego A. Álvarez-Díaz, Carlos Franco-Muñoz, Héctor A. Ruiz-Moreno, Paola Rojas-Estevez, Andres Prada, Alicia Rosales, Marcela Mercado-Reyes
EPI_ISL_13530881	Laboratorio de Referencia Nacional de Virus Respiratorios. Centro Nacional de Salud Publica. Instituto Nacional de Salud Peru.	Laboratorio de Referencia Nacional de Virus Respiratorios. Centro Nacional de Salud Publica. Instituto Nacional de Salud Peru.	Carlos Padilla Rojas, Veronica Hurtado Vela, Iris Silva Molina, Luren Sevilla Castañeda, Victor Jimenez Vasquez, Orson Mestanza Millones, Luis Barcena Flores, Wendy Lizarraga Olivares, Alicia Nuñez Llanos, Steve Acedo Lazo, Francisco Ascue Orosco, Kelly Izarra Rojas, Princesa Medrano Alhuay, Karla Vasquez Cajachahua, Estela Huaman Angeles, Jorge Giraldo Chavez, Lilian Huarca Balbin, Lisbet Roxana Inga Angulo, Maria Sandra Villar Saavedra, Henri Bailon Calderon, Lely Solari Zerpa, Gloria Arotinco Garayar. Equipo de vigilancia genomica del Instituto Nacional de Salud.
EPI_ISL_13537922	Instituto de Medicina Tropical de Sao Paulo (IMT-USP)	School of Public Health, Imperial College London	Coletti,T.M., Ghilardi,F., khan,M.J., Claro,I.M., Valenca,I.N., Faria,N.R. and Sabino,E.C.
EPI_ISL_13537923	Microbiology, Immunology and Transplantation, KU Leuven, Rega Institute	Microbiology, Immunology and Transplantation, KU Leuven, Rega Institute	Wawina-Bokalanga,T., Vanmechelen,B., Logist,A.-S., Sinnesael,R., Ysebaert,L., Bloemen,M. and Maes,P.
EPI_ISL_13537924, EPI_ISL_13537925, EPI_ISL_13537926	Microbiology, Immunology and Transplantation, KU Leuven, Rega Institute	Microbiology, Immunology and Transplantation, KU Leuven, Rega Institute	Vanmechelen,B., Wawina-Bokalanga,T., Logist,A.-S., Sinnesael,R., Ysebaert,L., Verlinden,J., Van Holm,B., Bloemen,M. and Maes,P.
EPI_ISL_13544223, EPI_ISL_13544224, EPI_ISL_13544225, EPI_ISL_13544226, EPI_ISL_13544227, EPI_ISL_13544228, EPI_ISL_13544229, EPI_ISL_13544230, EPI_ISL_13544231, EPI_ISL_13544232, EPI_ISL_13544233, EPI_ISL_13544234, EPI_ISL_13544235			
see above	Public Health Agency of Canada, National Microbiology Laboratory	Public Health Agency of Canada, National Microbiology Laboratory	Duggan,A., Hole,D., Knox,N., Yadav,C., Haidl,E., Chapel,M., Domselaar,G.V., Jolly,G., Audet,J., Fernando,L., Antonation,K., Safronetz,D., Hagan,M., Griffiths,E., Leung,A., Graham,M., Peters,G., Go,A., Laminman,V., Kaplen,B., Eshaghi,A., Gubbay,J.B., Hasso,M., Marchand-Austin,A., Olsha,R. and Patel,S.N.
EPI_ISL_13544237, EPI_ISL_13544238, EPI_ISL_13544239, EPI_ISL_13544240, EPI_ISL_13544241, EPI_ISL_13544243, EPI_ISL_13544244, EPI_ISL_13544245, EPI_ISL_13544246, EPI_ISL_13544247, EPI_ISL_13544248, EPI_ISL_13544249, EPI_ISL_13544250, EPI_ISL_13544251, EPI_ISL_13544252, EPI_ISL_13544253, EPI_ISL_13544254, EPI_ISL_13544255, EPI_ISL_13544256, EPI_ISL_13544257, EPI_ISL_13544258, EPI_ISL_13544259, EPI_ISL_13544260, EPI_ISL_13544261, EPI_ISL_13544262, EPI_ISL_13544263, EPI_ISL_13544264, EPI_ISL_13544265, EPI_ISL_13544266, EPI_ISL_13544267			
see above	Public Health Agency of Canada, National Microbiology Laboratory	Public Health Agency of Canada, National Microbiology Laboratory	Duggan,A., Hole,D., Knox,N., Yadav,C., Haidl,E., Chapel,M., Domselaar,G.V., Fernando,L., Graham,M., Antonation,K., Audet,J., Hagan,M., Safronetz,D., Leung,A., Peters,G., Go,A., Laminman,V., Kaplen,B., Jolly,G., Charest,H., Levade,I. and Fafard,J.
EPI_ISL_13573943	Center for Virology, Medical University of Vienna	Medical University of Vienna Center for Virology	Jeremy V. Camp, Monika Redlberger-Fritz, Stephan W. Aberle
EPI_ISL_13584854, EPI_ISL_13586184	Institute for Virology, Philipps-University Marburg	Institute for Virology, Philipps-University Marburg	Eickmann, M., Lier, C., Kowalski, K., Kraft, F., Becker, S.
EPI_ISL_13607904	Servicio de Infectologia, Hospital Universitario Dr. José Eleuterio Gonzalez, Universidad Autonoma de Nuevo Leon	Centro de Investigacion e Innovacion en Virologia Medica, Departamento de Bioquimica y Medicina Molecular, Facultad de Medicina, Universidad Autonoma de Nuevo Leon	Kame A. Galan-Huerta, Manuel Paz Infanzon, Ali F. Ruiz Higareda, Laura Nuzzolo-Shihadeh, Adrian Camacho-Ortiz, Paola Bocanegra-Ibarias, Ana M. Rivas-Estilla, Daniel Zacarias-Villarreal, Luis A. Yamallel-Ortega, Maria D. Guerrero-Putz, Jorge Ocampo-Candiani
EPI_ISL_13624509	Instituto de Diagnóstico y Referencia Epidemiológicos/Jurisdicción Sanitaria Cuauhtémoc/Hospital Ángeles Roma	Instituto de Diagnóstico y Referencia Epidemiológicos/Instituto de Biotecnología UNAM	Adnan Araiza-Rodríguez, Adriana Salvador-Patiño, Alejandro Sánchez-Flores, América del Pilar Mandujano-Martínez, Blanca Taboada, Carlos Eduardo Hernández-Sánchez, Carlos F. Arias, Claudia Elena Wong-Arámbula, Daniel José Regalado-Santiago, David Esaú Fragoso-Fonseca, Elizabeth Andrade-Montiel, Fabiola Garcés-Ayala, Fernando González-Domínguez, Gabriel García-Rodríguez, Gloria Vázquez-Castro, Hugo López Gatell Ramírez, Irma López-Martínez, Jerome Verleyen, Jesús Trujillo, Jorge Ochoa, José Ernesto Ramírez-González, Karel Estrada-Guerra, Lucía Hernández-Rivas, Magaly Guadalupe Landa-Flores, Maribel González-Villa, Mireya Mederos-Michel, Nancy Martínez-Velázquez, Noé Escobar-Escamilla, Oliva López, Ricardo Cortés-Alcalá, Ricardo Grande, Verónica Jiménez-Jacinto
EPI_ISL_13632071	Center of Diagnostics and Vaccine Development, Centers for Disease Control, Taiwan	Center of Diagnostics and Vaccine Development, Centers for Disease Control, Taiwan	Jih-Hui Lin, Shu-Chun Chiu, Hsin-I, Huang, Wei-Lun Huang, Wen-Bin, Fann, Pei-Yu, Hsieh, Jyh-Yuan Yang
EPI_ISL_13632288	National Institute for Communicable Diseases of the National Health Laboratory Service	National Institute for Communicable Diseases of the National Health Laboratory Service	Chan WY, Mtshali PS, Grobbelaar A, Moolla N, Mohale T, Lowe M, Du Plessis MG, Ismail A, Weyer J
EPI_ISL_13651348, EPI_ISL_13651349, EPI_ISL_13651350	Laboratorio de Referencia Nacional de Virus Respiratorio. Centro Nacional de Salud Publica. Instituto Nacional de Salud.	Laboratorio de Referencia Nacional de Virus Respiratorio. Centro Nacional de Salud Publica. Instituto Nacional de Salud.	Carlos Padilla Rojas, Veronica Hurtado Vela, Iris Silva Molina, Luren Sevilla Castañeda, Victor Jimenez Vasquez, Orson Mestanza Millones, Luis Barcena Flores, Wendy Lizarraga Olivares, Alicia Nuñez Llanos, Steve Acedo Lazo, Francisco Ascue Orosco, Kelly Izarra Rojas, Princesa Medrano Alhuay, Karla Vasquez Cajachahua, Estela Huaman Angeles, Jorge Giraldo Chavez, Lilian Huarca Balbin, Lisbet Roxana Inga Angulo, Maria Sandra Villar Saavedra, Henri Bailon Calderon, Lely Solari Zerpa, Gloria Arotinco Garayar. Equipo de vigilancia genomica del Instituto Nacional de Salud.
EPI_ISL_13658019, EPI_ISL_13658021	Erasmus Medical Center Department of Virology	Erasmus Medical Center Department of Virology	Bas Oude Munnink, Marjan Boter, Babette Weller, Richard Molenkamp, Janette Rahamat-Langendoen, Reina Sikkema, Marion Koopmans
EPI_ISL_13660191	Hospital Center Luxembourg	Laboratoire National de Santé Microbiology	Eric Hugoson, Ines Kozar, Sibel Berger, Anke Wienecke-Baldacchino, Bas Oude Munnink, Michel Kohnen, Jean-Hugues Francois, Tamir Abdelrahman

EPI_ISL_13705358	Hosp. Alemao Oswaldo Cruz	Instituto Adolfo Lutz Strategic Laboratory	Claudio Tavares Sacchi, Karoline Rodrigues Campos, Ariadne Ferreira Amarante, Marlon Benedito Nascimento Santos, Alex Domingos Reis, Adriano Abbud, Adriana Bugno
EPI_ISL_13705407	Hosp. Sirio-Libanes	Instituto Adolfo Lutz Strategic Laboratory	Claudio Tavares Sacchi, Karoline Rodrigues Campos, Ariadne Ferreira Amarante, Marlon Benedito Nascimento Santos, Alex Domingos Reis, Adriano Abbud, Adriana Bugno
EPI_ISL_13717674	Hospital Center Luxembourg	Laboratoire National de Santé Microbiology	Eric Hugoson, Ines Kozar, Sibel Berger, Anke Wienecke-Baldacchino, Bas Oude Munnink, Michel Kohnen, Jean-Hugues Francois, Tamir Abdelrahman
EPI_ISL_13728303	Department of Medical Microbiology & Infection prevention, Amsterdam University Medical Centers location AMC	Department of Medical Microbiology & Infection prevention, Amsterdam University Medical Centers location AMC	Matthijs Welkers, Jelle Koopsen, Robin van Houdt, Marcel Jonges, Sebastien Matamoros, Sjoerd Rebers, Fokla Zorgdrager, Sylvia Bruisten, Judith den Uil, Akke Cornelissen, Janke Schinkel, Menno de Jong, Gini van Rijckevorsel and Mariken van der Lubben on behalf of the Amsterdam Regional Genomic epidemiology and Outbreak Surveillance (ARGOS) consortium
EPI_ISL_13732932	Hosp. Sao Joaquim - Beneficiencia Portuguesa	Instituto Adolfo Lutz Strategic Laboratory	Claudio Tavares Sacchi, Karoline Rodrigues Campos, Ariadne Ferreira Amarante, Marlon Benedito Nascimento Santos, Alex Domingos Reis, Adriano Abbud, Adriana Bugno
EPI_ISL_13734233	Microbial Genomics, Hospital General Universitario Gregorio Maranon	Microbial Genomics, Hospital General Universitario Gregorio Maranon	Palomino-Cabrera,R., Penas-Utrilla,D., Buenestado-Serrano,S., Perez-Lago,L., Herranz Martin,M., Veintimilla,C., Catalan,P., Munoz,P. and Garcia de Viedma,D.
EPI_ISL_13734269	Department of Clinical Sciences, Institute of Tropica Medicine	Department of Clinical Sciences, Institute of Tropica Medicine	De Baetselier,I., Van Dijck,C., Kenyon,C., Coppens,J., Smet,H., de Block,T., Coppens,S., Vanroye,F., Bugert,J., Girl,P., Liesenborghs,L., Selhorst,P., Arien,K., Van den Bossche,D., Florence,E., Rezende,A.M., Vercauteren,K. and Van Esbroeck,M.
EPI_ISL_13744902	Department of Virology, Faculty of Medicine, University of Helsinki	Department of Virology, Faculty of Medicine, University of Helsinki	Kant,R., Smura,T., Vauhkonen,H. and Vapalahti,O.
EPI_ISL_13822667, EPI_ISL_13822668, EPI_ISL_13822669, EPI_ISL_13822718	Erasmus Medical Center Department of Virology	Erasmus Medical Center Department of Virology	Bas Oude Munnink, Marjan Boter, Babette Weller, Richard Molenkamp, Janette Rahamat-Langendoen, Reina Sikkema, Marion Koopmans
EPI_ISL_13827273, EPI_ISL_13827274, EPI_ISL_13827275, EPI_ISL_13827277, EPI_ISL_13827278, EPI_ISL_13827279, EPI_ISL_13827280, EPI_ISL_13827281, EPI_ISL_13827282	Public Health Agency of Canada, National Microbiology Laboratory	Public Health Agency of Canada, National Microbiology Laboratory	Duggan,A., Hole,D., Yadav,C., Knox,N., Haidl,E., Chapel,M., Domselaar,G.V., Fernando,L., Graham,M., Antonation,K., Audet,J., Hagan,M., Safronetz,D., Leung,A., Peters,G., Go,A., Laminman,V., Kaplen,B., Jolly,G., Marchand-Austin,A., Eshaghi,A., Patel,S.N., Hasso,M., Gubbay,J.B. and Olsha,R.
EPI_ISL_13833194, EPI_ISL_13833195, EPI_ISL_13833196, EPI_ISL_13833197	Laboratorio de Referencia Nacional de Virus Respiratorio. Centro Nacional de Salud Publica. Instituto Nacional de Salud.	Laboratorio de Referencia Nacional de Virus Respiratorio. Centro Nacional de Salud Publica. Instituto Nacional de Salud.	Carlos Padilla Rojas, Veronica Hurtado Vela, Iris Silva Molina, Luren Sevilla Castañeda, Victor Jimenez Vasquez, Orson Mestanza Millones, Luis Barcena Flores, Wendy Lizarraga Olivares, Alicia Nuñez Llanos, Steve Acedo Lazo, Francisco Ascue Orosco, Kelly Izarra Rojas, Princesa Medrano Alhuay, Karla Vasquez Cajachahua, Estela Huaman Angeles, Jorge Giraldo Chavez, Lilian Huarca Balbin, Lisbet Roxana Inga Angulo, Maria Sandra Villar Saavedra, Henri Bailon Calderon, Lely Solari Zerpa, Gloria Arotinco Garayar. Equipo de vigilancia genomica del Instituto Nacional de Salud.
EPI_ISL_13842269, EPI_ISL_13842548	Center for Virology, Medical University of Vienna	Medical University of Vienna Center for Virology	Jeremy V. Camp, Monika Redlberger-Fritz, Stephan W. Aberle
EPI_ISL_13889435, EPI_ISL_13889436, EPI_ISL_13889437, EPI_ISL_13889438, EPI_ISL_13889439, EPI_ISL_13889440, EPI_ISL_13889441, EPI_ISL_13889442, EPI_ISL_13889443, EPI_ISL_13889444, EPI_ISL_13889445, EPI_ISL_13889446, EPI_ISL_13889447, EPI_ISL_13889448, EPI_ISL_13889449, EPI_ISL_13889450, EPI_ISL_13889515, EPI_ISL_13889590, EPI_ISL_13889660, EPI_ISL_13889729, EPI_ISL_13889796, EPI_ISL_13889908, EPI_ISL_13889977, EPI_ISL_13890048, EPI_ISL_13890135, EPI_ISL_13890204, EPI_ISL_13890273, EPI_ISL_13890338, EPI_ISL_13890408, EPI_ISL_13890464, EPI_ISL_13890465, EPI_ISL_13890466, EPI_ISL_13890467, EPI_ISL_13890468, EPI_ISL_13890469, EPI_ISL_13890470, EPI_ISL_13890471, EPI_ISL_13890472, EPI_ISL_13890473, EPI_ISL_13890474, EPI_ISL_13890475, EPI_ISL_13890476, EPI_ISL_13890477, EPI_ISL_13890478, EPI_ISL_13890479, EPI_ISL_13890480, EPI_ISL_13890481, EPI_ISL_13890482			
see above	Charité Universitätsmedizin Berlin, Institut für Virologie/Labor Berlin	Charité Universitätsmedizin Berlin, Institut für Virologie	Terry C. Jones, Julia Schneider, Barbara Mühlemann, Talitha Veith, Jörn Beheim-Schwarzbach, Julia Tesch, Marie Luisa Schmidt, Felix Walper, Tobias Bleicker, Caroline Isner, Frieder Pfäfflin, Ricardo Niklas Werner, Victor M. Corman, Christian Drosten
EPI_ISL_13891126	Ministry of Health Turkey	Ministry of Health Turkey	Fatma Bayrakdar, Suleyman Yalcin, Gulay Korukluoglu
EPI_ISL_13908329, EPI_ISL_13908332, EPI_ISL_13908333, EPI_ISL_13908334, EPI_ISL_13908335, EPI_ISL_13908336, EPI_ISL_13908337, EPI_ISL_13908338, EPI_ISL_13908339, EPI_ISL_13908340, EPI_ISL_13908341, EPI_ISL_13908342, EPI_ISL_13908343, EPI_ISL_13908345			
see above	Public Health Agency of Canada, National Microbiology Laboratory	Public Health Agency of Canada, National Microbiology Laboratory	Duggan,A., Hole,D., Yadav,C., Knox,N., Chapel,M., Tyler,A., Haidl,E., Domselaar,G.V., Antonation,K., Audet,J., Fernando,L., Hagan,M., Safronetz,D., Graham,M., Peters,G., Go,A., Laminman,V., Kaplen,B., Leung,A., Jolly,G., Fafard,J., Charest,H. and Levade,I.
EPI_ISL_13953610, EPI_ISL_13953611	Indian Council of Medical Research-National Institute of Virology	Indian Council of Medical Research-National Institute of Virology	Pragya Yadav, Rima Sahay, Anita Aich Shete, Sreelekshmy Mohandas, Priya Abraham
EPI_ISL_13955501	Public Health Authority of the Slovak Republic	Laboratory of Genomics and Bioinformatics, Comenius University Science Park	Tomáš Szemes, Edita Staroňová, Elena Tichá, Lucia Ševčíková, Terézia Vrabľová, Tatiana Sedláčková, Miroslav Böhmer, Jaroslav Budiš, Pavol Mišenko
EPI_ISL_13958697	Research and Evaluation, UKHSA	Research and Evaluation, UKHSA	Groves,N., Osman,K.L., Lewandowski,K.S., Carter,D.P., Pullan,S.T., Myers,R., Vipond,R. and Chand,M.
EPI_ISL_13983354, EPI_ISL_13983355	Instituto de Infectologia Emilio Ribas	Instituto Adolfo Lutz Strategic Laboratory	Claudio Tavares Sacchi, Karoline Rodrigues Campos, Ariadne Ferreira Amarante, Marlon Benedito Nascimento Santos, Alex Domingos Reis, Adriano Abbud, Adriana Bugno
EPI_ISL_13983356	INSPI-Centro de Referencia Nacional de Virus Exantemáticos, Gastroentéricos y Transmitido por Vectores.	INSPI-Dirección Técnica de Investigación, Desarrollo e Innovación INSPI-Centro de Referencia Nacional de Genómica, Secuenciación y Bioinformática	Andrés Carrazco-Montalvo, Diana Gutiérrez, Naomi Mora, Silvia Salgado-Cisneros, Johana Parrales-Valdiviezo, Martha Sánchez-Domenech, Diego Morales, Gulnara Borja-Cabrera, Leandro Patiño*.
EPI_ISL_13983888	Bangkok Hospital Phuket	National Institute of Health, Department of Medical Sciences, Ministry of Public Health, Thailand	Pilailuk Okada; Siripaporn Phuygun; Nuttida Thongpramul; Thanutsapa Thanadachakul; Kazuhisa Okada; Archawin Rojanawiwat; Chakkarat Pitayawonganon; Supakit Sirilak
EPI_ISL_13993734, EPI_ISL_13993735, EPI_ISL_13993737, EPI_ISL_13993738, EPI_ISL_13993739	California Department of Public Health	California Department of Public Health	Viral and Rickettsial Disease Laboratory
EPI_ISL_14003930	University of Rochester Medical Center	University of Rochester Medical Center	Andrew Cameron, Mondraya Howard, Sara Connelly, Dwight Hardy, Kelly DeLary
EPI_ISL_14011193	Bangkok Hospital Phuket	Thai Red Cross Emerging Infectious Diseases Clinical Center and Faculty of Medicine, Chulalongkorn University	Kusak Kukiattikoon, Waritta Dararattanaroj, Rome Buathong, Supaporn Wacharapluesadee, Sininat Petcharat, Ananporn Supataragul, Stefan Fernandez, Achawin Rojanaviwat, Chonticha Klungthong, Pilailuk Okada, Khajohn Joonlasak, Chakkarat Pitayawonganon, Opass Putcharoen
EPI_ISL_14021725	Hosp. Municipal Enf. Antonio Policarpo de Oliveira	Instituto Adolfo Lutz Strategic Laboratory	Claudio Tavares Sacchi, Karoline Rodrigues Campos, Ariadne Ferreira Amarante, Marlon Benedito Nascimento Santos, Alex Domingos Reis, Adriano Abbud, Adriana Bugno
EPI_ISL_14049244, EPI_ISL_14049245	Indian Council of Medical Research-National Institute of Virology	Indian Council of Medical Research-National Institute of Virology	Pragya Yadav, Rima Sahay, Anita Aich Shete, Sreelekshmy Mohandas, Priya Abraham
EPI_ISL_14050451, EPI_ISL_14050453, EPI_ISL_14050454, EPI_ISL_14050455, EPI_ISL_14050456, EPI_ISL_14050458	Public Health Agency of Canada, National Microbiology Laboratory	Public Health Agency of Canada, National Microbiology Laboratory	Duggan,A., Hole,D., Yadav,C., Knox,N., Tyler,A., Haidl,E., Chapel,M., Domselaar,G.V., Graham,M., Audet,J., Fernando,L., Hagan,M., Safronetz,D., Leung,A., Peters,G., Go,A., Laminman,V., Kaplen,B., Antonation,K., Jolly,G., Griffiths,E., Charest,H., Levade,I. and Fafard,J.
EPI_ISL_14070493, EPI_ISL_14070852, EPI_ISL_14070854, EPI_ISL_14070855	Instituto de Infectologia Emilio Ribas	Instituto Adolfo Lutz Strategic Laboratory	Claudio Tavares Sacchi, Karoline Rodrigues Campos, Ariadne Ferreira Amarante, Marlon Benedito Nascimento Santos, Alex Domingos Reis, Adriano Abbud, Adriana Bugno
EPI_ISL_14153982	Vajira Hospital	National Institute of Health, Department of Medical Sciences, Ministry of Public Health, Thailand	Pilailuk Okada; Siripaporn Phuygun; Nuttida Thongpramul; Thanutsapa Thanadachakul; Kazuhisa Okada; Archawin Rojanawiwat; Chakkarat Pitayawonganon; Supakit Sirilak
EPI_ISL_14166709	Medical University of Vienna Center for Virology	Medical University of Vienna Center for Virology	Jeremy V Camp, Monika Redlberger-Fritz, Stephan W. Aberle
EPI_ISL_14167248, EPI_ISL_14167573, EPI_ISL_14167574, EPI_ISL_14167575	Medical University of Vienna Center for Virology	Medical University of Vienna Center for Virology	Jeremy V. Camp, Monika Redlberger-Fritz, Stephan W. Aberle
EPI_ISL_14170201	Erasmus Medical Center Department of Virology	Erasmus Medical Center Department of Virology	Bas Oude Munnink, Marjan Boter, Babette Weller, Babs Verstrepen, Richard Molenkamp, Janette Rahamat-Langendoen, Reina Sikkema, Marion Koopmans
EPI_ISL_14189016	Los Angeles County Public Health Laboratories	Los Angeles County Public Health Laboratories	P. Hemarajata et al.
EPI_ISL_14207724, EPI_ISL_14207725, EPI_ISL_14207726, EPI_ISL_14207727, EPI_ISL_14207728, EPI_ISL_14207729, EPI_ISL_14207730, EPI_ISL_14207731, EPI_ISL_14207732, EPI_ISL_14207733, EPI_ISL_14207734, EPI_ISL_14207735, EPI_ISL_14207736, EPI_ISL_14207737, EPI_ISL_14207738, EPI_ISL_14207739, EPI_ISL_14207740, EPI_ISL_14207741			
see above	Laboratorio de Referencia Nacional de Virus Respiratorio. Centro Nacional de Salud Publica. Instituto Nacional de Salud.	Laboratorio de Referencia Nacional de Virus Respiratorio. Centro Nacional de Salud Publica. Instituto Nacional de Salud.	Carlos Padilla Rojas, Veronica Hurtado Vela, Iris Silva Molina, Luren Sevilla Castañeda, Victor Jimenez Vasquez, Orson Mestanza Millones, Luis Barcena Flores, Wendy Lizarraga Olivares, Alicia Nuñez Llanos, Steve Acedo Lazo, Francisco Ascue Orosco, Kelly Izarra Rojas, Princesa Medrano Alhuay, Karla Vasquez Cajachahua, Estela Huaman Angeles, Jorge Giraldo Chavez, Lilian Huarca Balbin, Lisbet Roxana Inga Angulo, Maria Sandra Villar Saavedra, Henri Bailon Calderon, Lely Solari Zerpa, Gloria Arotinco Garayar. Equipo de vigilancia genomica del Instituto Nacional de Salud.
EPI_ISL_14211644, EPI_ISL_14211645	Public Health Authority of the Slovak Republic	Laboratory of Genomics and Bioinformatics, Comenius University Science Park	Tomáš Szemes, Edita Staroňová, Elena Tichá, Lucia Ševčíková, Terézia Vrabľová, Tatiana Sedláčková, Miroslav Böhmer, Jaroslav Budiš, Pavol Mišenko
EPI_ISL_14224334	Genetica Molecular and Subdepartamento de Virologia ISP Chile	Instituto de Salud Publica de Chile	Paulo C. Covarrubias, Andrés E. Castillo, Constanza Campano, Mariela Guajardo, Bárbara Parra, Rodrigo Fasce Pineda, Jorge Fernández
EPI_ISL_14251112	University of Rochester Medical Center	University of Rochester Medical Center	Andrew Cameron, Mondraya Howard, Joel Maki, Sara Connelly, Kelly Delary, Dwight Hardy
EPI_ISL_14254435, EPI_ISL_14254436, EPI_ISL_14254437, EPI_ISL_14254438	Erasmus Medical Center Department of Virology	Erasmus Medical Center Department of Virology	Bas Oude Munnink, Marjan Boter, Babette Weller, Babs Verstrepen, Richard Molenkamp, Janette Rahamat-Langendoen, Reina Sikkema, Marion Koopmans
EPI_ISL_14295679	Bangkok Hospital Phuket	National Institute of Health, Department of Medical Sciences, Ministry of Public Health, Thailand	Pilailuk Okada; Siripaporn Phuygun; Nuttida Thongpramul; Thanutsapa Thanadachakul; Kazuhisa Okada; Archawin Rojanawiwat; Chakkarat Pitayawonganon; Supakit Sirilak
EPI_ISL_14326638, EPI_ISL_14326639, EPI_ISL_14326640, EPI_ISL_14326641, EPI_ISL_14326642, EPI_ISL_14326643	Environmental, Agricultural, and Occupational Health, University of Nebraska Medical Center, 984388 Nebraska Medical Center	Environmental, Agricultural, and Occupational Health, University of Nebraska Medical Center, 984388 Nebraska Medical Center	Tegomoh,B., Cross,S.T., Chapman,R.C., Bernhard,K., McCutchen,E.L., Fauver,J.R., Pratt,C.B., Warden,D.E., Iwen,P.C., Donahue,M. and Wiley,M.R.
EPI_ISL_14326644	Environmental, Agricultural, and Occupational Health, University of Nebraska Medical Center, 984388 Nebraska Medical Center	Environmental, Agricultural, and Occupational Health, University of Nebraska Medical Center, 984388 Nebraska Medical Center	Tegomoh,B., Cross,S.T., Chapman,R.C., Bernhard,K., McCutchen,E.L., Fauver,J.R., Pratt,C.B., Warden,D.E., Iwen,P.C., Donahue,M. and Wiley,M.R
EPI_ISL_14414948	UMS Parque Industrial Curitiba	Instituto Adolfo Lutz Strategic Laboratory	Claudio Tavares Sacchi, Karoline Rodrigues Campos, Ariadne Ferreira Amarante, Marlon Benedito Nascimento Santos, Alex Domingos Reis, Adriano Abbud, Adriana Bugno
EPI_ISL_14415810	CTA Sao Miguel	Instituto Adolfo Lutz Strategic Laboratory	Claudio Tavares Sacchi, Karoline Rodrigues Campos, Ariadne Ferreira Amarante, Marlon Benedito Nascimento Santos, Alex Domingos Reis, Adriano Abbud, Adriana Bugno
EPI_ISL_14439712, EPI_ISL_14439713, EPI_ISL_14439714, EPI_ISL_14439715, EPI_ISL_14439716, EPI_ISL_14439717, EPI_ISL_14439718, EPI_ISL_14439719, EPI_ISL_14439720, EPI_ISL_14439721, EPI_ISL_14439722, EPI_ISL_14439723, EPI_ISL_14439724, EPI_ISL_14439725, EPI_ISL_14439726, EPI_ISL_14439727, EPI_ISL_14439728, EPI_ISL_14439729, EPI_ISL_14439730, EPI_ISL_14439731, EPI_ISL_14439732, EPI_ISL_14439733, EPI_ISL_14439734, EPI_ISL_14439735, EPI_ISL_14439736, EPI_ISL_14439737, EPI_ISL_14439738, EPI_ISL_14439739, EPI_ISL_14439740, EPI_ISL_14439741, EPI_ISL_14439742, EPI_ISL_14439743, EPI_ISL_14439745, EPI_ISL_14439746, EPI_ISL_14439747, EPI_ISL_14439748, EPI_ISL_14439749, EPI_ISL_14439750, EPI_ISL_14439751, EPI_ISL_14439752, EPI_ISL_14439753, EPI_ISL_14439754, EPI_ISL_14439755, EPI_ISL_14439756, EPI_ISL_14439757, EPI_ISL_14439758, EPI_ISL_14439759, EPI_ISL_14439760, EPI_ISL_14439761, EPI_ISL_14439762, EPI_ISL_14439763, EPI_ISL_14439764, EPI_ISL_14439765, EPI_ISL_14439766, EPI_ISL_14439767, EPI_ISL_14439768, EPI_ISL_14439769, EPI_ISL_14439770, EPI_ISL_14439771, EPI_ISL_14439772,			

EPI_ISL_14439773, EPI_ISL_14439774, EPI_ISL_14439775, EPI_ISL_14439776, EPI_ISL_14439777, EPI_ISL_14439778, EPI_ISL_14439779, EPI_ISL_14439780, EPI_ISL_14439781, EPI_ISL_14439782, EPI_ISL_14439783, EPI_ISL_14439784, EPI_ISL_14439785			
see above	Research and Evaluation, UKHSA	Research and Evaluation, UKHSA	Groves,N., Osman,K.L., Lewandowski,K.S., Carter,D.P., Pullan,S.T., Myers,R., Vipond,R. and Chand,M.
EPI_ISL_14445098, EPI_ISL_14445100, EPI_ISL_14445101, EPI_ISL_14445102, EPI_ISL_14445103, EPI_ISL_14445104, EPI_ISL_14445107, EPI_ISL_14445109, EPI_ISL_14445111, EPI_ISL_14445113, EPI_ISL_14445114, EPI_ISL_14445115, EPI_ISL_14445116, EPI_ISL_14445117, EPI_ISL_14445118, EPI_ISL_14445119, EPI_ISL_14445120, EPI_ISL_14445121, EPI_ISL_14445122, EPI_ISL_14445123, EPI_ISL_14445124, EPI_ISL_14445125, EPI_ISL_14445126, EPI_ISL_14445127, EPI_ISL_14445128, EPI_ISL_14445129, EPI_ISL_14445130, EPI_ISL_14445131, EPI_ISL_14445132, EPI_ISL_14445133, EPI_ISL_14445134, EPI_ISL_14445135, EPI_ISL_14445136, EPI_ISL_14445137, EPI_ISL_14445138, EPI_ISL_14445139, EPI_ISL_14445140, EPI_ISL_14445141, EPI_ISL_14445142, EPI_ISL_14445143, EPI_ISL_14445144, EPI_ISL_14445145, EPI_ISL_14445146, EPI_ISL_14445147, EPI_ISL_14445148, EPI_ISL_14445149, EPI_ISL_14445150, EPI_ISL_14445151, EPI_ISL_14445152, EPI_ISL_14445153			
see above	Laboratorio de Referencia Nacional de Virus Respiratorio. Centro Nacional de Salud Publica. Instituto Nacional de Salud.	Laboratorio de Referencia Nacional de Virus Respiratorio. Centro Nacional de Salud Publica. Instituto Nacional de Salud.	Carlos Padilla Rojas, Veronica Hurtado Vela, Iris Silva Molina, Luren Sevilla Castañeda, Victor Jimenez Vasquez, Orson Mestanza Millones, Luis Barcena Flores, Wendy Lizarraga Olivares, Alicia Nuñez Llanos, Steve Acedo Lazo, Francisco Ascue Orosco, Kelly Izarra Rojas, Princesa Medrano Alhuay, Karla Vasquez Cajachahua, Estela Huaman Angeles, Jorge Giraldo Chavez, Lilian Huarca Balbin, Lisbet Roxana Inga Angulo, Maria Sandra Villar Saavedra, Henri Bailon Calderon, Lely Solari Zerpa, Gloria Arotinco Garayar. Equipo de vigilancia genomica del Instituto Nacional de Salud.
EPI_ISL_14465517	Centro de Desenvolvimento Científico e Tecnológico (CDCT), Centro Estadual de Vigilância em Saúde (CEVS) da Secretaria Estadual da Saúde (SES-RS)	Centro de Desenvolvimento Científico e Tecnológico (CDCT), Centro Estadual de Vigilância em Saúde (CEVS) da Secretaria Estadual da Saúde (SES-RS)	Richard Steiner Salvato, Regina Bones Barcellos, Fernanda Marques Godinho
EPI_ISL_14467428, EPI_ISL_14467429	Laboratório Central de Saúde Pública do Amazonas - LACEN-AM	Laboratório de Ecologia de Doenças Transmissíveis na Amazônia, Instituto Leônidas e Maria Deane - Fiocruz Amazônia	Victor Souza, Fernanda Nascimento, Matilde Mejía, Dejanane Silva, Luciana Gonçalves, Tatyana Costa Amorim Ramos, Ana Ruth Lima Arcanjo, Valdinete Nascimento, Felipe Naveca on behalf of the Fiocruz COVID-19 Genomic Surveillance Network
EPI_ISL_14515173, EPI_ISL_14515174, EPI_ISL_14515182, EPI_ISL_14515186, EPI_ISL_14515187, EPI_ISL_14515188, EPI_ISL_14515193, EPI_ISL_14515201, EPI_ISL_14515203, EPI_ISL_14515210, EPI_ISL_14515219, EPI_ISL_14515220			
see above	Department of Infectious Diseases, National Institute of Health Doutor Ricardo Jorge, Portugal (INSA)	Department of Infectious Diseases, National Institute of Health Doutor Ricardo Jorge, Portugal (INSA)	Isidro,J., Borges,V., Pinto,M., Sobral,D., Santos,J., Nunes,A., Mixao,V., Ferreira,R., Santos,D., Duarte,S., Vieira,L., Borrego,M.J., Nuncio,S., Lopes de Carvalho,I., Pelerito,A., Cordeiro,R. and Gomes,J.P.
EPI_ISL_14526939, EPI_ISL_14526940, EPI_ISL_14526942, EPI_ISL_14526943, EPI_ISL_14526944, EPI_ISL_14526945, EPI_ISL_14526948, EPI_ISL_14526949, EPI_ISL_14526950, EPI_ISL_14526952, EPI_ISL_14526953, EPI_ISL_14526954, EPI_ISL_14526955, EPI_ISL_14526956			
see above	Connecticut Department of Public Health	Grubaugh Lab - Yale School of Public Health	Nicholas F. G. Chen, Chrispin Chaguza, Kien Pham, Nathan D. Grubaugh, Christina Nishimura, Claire Pearson, Kutluhan Incekara, Jian Ping Huang, Emily Gagnon, Ethan Reever, Jafar Razeq, Anthony Muyombwe, Chantal B. F. Vogels
EPI_ISL_14541645, EPI_ISL_14541647, EPI_ISL_14541649, EPI_ISL_14541652, EPI_ISL_14541654	Public Health Authority of the Slovak Republic	Laboratory of Genomics and Bioinformatics, Comenius University Science Park	Tomáš Szemes, Edita Staroňová, Elena Tichá, Lucia Ševčíková, Terézia Vrabľová, Tatiana Sedláčková, Miroslav Böhmer, Jaroslav Budiš, Pavol Mišenko
EPI_ISL_14561924	Los Angeles County Public Health Laboratories	Los Angeles County Public Health Laboratories	P. Hemarajata et al.
EPI_ISL_14571429	Hosp. Municipal Dr. Jose de Carvalho Florence	Instituto Adolfo Lutz Strategic Laboratory	Claudio Tavares Sacchi, Karoline Rodrigues Campos, Ariadne Ferreira Amarante, Marlon Benedito Nascimento Santos, Alex Domingos Reis, Adriano Abbud, Adriana Bugno
EPI_ISL_14571433	Casa de Saude Stella Maris	Instituto Adolfo Lutz Strategic Laboratory	Claudio Tavares Sacchi, Karoline Rodrigues Campos, Ariadne Ferreira Amarante, Marlon Benedito Nascimento Santos, Alex Domingos Reis, Adriano Abbud, Adriana Bugno
EPI_ISL_14571435	Secrtetaria Municipal de Saude de Sertaozinho	Instituto Adolfo Lutz Strategic Laboratory	Claudio Tavares Sacchi, Karoline Rodrigues Campos, Ariadne Ferreira Amarante, Marlon Benedito Nascimento Santos, Alex Domingos Reis, Adriano Abbud, Adriana Bugno
EPI_ISL_14571439	Secrtetaria Municipal de Saude de Sata Barbara D Oeste	Instituto Adolfo Lutz Strategic Laboratory	Claudio Tavares Sacchi, Karoline Rodrigues Campos, Ariadne Ferreira Amarante, Marlon Benedito Nascimento Santos, Alex Domingos Reis, Adriano Abbud, Adriana Bugno
EPI_ISL_14571441	Hosp. Municipal Dr. Waldemar Tebaldi	Instituto Adolfo Lutz Strategic Laboratory	Claudio Tavares Sacchi, Karoline Rodrigues Campos, Ariadne Ferreira Amarante, Marlon Benedito Nascimento Santos, Alex Domingos Reis, Adriano Abbud, Adriana Bugno
EPI_ISL_14571442	Instituto de Infectologia Emilio Ribas II Baixada Santista	Instituto Adolfo Lutz Strategic Laboratory	Claudio Tavares Sacchi, Karoline Rodrigues Campos, Ariadne Ferreira Amarante, Marlon Benedito Nascimento Santos, Alex Domingos Reis, Adriano Abbud, Adriana Bugno
EPI_ISL_14571444	UBDS DR. Italo Baruffi Castelo Branco	Instituto Adolfo Lutz Strategic Laboratory	Claudio Tavares Sacchi, Karoline Rodrigues Campos, Ariadne Ferreira Amarante, Marlon Benedito Nascimento Santos, Alex Domingos Reis, Adriano Abbud, Adriana Bugno
EPI_ISL_14584274, EPI_ISL_14584275, EPI_ISL_14584276, EPI_ISL_14584277, EPI_ISL_14584278, EPI_ISL_14584279, EPI_ISL_14584281, EPI_ISL_14584282, EPI_ISL_14584283, EPI_ISL_14584284, EPI_ISL_14584286, EPI_ISL_14584287, EPI_ISL_14584289, EPI_ISL_14584290, EPI_ISL_14584291, EPI_ISL_14584292, EPI_ISL_14584293, EPI_ISL_14584294, EPI_ISL_14584295, EPI_ISL_14584296, EPI_ISL_14584297, EPI_ISL_14584298, EPI_ISL_14584299, EPI_ISL_14584300, EPI_ISL_14584301, EPI_ISL_14584302, EPI_ISL_14584303, EPI_ISL_14584304, EPI_ISL_14584305, EPI_ISL_14584306, EPI_ISL_14584307, EPI_ISL_14584308, EPI_ISL_14584309, EPI_ISL_14584310, EPI_ISL_14584311			
see above	Laboratorio de Referencia Nacional de Virus Respiratorio. Centro Nacional de Salud Publica. Instituto Nacional de Salud.	Laboratorio de Referencia Nacional de Virus Respiratorio. Centro Nacional de Salud Publica. Instituto Nacional de Salud.	Carlos Padilla Rojas, Veronica Hurtado Vela, Iris Silva Molina, Luren Sevilla Castañeda, Victor Jimenez Vasquez, Orson Mestanza Millones, Luis Barcena Flores, Wendy Lizarraga Olivares, Alicia Nuñez Llanos, Steve Acedo Lazo, Francisco Ascue Orosco, Kelly Izarra Rojas, Princesa Medrano Alhuay, Karla Vasquez Cajachahua, Estela Huaman Angeles, Jorge Giraldo Chavez, Lilian Huarca Balbin, Lisbet Roxana Inga Angulo, Maria Sandra Villar Saavedra, Henri Bailon Calderon, Lely Solari Zerpa, Gloria Arotinco Garayar. Equipo de vigilancia genomica del Instituto Nacional de Salud.
EPI_ISL_14586688	Public Health Authority of the Slovak Republic	Laboratory of Genomics and Bioinformatics, Comenius University Science Park	Tomáš Szemes, Edita Staroňová, Elena Tichá, Lucia Ševčíková, Terézia Vrabľová, Tatiana Sedláčková, Miroslav Böhmer, Jaroslav Budiš, Pavol Mišenko
EPI_ISL_14587544, EPI_ISL_14587545, EPI_ISL_14587546, EPI_ISL_14587548, EPI_ISL_14587549, EPI_ISL_14587550, EPI_ISL_14587551	Public Health Agency of Canada, National Microbiology Laboratory	Public Health Agency of Canada, National Microbiology Laboratory	Duggan,A., Hole,D., Yadav,C., Knox,N., Tyler,A., Haidl,E., Chapel,M., Domselaar,G.V., Graham,M., Audet,J., Fernando,L., Hagan,M., Safronetz,D., Leung,A., Peters,G., Go,A., Laminman,V., Kaplen,B., Antonation,K., Griffiths,E., Jolly,G., Charest,H., Levade,I. and Fafard,J.
EPI_ISL_14594041, EPI_ISL_14594042, EPI_ISL_14594043, EPI_ISL_14594047, EPI_ISL_14594049, EPI_ISL_14594050, EPI_ISL_14594051, EPI_ISL_14594052, EPI_ISL_14594053, EPI_ISL_14594054, EPI_ISL_14594055, EPI_ISL_14594056			
see above	Public Health Agency of Canada, National Microbiology Laboratory	Public Health Agency of Canada, National Microbiology Laboratory	Duggan,A., Hole,D., Yadav,C., Knox,N., Tyler,A., Haidl,E., Chapel,M., Domselaar,G.V., Graham,M., Audet,J., Fernando,L., Antonation,K., Safronetz,D., Hagan,M., Peters,G., Go,A., Laminman,V., Kaplen,B., Leung,A., Griffiths,E., Jolly,G., Eshaghi,A., Gubbay,J.B., Hasso,M., Marchand-Austin,A., Olsha,R. and Patel,S.N.
EPI_ISL_14615579	RSUPN dr. Cipto Mangunkusumo	National Institute of Health Research and Development	Hana Apsari Pawestri, Arie Ardiansyah Nugraha, Fajar Nur Sulistiyohadi, Subangkit, Krisna NA Pangesti, Tze Minn Mak, I Gede Made Wirabrata
EPI_ISL_14621526	Virology, APHP Pitie Salpetriere SU	Virology, APHP Pitie Salpetriere SU	Seang,S., Burrel,S., Todesco,E., Leducq,V., Monsel,G., Le Pluart,D., Cordevant,C., Pourcher,V. and Palich,R.
EPI_ISL_14622055	Instituto de Infectologia Emilio Ribas	Instituto Adolfo Lutz Strategic Laboratory	Claudio Tavares Sacchi, Karoline Rodrigues Campos, Ariadne Ferreira Amarante, Marlon Benedito Nascimento Santos, Alex Domingos Reis, Adriano Abbud, Adriana Bugno
EPI_ISL_14622520	UBS Jovaia	Instituto Adolfo Lutz Strategic Laboratory	Claudio Tavares Sacchi, Karoline Rodrigues Campos, Ariadne Ferreira Amarante, Marlon Benedito Nascimento Santos, Alex Domingos Reis, Adriano Abbud, Adriana Bugno
EPI_ISL_14622705	UBS Jardim Santista	Instituto Adolfo Lutz Strategic Laboratory	Claudio Tavares Sacchi, Karoline Rodrigues Campos, Ariadne Ferreira Amarante, Marlon Benedito Nascimento Santos, Alex Domingos Reis, Adriano Abbud, Adriana Bugno
EPI_ISL_14622706	Centro de Referencia Modulo I SAE II Bauru	Instituto Adolfo Lutz Strategic Laboratory	Claudio Tavares Sacchi, Karoline Rodrigues Campos, Ariadne Ferreira Amarante, Marlon Benedito Nascimento Santos, Alex Domingos Reis, Adriano Abbud, Adriana Bugno
EPI_ISL_14622707	USF Boicucanga I Sao Sebastiao	Instituto Adolfo Lutz Strategic Laboratory	Claudio Tavares Sacchi, Karoline Rodrigues Campos, Ariadne Ferreira Amarante, Marlon Benedito Nascimento Santos, Alex Domingos Reis, Adriano Abbud, Adriana Bugno
EPI_ISL_14622913	Secretaria Municipal de Saude de Caxias do Sul	Instituto Adolfo Lutz Strategic Laboratory	Claudio Tavares Sacchi, Karoline Rodrigues Campos, Ariadne Ferreira Amarante, Marlon Benedito Nascimento Santos, Alex Domingos Reis, Adriano Abbud, Adriana Bugno
EPI_ISL_14622953	Sistema de Vigilancia em Saude Viamao	Instituto Adolfo Lutz Strategic Laboratory	Claudio Tavares Sacchi, Karoline Rodrigues Campos, Ariadne Ferreira Amarante, Marlon Benedito Nascimento Santos, Alex Domingos Reis, Adriano Abbud, Adriana Bugno
EPI_ISL_14622960	Vigilancia Epidemiologica Municipal	Instituto Adolfo Lutz Strategic Laboratory	Claudio Tavares Sacchi, Karoline Rodrigues Campos, Ariadne Ferreira Amarante, Marlon Benedito Nascimento Santos, Alex Domingos Reis, Adriano Abbud, Adriana Bugno
EPI_ISL_14623175	Centro de Referencia em Especialidades Central Rib Preto	Instituto Adolfo Lutz Strategic Laboratory	Claudio Tavares Sacchi, Karoline Rodrigues Campos, Ariadne Ferreira Amarante, Marlon Benedito Nascimento Santos, Alex Domingos Reis, Adriano Abbud, Adriana Bugno
EPI_ISL_14623523	Laboratorio Municipal de Piracicaba	Instituto Adolfo Lutz Strategic Laboratory	Claudio Tavares Sacchi, Karoline Rodrigues Campos, Ariadne Ferreira Amarante, Marlon Benedito Nascimento Santos, Alex Domingos Reis, Adriano Abbud, Adriana Bugno
EPI_ISL_14623704	Unidade Basica de Saude Esplanada	Instituto Adolfo Lutz Strategic Laboratory	Claudio Tavares Sacchi, Karoline Rodrigues Campos, Ariadne Ferreira Amarante, Marlon Benedito Nascimento Santos, Alex Domingos Reis, Adriano Abbud, Adriana Bugno
EPI_ISL_14624411	Hospital Albert Sabin Atibaia	Instituto Adolfo Lutz Strategic Laboratory	Claudio Tavares Sacchi, Karoline Rodrigues Campos, Ariadne Ferreira Amarante, Marlon Benedito Nascimento Santos, Alex Domingos Reis, Adriano Abbud, Adriana Bugno
EPI_ISL_14624610	USAFA Forte	Instituto Adolfo Lutz Strategic Laboratory	Claudio Tavares Sacchi, Karoline Rodrigues Campos, Ariadne Ferreira Amarante, Marlon Benedito Nascimento Santos, Alex Domingos Reis, Adriano Abbud, Adriana Bugno
EPI_ISL_14624698	Centro de Referencia em AIDS SECRAIDS	Instituto Adolfo Lutz Strategic Laboratory	Claudio Tavares Sacchi, Karoline Rodrigues Campos, Ariadne Ferreira Amarante, Marlon Benedito Nascimento Santos, Alex Domingos Reis, Adriano Abbud, Adriana Bugno
EPI_ISL_14624832	Servico de Vigilancia Epidemiologica e de Zoonoses do Guaruja	Instituto Adolfo Lutz Strategic Laboratory	Claudio Tavares Sacchi, Karoline Rodrigues Campos, Ariadne Ferreira Amarante, Marlon Benedito Nascimento Santos, Alex Domingos Reis, Adriano Abbud, Adriana Bugno
EPI_ISL_14624915	SMS Aruja	Instituto Adolfo Lutz Strategic Laboratory	Claudio Tavares Sacchi, Karoline Rodrigues Campos, Ariadne Ferreira Amarante, Marlon Benedito Nascimento Santos, Alex Domingos Reis, Adriano Abbud, Adriana Bugno
EPI_ISL_14625156	Secretaria Municipal de Saude de Suzano	Instituto Adolfo Lutz Strategic Laboratory	Claudio Tavares Sacchi, Karoline Rodrigues Campos, Ariadne Ferreira Amarante, Marlon Benedito Nascimento Santos, Alex Domingos Reis, Adriano Abbud, Adriana Bugno
EPI_ISL_14625157	PSF Vila Nossa Senhora de Fatima Fartura	Instituto Adolfo Lutz Strategic Laboratory	Claudio Tavares Sacchi, Karoline Rodrigues Campos, Ariadne Ferreira Amarante, Marlon Benedito Nascimento Santos, Alex Domingos Reis, Adriano Abbud, Adriana Bugno
EPI_ISL_14625190	Ambulatorio de Atendimentode DST de Guariba	Instituto Adolfo Lutz Strategic Laboratory	Claudio Tavares Sacchi, Karoline Rodrigues Campos, Ariadne Ferreira Amarante, Marlon Benedito Nascimento Santos, Alex Domingos Reis, Adriano Abbud, Adriana Bugno
EPI_ISL_14625230	UBS Centro Clair Aparecida Pavan	Instituto Adolfo Lutz Strategic Laboratory	Claudio Tavares Sacchi, Karoline Rodrigues Campos, Ariadne Ferreira Amarante, Marlon Benedito Nascimento Santos, Alex Domingos Reis, Adriano Abbud, Adriana Bugno
EPI_ISL_14625256	UMS Campina do Siqueira	Instituto Adolfo Lutz Strategic Laboratory	Claudio Tavares Sacchi, Karoline Rodrigues Campos, Ariadne Ferreira Amarante, Marlon Benedito Nascimento Santos, Alex Domingos Reis, Adriano Abbud, Adriana Bugno
EPI_ISL_14625282	Hospital Edmundo Vasconcelos	Instituto Adolfo Lutz Strategic Laboratory	Claudio Tavares Sacchi, Karoline Rodrigues Campos, Ariadne Ferreira Amarante, Marlon Benedito Nascimento Santos, Alex Domingos Reis, Adriano Abbud, Adriana Bugno
EPI_ISL_14666780	Public Health Authority of the Slovak Republic	Laboratory of Genomics and Bioinformatics, Comenius University Science Park	Tomáš Szemes, Edita Staroňová, Elena Tichá, Lucia Ševčíková, Terézia Vrabľová, Tatiana Sedláčková, Miroslav Böhmer, Jaroslav Budiš, Pavol Mišenko
EPI_ISL_14676265	Centro de Desenvolvimento Científico e Tecnológico (CDCT)/CEVS/SES-RS	Centro de Desenvolvimento Científico e Tecnológico (CDCT)/CEVS/SES-RS	Richard Steiner Salvato, Regina Bones Barcellos, Fernanda Marques Godinho
EPI_ISL_14707250	Charité Universitätsmedizin Berlin, Institut für Virologie	Charité Universitätsmedizin Berlin, Institut für Virologie	Julia Schneider, Victor M Corman, Terry C Jones, Christian Drosten
EPI_ISL_14721255, EPI_ISL_14721256, EPI_ISL_14721259, EPI_ISL_14721262, EPI_ISL_14721263, EPI_ISL_14721264, EPI_ISL_14721265	National Public Health Laboratory, National Centre for Infectious Diseases	National Public Health Laboratory, National Centre for Infectious Diseases	Yichen Ding, Benny Yeo, Daniel Lim, Zhenyang Zhou, Royce Ang, Samuel Loo, Lin Cui, Raymond Tzer Pin Lin
EPI_ISL_14736400, EPI_ISL_14736402, EPI_ISL_14736403	California Department of Public Health	California Department of Public Health	Viral and Rickettsial Disease Laboratory
EPI_ISL_14752090, EPI_ISL_14752091,	Environmental, Agricultural, and Occupational Health,	Environmental, Agricultural, and Occupational Health,	Tegomoh,B., Cross,S.T., Chapman,R.C., Bernhard,K., McCutchen,E.L., Fauver,J.R., Pratt,C.B., Warden,D.E., Iwen,P.C., Donahue,M. and Wiley,M.R.

EPI_ISL_14752093, EPI_ISL_14752094, EPI_ISL_14752096	University of Nebraska Medical Center	University of Nebraska Medical Center	
EPI_ISL_14752098, EPI_ISL_14752100, EPI_ISL_14752106, EPI_ISL_14752108, EPI_ISL_14752109, EPI_ISL_14752111, EPI_ISL_14752124, EPI_ISL_14752126, EPI_ISL_14752127, EPI_ISL_14752128, EPI_ISL_14752129, EPI_ISL_14752130, EPI_ISL_14752131, EPI_ISL_14752132, EPI_ISL_14752133, EPI_ISL_14752145, EPI_ISL_14752146, EPI_ISL_14752148, EPI_ISL_14752156, EPI_ISL_14752165, EPI_ISL_14752167, EPI_ISL_14752169, EPI_ISL_14752173, EPI_ISL_14752177, EPI_ISL_14752178, EPI_ISL_14752180, EPI_ISL_14752182, EPI_ISL_14752186, EPI_ISL_14752189, EPI_ISL_14752191, EPI_ISL_14752193, EPI_ISL_14752195, EPI_ISL_14752202, EPI_ISL_14752204, EPI_ISL_14752206, EPI_ISL_14752208, EPI_ISL_14752211, EPI_ISL_14752212, EPI_ISL_14752213, EPI_ISL_14752216, EPI_ISL_14752218, EPI_ISL_14752219, EPI_ISL_14752222, EPI_ISL_14752224, EPI_ISL_14752229, EPI_ISL_14752231, EPI_ISL_14752233, EPI_ISL_14752242, EPI_ISL_14752248, EPI_ISL_14752249			
see above	Department of Infectious Diseases, National Institute of Health Doutor Ricardo Jorge (INSA)	Department of Infectious Diseases, National Institute of Health Doutor Ricardo Jorge (INSA)	Isidro,J., Borges,V., Pinto,M., Sobral,D., Santos,J., Nunes,A., Mixao,V., Ferreira,R., Santos,D., Duarte,S., Vieira,L., Borrego,M.J., Nuncio,S., Lopes de Carvalho,I., Pelerito,A., Cordeiro,R. and Gomes,J.P.
EPI_ISL_14752284	Research and Evaluation, UKHSA	Research and Evaluation, UKHSA	Grove,N., Osman,K.L., Lewandowski,K.S., Carter,D.P., Pullan,S.T., Myers,R., Vipond,R. and Chand,M.
EPI_ISL_14752286, EPI_ISL_14752288, EPI_ISL_14752290, EPI_ISL_14752291	Research and Evaluation, UKHSA	Research and Evaluation, UKHSA	Groves,N., Osman,K.L., Lewandowski,K.S., Carter,D.P., Pullan,S.T., Myers,R., Vipond,R. and Chand,M.
EPI_ISL_14752293	Medical Microbiology & Infection Prevention, Amsterdam Medical Centres location AMC	Medical Microbiology & Infection Prevention, Amsterdam Medical Centres location AMC	Welkers,M., Jonges,M., de Regt,M., Ooijevaar,R. and Wagemakers,A.
EPI_ISL_14772317	Policlinica Jacare Wilson Federzoni Cabreuva	Instituto Adolfo Lutz Strategic Laboratory	Claudio Tavares Sacchi, Karoline Rodrigues Campos, Ariadne Ferreira Amarante, Marlon Benedito Nascimento Santos, Alex Domingos Reis, Adriano Abbud, Adriana Bugno
EPI_ISL_14772318	Secretaria Municipal de Saude de Sertaozinho	Instituto Adolfo Lutz Strategic Laboratory	Claudio Tavares Sacchi, Karoline Rodrigues Campos, Ariadne Ferreira Amarante, Marlon Benedito Nascimento Santos, Alex Domingos Reis, Adriano Abbud, Adriana Bugno
EPI_ISL_14772912	USF Jardim Oratorio	Instituto Adolfo Lutz Strategic Laboratory	Claudio Tavares Sacchi, Karoline Rodrigues Campos, Ariadne Ferreira Amarante, Marlon Benedito Nascimento Santos, Alex Domingos Reis, Adriano Abbud, Adriana Bugno
EPI_ISL_14772913	Vigilancia Epidemiologica Jardinopolis - SP	Instituto Adolfo Lutz Strategic Laboratory	Claudio Tavares Sacchi, Karoline Rodrigues Campos, Ariadne Ferreira Amarante, Marlon Benedito Nascimento Santos, Alex Domingos Reis, Adriano Abbud, Adriana Bugno
EPI_ISL_14772914	Pronto Atendimento Infantil e entral de Quimioterapia SJrpreto	Instituto Adolfo Lutz Strategic Laboratory	Claudio Tavares Sacchi, Karoline Rodrigues Campos, Ariadne Ferreira Amarante, Marlon Benedito Nascimento Santos, Alex Domingos Reis, Adriano Abbud, Adriana Bugno
EPI_ISL_14773001	CEDIC CTA	Instituto Adolfo Lutz Strategic Laboratory	Claudio Tavares Sacchi, Karoline Rodrigues Campos, Ariadne Ferreira Amarante, Marlon Benedito Nascimento Santos, Alex Domingos Reis, Adriano Abbud, Adriana Bugno
EPI_ISL_14783237	Sicilian Regional Laboratory - AOUP "P. Giaccone" - University of Palermo	Sicilian Regional Laboratory - AOUP "P. Giaccone" - University of Palermo	Fabio Tramuto, Carmelo Massimo Maida, Giulia Randazzo, Valeria Guzzetta, Walter Mazzucco, Giorgio Graziano, Vincenzo Restivo, Claudio Costantino, Francesco Vitale
EPI_ISL_14786290	IRCCS Sacro Cuore Don Calabria Hospital, Department of Infectious, Tropical Diseases & Microbiology	Department of Infectious, Tropical Diseases & Microbiology,IRCCS Sacro Cuore Don Calabria Hospital	Michela Deiana, Antonio Mori, Concetta Castilletti, Chiara Piubelli, Denise Lavezzari, Elena Pomari
EPI_ISL_14786346	IRCCS Sacro Cuore Don Calabria Hospital, Department of Infectious, Tropical Diseases & Microbiology	IRCCS Sacro Cuore Don Calabria Hospital, Department of Infectious, Tropical Diseases & Microbiology	Michela Deiana, Antonio Mori, Concetta Castilletti, Chiara Piubelli, Denise Lavezzari, Elena Pomari
EPI_ISL_14793992, EPI_ISL_14795058, EPI_ISL_14795084, EPI_ISL_14795085, EPI_ISL_14795259	Erasmus Medical Center Department of Virology	Erasmus Medical Center Department of Virology	Bas Oude Munnink, Leonard Schuele, Marjan Boter, Babette Weller, Babs Verstrepen, Richard Molenkamp, Janette Rahamat-Langendoen, Reina Sikkema, Marion Koopmans
EPI_ISL_14804638, EPI_ISL_14804639, EPI_ISL_14804640, EPI_ISL_14804641, EPI_ISL_14804642, EPI_ISL_14804643, EPI_ISL_14804644, EPI_ISL_14804645, EPI_ISL_14804646, EPI_ISL_14804647	Nebraska Public Health Laboratory	University of Nebraska Medical Center, Oklahoma Pathogen Genomics Consortium	Chapman,R.C., Bernhard,K., McCutchen,E.L., Fauver,J.R., O'Dell,J.X., Mannell,M., Wiley,M.R., Cross,S.T.
EPI_ISL_14809096	AMA Capao Redondo	Instituto Adolfo Lutz Strategic Laboratory	Claudio Tavares Sacchi, Karoline Rodrigues Campos, Ariadne Ferreira Amarante, Marlon Benedito Nascimento Santos, Alex Domingos Reis, Adriano Abbud, Adriana Bugno
EPI_ISL_14809097	Pronto Socorro Municipal de Taubate	Instituto Adolfo Lutz Strategic Laboratory	Claudio Tavares Sacchi, Karoline Rodrigues Campos, Ariadne Ferreira Amarante, Marlon Benedito Nascimento Santos, Alex Domingos Reis, Adriano Abbud, Adriana Bugno
EPI_ISL_14809098	Laboratorio Municipal de Piracicaba	Instituto Adolfo Lutz Strategic Laboratory	Claudio Tavares Sacchi, Karoline Rodrigues Campos, Ariadne Ferreira Amarante, Marlon Benedito Nascimento Santos, Alex Domingos Reis, Adriano Abbud, Adriana Bugno
EPI_ISL_14809099	Centro de Saude Gabriel de Lara	Instituto Adolfo Lutz Strategic Laboratory	Claudio Tavares Sacchi, Karoline Rodrigues Campos, Ariadne Ferreira Amarante, Marlon Benedito Nascimento Santos, Alex Domingos Reis, Adriano Abbud, Adriana Bugno
EPI_ISL_14809100	Secretaria Municipal da Saude de Joanopolis	Instituto Adolfo Lutz Strategic Laboratory	Claudio Tavares Sacchi, Karoline Rodrigues Campos, Ariadne Ferreira Amarante, Marlon Benedito Nascimento Santos, Alex Domingos Reis, Adriano Abbud, Adriana Bugno
EPI_ISL_14810370, EPI_ISL_14810404, EPI_ISL_14810405, EPI_ISL_14810406, EPI_ISL_14810407	Erasmus Medical Center Department of Virology	Erasmus Medical Center Department of Virology	Leonard Schuele, Bas Oude Munnink, Marjan Boter, Babette Weller, Babs Verstrepen, Richard Molenkamp, Janette Rahamat-Langendoen, Reina Sikkema, Marion Koopmans
EPI_ISL_14818585, EPI_ISL_14818587, EPI_ISL_14818589, EPI_ISL_14818590, EPI_ISL_14818595, EPI_ISL_14818600, EPI_ISL_14818602, EPI_ISL_14818603, EPI_ISL_14818611, EPI_ISL_14818615	Los Angeles County Public Health Laboratories	Los Angeles County Public Health Laboratories	P. Hemarajata et al.
EPI_ISL_14818783, EPI_ISL_14818784, EPI_ISL_14818785, EPI_ISL_14818786, EPI_ISL_14818787, EPI_ISL_14818788, EPI_ISL_14818789, EPI_ISL_14818790, EPI_ISL_14818791, EPI_ISL_14818792, EPI_ISL_14818793, EPI_ISL_14818794, EPI_ISL_14818795, EPI_ISL_14818796, EPI_ISL_14818797, EPI_ISL_14818798, EPI_ISL_14818799, EPI_ISL_14818800, EPI_ISL_14818801, EPI_ISL_14818802, EPI_ISL_14818803, EPI_ISL_14818804, EPI_ISL_14818805, EPI_ISL_14818806, EPI_ISL_14818807, EPI_ISL_14818808, EPI_ISL_14818809, EPI_ISL_14818810, EPI_ISL_14818811, EPI_ISL_14818812, EPI_ISL_14818813, EPI_ISL_14818814, EPI_ISL_14818815, EPI_ISL_14818817, EPI_ISL_14818818, EPI_ISL_14818819, EPI_ISL_14818820, EPI_ISL_14818821, EPI_ISL_14818822			
see above	Laboratorio de Referencia Nacional de Virus Inmunoprevenibles. Centro Nacional de Salud Publica. Instituto Nacional de Salud.	Laboratorio de Referencia Nacional de Virus Inmunoprevenibles. Centro Nacional de Salud Publica. Instituto Nacional de Salud.	Carlos Padilla Rojas, Veronica Hurtado Vela, Iris Silva Molina, Luren Sevilla Castañeda, Victor Jimenez Vasquez, Luis Barcena Flores, Alicia Nuñez Llanos, Kelly Izarra Rojas, Karla Vasquez Cajachahua, Estela Huaman Angeles, Jorge Giraldo Chavez, Lilian Huarca Balbin, Maria Sandra Villar Saavedra, Henri Bailon Calderon, Lely Solari Zerpa, Gloria Arotinco Garayar. Equipo de vigilancia genomica del Instituto Nacional de Salud.
EPI_ISL_14863040, EPI_ISL_14863041, EPI_ISL_14863042, EPI_ISL_14863044, EPI_ISL_14863046	Molecular Epidemiology, Idaho Bureau of Laboratories	Molecular Epidemiology, Idaho Bureau of Laboratories	Ceniseros,A.
EPI_ISL_14863048	MEPHI, IHU - Mediterranee Infection	MEPHI, IHU - Mediterranee Infection	Colson,P.
EPI_ISL_14863049	Molecular Epidemiology, Idaho Bureau of Laboratories	Molecular Epidemiology, Idaho Bureau of Laboratories	Ceniseros,A.
EPI_ISL_14863050, EPI_ISL_14863051, EPI_ISL_14863052, EPI_ISL_14863053, EPI_ISL_14863054, EPI_ISL_14863055, EPI_ISL_14863057, EPI_ISL_14863058, EPI_ISL_14863059, EPI_ISL_14863060, EPI_ISL_14863061, EPI_ISL_14863062, EPI_ISL_14863064, EPI_ISL_14863065			
see above	MEPHI, IHU - Mediterranee Infection	MEPHI, IHU - Mediterranee Infection	Colson,P.
EPI_ISL_14865785	UBS J COPA	Instituto Adolfo Lutz Strategic Laboratory	Claudio Tavares Sacchi, Karoline Rodrigues Campos, Ariadne Ferreira Amarante, Marlon Benedito Nascimento Santos, Alex Domingos Reis, Adriano Abbud, Adriana Bugno
EPI_ISL_14866481	PR S da Familia Unidade de Saude Adalberto Rocha	Instituto Adolfo Lutz Strategic Laboratory	Claudio Tavares Sacchi, Karoline Rodrigues Campos, Ariadne Ferreira Amarante, Marlon Benedito Nascimento Santos, Alex Domingos Reis, Adriano Abbud, Adriana Bugno
EPI_ISL_14866751	Pronto Socorro da Vila Dirce	Instituto Adolfo Lutz Strategic Laboratory	Claudio Tavares Sacchi, Karoline Rodrigues Campos, Ariadne Ferreira Amarante, Marlon Benedito Nascimento Santos, Alex Domingos Reis, Adriano Abbud, Adriana Bugno
EPI_ISL_14866752	Secretaria Municipal de Saude Sao Carlos	Instituto Adolfo Lutz Strategic Laboratory	Claudio Tavares Sacchi, Karoline Rodrigues Campos, Ariadne Ferreira Amarante, Marlon Benedito Nascimento Santos, Alex Domingos Reis, Adriano Abbud, Adriana Bugno
EPI_ISL_14887952, EPI_ISL_14887957, EPI_ISL_14887958, EPI_ISL_14887960, EPI_ISL_14887961, EPI_ISL_14887962, EPI_ISL_14887963, EPI_ISL_14887966, EPI_ISL_14887968, EPI_ISL_14887971, EPI_ISL_14887972			
see above	Viral Genotyping Reference Laboratory, Royal Infirmary of Edinburgh	Viral Genotyping Reference Laboratory, Royal Infirmary of Edinburgh	McHugh,M.P., Maloney,D., Parker,A., Mathers,K., Dewar,R., Kenicer,J., Cotton,S., Wild,J. and Templeton,K.E.
EPI_ISL_14910886	Research and Evaluation, UKHSA	Research and Evaluation, UKHSA	Burton,J., Easterbrook,L., Drinkwater,E., Groves,N., Osman,K.L., Lewandowski,K.S., Carter,D., Pullan,S.T., Myers,R., Vipond,R. and Chand,M.
EPI_ISL_14917565, EPI_ISL_14917569, EPI_ISL_14917571, EPI_ISL_14917576, EPI_ISL_14917580, EPI_ISL_14917581, EPI_ISL_14917587	Los Angeles County Public Health Laboratories	Los Angeles County Public Health Laboratories	P. Hemarajata et al.
EPI_ISL_14934116	Medical Center of Vienna Center for Virology	Medical University of Vienna Center for Virology	Jeremy V. Camp, Monika Redlberger-Fritz, Stephan W. Aberle
EPI_ISL_14934140	Center for Virology Medical Unviersity of Vienna	Medical University of Vienna Center for Virology	Jeremy V. Camp, Monika Redlberger-Fritz, Stephan W. Aberle
EPI_ISL_14934382	Medical University of Vienna Center for Virology	Medical University of Vienna Center for Virology	Jeremy V. Camp, Monika Redlberger-Fritz, Stephan W. Aberle
EPI_ISL_14934478	Medical University of Vienna Center for Virology	Medical University of Vienna Center for Virology	Jeremy V. Camp, Monika Redlberg-Fritz, Stephan W. Aberle
EPI_ISL_14934496, EPI_ISL_14934497, EPI_ISL_14934498, EPI_ISL_14934499, EPI_ISL_14934500, EPI_ISL_14934501, EPI_ISL_14934502, EPI_ISL_14934503, EPI_ISL_14934510, EPI_ISL_14934511, EPI_ISL_14934512, EPI_ISL_14934513, EPI_ISL_14934514, EPI_ISL_14934515, EPI_ISL_14934517, EPI_ISL_14934518, EPI_ISL_14934519, EPI_ISL_14934520, EPI_ISL_14934521, EPI_ISL_14934522, EPI_ISL_14934523, EPI_ISL_14934524, EPI_ISL_14934525, EPI_ISL_14934526, EPI_ISL_14934527, EPI_ISL_14934528, EPI_ISL_14934529, EPI_ISL_14934530, EPI_ISL_14934531, EPI_ISL_14934534, EPI_ISL_14934535, EPI_ISL_14934536, EPI_ISL_14934537, EPI_ISL_14934538, EPI_ISL_14934539, EPI_ISL_14934540, EPI_ISL_14934541, EPI_ISL_14934543, EPI_ISL_14934544, EPI_ISL_14934545, EPI_ISL_14934546, EPI_ISL_14934547, EPI_ISL_14934548, EPI_ISL_14934549, EPI_ISL_14934550, EPI_ISL_14934551, EPI_ISL_14934554, EPI_ISL_14934555, EPI_ISL_14934556, EPI_ISL_14934558, EPI_ISL_14934559, EPI_ISL_14934560, EPI_ISL_14934561, EPI_ISL_14934562, EPI_ISL_14934563, EPI_ISL_14934564, EPI_ISL_14934565, EPI_ISL_14934566, EPI_ISL_14934568, EPI_ISL_14934569, EPI_ISL_14934570, EPI_ISL_14934572, EPI_ISL_14934573, EPI_ISL_14934574, EPI_ISL_14934575, EPI_ISL_14934576, EPI_ISL_14934578, EPI_ISL_14934579, EPI_ISL_14934580, EPI_ISL_14934581, EPI_ISL_14934582, EPI_ISL_14934583, EPI_ISL_14934586, EPI_ISL_14934587, EPI_ISL_14934588, EPI_ISL_14934589, EPI_ISL_14934608, EPI_ISL_14934611, EPI_ISL_14934612, EPI_ISL_14934613, EPI_ISL_14934614, EPI_ISL_14934615, EPI_ISL_14934616, EPI_ISL_14934619			
see above	Department of Infectious Diseases, National Institute of Health Doutor Ricardo Jorge, Portugal (INSA)	Department of Infectious Diseases, National Institute of Health Doutor Ricardo Jorge, Portugal (INSA)	Isidro,J., Borges,V., Pinto,M., Sobral,D., Santos,J., Nunes,A., Mixao,V., Ferreira,R., Santos,D., Duarte,S., Vieira,L., Borrego,M.J., Nuncio,S., Lopes de Carvalho,I., Pelerito,A., Cordeiro,R. and Gomes,J.P.
EPI_ISL_14945299	Department of Microbiology, The University of Hong Kong	Department of Microbiology, The University of Hong Kong	Kelvin K.W. To, Kwok-Yung Yuen
EPI_ISL_14952916	Indian Council of Medical Research-National Institute of Virology	Indian Council of Medical Research-National Institute of Virology	Pragya Yadav, Rima Sahay, Anita Aich Shete, Sreelekshmy Mohandas, Priya Abraham
EPI_ISL_14961089, EPI_ISL_14961090	Public Health Authority of the Slovak Republic	Laboratory of Genomics and Bioinformatics, Comenius University Science Park	Tomáš Szemes, Edita Staroňová, Elena Tichá, Lucia Ševčíková, Terézia Vrabľová, Tatiana Sedláčková, Miroslav Böhmer, Jaroslav Budiš, Pavol Mišenko
EPI_ISL_14962734	Laboratory of Virology, University Hospitals of Geneva	Laboratory of Virology, University Hospitals of Geneva	Laubscher,F., Chudzinsk,V., Cordey,S., Schibler,M., Kaiser,L. and Renzoni,A.

EPI_ISL_14977306, EPI_ISL_14977307, EPI_ISL_14977308, EPI_ISL_14977309, EPI_ISL_14977310	Environmental, Agricultural, and Occupational Health, University of Nebraska Medical Center	Environmental, Agricultural, and Occupational Health, University of Nebraska Medical Center	Tegomoh,B., Cross,S.T., Chapman,R.C., Bernhard,K., McCutchen,E.L., Fauver,J.R., Pratt,C.B., Warden,D.E., Iwen,P.C., Donahue,M. and Wiley,M.R.
EPI_ISL_14994740	UBS Vila California Zeilival Bruscagin	Instituto Adolfo Lutz Strategic Laboratory	Claudio Tavares Sacchi, Karoline Rodrigues Campos, Ariadne Ferreira Amarante, Marlon Benedito Nascimento Santos, Alex Domingos Reis, Adriano Abbud, Adriana Bugno
EPI_ISL_14995206	Pronto Socorro Municiplal de Cravinhos	Instituto Adolfo Lutz Strategic Laboratory	Claudio Tavares Sacchi, Karoline Rodrigues Campos, Ariadne Ferreira Amarante, Marlon Benedito Nascimento Santos, Alex Domingos Reis, Adriano Abbud, Adriana Bugno
EPI_ISL_14995578	Hosp. Municipal de Ilhabela Gov. Mario Covas Jr.	Instituto Adolfo Lutz Strategic Laboratory	Claudio Tavares Sacchi, Karoline Rodrigues Campos, Ariadne Ferreira Amarante, Marlon Benedito Nascimento Santos, Alex Domingos Reis, Adriano Abbud, Adriana Bugno
EPI_ISL_14995579	Secretaria Municipal de Saude de Feira de Santana	Instituto Adolfo Lutz Strategic Laboratory	Claudio Tavares Sacchi, Karoline Rodrigues Campos, Ariadne Ferreira Amarante, Marlon Benedito Nascimento Santos, Alex Domingos Reis, Adriano Abbud, Adriana Bugno
EPI_ISL_14995580	UBS Alexander Fleming Simioni	Instituto Adolfo Lutz Strategic Laboratory	Claudio Tavares Sacchi, Karoline Rodrigues Campos, Ariadne Ferreira Amarante, Marlon Benedito Nascimento Santos, Alex Domingos Reis, Adriano Abbud, Adriana Bugno
EPI_ISL_14995581	Secretaria Municipal de Saude Sorocaba	Instituto Adolfo Lutz Strategic Laboratory	Claudio Tavares Sacchi, Karoline Rodrigues Campos, Ariadne Ferreira Amarante, Marlon Benedito Nascimento Santos, Alex Domingos Reis, Adriano Abbud, Adriana Bugno
EPI_ISL_14995582	Hosp. Municipla. Dr. Jose de Carvalho Florence	Instituto Adolfo Lutz Strategic Laboratory	Claudio Tavares Sacchi, Karoline Rodrigues Campos, Ariadne Ferreira Amarante, Marlon Benedito Nascimento Santos, Alex Domingos Reis, Adriano Abbud, Adriana Bugno
EPI_ISL_14995583	UBS Agua Rasa	Instituto Adolfo Lutz Strategic Laboratory	Claudio Tavares Sacchi, Karoline Rodrigues Campos, Ariadne Ferreira Amarante, Marlon Benedito Nascimento Santos, Alex Domingos Reis, Adriano Abbud, Adriana Bugno
EPI_ISL_14995585	Pronto Socorro Municipal do Promorar	Instituto Adolfo Lutz Strategic Laboratory	Claudio Tavares Sacchi, Karoline Rodrigues Campos, Ariadne Ferreira Amarante, Marlon Benedito Nascimento Santos, Alex Domingos Reis, Adriano Abbud, Adriana Bugno
EPI_ISL_14995586	UPA Centro	Instituto Adolfo Lutz Strategic Laboratory	Claudio Tavares Sacchi, Karoline Rodrigues Campos, Ariadne Ferreira Amarante, Marlon Benedito Nascimento Santos, Alex Domingos Reis, Adriano Abbud, Adriana Bugno
EPI_ISL_14995587, EPI_ISL_14995588	Centro de Saude 24 horas	Instituto Adolfo Lutz Strategic Laboratory	Claudio Tavares Sacchi, Karoline Rodrigues Campos, Ariadne Ferreira Amarante, Marlon Benedito Nascimento Santos, Alex Domingos Reis, Adriano Abbud, Adriana Bugno
EPI_ISL_14995589	Cresser Centro de Referencia da Saúde Sexual e Reprodutiva	Instituto Adolfo Lutz Strategic Laboratory	Claudio Tavares Sacchi, Karoline Rodrigues Campos, Ariadne Ferreira Amarante, Marlon Benedito Nascimento Santos, Alex Domingos Reis, Adriano Abbud, Adriana Bugno
EPI_ISL_14995590, EPI_ISL_14995591	Instituto de Infectologia Emilio Ribas	Instituto Adolfo Lutz Strategic Laboratory	Claudio Tavares Sacchi, Karoline Rodrigues Campos, Ariadne Ferreira Amarante, Marlon Benedito Nascimento Santos, Alex Domingos Reis, Adriano Abbud, Adriana Bugno
EPI_ISL_14995592	Unidade de Pronto Atendimento Cipo	Instituto Adolfo Lutz Strategic Laboratory	Claudio Tavares Sacchi, Karoline Rodrigues Campos, Ariadne Ferreira Amarante, Marlon Benedito Nascimento Santos, Alex Domingos Reis, Adriano Abbud, Adriana Bugno
EPI_ISL_14995593	SAE DST / Aids Ipiranga Jose Francisco Araujo	Instituto Adolfo Lutz Strategic Laboratory	Claudio Tavares Sacchi, Karoline Rodrigues Campos, Ariadne Ferreira Amarante, Marlon Benedito Nascimento Santos, Alex Domingos Reis, Adriano Abbud, Adriana Bugno
EPI_ISL_14995611	UBS Horto Florestal	Instituto Adolfo Lutz Strategic Laboratory	Claudio Tavares Sacchi, Karoline Rodrigues Campos, Ariadne Ferreira Amarante, Marlon Benedito Nascimento Santos, Alex Domingos Reis, Adriano Abbud, Adriana Bugno
EPI_ISL_14995612	Secretaria Minicipal de Saude de IRECE	Instituto Adolfo Lutz Strategic Laboratory	Claudio Tavares Sacchi, Karoline Rodrigues Campos, Ariadne Ferreira Amarante, Marlon Benedito Nascimento Santos, Alex Domingos Reis, Adriano Abbud, Adriana Bugno
EPI_ISL_14995619	Hosp. Tereza de Lisieux	Instituto Adolfo Lutz Strategic Laboratory	Claudio Tavares Sacchi, Karoline Rodrigues Campos, Ariadne Ferreira Amarante, Marlon Benedito Nascimento Santos, Alex Domingos Reis, Adriano Abbud, Adriana Bugno
EPI_ISL_14995622	UBS Parque Meia Lua	Instituto Adolfo Lutz Strategic Laboratory	Claudio Tavares Sacchi, Karoline Rodrigues Campos, Ariadne Ferreira Amarante, Marlon Benedito Nascimento Santos, Alex Domingos Reis, Adriano Abbud, Adriana Bugno
EPI_ISL_14995631	UPA Novo Horizonte	Instituto Adolfo Lutz Strategic Laboratory	Claudio Tavares Sacchi, Karoline Rodrigues Campos, Ariadne Ferreira Amarante, Marlon Benedito Nascimento Santos, Alex Domingos Reis, Adriano Abbud, Adriana Bugno
EPI_ISL_14995649	Instituto de Infectologia Emilio Ribas	Instituto Adolfo Lutz Strategic Laboratory	Claudio Tavares Sacchi, Karoline Rodrigues Campos, Ariadne Ferreira Amarante, Marlon Benedito Nascimento Santos, Alex Domingos Reis, Adriano Abbud, Adriana Bugno
EPI_ISL_14995652	Hosp. Dr. Osiris Florindo Coelho Ferraz de Vasconcelos	Instituto Adolfo Lutz Strategic Laboratory	Claudio Tavares Sacchi, Karoline Rodrigues Campos, Ariadne Ferreira Amarante, Marlon Benedito Nascimento Santos, Alex Domingos Reis, Adriano Abbud, Adriana Bugno
EPI_ISL_14995653	Unidade Basica de Saude Vila Cristina	Instituto Adolfo Lutz Strategic Laboratory	Claudio Tavares Sacchi, Karoline Rodrigues Campos, Ariadne Ferreira Amarante, Marlon Benedito Nascimento Santos, Alex Domingos Reis, Adriano Abbud, Adriana Bugno
EPI_ISL_14995723	Unidade Mista de Atendimento Infantil Carapicuiba	Instituto Adolfo Lutz Strategic Laboratory	Claudio Tavares Sacchi, Karoline Rodrigues Campos, Ariadne Ferreira Amarante, Marlon Benedito Nascimento Santos, Alex Domingos Reis, Adriano Abbud, Adriana Bugno
EPI_ISL_14995724	Hosp. Carlos Chagas	Instituto Adolfo Lutz Strategic Laboratory	Claudio Tavares Sacchi, Karoline Rodrigues Campos, Ariadne Ferreira Amarante, Marlon Benedito Nascimento Santos, Alex Domingos Reis, Adriano Abbud, Adriana Bugno
EPI_ISL_15005641	Chongqing Municipal Center for Disease Control and Prevention	Chongqing Municipal Center for Disease Control and Prevention	Sheng Ye, Yun Tang, Shuang Chen, Mingyue Wang, Zhangping Tan, Zhen Yu
EPI_ISL_15008574, EPI_ISL_15008575, EPI_ISL_15008577, EPI_ISL_15022589	Indian Council of Medical Research-National Institute of Virology	Indian Council of Medical Research-National Institute of Virology	Pragya Yadav, Rima Sahay, Anita Aich Shete, Sreelekshmy Mohandas, Priya Abraham
EPI_ISL_15055820	Sicilian Regional Laboratory - AOUP "P. Giaccone" - University of Palermo	Sicilian Regional Laboratory - AOUP "P. Giaccone" - University of Palermo	Fabio Tramuto, Carmelo Massimo Maida, Giulia Randazzo, Valeria Guzzetta, Walter Mazzucco, Giorgio Graziano, Vincenzo Restivo, Claudio Costantino, Francesco Vitale
EPI_ISL_15076130, EPI_ISL_15076131	Environmental, Agricultural, and Occupational Health, University of Nebraska Medical Center	Environmental, Agricultural, and Occupational Health, University of Nebraska Medical Center	Chapman,R.C., Bernhard,K., McCutchen,E.L., Fauver,J.R., O'Dell,J.X., Mannell,M., Wiley,M.R. and Cross,S.T.
EPI_ISL_15076180, EPI_ISL_15076181, EPI_ISL_15076182, EPI_ISL_15076183, EPI_ISL_15076184, EPI_ISL_15076185, EPI_ISL_15076186, EPI_ISL_15076187, EPI_ISL_15076188, EPI_ISL_15076189, EPI_ISL_15076191, EPI_ISL_15076192, EPI_ISL_15076193, EPI_ISL_15076194, EPI_ISL_15076195, EPI_ISL_15076196, EPI_ISL_15076197			
see above	Department of Genetics, University of North Carolina at Chapel Hill	Department of Genetics, University of North Carolina at Chapel Hill	Deanhardt,B., Miller,M. and Wang,J.R.
EPI_ISL_15104903	Institute for Virology, Philipps-University Marburg	Institute for Virology, Philipps-University Marburg	Eickmann, M., Lier, C., Kowalski, K., Kraft, F., Becker, S.
EPI_ISL_15120448, EPI_ISL_15120449, EPI_ISL_15120452, EPI_ISL_15120454, EPI_ISL_15120461, EPI_ISL_15120464, EPI_ISL_15120470, EPI_ISL_15120473, EPI_ISL_15120474, EPI_ISL_15120475, EPI_ISL_15120479, EPI_ISL_15120480, EPI_ISL_15120481, EPI_ISL_15120495, EPI_ISL_15120496			
see above	Los Angeles County Public Health Laboratories	Los Angeles County Public Health Laboratories	P. Hemarajata et al.
EPI_ISL_15158315, EPI_ISL_15158316, EPI_ISL_15158317, EPI_ISL_15158318, EPI_ISL_15158319, EPI_ISL_15158320, EPI_ISL_15158321, EPI_ISL_15158322, EPI_ISL_15158324, EPI_ISL_15158325, EPI_ISL_15158326, EPI_ISL_15158327, EPI_ISL_15158328, EPI_ISL_15158329, EPI_ISL_15158330, EPI_ISL_15158331, EPI_ISL_15158332, EPI_ISL_15158333, EPI_ISL_15158335, EPI_ISL_15158336, EPI_ISL_15158337, EPI_ISL_15158340, EPI_ISL_15158341, EPI_ISL_15158344, EPI_ISL_15158346, EPI_ISL_15158347, EPI_ISL_15158348, EPI_ISL_15158350, EPI_ISL_15158354, EPI_ISL_15158358, EPI_ISL_15158360, EPI_ISL_15158361, EPI_ISL_15158363, EPI_ISL_15158364, EPI_ISL_15158366, EPI_ISL_15158367, EPI_ISL_15158368, EPI_ISL_15158369, EPI_ISL_15158370, EPI_ISL_15158371, EPI_ISL_15158374, EPI_ISL_15158376, EPI_ISL_15158378, EPI_ISL_15158379, EPI_ISL_15158380, EPI_ISL_15158381, EPI_ISL_15158384, EPI_ISL_15158385, EPI_ISL_15158387, EPI_ISL_15158388, EPI_ISL_15158389, EPI_ISL_15158390, EPI_ISL_15158391, EPI_ISL_15158393, EPI_ISL_15158394, EPI_ISL_15158395, EPI_ISL_15158398			
see above	Molecular Biology, Microbiology, and Biochemistry, Southern Illinois University	Molecular Biology, Microbiology, and Biochemistry, Southern Illinois University	Gagnon,K.T.
EPI_ISL_15165602, EPI_ISL_15165603, EPI_ISL_15165604, EPI_ISL_15165605, EPI_ISL_15165606, EPI_ISL_15165607, EPI_ISL_15165608, EPI_ISL_15165610, EPI_ISL_15165611, EPI_ISL_15165612, EPI_ISL_15165614, EPI_ISL_15165615, EPI_ISL_15165616, EPI_ISL_15165617, EPI_ISL_15165618			
see above	Centro de Desenvolvimento Científico e Tecnológico (CDCT), Centro Estadual de Vigilância em Saúde (CEVS) da Secretaria Estadual da Saúde (SES-RS)	Centro de Desenvolvimento Científico e Tecnológico (CDCT), Centro Estadual de Vigilância em Saúde (CEVS) da Secretaria Estadual da Saúde (SES-RS)	Richard Steiner Salvato, Fernanda Marques Godinho, Regina Bones Barcellos, Patricia Sesterheim, Amanda Pellenz Ruivo, Viviane Horn de Melo, Júlio Augusto Schroder
EPI_ISL_15199627, EPI_ISL_15199628, EPI_ISL_15199630, EPI_ISL_15199632, EPI_ISL_15199636, EPI_ISL_15199638, EPI_ISL_15199639, EPI_ISL_15199645, EPI_ISL_15199646, EPI_ISL_15199649, EPI_ISL_15199652, EPI_ISL_15199653, EPI_ISL_15199656, EPI_ISL_15199659, EPI_ISL_15199661, EPI_ISL_15199662, EPI_ISL_15199665, EPI_ISL_15199668, EPI_ISL_15199669, EPI_ISL_15199670, EPI_ISL_15199671, EPI_ISL_15199672, EPI_ISL_15199673, EPI_ISL_15199676, EPI_ISL_15199677, EPI_ISL_15199679, EPI_ISL_15199680, EPI_ISL_15199681, EPI_ISL_15199682, EPI_ISL_15199685, EPI_ISL_15199687, EPI_ISL_15199688, EPI_ISL_15199690, EPI_ISL_15199691, EPI_ISL_15199693, EPI_ISL_15199696, EPI_ISL_15199697, EPI_ISL_15199698, EPI_ISL_15199699, EPI_ISL_15199700, EPI_ISL_15199702, EPI_ISL_15199703, EPI_ISL_15199705, EPI_ISL_15199706, EPI_ISL_15199717, EPI_ISL_15199721, EPI_ISL_15199724, EPI_ISL_15199725, EPI_ISL_15199726, EPI_ISL_15199727, EPI_ISL_15199728, EPI_ISL_15199729, EPI_ISL_15199730, EPI_ISL_15199731, EPI_ISL_15199732, EPI_ISL_15199733, EPI_ISL_15199735, EPI_ISL_15199736, EPI_ISL_15199737, EPI_ISL_15199738, EPI_ISL_15199739, EPI_ISL_15199740, EPI_ISL_15199742, EPI_ISL_15199743, EPI_ISL_15199744, EPI_ISL_15199747, EPI_ISL_15199749, EPI_ISL_15199750, EPI_ISL_15199751, EPI_ISL_15199752, EPI_ISL_15199753, EPI_ISL_15199754, EPI_ISL_15199757, EPI_ISL_15199761, EPI_ISL_15199762, EPI_ISL_15199764, EPI_ISL_15199765, EPI_ISL_15199767, EPI_ISL_15199768, EPI_ISL_15199769, EPI_ISL_15199773, EPI_ISL_15199775, EPI_ISL_15199776, EPI_ISL_15199777, EPI_ISL_15199778, EPI_ISL_15199780, EPI_ISL_15199781, EPI_ISL_15199787, EPI_ISL_15199788, EPI_ISL_15199789, EPI_ISL_15199790, EPI_ISL_15199791, EPI_ISL_15199793, EPI_ISL_15199794, EPI_ISL_15199795, EPI_ISL_15199796, EPI_ISL_15199797, EPI_ISL_15199798, EPI_ISL_15199799, EPI_ISL_15199830, EPI_ISL_15199856, EPI_ISL_15199861			
see above	Department of Infectious Diseases, National Institute of Health Doutor Ricardo Jorge, Portugal (INSA)	Department of Infectious Diseases, National Institute of Health Doutor Ricardo Jorge, Portugal (INSA)	Isidro,J., Borges,V., Pinto,M., Sobral,D., Santos,J., Nunes,A., Mixao,V., Ferreira,R., Santos,D., Duarte,S., Vieira,L., Borrego,M.J., Nuncio,S., Lopes de Carvalho,I., Pelerito,A., Cordeiro,R. and Gomes,J.P.
EPI_ISL_15226672, EPI_ISL_15226682, EPI_ISL_15226685, EPI_ISL_15226687, EPI_ISL_15226691, EPI_ISL_15226694, EPI_ISL_15226695	Los Angeles County Public Health Laboratories	Los Angeles County Public Health Laboratories	P. Hemarajata et al.
EPI_ISL_15247221, EPI_ISL_15247222, EPI_ISL_15247226, EPI_ISL_15247227	Medical University of Vienna Center for Virology	Medical University of Vienna Center for Virology	Jeremy V. Camp, Monika Redlberger-Fritz, Stephan W. Aberle
EPI_ISL_15257669	Public Health Agency of Canada, National Microbiology Laboratory	Public Health Agency of Canada, National Microbiology Laboratory	Duggan,A., Hole,D., Yadav,C., Knox,N., Haidl,E., Chapel,M., Tyler,A.D., Domselaar,G.V., Graham,M., Audet,J., Fernando,L., Hagan,M., Safronetz,D., Leung,A., Peters,G., Go,A., Kaplen,B., Antonation,K., Laminman,V., Jolly,G., Croxen,M., Deo,A., Dieu,P., Dong,X., Gill,K., Granger,D., Ferrato,C., Ikkurti,V., Kanji,J., Koleva,P., Li,V., Lloyd,C., Lynch,T., Ma,R., Pabbaraju,K., Rotich,S., Sergeant,H., Skitsko,T., Tipples,G., Thayer,J., Shideler,S. and Wong,A.
EPI_ISL_15257681, EPI_ISL_15257682, EPI_ISL_15257684, EPI_ISL_15257687	Viral and Rickettsial Disease Laboratory, California Department of Public Health	Viral and Rickettsial Disease Laboratory, California Department of Public Health	Probert,W., Espinosa,A., Kath,C., Haw,M., O'Neil,R., Bell,J. and Hacker,J.
EPI_ISL_15260057	High Containment Facility, Virology Division, Defence Research & Develeopment Establishment (DRDE)	Virology Division, Defence Research & Develeopment Establishment (DRDE)	Dr Sushil Kumar Sharma, Dr Paban Kumar Dash, Ram Govind Yadav, Ambuj Shrivastava, Rohit Menon, Dr Jyoti S Kumar, Dr Shashi Sharma, Dr Suman Dhankher, Divya Kumari, Dr Manmohan Parida
EPI_ISL_15263355	Sicilian Regional Laboratory - AOUP "P. Giaccone" - University of Palermo	Sicilian Regional Laboratory - AOUP "P. Giaccone" - University of Palermo	Fabio Tramuto, Carmelo Massimo Maida, Giulia Randazzo, Valeria Guzzetta, Walter Mazzucco, Giorgio Graziano, Vincenzo Restivo, Claudio Costantino, Francesco Vitale
EPI_ISL_15264003, EPI_ISL_15266514, EPI_ISL_15266518, EPI_ISL_15266615	Erasmus Medical Center Department of Virology	Erasmus Medical Center Department of Virology	Leonard Schuele, Bas Oude Munnink, Marjan Boter, Babette Weller, Babs Verstrepen, Richard Molenkamp, Janette Rahamat-Langendoen, Reina Sikkema, Marion Koopmans
EPI_ISL_15266843	Erasmus Medical Center Department of Virology	Erasmus Medical Center Department of Virology	Leonard Schuele, Bas Oude Munnink, Marjan Boter, David Nieuwenhuijse, Babette Weller, Babs Verstrepen, Richard Molenkamp, Janette Rahamat-Langendoen, Reina Sikkema, Marion Koopmans
EPI_ISL_15266988, EPI_ISL_15267015, EPI_ISL_15267029, EPI_ISL_15267031, EPI_ISL_15267800, EPI_ISL_15268281, EPI_ISL_15269049	Erasmus Medical Center Department of Virology	Erasmus Medical Center Department of Virology	Leonard Schuele, Bas Oude Munnink, Marjan Boter, Babette Weller, Babs Verstrepen, Richard Molenkamp, Janette Rahamat-Langendoen, Reina Sikkema, Marion Koopmans
EPI_ISL_15269237, EPI_ISL_15269238, EPI_ISL_15269375, EPI_ISL_15269384, EPI_ISL_15269386, EPI_ISL_15269387, EPI_ISL_15269388, EPI_ISL_15269544, EPI_ISL_15269595, EPI_ISL_15269598	Erasmus Medical Center Department of Virology	Erasmus Medical Center Department of Virology	Leonard Schuele, Bas Oude Munnink, Marjan Boter, David Nieuwenhuijse, Babette Weller, Babs Verstrepen, Richard Molenkamp, Janette Rahamat-Langendoen, Reina Sikkema, Marion Koopmans
EPI_ISL_15269698, EPI_ISL_15269699,	Erasmus Medical Center Department of Virology	Erasmus Medical Center Department of Virology	Leonard Schuele, Bas Oude Munnink, Marjan Boter, Babette Weller, Babs Verstrepen, Richard Molenkamp, Janette Rahamat-Langendoen, Reina Sikkema, Marion Koopmans

EPI_ISL_15269702, EPI_ISL_15269704, EPI_ISL_15269708			
EPI_ISL_15292947	Center of Scientific Excellence for Influenza Viruses, National Research Centre (NRC)	Center of Scientific Excellence for Influenza Viruses, National Research Centre (NRC)	Roshdy,W.H., El-Shesheny,R., Moatasim,Y.M., Kamel,M., Shawky,S., Gomaa,M., Naguib,A., El Guindy,N., Fahim,M., Khalifa,M., Galal,R., Hassany,M., Mohsen,A., Ali,M.A. and Kandeel,A.
EPI_ISL_15293815	National Institute for Viral Disease Control and Prevention (IVDC), Chinese Center for Disease Control and Prevention , Beijing, China	National Institute for Viral Disease Control and Prevention (IVDC), Chinese Center for Disease Control and Prevention , Beijing, China	Wenjie Tan, Changcheng Wu, Ruhan A, Wenling Wang, Roujian Lu, Li Zhao, Baoying Huang, Fei Ye, Wenbo Xu
EPI_ISL_15325417, EPI_ISL_15325422	Los Angeles County Public Health Laboratories	Los Angeles County Public Health Laboratories	P. Hemarajata et al.
EPI_ISL_15332336, EPI_ISL_15332337	Environmental, Agricultural, and Occupational Health, University of Nebraska Medical Center	Environmental, Agricultural, and Occupational Health, University of Nebraska Medical Center	Tegomoh,B., Cross,S.T., Chapman,R.C., Bernhard,K., McCutchen,E.L., Fauver,J.R., Pratt,C.B., Warden,D.E., Iwen,P.C., Donahue,M. and Wiley,M.R.
EPI_ISL_15332338, EPI_ISL_15332339, EPI_ISL_15332340	Environmental, Agricultural, and Occupational Health, University of Nebraska Medical Center	Environmental, Agricultural, and Occupational Health, University of Nebraska Medical Center	Chapman,R.C., Bernhard,K., McCutchen,E.L., Fauver,J.R., O'Dell,J.X., Mannell,M., Wiley,M.R. and Cross,S.T.
EPI_ISL_15370065, EPI_ISL_15370066, EPI_ISL_15370068, EPI_ISL_15370069, EPI_ISL_15370071, EPI_ISL_15370072, EPI_ISL_15370073, EPI_ISL_15370074, EPI_ISL_15370075, EPI_ISL_15370076, EPI_ISL_15370077, EPI_ISL_15370078, EPI_ISL_15370079, EPI_ISL_15370080, EPI_ISL_15370081, EPI_ISL_15370082			
see above	Nigeria Centre for Disease Control, National Reference Laboratory	Chemical, Biological and Radiological Sciences, Defence Science and Technology Laboratory	Ndodo,N., Ashcroft,J., Lewandowski,K., Yinka-Ogunleye,A., Chukwu,C., Ahmad,A., King,D., Akinpelu,A., Maluquer de Motes,C., Ribeca,P., Summer,R.P., Rambaut,A., Chester,M., Maishman,T., Babatunde,O., Mba,N., Babatunde,O., Aruna,O., Pullan,S.T., Gannon,B., Brown,C., Ihekweazu,C., Adetifa,I. and Ulaeto,D.O.
EPI_ISL_15373792	Department of immunology and microbiology - Pasteur Institute in Ho Chi Minh city	Department of immunology and microbiology - Pasteur Institute in Ho Chi Minh city	Manh H. Dao, Nhung H. P. Vu, Hang T. T. Pham, Thang M. Cao, Thinh V. Nguyen, Quang D. Pham, Quang C. Luong, Trung V. Nguyen
EPI_ISL_15380492	Eastwood Medical City	Molecular Biology Laboratory, Research Institute for Tropical Medicine	Samantha Louise P. Bado, Niquitta B. Galap, Bea C. Mateo, Chelsea Mae M. Reyes, Amalea Dulcene Nicolasora, Miguel Francisco B. Abulencia, Francisco Gerardo M. Polotan on behalf of the Research Institute for Tropical Medicine
EPI_ISL_15412318	CT Department of Public Health	CT Department of Public Health	Claire Pearson, Tu N. Nguyen, Kutluhan Incekara, Neranjan V. Perera
EPI_ISL_15417989, EPI_ISL_15417994, EPI_ISL_15417995, EPI_ISL_15417997, EPI_ISL_15418009, EPI_ISL_15418011	Los Angeles County Public Health Laboratories	Los Angeles County Public Health Laboratories	P. Hemarajata et al.
EPI_ISL_15419131	Instituto de Infectologia Emilio Ribas	Instituto Adolfo Lutz Strategic Laboratory	Claudio Tavares Sacchi, Karoline Rodrigues Campos, Ariadne Ferreira Amarante, Marlon Benedito Nascimento Santos, Adriano Abbud, Adriana Bugno
EPI_ISL_15419132	Centro de Saude de Sao Roque Dr Jose Carvalho Brito	Instituto Adolfo Lutz Strategic Laboratory	Claudio Tavares Sacchi, Karoline Rodrigues Campos, Ariadne Ferreira Amarante, Marlon Benedito Nascimento Santos, Adriano Abbud, Adriana Bugno
EPI_ISL_15419133	UBS II COHAB Presidente Prudente	Instituto Adolfo Lutz Strategic Laboratory	Claudio Tavares Sacchi, Karoline Rodrigues Campos, Ariadne Ferreira Amarante, Marlon Benedito Nascimento Santos, Adriano Abbud, Adriana Bugno
EPI_ISL_15419134	CTA Centro de Testagem e Aconselhamento de Caieiras	Instituto Adolfo Lutz Strategic Laboratory	Claudio Tavares Sacchi, Karoline Rodrigues Campos, Ariadne Ferreira Amarante, Marlon Benedito Nascimento Santos, Adriano Abbud, Adriana Bugno
EPI_ISL_15419135	SAE DST AIDS Cidade Dutra	Instituto Adolfo Lutz Strategic Laboratory	Claudio Tavares Sacchi, Karoline Rodrigues Campos, Ariadne Ferreira Amarante, Marlon Benedito Nascimento Santos, Adriano Abbud, Adriana Bugno
EPI_ISL_15419136	UBS J Nordeste	Instituto Adolfo Lutz Strategic Laboratory	Claudio Tavares Sacchi, Karoline Rodrigues Campos, Ariadne Ferreira Amarante, Marlon Benedito Nascimento Santos, Adriano Abbud, Adriana Bugno
EPI_ISL_15419137	CTA Centro de Testagem e Aconselhamento Favo de Mel	Instituto Adolfo Lutz Strategic Laboratory	Claudio Tavares Sacchi, Karoline Rodrigues Campos, Ariadne Ferreira Amarante, Marlon Benedito Nascimento Santos, Adriano Abbud, Adriana Bugno
EPI_ISL_15419138	Vigilancia Epidemiologica e Controle de Vetores de Pirassununga	Instituto Adolfo Lutz Strategic Laboratory	Claudio Tavares Sacchi, Karoline Rodrigues Campos, Ariadne Ferreira Amarante, Marlon Benedito Nascimento Santos, Adriano Abbud, Adriana Bugno
EPI_ISL_15419139	Policlinica Maria Dirce	Instituto Adolfo Lutz Strategic Laboratory	Claudio Tavares Sacchi, Karoline Rodrigues Campos, Ariadne Ferreira Amarante, Marlon Benedito Nascimento Santos, Adriano Abbud, Adriana Bugno
EPI_ISL_15419140	Secretaria Municipal de Saude de Batatais SP	Instituto Adolfo Lutz Strategic Laboratory	Claudio Tavares Sacchi, Karoline Rodrigues Campos, Ariadne Ferreira Amarante, Marlon Benedito Nascimento Santos, Adriano Abbud, Adriana Bugno
EPI_ISL_15419141	UBS J Nordeste	Instituto Adolfo Lutz Strategic Laboratory	Claudio Tavares Sacchi, Karoline Rodrigues Campos, Ariadne Ferreira Amarante, Marlon Benedito Nascimento Santos, Adriano Abbud, Adriana Bugno
EPI_ISL_15419142	Instituto de Infectologia Emilio Ribas	Instituto Adolfo Lutz Strategic Laboratory	Claudio Tavares Sacchi, Karoline Rodrigues Campos, Ariadne Ferreira Amarante, Marlon Benedito Nascimento Santos, Adriano Abbud, Adriana Bugno
EPI_ISL_15419143	Santa Casa de Barretos	Instituto Adolfo Lutz Strategic Laboratory	Claudio Tavares Sacchi, Karoline Rodrigues Campos, Ariadne Ferreira Amarante, Marlon Benedito Nascimento Santos, Adriano Abbud, Adriana Bugno
EPI_ISL_15419144	Hospital Vera Cruz	Instituto Adolfo Lutz Strategic Laboratory	Claudio Tavares Sacchi, Karoline Rodrigues Campos, Ariadne Ferreira Amarante, Marlon Benedito Nascimento Santos, Adriano Abbud, Adriana Bugno
EPI_ISL_15419145	NotreDame Intermedica Saude	Instituto Adolfo Lutz Strategic Laboratory	Claudio Tavares Sacchi, Karoline Rodrigues Campos, Ariadne Ferreira Amarante, Marlon Benedito Nascimento Santos, Adriano Abbud, Adriana Bugno
EPI_ISL_15419146	Hospital e Maternidade Santa Maria Cruz Azul	Instituto Adolfo Lutz Strategic Laboratory	Claudio Tavares Sacchi, Karoline Rodrigues Campos, Ariadne Ferreira Amarante, Marlon Benedito Nascimento Santos, Adriano Abbud, Adriana Bugno
EPI_ISL_15419147	Pronto Socorro Central de Diadema	Instituto Adolfo Lutz Strategic Laboratory	Claudio Tavares Sacchi, Karoline Rodrigues Campos, Ariadne Ferreira Amarante, Marlon Benedito Nascimento Santos, Adriano Abbud, Adriana Bugno
EPI_ISL_15419148	NotreDame Intermedica Saude Santo Andre	Instituto Adolfo Lutz Strategic Laboratory	Claudio Tavares Sacchi, Karoline Rodrigues Campos, Ariadne Ferreira Amarante, Marlon Benedito Nascimento Santos, Adriano Abbud, Adriana Bugno
EPI_ISL_15419149	UBS Dom Angelico	Instituto Adolfo Lutz Strategic Laboratory	Claudio Tavares Sacchi, Karoline Rodrigues Campos, Ariadne Ferreira Amarante, Marlon Benedito Nascimento Santos, Adriano Abbud, Adriana Bugno
EPI_ISL_15419150	Secao de Centro de Diagnostico SECEDI	Instituto Adolfo Lutz Strategic Laboratory	Claudio Tavares Sacchi, Karoline Rodrigues Campos, Ariadne Ferreira Amarante, Marlon Benedito Nascimento Santos, Adriano Abbud, Adriana Bugno
EPI_ISL_15419151	UPA 24H Brotas	Instituto Adolfo Lutz Strategic Laboratory	Claudio Tavares Sacchi, Karoline Rodrigues Campos, Ariadne Ferreira Amarante, Marlon Benedito Nascimento Santos, Adriano Abbud, Adriana Bugno
EPI_ISL_15419152	Hospital Nossa Senhora de Lourdes	Instituto Adolfo Lutz Strategic Laboratory	Claudio Tavares Sacchi, Karoline Rodrigues Campos, Ariadne Ferreira Amarante, Marlon Benedito Nascimento Santos, Adriano Abbud, Adriana Bugno
EPI_ISL_15419153	AMA Paraisopolis	Instituto Adolfo Lutz Strategic Laboratory	Claudio Tavares Sacchi, Karoline Rodrigues Campos, Ariadne Ferreira Amarante, Marlon Benedito Nascimento Santos, Adriano Abbud, Adriana Bugno
EPI_ISL_15419154	Pronto Atendimento Infantil e Central de Quimioterapia de Sao Jose do Rio Preto	Instituto Adolfo Lutz Strategic Laboratory	Claudio Tavares Sacchi, Karoline Rodrigues Campos, Ariadne Ferreira Amarante, Marlon Benedito Nascimento Santos, Adriano Abbud, Adriana Bugno
EPI_ISL_15419155	Santa Casa de Atibaia Pro Saude	Instituto Adolfo Lutz Strategic Laboratory	Claudio Tavares Sacchi, Karoline Rodrigues Campos, Ariadne Ferreira Amarante, Marlon Benedito Nascimento Santos, Adriano Abbud, Adriana Bugno
EPI_ISL_15419156	UPA Vila Mariana	Instituto Adolfo Lutz Strategic Laboratory	Claudio Tavares Sacchi, Karoline Rodrigues Campos, Ariadne Ferreira Amarante, Marlon Benedito Nascimento Santos, Adriano Abbud, Adriana Bugno
EPI_ISL_15419157	Secretaria de Saude de Mogi das Cruzes	Instituto Adolfo Lutz Strategic Laboratory	Claudio Tavares Sacchi, Karoline Rodrigues Campos, Ariadne Ferreira Amarante, Marlon Benedito Nascimento Santos, Adriano Abbud, Adriana Bugno
EPI_ISL_15419158	Unidade Mista de saude Mariano Gayoso Castelo Branco	Instituto Adolfo Lutz Strategic Laboratory	Claudio Tavares Sacchi, Karoline Rodrigues Campos, Ariadne Ferreira Amarante, Marlon Benedito Nascimento Santos, Adriano Abbud, Adriana Bugno
EPI_ISL_15419159	Hospital Vivalle	Instituto Adolfo Lutz Strategic Laboratory	Claudio Tavares Sacchi, Karoline Rodrigues Campos, Ariadne Ferreira Amarante, Marlon Benedito Nascimento Santos, Adriano Abbud, Adriana Bugno
EPI_ISL_15419160	UPA Nova Hortolandia Manoel Geogino Lopes	Instituto Adolfo Lutz Strategic Laboratory	Claudio Tavares Sacchi, Karoline Rodrigues Campos, Ariadne Ferreira Amarante, Marlon Benedito Nascimento Santos, Adriano Abbud, Adriana Bugno
EPI_ISL_15419161	Centro de Saude I Albertino Affonso Jaboticabal	Instituto Adolfo Lutz Strategic Laboratory	Claudio Tavares Sacchi, Karoline Rodrigues Campos, Ariadne Ferreira Amarante, Marlon Benedito Nascimento Santos, Adriano Abbud, Adriana Bugno
EPI_ISL_15419162	SAE DST AIDS M Boi Mirim Servico de Atencao Especializada	Instituto Adolfo Lutz Strategic Laboratory	Claudio Tavares Sacchi, Karoline Rodrigues Campos, Ariadne Ferreira Amarante, Marlon Benedito Nascimento Santos, Adriano Abbud, Adriana Bugno
EPI_ISL_15419163	Instituto de Infectologia Emilio Ribas	Instituto Adolfo Lutz Strategic Laboratory	Claudio Tavares Sacchi, Karoline Rodrigues Campos, Ariadne Ferreira Amarante, Marlon Benedito Nascimento Santos, Adriano Abbud, Adriana Bugno
EPI_ISL_15455916, EPI_ISL_15455917	Direccion de Investigacion en Salud Publica, Instituto Nacional de Salud	Direccion de Investigacion en Salud Publica, Instituto Nacional de Salud	Laiton-Donato,K., Alvarez-Diaz,D.A., Franco-Munoz,C., Ruiz-Moreno,H.A., Rojas-Estevez,P., Rosales,A., Martinez,D., Flores,A., Prieto,F., Walteros,D., Gomez,S. and Mercado-Reyes,M.
EPI_ISL_15458903, EPI_ISL_15458904, EPI_ISL_15458905, EPI_ISL_15458906	Nebraska Public Health Laboratory (NPHL)	Environmental, Agricultural, and Occupational Health, University of Nebraska Medical Center	Chapman,R.C., Bernhard,K., McCutchen,E.L., Fauver,J.R., O'Dell,J.X., Mannell,M., Wiley,M.R. and Cross,S.T.
EPI_ISL_15502326, EPI_ISL_15502328	Los Angeles County Public Health Laboratories	Los Angeles County Public Health Laboratories	P. Hemarajata et al.
EPI_ISL_15528148	Department of Clinical Sciences, Institute of Tropica Medicine	Department of Clinical Sciences, Institute of Tropica Medicine	Berens-Riha,N., De Block,T., Rutgers,J., Van Gestel,L., Hens,M., Kenyon,C., Soentjens,P., Van Griensven,J., Brosius,I., Arien,K., Van Esbroeck,M., Rezende,A.M., Vercauteren,K. and Liesenborghs,L.
EPI_ISL_15528149, EPI_ISL_15528150, EPI_ISL_15528151	Department of Clinical Sciences, Institute of Tropica Medicine	Department of Clinical Sciences, Institute of Tropica Medicine	Berens-Riha,N., De Block,T., Rutgers,J., Van Gestel,L., Hens,M., Kenyon,C., Soentjens,P., Van Griensven,J., Brosius,I., Arien,K., Van Esbroeck,M., Rezende,A.M. and Vercauteren,K.
EPI_ISL_15593717	LESP State of Mexico	Instituto de Diagnostico y Referencia Epidemiologicos (INDRE)	Abril Rodríguez-Maldonado; Claudia Wong-Arámbula; Felipe Arguijo-Perez; Helios Cárdenas-Hernández; Carmen Castro-Méndez; Lidia García-Torres; Ruth Madera-Sandoval; América Mandujano-Martínez; Nancy Martínez-Velázquez; Mireya Mederos-Michel; Angélica Pedraza-Meléndez; Joaquín Quiroz-Mercado; Daniel Regalado-Santiago; Silvia Rivero-Arredondo; Erika Sierra-Atanacio; Fernando González-Domínguez; Lucia Hernández-Rivas, Irma López-Martínez; Ernesto Ramírez-González; Maribel González-Villa
EPI_ISL_15593718	LESP Mexico City	Instituto de Diagnostico y Referencia Epidemiologicos (INDRE)	Abril Rodríguez-Maldonado; Claudia Wong-Arámbula; Felipe Arguijo-Perez; Helios Cárdenas-Hernández; Carmen Castro-Méndez; Lidia García-Torres; Ruth Madera-Sandoval; América Mandujano-Martínez; Nancy Martínez-Velázquez; Mireya Mederos-Michel; Angélica Pedraza-Meléndez; Joaquín Quiroz-Mercado; Daniel Regalado-Santiago; Silvia Rivero-Arredondo; Erika Sierra-Atanacio; Fernando González-Domínguez; Lucia Hernández-Rivas, Irma López-Martínez; Ernesto Ramírez-González; Maribel González-Villa
EPI_ISL_15593719	LESP Puebla	Instituto de Diagnostico y Referencia Epidemiologicos (INDRE)	Abril Rodríguez-Maldonado; Claudia Wong-Arámbula; Felipe Arguijo-Perez; Helios Cárdenas-Hernández; Carmen Castro-Méndez; Lidia García-Torres; Ruth Madera-Sandoval; América Mandujano-Martínez; Nancy Martínez-Velázquez; Mireya Mederos-Michel; Angélica Pedraza-Meléndez; Joaquín Quiroz-Mercado; Daniel Regalado-Santiago; Silvia Rivero-Arredondo; Erika Sierra-Atanacio; Fernando González-Domínguez; Lucia Hernández-Rivas, Irma López-Martínez; Ernesto Ramírez-González; Maribel González-Villa
EPI_ISL_15593720	LESP Tamaulipas	Instituto de Diagnostico y Referencia Epidemiologicos (INDRE)	Abril Rodríguez-Maldonado; Claudia Wong-Arámbula; Felipe Arguijo-Perez; Helios Cárdenas-Hernández; Carmen Castro-Méndez; Lidia García-Torres; Ruth Madera-Sandoval; América Mandujano-Martínez; Nancy Martínez-Velázquez; Mireya Mederos-Michel; Angélica Pedraza-Meléndez; Joaquín Quiroz-Mercado; Daniel Regalado-Santiago; Silvia Rivero-Arredondo; Erika Sierra-Atanacio; Fernando González-Domínguez; Lucia Hernández-Rivas, Irma López-Martínez; Ernesto Ramírez-González; Maribel González-Villa
EPI_ISL_15593721	LESP Baja California	Instituto de Diagnostico y Referencia Epidemiologicos (INDRE)	Abril Rodríguez-Maldonado; Claudia Wong-Arámbula; Felipe Arguijo-Perez; Helios Cárdenas-Hernández; Carmen Castro-Méndez; Lidia García-Torres; Ruth Madera-Sandoval; América Mandujano-Martínez; Nancy Martínez-Velázquez; Mireya Mederos-Michel; Angélica Pedraza-Meléndez; Joaquín Quiroz-Mercado; Daniel Regalado-Santiago; Silvia Rivero-Arredondo; Erika Sierra-Atanacio; Fernando González-Domínguez; Lucia Hernández-Rivas, Irma López-Martínez;

			Ernesto Ramírez-González; Maribel González-Villa
EPI_ISL_15593722	LESP Nuevo Leon	Instituto de Diagnostico y Referencia Epidemiologicos (INDRE)	Abril Rodríguez-Maldonado; Claudia Wong-Arámbula; Felipe Arguijo-Perez; Helios Cárdenas-Hernández; Carmen Castro-Méndez; Lidia García-Torres; Ruth Madera-Sandoval; América Mandujano-Martínez; Nancy Martínez-Velázquez; Mireya Mederos-Michel; Angélica Pedraza-Meléndez; Joaquín Quiroz-Mercado; Daniel Regalado-Santiago; Silvia Rivero-Arredondo; Erika Sierra-Atanacio; Fernando González-Domínguez; Lucia Hernández-Rivas, Irma López-Martínez; Ernesto Ramírez-González; Maribel González-Villa
EPI_ISL_15597040, EPI_ISL_15597041, EPI_ISL_15597042, EPI_ISL_15597043, EPI_ISL_15597044, EPI_ISL_15597045	UCLA Clinical Micro Lab	Los Angeles County Public Health Laboratories	P. Hemarajata et al.
EPI_ISL_15597047, EPI_ISL_15597048	Quest Diagnostics Nichols Institute	Los Angeles County Public Health Laboratories	P. Hemarajata et al.
EPI_ISL_15597059, EPI_ISL_15597062	Los Angeles County Public Health Laboratories	Los Angeles County Public Health Laboratories	P. Hemarajata et al.
EPI_ISL_15608908, EPI_ISL_15608909, EPI_ISL_15608910	Southern Nevada Public Health Laboratory	Southern Nevada Public Health Laboratory	Michael Picker
EPI_ISL_15641541, EPI_ISL_15641542, EPI_ISL_15641543, EPI_ISL_15641544, EPI_ISL_15641545, EPI_ISL_15641546, EPI_ISL_15641547, EPI_ISL_15641548, EPI_ISL_15641549, EPI_ISL_15641550, EPI_ISL_15641551, EPI_ISL_15641552, EPI_ISL_15641553, EPI_ISL_15641554, EPI_ISL_15641555, EPI_ISL_15641556, EPI_ISL_15641557, EPI_ISL_15641559, EPI_ISL_15641560, EPI_ISL_15641561, EPI_ISL_15641562, EPI_ISL_15641563, EPI_ISL_15641564, EPI_ISL_15641565, EPI_ISL_15641566, EPI_ISL_15641567, EPI_ISL_15641568, EPI_ISL_15641569, EPI_ISL_15641570, EPI_ISL_15641571, EPI_ISL_15641572, EPI_ISL_15641573, EPI_ISL_15641574, EPI_ISL_15641576, EPI_ISL_15641577, EPI_ISL_15641578, EPI_ISL_15641579, EPI_ISL_15641580, EPI_ISL_15641581, EPI_ISL_15641582, EPI_ISL_15641583, EPI_ISL_15641584, EPI_ISL_15641585, EPI_ISL_15641586, EPI_ISL_15641587, EPI_ISL_15641588, EPI_ISL_15641589, EPI_ISL_15641590, EPI_ISL_15641591, EPI_ISL_15641592, EPI_ISL_15641593, EPI_ISL_15641594, EPI_ISL_15641595, EPI_ISL_15641596, EPI_ISL_15641597, EPI_ISL_15641598, EPI_ISL_15641599, EPI_ISL_15641600, EPI_ISL_15641601, EPI_ISL_15641602, EPI_ISL_15641603, EPI_ISL_15641604, EPI_ISL_15641605, EPI_ISL_15641606, EPI_ISL_15641607, EPI_ISL_15641608, EPI_ISL_15641609			
see above	Public Health Laboratory, Public Health Service Amsterdam, The Netherlands	Department of Medical Microbiology & Infection prevention, Amsterdam University Medical Centers location AMC	Matthijs Welkers, Jelle Koopsen, Robin van Houdt, Marcel Jonges, Sebastien Matamoros, Sjoerd Rebers, Fokla Zorgdrager, Sylvia Bruisten, Akke Cornelissen, Janke Schinkel, Ewout Fanoy, Roisin Bavalia, Menno de Jong and Mariken van der Lubben on behalf of the Amsterdam Regional Genomic epidemiology and Outbreak Surveillance (ARGOS) consortium
EPI_ISL_15655944	Erasmus Medical Center Department of Virology	Erasmus Medical Center Department of Virology	Leonard Schuele, Bas Oude Munnink, Marjan Boter, Babette Weller, Babs Verstrepen, Richard Molenkamp, Janette Rahamat-Langendoen, Reina Sikkema, Marion Koopmans
EPI_ISL_15684645	Laboratory of Virology, University Hospitals of Geneva	Laboratory of Virology, University Hospitals of Geneva	Laubscher,F., Marques-Melancia,S., Cordey,S., Schibler,M.,Kaiser,L. and Renzoni,A.
EPI_ISL_15702015, EPI_ISL_15702711, EPI_ISL_15704845, EPI_ISL_15712853, EPI_ISL_15712860, EPI_ISL_15714234, EPI_ISL_15714244, EPI_ISL_15714245, EPI_ISL_15714286, EPI_ISL_15714287, EPI_ISL_15714288, EPI_ISL_15714299, EPI_ISL_15714539, EPI_ISL_15714920, EPI_ISL_15714989, EPI_ISL_15714990, EPI_ISL_15715289, EPI_ISL_15715292, EPI_ISL_15715305, EPI_ISL_15718514, EPI_ISL_15718920			
see above	Erasmus Medical Center Department of Virology	Erasmus Medical Center Department of Virology	Leonard Schuele, Bas Oude Munnink, Marjan Boter, Babette Weller, Babs Verstrepen, Richard Molenkamp, Janette Rahamat-Langendoen, Reina Sikkema, Marion Koopmans
EPI_ISL_15722680, EPI_ISL_15722681, EPI_ISL_15722682, EPI_ISL_15722683, EPI_ISL_15722684, EPI_ISL_15722685, EPI_ISL_15722686, EPI_ISL_15722687, EPI_ISL_15722688, EPI_ISL_15722689, EPI_ISL_15722690			
see above	Rush University Medical Center	RIPHL at Rush University Medical Center	Stefan Green, Kevin Kunstman, Hannah Barbian, Felix Araujo Perez, Edith Perez, Sofiya Bobrovska, Alyse Kittner, Cecilia Chau, Giancarlo Balangue, Lok Yiu Ashley Wu, Mary Hayden, Joyce Houlihan, Diane Springer, Nicholas Moore
EPI_ISL_15747900	WHO National Influenza Centre Russian Federation	WHO National Influenza Centre Russian Federation	Andrey Komissarov, Artem Fadeev, Nikita Yolshin, Evgeny Venev, Kseniya Komissarova, Daria Danilenko, Dmitry Lioznov
EPI_ISL_15763810, EPI_ISL_15763811, EPI_ISL_15763812, EPI_ISL_15763813, EPI_ISL_15763814, EPI_ISL_15763815, EPI_ISL_15763816, EPI_ISL_15763817, EPI_ISL_15763818, EPI_ISL_15763819, EPI_ISL_15763820, EPI_ISL_15763821, EPI_ISL_15763822, EPI_ISL_15763823, EPI_ISL_15763824, EPI_ISL_15763825, EPI_ISL_15763826, EPI_ISL_15763827, EPI_ISL_15763828, EPI_ISL_15763829, EPI_ISL_15763830, EPI_ISL_15763831, EPI_ISL_15763832			
see above	National Virus Reference Laboratory	National Virus Reference Laboratory	Gabriel Gonzalez, Michael Carr, Brian Keogan, Jose Maria Urtasun Elizari, Jonathan Dean, Daniel Hare, Cillian F De Gascun
EPI_ISL_15802695, EPI_ISL_15802697, EPI_ISL_15802698, EPI_ISL_15802700, EPI_ISL_15802703, EPI_ISL_15802704, EPI_ISL_15802705, EPI_ISL_15802706, EPI_ISL_15802707	Direccion de Investigacion en Salud Publica, Instituto Nacional de Salud	Direccion de Investigacion en Salud Publica, Instituto Nacional de Salud	Laiton-Donato,K.D., Franco,C.E., Alvarez-Diaz,D.A., Ruiz-Moreno,H.A., Prada,D.A., Martinez,D. and Mercado-Reyes,M.M.
EPI_ISL_15802709	Animal Health, Istituto Zooprofilattico Sperimentale del Mezzogiorno	Animal Health, Istituto Zooprofilattico Sperimentale del Mezzogiorno	Viscardi,M., Cozzolino,L., De Martinis,C., Cardillo,L. and Fusco,G.
EPI_ISL_15802710, EPI_ISL_15802713, EPI_ISL_15802718, EPI_ISL_15802721	Direccion de Investigacion en Salud Publica, Instituto Nacional de Salud	Direccion de Investigacion en Salud Publica, Instituto Nacional de Salud	Laiton-Donato,K.D., Franco,C.E., Alvarez-Diaz,D.A., Ruiz-Moreno,H.A., Prada,D.A., Martinez,D. and Mercado-Reyes,M.M.
EPI_ISL_15802722, EPI_ISL_15802723, EPI_ISL_15802724, EPI_ISL_15802725, EPI_ISL_15802726, EPI_ISL_15802727, EPI_ISL_15802728, EPI_ISL_15802729, EPI_ISL_15802730, EPI_ISL_15802731, EPI_ISL_15802732, EPI_ISL_15802733, EPI_ISL_15802734, EPI_ISL_15802735, EPI_ISL_15802736, EPI_ISL_15802737, EPI_ISL_15802738, EPI_ISL_15802739, EPI_ISL_15802740, EPI_ISL_15802741, EPI_ISL_15802742, EPI_ISL_15802743			
see above	Centre for Biological Threats, Highly Pathogenic Viruses, Robert Koch Institute	Centre for Biological Threats, Highly Pathogenic Viruses, Robert Koch Institute	Brinkmann,A., Kohl,C., Pape,K., Schrick,L., Michel,J., Schaade,L. and Nitsche,A.
EPI_ISL_15819629	California Department of Public Health	California Department of Public Health	Viral and Rickettsial Disease Laboratory
EPI_ISL_15831211	División Diagnóstico Molecular Hospital México	División Diagnóstico Molecular Hospital México	Juan Carlos Villalobos Ugalde, Vanessa Villalobos Alfaro, Carlos Ramirez Chavarría
EPI_ISL_15831212	División Diagnóstico Molecular Hospital México	División Diagnóstico Molecular Hospital México	Juan Carlos Villalobos Ugalde, Vanessa Villalobos Alfaro, Sofía Villalobos Abarca
EPI_ISL_15889147	Public Health and Environmental Laboratories, New Jersey Department of Health	Public Health and Environmental Laboratories, New Jersey Department of Health	Palmateer,N.C.
EPI_ISL_15896303, EPI_ISL_15896305	Los Angeles County Public Health Laboratories	Los Angeles County Public Health Laboratories	P. Hemarajata et al.
EPI_ISL_15896313, EPI_ISL_15896315, EPI_ISL_15896317, EPI_ISL_15896322, EPI_ISL_15896324, EPI_ISL_15896326	Kaiser Permanente Chino Hills Regional Reference Laboratories	Los Angeles County Public Health Laboratories	P. Hemarajata et al.
EPI_ISL_15896329, EPI_ISL_15896341, EPI_ISL_15896342, EPI_ISL_15896345, EPI_ISL_15896350, EPI_ISL_15896351	Los Angeles County Public Health Laboratories	Los Angeles County Public Health Laboratories	P. Hemarajata et al.
EPI_ISL_15912317, EPI_ISL_15912318, EPI_ISL_15912319, EPI_ISL_15912320, EPI_ISL_15912321, EPI_ISL_15912322, EPI_ISL_15912323, EPI_ISL_15912324, EPI_ISL_15912325, EPI_ISL_15912326, EPI_ISL_15912327, EPI_ISL_15912328, EPI_ISL_15912329, EPI_ISL_15912330, EPI_ISL_15912331, EPI_ISL_15912332, EPI_ISL_15912333, EPI_ISL_15912334, EPI_ISL_15912335, EPI_ISL_15912336, EPI_ISL_15912337, EPI_ISL_15912338, EPI_ISL_15912339, EPI_ISL_15912340, EPI_ISL_15912341, EPI_ISL_15912342, EPI_ISL_15912343, EPI_ISL_15912344			
see above	Indian Council of Medical Research-National Institute of Virology, Microbial Containment Complex	Indian Council of Medical Research-National Institute of Virology, Microbial Containment Complex	Pragya D. Yadav
EPI_ISL_15912916	The Microbiology laboratory, AUSL Romagna, Piesestina, Cesena, FC	Dipartimento di Medicina Specialistica Diagnostica e Sperimentale, University of Bologna Italy	Alessandra Scagliarini, Vittorio Sambri, MAria Elena Turba, Fabio Gentilini
EPI_ISL_15942296	Sexually Transmitted Diseases (STDs) outpatient service of Dermatology Unit, Fondazione IRCCS Ca' Granda Ospedale Maggiore Policlinico of Milan	Bioinformatic lab, Scientific Institute IRCCS E. Medea	Diego Forni, Rachele Cagliani, Manuela Sironi, Chiara Moltrasio, Luigia Venegoni, Eleonora Quattri, Angelo Marzano
EPI_ISL_15942637, EPI_ISL_15942638, EPI_ISL_15942885, EPI_ISL_15942886	Sexually Transmitted Diseases (STDs) outpatient service of Dermatology Unit, Fondazione IRCCS Ca' Granda Ospedale Maggiore Policlinico of Milan	Bioinformatic Lab, Scientific Institute IRCCS E. Medea	Diego Forni, Rachele Cagliani, Manuela Sironi, Chiara Moltrasio, Luigia Venegoni, Eleonora Quattri, Angelo Marzano
EPI_ISL_15955333, EPI_ISL_15955334, EPI_ISL_15955337	Kaiser Permanente Chino Hills Regional Reference Laboratories	Los Angeles County Public Health Laboratories	P. Hemarajata et al.
EPI_ISL_15955339	Los Angeles County Public Health Laboratories	Los Angeles County Public Health Laboratories	P. Hemarajata et al.
EPI_ISL_15955341, EPI_ISL_15955342, EPI_ISL_15955344, EPI_ISL_15955345, EPI_ISL_15955349, EPI_ISL_15955352	Kaiser Permanente Chino Hills Regional Reference Laboratories	Los Angeles County Public Health Laboratories	P. Hemarajata et al.
EPI_ISL_15969892	Oxford University Clinical Research Unit	Oxford University Clinical Research Unit	Nguyen Thanh Dung, Le Manh Hung, Huynh Thi Thuy Hoa, Tang Chi Thuong, Le Hong Nga, Nguyen Huu Hung, Nghiem My Ngoc, Nguyen Thi Thu Hong, Vo Truong Quy, Vu Thi Kim Thoa, Nguyen Thi Thanh, Phan Vinh Tho, Nguyen Le Nhu Tung, Le Mau Toan, Vo Minh Quang, Dinh Nguyen Huy Man, Nguyễn Tấn Phát, Trần Thị Lan Phương, Trần Thị Thanh Tâm, Phạm Thị Ngọc Thoa, Nguyen Hong Tam, Truong Thi Thanh Lan, Nguyen Thi Han Ny, Tran Tan Thanh, Le Thuy Thuy Khanh, Lam Minh Yen, Guy Thwaites, Louise Thwaites, Nguyen Van Vinh Chau, Nguyen To Anh, Le Van Tan
EPI_ISL_15972406, EPI_ISL_15972407, EPI_ISL_15972408, EPI_ISL_15972409	Laboratorio Central, Ministerio de Salud Córdoba	Laboratorio Central, Ministerio de Salud Córdoba	Castro, G.; Sicilia, P.; Poklepovich, T.; Campos, J.; Barbas, G.
EPI_ISL_15992095	LESP State of Mexico	Instituto de Diagnostico y Referencia Epidemiologicos (INDRE)	Abril Rodríguez-Maldonado; Claudia Wong-Arámbula; Felipe Arguijo-Perez; Helios Cárdenas-Hernández; Carmen Castro-Méndez; Lidia García-Torres; Ruth Madera-Sandoval; América Mandujano-Martínez; Nancy Martínez-Velázquez; Mireya Mederos-Michel; Angélica Pedraza-Meléndez; Joaquín Quiroz-Mercado; Daniel Regalado-Santiago; Silvia Rivero-Arredondo; Erika Sierra-Atanacio; Fernando González-Domínguez; Lucia Hernández-Rivas, Irma López-Martínez; Ernesto Ramírez-González; Maribel González-Villa
EPI_ISL_15992096	LESP Jalisco	Instituto de Diagnostico y Referencia Epidemiologicos (INDRE)	Abril Rodríguez-Maldonado; Claudia Wong-Arámbula; Felipe Arguijo-Perez; Helios Cárdenas-Hernández; Carmen Castro-Méndez; Lidia García-Torres; Ruth Madera-Sandoval; América Mandujano-Martínez; Nancy Martínez-Velázquez; Mireya Mederos-Michel; Angélica Pedraza-Meléndez; Joaquín Quiroz-Mercado; Daniel Regalado-Santiago; Silvia Rivero-Arredondo; Erika Sierra-Atanacio; Fernando González-Domínguez; Lucia Hernández-Rivas, Irma López-Martínez; Ernesto Ramírez-González; Maribel González-Villa
EPI_ISL_15992097	LESP Morelos	Instituto de Diagnostico y Referencia Epidemiologicos (INDRE)	Abril Rodríguez-Maldonado; Claudia Wong-Arámbula; Felipe Arguijo-Perez; Helios Cárdenas-Hernández; Carmen Castro-Méndez; Lidia García-Torres; Ruth Madera-Sandoval; América Mandujano-Martínez; Nancy Martínez-Velázquez; Mireya Mederos-Michel; Angélica Pedraza-Meléndez; Joaquín Quiroz-Mercado; Daniel Regalado-Santiago; Silvia Rivero-Arredondo; Erika Sierra-Atanacio; Fernando González-Domínguez; Lucia Hernández-Rivas, Irma López-Martínez; Ernesto Ramírez-González; Maribel González-Villa
EPI_ISL_15992098	LESP Nuevo Leon	Instituto de Diagnostico y Referencia Epidemiologicos (INDRE)	Abril Rodríguez-Maldonado; Claudia Wong-Arámbula; Felipe Arguijo-Perez; Helios Cárdenas-Hernández; Carmen Castro-Méndez; Lidia García-Torres; Ruth Madera-Sandoval; América Mandujano-Martínez; Nancy Martínez-Velázquez; Mireya Mederos-Michel; Angélica Pedraza-Meléndez; Joaquín Quiroz-Mercado; Daniel Regalado-Santiago; Silvia Rivero-Arredondo; Erika Sierra-Atanacio; Fernando González-Domínguez; Lucia Hernández-Rivas, Irma López-Martínez; Ernesto Ramírez-González; Maribel González-Villa
EPI_ISL_15992099	LESP Hidalgo	Instituto de Diagnostico y Referencia Epidemiologicos (INDRE)	Abril Rodríguez-Maldonado; Claudia Wong-Arámbula; Felipe Arguijo-Perez; Helios Cárdenas-Hernández; Carmen Castro-Méndez; Lidia García-Torres; Ruth Madera-Sandoval; América Mandujano-Martínez; Nancy Martínez-Velázquez; Mireya Mederos-Michel; Angélica Pedraza-Meléndez; Joaquín Quiroz-Mercado; Daniel Regalado-Santiago; Silvia Rivero-Arredondo; Erika Sierra-Atanacio; Fernando González-Domínguez; Lucia Hernández-Rivas, Irma López-Martínez;

			Ernesto Ramírez-González; Maribel González-Villa
EPI_ISL_15992100	LESP Campeche	Instituto de Diagnostico y Referencia Epidemiologicos (INDRE)	Abril Rodríguez-Maldonado; Claudia Wong-Arámbula; Felipe Arguijo-Perez; Helios Cárdenas-Hernández; Carmen Castro-Méndez; Lidia García-Torres; Ruth Madera-Sandoval; América Mandujano-Martínez; Nancy Martínez-Velázquez; Mireya Mederos-Michel; Angélica Pedraza-Meléndez; Joaquín Quiroz-Mercado; Daniel Regalado-Santiago; Silvia Rivero-Arredondo; Erika Sierra-Atanacio; Fernando González-Domínguez; Lucia Hernández-Rivas, Irma López-Martínez; Ernesto Ramírez-González; Maribel González-Villa
EPI_ISL_15992101	LESP Tlaxcala	Instituto de Diagnostico y Referencia Epidemiologicos (INDRE)	Abril Rodríguez-Maldonado; Claudia Wong-Arámbula; Felipe Arguijo-Perez; Helios Cárdenas-Hernández; Carmen Castro-Méndez; Lidia García-Torres; Ruth Madera-Sandoval; América Mandujano-Martínez; Nancy Martínez-Velázquez; Mireya Mederos-Michel; Angélica Pedraza-Meléndez; Joaquín Quiroz-Mercado; Daniel Regalado-Santiago; Silvia Rivero-Arredondo; Erika Sierra-Atanacio; Fernando González-Domínguez; Lucia Hernández-Rivas, Irma López-Martínez; Ernesto Ramírez-González; Maribel González-Villa
EPI_ISL_15992102	LESP Aguascalientes	Instituto de Diagnostico y Referencia Epidemiologicos (INDRE)	Abril Rodríguez-Maldonado; Claudia Wong-Arámbula; Felipe Arguijo-Perez; Helios Cárdenas-Hernández; Carmen Castro-Méndez; Lidia García-Torres; Ruth Madera-Sandoval; América Mandujano-Martínez; Nancy Martínez-Velázquez; Mireya Mederos-Michel; Angélica Pedraza-Meléndez; Joaquín Quiroz-Mercado; Daniel Regalado-Santiago; Silvia Rivero-Arredondo; Erika Sierra-Atanacio; Fernando González-Domínguez; Lucia Hernández-Rivas, Irma López-Martínez; Ernesto Ramírez-González; Maribel González-Villa
EPI_ISL_15992103, EPI_ISL_15992104	LESP San Luis Potosi	Instituto de Diagnostico y Referencia Epidemiologicos (INDRE)	Abril Rodríguez-Maldonado; Claudia Wong-Arámbula; Felipe Arguijo-Perez; Helios Cárdenas-Hernández; Carmen Castro-Méndez; Lidia García-Torres; Ruth Madera-Sandoval; América Mandujano-Martínez; Nancy Martínez-Velázquez; Mireya Mederos-Michel; Angélica Pedraza-Meléndez; Joaquín Quiroz-Mercado; Daniel Regalado-Santiago; Silvia Rivero-Arredondo; Erika Sierra-Atanacio; Fernando González-Domínguez; Lucia Hernández-Rivas, Irma López-Martínez; Ernesto Ramírez-González; Maribel González-Villa
EPI_ISL_16012468, EPI_ISL_16012469, EPI_ISL_16012470, EPI_ISL_16012471, EPI_ISL_16012472, EPI_ISL_16012473, EPI_ISL_16012474, EPI_ISL_16012475, EPI_ISL_16012476, EPI_ISL_16012477, EPI_ISL_16012478, EPI_ISL_16012479, EPI_ISL_16012480, EPI_ISL_16012481, EPI_ISL_16012482, EPI_ISL_16012483, EPI_ISL_16012484, EPI_ISL_16012485, EPI_ISL_16012486, EPI_ISL_16012487, EPI_ISL_16012488, EPI_ISL_16012489, EPI_ISL_16012490, EPI_ISL_16012491, EPI_ISL_16012492, EPI_ISL_16012493, EPI_ISL_16012494, EPI_ISL_16012495, EPI_ISL_16012496, EPI_ISL_16012497, EPI_ISL_16012498, EPI_ISL_16012499, EPI_ISL_16012500, EPI_ISL_16012501, EPI_ISL_16012502, EPI_ISL_16012503, EPI_ISL_16012504, EPI_ISL_16012505, EPI_ISL_16012506, EPI_ISL_16012507, EPI_ISL_16012508, EPI_ISL_16012509, EPI_ISL_16012510, EPI_ISL_16012511, EPI_ISL_16012512, EPI_ISL_16012513, EPI_ISL_16012514, EPI_ISL_16012515, EPI_ISL_16012516, EPI_ISL_16012517, EPI_ISL_16012518, EPI_ISL_16012519, EPI_ISL_16012520, EPI_ISL_16012521, EPI_ISL_16012522, EPI_ISL_16012523, EPI_ISL_16012524, EPI_ISL_16012525, EPI_ISL_16012526, EPI_ISL_16012527, EPI_ISL_16012528, EPI_ISL_16012529, EPI_ISL_16012530, EPI_ISL_16012531, EPI_ISL_16012532, EPI_ISL_16012533, EPI_ISL_16012534, EPI_ISL_16012535, EPI_ISL_16012536			
see above	National Virus Reference Laboratory	National Virus Reference Laboratory	Gabriel Gonzalez, Michael Carr, Brian Keogan, Jose Maria Urtasun Elizari, Jonathan Dean, Daniel Hare, Cillian F De Gascun
EPI_ISL_16104842, EPI_ISL_16104843, EPI_ISL_16104844, EPI_ISL_16104845, EPI_ISL_16104846, EPI_ISL_16104847, EPI_ISL_16104848, EPI_ISL_16104849, EPI_ISL_16104850, EPI_ISL_16104851, EPI_ISL_16104852, EPI_ISL_16104853, EPI_ISL_16104854, EPI_ISL_16104855, EPI_ISL_16104856, EPI_ISL_16104857, EPI_ISL_16104858, EPI_ISL_16104859, EPI_ISL_16104860, EPI_ISL_16104861, EPI_ISL_16104862, EPI_ISL_16104863, EPI_ISL_16104864, EPI_ISL_16104865, EPI_ISL_16104866, EPI_ISL_16104867, EPI_ISL_16104868, EPI_ISL_16104869, EPI_ISL_16104870, EPI_ISL_16104871, EPI_ISL_16104872, EPI_ISL_16104873, EPI_ISL_16104874, EPI_ISL_16104875, EPI_ISL_16104876, EPI_ISL_16104877, EPI_ISL_16104878, EPI_ISL_16104879, EPI_ISL_16104880, EPI_ISL_16104881, EPI_ISL_16104882, EPI_ISL_16104883, EPI_ISL_16104884, EPI_ISL_16104885, EPI_ISL_16104886, EPI_ISL_16104887, EPI_ISL_16104888, EPI_ISL_16104889, EPI_ISL_16104890, EPI_ISL_16104891, EPI_ISL_16104892, EPI_ISL_16104893, EPI_ISL_16104894, EPI_ISL_16104895, EPI_ISL_16104896, EPI_ISL_16104897, EPI_ISL_16104898, EPI_ISL_16104899, EPI_ISL_16104900, EPI_ISL_16104901, EPI_ISL_16104902, EPI_ISL_16104903, EPI_ISL_16104904, EPI_ISL_16104905, EPI_ISL_16104906, EPI_ISL_16104907, EPI_ISL_16104908, EPI_ISL_16104909, EPI_ISL_16104910, EPI_ISL_16104911, EPI_ISL_16104912, EPI_ISL_16104913, EPI_ISL_16104914, EPI_ISL_16104915, EPI_ISL_16104916, EPI_ISL_16104917, EPI_ISL_16104918, EPI_ISL_16104919, EPI_ISL_16104920, EPI_ISL_16104921, EPI_ISL_16104922, EPI_ISL_16116727, EPI_ISL_16116728, EPI_ISL_16116729, EPI_ISL_16116730, EPI_ISL_16116733, EPI_ISL_16116734, EPI_ISL_16116735, EPI_ISL_16116736, EPI_ISL_16116737, EPI_ISL_16116738, EPI_ISL_16116739, EPI_ISL_16116740, EPI_ISL_16116741, EPI_ISL_16116742, EPI_ISL_16116743, EPI_ISL_16116744, EPI_ISL_16116745, EPI_ISL_16116746, EPI_ISL_16116747, EPI_ISL_16116748, EPI_ISL_16116749, EPI_ISL_16116750, EPI_ISL_16116751, EPI_ISL_16116752, EPI_ISL_16116754, EPI_ISL_16116755, EPI_ISL_16116756, EPI_ISL_16116757, EPI_ISL_16116758, EPI_ISL_16116759, EPI_ISL_16116760, EPI_ISL_16116761, EPI_ISL_16116762, EPI_ISL_16116763, EPI_ISL_16116764, EPI_ISL_16116765			
see above	Laboratorio de Referencia Nacional de Viruas Inmunoprevenibles. Centro Nacional de Salud Publica. Instiuto Nacional de Salud	Laboratorio de Referencia Nacional de Viruas Inmunoprevenibles. Centro Nacional de Salud Publica. Instiuto Nacional de Salud	Carlos Patricio Padilla Rojas, Carmen Verónica Hurtado Vela, Juana Iris Silva Molina, Luis Bárcena Flores, Víctor Jiménez Vásquez, Alicia Elizabeth Núñez Llanos, Wendy Lizarraga Olivares, Luren Nieves Sevilla Catañeda, Kelly Vanessa Izarra Rojas, Karla Vasquez Cajachahua, Steve Vladimir Acedo Lazo, Omar Alberto Cáceres Rey, Henri Bailón Calderón, Priscila Nayu Lope Pari, Nancy Rojas Serrano, Gloria Arotinco Garayar. Equipo de vigilancia genomica del Instituto Nacional de Salud.
EPI_ISL_16138920, EPI_ISL_16138921, EPI_ISL_16138923, EPI_ISL_16138924, EPI_ISL_16138925, EPI_ISL_16138926	California Department of Public Health	California Department of Public Health	Kath, C., Haw, M., Espinosa, A., and Hacker, J.
EPI_ISL_16190089, EPI_ISL_16190090, EPI_ISL_16190092, EPI_ISL_16190094, EPI_ISL_16190099	Rush University Medical Center	RIPHL at Rush University Medical Center	Stefan Green, Kevin Kunstman, Hannah Barbian, Felix Araujo Perez, Edith Perez, Sofiya Bobrovska, Alyse Kittner, Cecilia Chau, Giancarlo Balangue, Lok Yiu Ashley Wu, Mary Hayden, Joyce Houlihan, Diane Springer, Nicholas Moore
EPI_ISL_16233781	Complejo Hospitalario Universitario de Pontevedra	Microbiology Department. Complexo Hospitalario Universitario de Vigo	Daviña C, Pizcueta J, Trigo M, Perez-Castro S
EPI_ISL_16233782, EPI_ISL_16233783, EPI_ISL_16233784, EPI_ISL_16233785, EPI_ISL_16233786, EPI_ISL_16233787, EPI_ISL_16233788	Microbiology Department. Complexo Hospitalario Universitario de Vigo	Microbiology Department. Complexo Hospitalario Universitario de Vigo	Daviña C, Pizcueta J, Perez-Castro S
EPI_ISL_16260351	Centre Médical de l'Institut Pasteur	Cellule d'Intervention Biologique d'Urgence, Institut Pasteur	Charlotte Balière, Véronique Hourdel, Aurelia Kwasiborski, Quentin Grassin, Maxence Feher, Damien Hoinard, Jessica Vanhomwegen, Fabien Taieb, Paul-Henri Consigny, Jean-Claude Manuguerra, India Leclercq, Christophe Batéjat, Valérie Caro
EPI_ISL_16260402	Cellule d'Intervention Biologique d'Urgence, Institut Pasteur	Cellule d'Intervention Biologique d'Urgence, Institut Pasteur	Charlotte Balière, Véronique Hourdel, Aurelia Kwasiborski, Quentin Grassin, Maxence Feher, Damien Hoinard, Jessica Vanhomwegen, Fabien Taieb, Paul-Henri Consigny, Jean-Claude Manuguerra, India Leclercq, Christophe Batéjat, Valérie Caro
EPI_ISL_16350819	Laboratorio de Biologia Molecular y Biotecnologia / Facultad de ciencias de la salud, Universidad Tecnologica de Pereira	Laboratorio de Biologia Molecular y Biotecnologia / Facultad de ciencias de la salud, Universidad Tecnologica de Pereira	Orjuela,M., Tabares,F.A., Anacona,J.D., Lopez,P.A., Zuluaga-Velez,A. and Sepulveda-Arias,J.C.
EPI_ISL_16467111	IRCCS Sacro Cuore Don Calabria Hospital, Department of Infectious, Tropical Diseases & Microbiology	IRCCS Sacro Cuore Don Calabria Hospital, Department of Infectious, Tropical Diseases & Microbiology	Michela Deiana, Denise Lavezzari, Silvia Accordini, Concetta Castilletti, Antonio Mori, Elena Pomari, Chiara Piubelli
EPI_ISL_16510131, EPI_ISL_16510132, EPI_ISL_16510134, EPI_ISL_16510136, EPI_ISL_16510138, EPI_ISL_16510140, EPI_ISL_16510141, EPI_ISL_16510143, EPI_ISL_16510145, EPI_ISL_16510147, EPI_ISL_16510148, EPI_ISL_16510150, EPI_ISL_16510153, EPI_ISL_16510154, EPI_ISL_16510155, EPI_ISL_16510156, EPI_ISL_16510157, EPI_ISL_16510158, EPI_ISL_16510159, EPI_ISL_16510162, EPI_ISL_16510163, EPI_ISL_16510164, EPI_ISL_16510165, EPI_ISL_16510166, EPI_ISL_16510167, EPI_ISL_16510168, EPI_ISL_16510169, EPI_ISL_16510170, EPI_ISL_16510171, EPI_ISL_16510172, EPI_ISL_16510173, EPI_ISL_16510174, EPI_ISL_16510175, EPI_ISL_16510177, EPI_ISL_16510178, EPI_ISL_16510179, EPI_ISL_16510180, EPI_ISL_16510181, EPI_ISL_16510182, EPI_ISL_16510183, EPI_ISL_16510184, EPI_ISL_16510185			
see above	National Virus Reference Laboratory	National Virus Reference Laboratory	Gabriel Gonzalez, Michael Carr, Brian Keogan, Jose Maria Urtasun Elizari, Jonathan Dean, Daniel Hare, Cillian F De Gascun
EPI_ISL_16526309	Direccion de Investigacion en Salud Publica, Instituto Nacional de Salud	Direccion de Investigacion en Salud Publica, Instituto Nacional de Salud	Laiton-Donato,K.D., Franco,C.E., Alvarez-Diaz,D.A., Ruiz-Moreno,H.A., Prada,D.A., Rosales,A. and Mercado-Reyes,M.M.
EPI_ISL_16645206	Division of High-risk Pathogens, Korea Disease Control and Prevention Agency	Division of High-risk Pathogens, Korea Disease Control and Prevention Agency	Rhie,G.-e.
EPI_ISL_16645207, EPI_ISL_16645208, EPI_ISL_16645209, EPI_ISL_16645214, EPI_ISL_16645216, EPI_ISL_16645218, EPI_ISL_16645219, EPI_ISL_16645220, EPI_ISL_16645221, EPI_ISL_16645223, EPI_ISL_16645224, EPI_ISL_16645226, EPI_ISL_16645227, EPI_ISL_16645228			
see above	Antioquia, Laboratorio Departamental de Salud Publica de Antioquia	Antioquia, Laboratorio Departamental de Salud Publica de Antioquia	Betancur,I.I.B., Velarde-Hoyos,C.-A.C.V., Gomez,R.R.G. and Mercado-Reyes,M.M.R.
EPI_ISL_16645229	Environment and Infectious Risks Unit, Insitut Pasteur	Environment and Infectious Risks Unit, Insitut Pasteur	Baliere,C., Hourdel,V., Kwasiborski,A., Grassin,Q., Feher,M., Hoinard,D., Vanhomwegen,J., Taieb,F., Consigny,P.-H., Manuguerra,J.-C., Leclercq,I., Batejat,C. and Caro,V.
EPI_ISL_16650246, EPI_ISL_16650247, EPI_ISL_16650248, EPI_ISL_16650249, EPI_ISL_16650251, EPI_ISL_16650260	Laboratorio Central de Saude Publica do Estado de Minas Gerais (Lacen-MG)	Laboratorio Central de Saude Publica do Estado de Minas Gerais (Lacen-MG)	Felipe Campos de Melo Iani, Ludmila Oliveira Lamounier, Luiz Marcelo Ribeiro Tomé, Natália Rocha Guimarães,Talita Emile Ribeiro Adelino.
EPI_ISL_16679206, EPI_ISL_16679208	Los Angeles County Public Health Laboratories	Los Angeles County Public Health Laboratories	P. Hemarajata et al.
EPI_ISL_16679210, EPI_ISL_16679216, EPI_ISL_16679217, EPI_ISL_16679219, EPI_ISL_16679220, EPI_ISL_16679222, EPI_ISL_16679225, EPI_ISL_16679226, EPI_ISL_16679235	Kaiser Permanente Chino Hills Regional Reference Laboratories	Los Angeles County Public Health Laboratories	P. Hemarajata et al.
EPI_ISL_16679238, EPI_ISL_16679240	Los Angeles County Public Health Laboratories	Los Angeles County Public Health Laboratories	P. Hemarajata et al.
EPI_ISL_16679258, EPI_ISL_16679273, EPI_ISL_16679276, EPI_ISL_16679277, EPI_ISL_16679279, EPI_ISL_16679281, EPI_ISL_16679283, EPI_ISL_16679284, EPI_ISL_16679285, EPI_ISL_16679286, EPI_ISL_16679287, EPI_ISL_16679293, EPI_ISL_16679298, EPI_ISL_16679301, EPI_ISL_16679304			
see above	California Department of Public Health	California Department of Public Health	Probert,W., Espinosa,A., Kath,C., Haw,M., O'Neil,R., Bell,J. and Hacker,J.
EPI_ISL_16871158, EPI_ISL_16871160, EPI_ISL_16871161, EPI_ISL_16871162, EPI_ISL_16871163	Laboratorio de Enterovirus, Instituto Oswaldo Cruz, Fiocruz	Instituto Oswaldo Cruz FIOCRUZ - Laboratory of Respiratory Viruses and Measles (LVRS)	Paola Resende, Elisa Cavalcante Pereira, Bruna Mendonça da Silva, Jéssica Graça Macedo de Carvalho, Larissa Macedo Pinto, Victor Guimaraes, Marilda Siqueira, Renan da Silva Faustino, Marilia Santini, Edson Elias da Silva on behalf of the Fiocruz Genomic Surveillance Network
EPI_ISL_16926988, EPI_ISL_16926991, EPI_ISL_16926994, EPI_ISL_16926997, EPI_ISL_16927000, EPI_ISL_16927003, EPI_ISL_16927007, EPI_ISL_16927010, EPI_ISL_16927013, EPI_ISL_16927016, EPI_ISL_16927018, EPI_ISL_16927021			
see above	Naval Infectious Diseases Diagnostic Laboratory	Naval Medical Research Center Biological Defense Research Directorate	Logan J. Voegtly, Gregory K. Rice, Adrian Pakey, Andrea E. Luquette, Maren C. Fitzpatrick, Hannah M. Drumm, Victor Sugiharto, Hua-Wei Chen, Francisco Malagon, Regina Z. Cer, Kimberly A. Bishop-Lilly
EPI_ISL_16930148, EPI_ISL_16930151, EPI_ISL_16930154, EPI_ISL_16930171, EPI_ISL_16930174	California Department of Public Health	California Department of Public Health	Probert,W., Espinosa,A., Kath,C., Haw,M., O'Neil,R., Bell,J. and Hacker,J.
EPI_ISL_16946400	Division de Microbiologia, Hospital Nacional de Niños Carlos Saenz Herrera	Instituto Costarricense de Investigacion y Enseñanza en Nutricion y Salud, Inciensa	Diana Cantillo, Hillary Serrano, Ana Isela Ruiz, Gustavo Vega, Claudio Soto-Garita, Adriana Godinez, Estela Cordero, Melany Calderon, Francisco Duarte
EPI_ISL_16955156	CT Department of Public Health	CT Department of Public Health	Claire Pearson, Tu N. Nguyen, Kutluhan Incekara, Neranjan V. Perera
EPI_ISL_16955240, EPI_ISL_16955243, EPI_ISL_16955247, EPI_ISL_16955270, EPI_ISL_16955271, EPI_ISL_16955272, EPI_ISL_16955275, EPI_ISL_16955278, EPI_ISL_16955279, EPI_ISL_16955283, EPI_ISL_16955289, EPI_ISL_16955290			
see above	Public Health Laboratory, NYC Department of Health and Mental Hygiene	Public Health Laboratory, NYC Department of Health and Mental Hygiene	Wang,J.C., Amin,H.S., Clabby,T.T., Taki,F., Su,M., Rahat,A., De La Cruz,N., Olsen,A., Thi,C., Silver,S., Akther,S., Chowdhury,M., Omoregie,E. and Hughes,S.
EPI_ISL_16955293	Laboratory of Virology, University Hospitals of Geneva	Laboratory of Virology, University Hospitals of Geneva	Laubscher,F., Chudzinsk,V., Schibler,M., Kaiser,L. and Renzoni,A.
EPI_ISL_16955294, EPI_ISL_16955297, EPI_ISL_16955299, EPI_ISL_16955300, EPI_ISL_16955302, EPI_ISL_16955305	Public Health Laboratory, NYC Department of Health and Mental Hygiene	Public Health Laboratory, NYC Department of Health and Mental Hygiene	Wang,J.C., Amin,H.S., Clabby,T.T., Taki,F., Su,M., Rahat,A., De La Cruz,N., Olsen,A., Thi,C., Silver,S., Akther,S., Chowdhury,M., Omoregie,E. and Hughes,S.
EPI_ISL_16985950, EPI_ISL_16985951, EPI_ISL_16985952, EPI_ISL_16985954, EPI_ISL_16985955, EPI_ISL_16985956, EPI_ISL_16985957, EPI_ISL_16985958, EPI_ISL_16985959, EPI_ISL_16985960, EPI_ISL_16985961, EPI_ISL_16985962, EPI_ISL_16985963, EPI_ISL_16985964, EPI_ISL_16985965, EPI_ISL_16985966, EPI_ISL_16985967, EPI_ISL_16985968, EPI_ISL_16985969, EPI_ISL_16985970,			

EPI_ISL_16985971, EPI_ISL_16985972			
see above	National Virus Reference Laboratory	National Virus Reference Laboratory	Gabriel Gonzalez, Michael Carr, Emer O'Byrne, Weronika Banka, Brian Keogan, Jose Maria Urtasun Elizari, Jonathan Dean, Daniel Hare, Cillian F De Gascun
EPI_ISL_16997394, EPI_ISL_16997397	California Department of Public Health	California Department of Public Health	Probert,W., Espinosa,A., Kath,C., Haw,M., O'Neil,R., Bell,J. and Hacker,J.
EPI_ISL_16997407, EPI_ISL_16997411	Los Angeles County Public Health Laboratories	Los Angeles County Public Health Laboratories	P. Hemarajata et al.
EPI_ISL_16997413, EPI_ISL_16997417, EPI_ISL_16997418, EPI_ISL_16997419, EPI_ISL_16997421, EPI_ISL_16997425, EPI_ISL_16997427, EPI_ISL_16997432, EPI_ISL_16997433, EPI_ISL_16997434, EPI_ISL_16997435, EPI_ISL_16997437, EPI_ISL_16997440, EPI_ISL_16997442, EPI_ISL_16997443, EPI_ISL_16997445, EPI_ISL_16997446			
see above	Kaiser Permanente Chino Hills Regional Reference Laboratories	Los Angeles County Public Health Laboratories	P. Hemarajata et al.
EPI_ISL_16997460, EPI_ISL_16997468	Quest Diagnostics Nichols Institute	Los Angeles County Public Health Laboratories	P. Hemarajata et al.
EPI_ISL_16997470	UCLA Clinical Micro Lab	Los Angeles County Public Health Laboratories	P. Hemarajata et al.
EPI_ISL_16999059, EPI_ISL_16999060, EPI_ISL_16999064, EPI_ISL_16999065, EPI_ISL_16999067, EPI_ISL_16999068, EPI_ISL_16999069, EPI_ISL_16999070, EPI_ISL_16999071, EPI_ISL_16999073, EPI_ISL_16999076, EPI_ISL_16999077, EPI_ISL_16999078, EPI_ISL_16999079, EPI_ISL_16999081, EPI_ISL_16999084, EPI_ISL_16999085, EPI_ISL_16999087, EPI_ISL_16999091, EPI_ISL_16999095, EPI_ISL_16999098, EPI_ISL_16999099, EPI_ISL_16999100, EPI_ISL_16999101, EPI_ISL_16999102, EPI_ISL_16999104, EPI_ISL_16999105, EPI_ISL_16999108, EPI_ISL_16999110, EPI_ISL_16999111, EPI_ISL_16999113, EPI_ISL_16999114, EPI_ISL_16999115, EPI_ISL_16999116, EPI_ISL_16999117, EPI_ISL_16999120, EPI_ISL_16999121, EPI_ISL_16999124, EPI_ISL_16999125, EPI_ISL_16999127, EPI_ISL_16999128, EPI_ISL_16999129, EPI_ISL_16999131, EPI_ISL_16999137, EPI_ISL_16999138, EPI_ISL_16999139			
see above	Laboratorio de Referencia Nacional de Virus Inmunoprevenibles. Centro Nacional de Salud Publica. Instiuto Nacional de Salud	Equipo de Vigilancia Genomica. Area de Innovación y Desarrollo. Centro Nacional de Salud Publica. Instiuto Nacional de Salud	Carlos Patricio Padilla Rojas, Carmen Verónica Hurtado Vela, Juana Iris Silva Molina, Luis Bárcena Flores, Víctor Jiménez Vásquez, Alicia Elizabeth Núñez Llanos, Wendy Lizarraga Olivares, Luren Nieves Sevilla Catañeda, Kelly Vanessa Izarra Rojas, Karla Vasquez Cajachahua, Steve Vladimir Acedo Lazo, Omar Alberto Cáceres Rey, Henri Bailón Calderón, Priscila Nayu Lope Pari, Nancy Rojas Serrano, Gloria Arotinco Garayar. Equipo de Vigilancia Genomica del Instituto Nacional de Salud.
EPI_ISL_17008293, EPI_ISL_17008294, EPI_ISL_17008295, EPI_ISL_17008296	Tokyo Metropolitan Institute of Public Health, Department of Microbiology	Tokyo Metropolitan Institute of Public Health, Department of Microbiology	Kasuya,F., Negishi,A., Kumagai,R., Hasegawa,M., Fujiwara,T., Miyake,H., Nagashima,M. and Sadamasu,K.
EPI_ISL_17012023, EPI_ISL_17012028, EPI_ISL_17012031, EPI_ISL_17012033, EPI_ISL_17012036, EPI_ISL_17012037, EPI_ISL_17012038, EPI_ISL_17012039, EPI_ISL_17012042, EPI_ISL_17012043, EPI_ISL_17012047			
see above	Laboratorio de Virus Exantematicos, Gastroentéricos y Otros Transmitidos por Vectores	Centro de Referencia Nacional de Genomica, Secuenciacion y Bioinformatica GENSBIO, INSPI-CZ9	Andrés Carrazco, Silvia Salgado, Diana Gutiérrez, Damaris Alarcón, Andrés Herrera, Andrés Tinizaray, Martha Sánchez, Johanna Parrales, Diego Morales, Jorge Bejarano, Leandro Patiño.
EPI_ISL_17012082, EPI_ISL_17012084, EPI_ISL_17012089, EPI_ISL_17012090, EPI_ISL_17012091, EPI_ISL_17012092, EPI_ISL_17012094, EPI_ISL_17012097, EPI_ISL_17012102, EPI_ISL_17012109	Laboratorio de Virus Exantematicos, Gastroentéricos y Otros Transmitidos por Vectores	Centro de Referencia Nacional de Genomica, Secuenciacion y Bioinformatica GENSBIO, INSPI-CZ9	Andrés Carrazco-Motalvo, Silvia Salgado, Diana Gutiérrez, Damaris Alarcón, Andrés Herrera, Andrés Tinizaray, Ruth Gómez, Martha Sánchez, Johanna Parrales, Diego Morales, Jorge Bejarano, Leandro Patiño.
EPI_ISL_17019462	Parkland Health and Hospital System	Dallas County Health & Human Services Public Health Laboratory	Kabir, Farruk; Plaisance, Erin; Stringer, Joey; Short, Luke.
EPI_ISL_17048204, EPI_ISL_17048205, EPI_ISL_17048206, EPI_ISL_17048207	Laboratorio Central de Saude Publica do Estado da Bahia (LACEN/BA)	Laboratory of Respiratory Viruses and Measles, Oswaldo Cruz Institute, FIOCRUZ	Paola Resende, Fernando Motta, Elisa Cavalcante Pereira, Bruna Mendonça da Silva, Jéssica Graça Macedo de Carvalho, Larissa Macedo Pinto, Victor Guimaraes, Felicidade Pereira, Marilda Siqueira, Renan da Silva Faustino, Marilia Santini, Edson Elias da Silva on behalf of the Fiocruz COVID-19 Genomic Surveillance Network
EPI_ISL_17048208	Laboratorio de Enterovirus, Instituto Oswaldo Cruz, Fiocruz	Laboratory of Respiratory Viruses and Measles, Oswaldo Cruz Institute, FIOCRUZ	Paola Resende, Elisa Cavalcante Pereira, Bruna Mendonça da Silva, Jéssica Graça Macedo de Carvalho, Larissa Macedo Pinto, Victor Guimaraes, Marilda Siqueira, Renan da Silva Faustino, Marilia Santini, Edson Elias da Silva on behalf of the Fiocruz Genomic Surveillance Network
EPI_ISL_17085703, EPI_ISL_17085706	Public Health Laboratory, NYC Department of Health and Mental Hygiene	Public Health Laboratory, NYC Department of Health and Mental Hygiene	Wang,J.C., Amin,H.S., Clabby,T.T., Taki,F., Su,M., Rahat,A., De La Cruz,N., Olsen,A., Thi,C., Silver,S., Akther,S., Chowdhury,M., Omoregie,E. and Hughes,S.
EPI_ISL_17085717, EPI_ISL_17085725, EPI_ISL_17085746, EPI_ISL_17085748, EPI_ISL_17085753, EPI_ISL_17085769, EPI_ISL_17085776, EPI_ISL_17085782, EPI_ISL_17085784, EPI_ISL_17085787, EPI_ISL_17085790, EPI_ISL_17085792, EPI_ISL_17085795, EPI_ISL_17085797, EPI_ISL_17085798, EPI_ISL_17085920, EPI_ISL_17085921, EPI_ISL_17085922, EPI_ISL_17085924, EPI_ISL_17085925, EPI_ISL_17085926, EPI_ISL_17085927, EPI_ISL_17085933, EPI_ISL_17085934, EPI_ISL_17085935, EPI_ISL_17085942, EPI_ISL_17085943, EPI_ISL_17085945, EPI_ISL_17085946, EPI_ISL_17085948, EPI_ISL_17085950, EPI_ISL_17085952, EPI_ISL_17085954, EPI_ISL_17085955, EPI_ISL_17085959, EPI_ISL_17085960, EPI_ISL_17085961			
see above	Public Health Laboratory, NYC Department of Health and Mental Hygiene	Public Health Laboratory, NYC Department of Health and Mental Hygiene	Clabby,T.T., Amin,H.S., Wang,J.C., Taki,F., Su,M., Rahat,A., De La Cruz,N., Olsen,A., Thi,C., Silver,S., Akther,S., Chowdhury,M., Omoregie,E. and Hughes,S.
EPI_ISL_17085975, EPI_ISL_17085977, EPI_ISL_17085984, EPI_ISL_17085986, EPI_ISL_17085987, EPI_ISL_17085988, EPI_ISL_17085989, EPI_ISL_17085990, EPI_ISL_17085991, EPI_ISL_17085993, EPI_ISL_17085995, EPI_ISL_17085996, EPI_ISL_17085997, EPI_ISL_17085998, EPI_ISL_17085999, EPI_ISL_17086000, EPI_ISL_17086001, EPI_ISL_17086002, EPI_ISL_17086003, EPI_ISL_17086005, EPI_ISL_17086006, EPI_ISL_17086007, EPI_ISL_17086009, EPI_ISL_17086010, EPI_ISL_17086011, EPI_ISL_17086012, EPI_ISL_17086013, EPI_ISL_17086015, EPI_ISL_17086018, EPI_ISL_17086021, EPI_ISL_17086038, EPI_ISL_17086040, EPI_ISL_17086046, EPI_ISL_17086052, EPI_ISL_17086061			
see above	Public Health Laboratory, NYC Department of Health and Mental Hygiene	Public Health Laboratory, NYC Department of Health and Mental Hygiene	Amin,H.S., Clabby,T.T., Wang,J.C., Taki,F., Su,M., Rahat,A., De La Cruz,N., Olsen,A., Thi,C., Silver,S., Akther,S., Chowdhury,M., Omoregie,E. and Hughes,S.
EPI_ISL_17086062, EPI_ISL_17086066, EPI_ISL_17086078, EPI_ISL_17086082, EPI_ISL_17086083, EPI_ISL_17086102, EPI_ISL_17086111, EPI_ISL_17086112, EPI_ISL_17086114, EPI_ISL_17086115, EPI_ISL_17086116, EPI_ISL_17086117, EPI_ISL_17086118, EPI_ISL_17086119, EPI_ISL_17086120, EPI_ISL_17086121, EPI_ISL_17086123, EPI_ISL_17086124, EPI_ISL_17086125, EPI_ISL_17086126, EPI_ISL_17086128, EPI_ISL_17086131, EPI_ISL_17086133, EPI_ISL_17086134, EPI_ISL_17086135, EPI_ISL_17086138, EPI_ISL_17086139, EPI_ISL_17086141, EPI_ISL_17086142, EPI_ISL_17086143, EPI_ISL_17086144, EPI_ISL_17086145, EPI_ISL_17086146, EPI_ISL_17086147, EPI_ISL_17086148, EPI_ISL_17086151, EPI_ISL_17086152, EPI_ISL_17086155, EPI_ISL_17086161, EPI_ISL_17086186, EPI_ISL_17086188, EPI_ISL_17086246, EPI_ISL_17086253, EPI_ISL_17086255, EPI_ISL_17086257, EPI_ISL_17086263, EPI_ISL_17086264			
see above	Public Health Laboratory, NYC Department of Health and Mental Hygiene	Public Health Laboratory, NYC Department of Health and Mental Hygiene	Wang,J.C., Amin,H.S., Clabby,T.T., Taki,F., Su,M., Rahat,A., De La Cruz,N., Olsen,A., Thi,C., Silver,S., Akther,S., Chowdhury,M., Omoregie,E. and Hughes,S.
EPI_ISL_17086282, EPI_ISL_17086298, EPI_ISL_17086321, EPI_ISL_17086323, EPI_ISL_17104205, EPI_ISL_17104207, EPI_ISL_17104214, EPI_ISL_17104247, EPI_ISL_17104354, EPI_ISL_17104410, EPI_ISL_17104419, EPI_ISL_17104441, EPI_ISL_17104445, EPI_ISL_17104512, EPI_ISL_17104527, EPI_ISL_17104537, EPI_ISL_17104539, EPI_ISL_17104541, EPI_ISL_17104542, EPI_ISL_17104546, EPI_ISL_17104547, EPI_ISL_17104551, EPI_ISL_17104552, EPI_ISL_17104556, EPI_ISL_17104557, EPI_ISL_17104560, EPI_ISL_17104561, EPI_ISL_17104563, EPI_ISL_17104564, EPI_ISL_17104565, EPI_ISL_17104567, EPI_ISL_17104569, EPI_ISL_17104570, EPI_ISL_17104573, EPI_ISL_17104574, EPI_ISL_17104577, EPI_ISL_17104579, EPI_ISL_17104581, EPI_ISL_17104583, EPI_ISL_17104585, EPI_ISL_17104586, EPI_ISL_17104587, EPI_ISL_17104588, EPI_ISL_17104589, EPI_ISL_17104590, EPI_ISL_17104591, EPI_ISL_17104592, EPI_ISL_17104593, EPI_ISL_17104595, EPI_ISL_17104597, EPI_ISL_17104621, EPI_ISL_17104623, EPI_ISL_17104626, EPI_ISL_17104628, EPI_ISL_17104631, EPI_ISL_17104632, EPI_ISL_17104637, EPI_ISL_17104642, EPI_ISL_17104644, EPI_ISL_17104646, EPI_ISL_17104647, EPI_ISL_17104649, EPI_ISL_17104653			
see above	Public Health Laboratory, NYC Department of Health and Mental Hygiene	Public Health Laboratory, NYC Department of Health and Mental Hygiene	Clabby,T.T., Amin,H.S., Wang,J.C., Taki,F., Su,M., Rahat,A., De La Cruz,N., Olsen,A., Thi,C., Silver,S., Akther,S., Chowdhury,M., Omoregie,E. and Hughes,S.
EPI_ISL_17118608, EPI_ISL_17118631, EPI_ISL_17118637, EPI_ISL_17118643, EPI_ISL_17118672, EPI_ISL_17118678, EPI_ISL_17118679, EPI_ISL_17118693	Public Health Laboratory, NYC Department of Health and Mental Hygiene	Public Health Laboratory, NYC Department of Health and Mental Hygiene	Wang,J.C., Amin,H.S., Clabby,T.T., Taki,F., Su,M., Rahat,A., De La Cruz,N., Olsen,A., Thi,C., Silver,S., Akther,S., Chowdhury,M., Omoregie,E. and Hughes,S.
EPI_ISL_17118738, EPI_ISL_17118739	Environmental, Agricultural, and Occupational Health, University of Nebraska Medical Center	Environmental, Agricultural, and Occupational Health, University of Nebraska Medical Center	Pentella,M., Chapman,R.C., Stapleton,J., Meier,J., Xiang,J., Li,M., Reeb,V., Benfer,J., Eveland,K., Wiley,M.R., Hottel,W. and Cross,S.T.
EPI_ISL_17165685, EPI_ISL_17165694, EPI_ISL_17165703, EPI_ISL_17165710, EPI_ISL_17165721, EPI_ISL_17165732, EPI_ISL_17165734, EPI_ISL_17165736, EPI_ISL_17165739, EPI_ISL_17165740, EPI_ISL_17165742, EPI_ISL_17165748, EPI_ISL_17165752, EPI_ISL_17165758, EPI_ISL_17165766, EPI_ISL_17165776			
see above	Public Health Laboratory, NYC Department of Health and Mental Hygiene (DOHMH)	Public Health Laboratory, NYC Department of Health and Mental Hygiene (DOHMH)	Clabby,T.T., Amin,H.S., Wang,J.C., Taki,F., Su,M., Rahat,A., De La Cruz,N., Olsen,A., Thi,C., Silver,S., Akther,S., Chowdhury,M., Omoregie,E. and Hughes,S.
EPI_ISL_17170656, EPI_ISL_17170658, EPI_ISL_17170659, EPI_ISL_17170661, EPI_ISL_17170662, EPI_ISL_17170663, EPI_ISL_17170664, EPI_ISL_17170665, EPI_ISL_17170666, EPI_ISL_17170667, EPI_ISL_17170668, EPI_ISL_17170669, EPI_ISL_17170670			
see above	California Department of Public Health	California Department of Public Health	Haw, M. , Kath, C., Espinosa, A., O'Neil, R., and Hacker, J.
EPI_ISL_17179627, EPI_ISL_17179628	St Jame's Hospital, Virology Department	National Virus Reference Laboratory	Patrice Keane, Yvonne Lynagh, Brendan Crowley, Gabriel Gonzalez, Michael Carr, Emer O'Byrne, Weronika Banka, Brian Keogan, Jose Maria Urtasun Elizari, Jonathan Dean, Daniel Hare, Cillian F De Gascun
EPI_ISL_17179629, EPI_ISL_17179630, EPI_ISL_17179631, EPI_ISL_17179632	National Virus Reference Laboratory	National Virus Reference Laboratory	Gabriel Gonzalez, Michael Carr, Emer O'Byrne, Weronika Banka, Brian Keogan, Jose Maria Urtasun Elizari, Jonathan Dean, Daniel Hare, Cillian F De Gascun
EPI_ISL_17179633	St Jame's Hospital, Virology Department	National Virus Reference Laboratory	Patrice Keane, Yvonne Lynagh, Brendan Crowley, Gabriel Gonzalez, Michael Carr, Emer O'Byrne, Weronika Banka, Brian Keogan, Jose Maria Urtasun Elizari, Jonathan Dean, Daniel Hare, Cillian F De Gascun
EPI_ISL_17179634, EPI_ISL_17179635, EPI_ISL_17179636, EPI_ISL_17179637	National Virus Reference Laboratory	National Virus Reference Laboratory	Gabriel Gonzalez, Michael Carr, Emer O'Byrne, Weronika Banka, Brian Keogan, Jose Maria Urtasun Elizari, Jonathan Dean, Daniel Hare, Cillian F De Gascun
EPI_ISL_17179638, EPI_ISL_17179639, EPI_ISL_17179640, EPI_ISL_17179641, EPI_ISL_17179642, EPI_ISL_17179643	St Jame's Hospital, Virology Department	National Virus Reference Laboratory	Patrice Keane, Yvonne Lynagh, Brendan Crowley, Gabriel Gonzalez, Michael Carr, Emer O'Byrne, Weronika Banka, Brian Keogan, Jose Maria Urtasun Elizari, Jonathan Dean, Daniel Hare, Cillian F De Gascun
EPI_ISL_17187497, EPI_ISL_17187498	Vajira Hospital	Thai Red Cross Emerging Infectious Diseases Clinical Center and Faculty of Medicine, Chulalongkorn University	Suppasit srisaeng, Praepoly Ruekmuang, Kusuma Swangpun, Arriya Panchaiyaphum, Pakita Salaeh, Natpusda Kongmaung, Pornsiri Limwattanawong, Noree Pholprasert, Montriya Unteamsom, Kanjana Jeknok, Withak Withaksabut, Sunisa Nilda, Artorn Niakul, Sopon Iamsirithaworn, Thitipong Yingyong, Rossaporn Kittiyaowamarn, Rome Buathong, Ratanaporn Tangwangvivat, Supaporn Wacharapluesadee, Sininat Petcharat, Ananporn Supataragul, Stefan Fernandez, Achawin Rojanaviwat, Chonticha Klungthong, Pilailuk Okada, Khajohn Joonlasak, Chakkarat Pitayawonganon, Opass Putcharoen
EPI_ISL_17187499	Department of Disease Control, Ministry of Public Health	Thai Red Cross Emerging Infectious Diseases Clinical Center and Faculty of Medicine, Chulalongkorn University	Suppasit srisaeng, Praepoly Ruekmuang, Kusuma Swangpun, Arriya Panchaiyaphum, Pakita Salaeh, Natpusda Kongmaung, Pornsiri Limwattanawong, Noree Pholprasert, Montriya Unteamsom, Kanjana Jeknok, Withak Withaksabut, Sunisa Nilda, Artorn Niakul, Sopon Iamsirithaworn, Thitipong Yingyong, Rossaporn Kittiyaowamarn, Rome Buathong, Ratanaporn Tangwangvivat, Supaporn Wacharapluesadee, Sininat Petcharat, Ananporn Supataragul, Stefan Fernandez, Achawin Rojanaviwat, Chonticha Klungthong, Pilailuk Okada, Khajohn Joonlasak, Chakkarat Pitayawonganon, Opass Putcharoen
EPI_ISL_17187500, EPI_ISL_17187501	Bangkok Hospital Phuket	Thai Red Cross Emerging Infectious Diseases Clinical Center and Faculty of Medicine, Chulalongkorn University	Nungrathai Srisong, Praepoly Ruekmuang, Kusuma Swangpun, Arriya Panchaiyaphum, Pakita Salaeh, Natpusda Kongmaung, Pornsiri Limwattanawong, Noree Pholprasert, Montriya Unteamsom, Kanjana Jeknok, Withak Withaksabut, Sunisa Nilda, Artorn Niakul, Sopon Iamsirithaworn, Thitipong Yingyong, Rossaporn Kittiyaowamarn, Rome Buathong, Ratanaporn Tangwangvivat, Supaporn Wacharapluesadee, Sininat Petcharat, Ananporn Supataragul, Stefan Fernandez, Achawin Rojanaviwat, Chonticha Klungthong, Pilailuk Okada, Khajohn Joonlasak, Chakkarat Pitayawonganon, Opass Putcharoen
EPI_ISL_17187502	Department of Disease Control, Ministry of Public Health	Thai Red Cross Emerging Infectious Diseases Clinical Center and Faculty of Medicine, Chulalongkorn University	Supanut Chotichavalrattanakul, , Praepoly Ruekmuang, Kusuma Swangpun, Arriya Panchaiyaphum, Pakita Salaeh, Natpusda Kongmaung, Pornsiri Limwattanawong, Noree Pholprasert, Montriya Unteamsom, Kanjana Jeknok, Withak Withaksabut, Sunisa Nilda, Artorn Niakul, Sopon Iamsirithaworn, Thitipong Yingyong, Rossaporn Kittiyaowamarn, Rome Buathong, Ratanaporn Tangwangvivat, Supaporn Wacharapluesadee, Sininat Petcharat, Ananporn Supataragul, Stefan Fernandez, Achawin Rojanaviwat, Chonticha Klungthong, Pilailuk Okada, Khajohn Joonlasak, Chakkarat Pitayawonganon, Opass Putcharoen
EPI_ISL_17187503, EPI_ISL_17187504	Suvarnabhumi Airport	Thai Red Cross Emerging Infectious Diseases Clinical Center and Faculty of Medicine, Chulalongkorn University	Phawinee Montri, Praepoly Ruekmuang, Kusuma Swangpun, Arriya Panchaiyaphum, Pakita Salaeh, Natpusda Kongmaung, Pornsiri Limwattanawong, Noree Pholprasert, Montriya Unteamsom, Kanjana Jeknok, Withak Withaksabut, Sunisa Nilda, Artorn Niakul, Sopon Iamsirithaworn, Thitipong Yingyong, Rossaporn Kittiyaowamarn, Rome Buathong, Ratanaporn Tangwangvivat, Supaporn Wacharapluesadee, Sininat Petcharat, Ananporn Supataragul, Stefan Fernandez, Achawin Rojanaviwat, Chonticha Klungthong, Pilailuk Okada, Khajohn Joonlasak, Chakkarat Pitayawonganon, Opass Putcharoen
EPI_ISL_17187505	Phuket Provincial Public Health Office	Thai Red Cross Emerging Infectious Diseases Clinical Center and Faculty of Medicine, Chulalongkorn University	Nungrathai Srisong, Praepoly Ruekmuang, Kusuma Swangpun, Arriya Panchaiyaphum, Pakita Salaeh, Natpusda Kongmaung, Pornsiri Limwattanawong, Noree Pholprasert, Montriya Unteamsom, Kanjana Jeknok, Withak Withaksabut, Sunisa Nilda, Artorn Niakul, Sopon Iamsirithaworn, Thitipong Yingyong, Rossaporn Kittiyaowamarn, Rome Buathong, Ratanaporn Tangwangvivat, Supaporn Wacharapluesadee, Sininat Petcharat, Ananporn Supataragul, Stefan Fernandez, Achawin Rojanaviwat, Chonticha Klungthong, Pilailuk Okada, Khajohn Joonlasak, Chakkarat Pitayawonganon, Opass Putcharoen
EPI_ISL_17201439, EPI_ISL_17201440,	Genomics Division, Instituto Tecnologico y de Energias	Genomics Division, Instituto Tecnologico y de Energias	Munoz-Barrera,A., Ciuffreda,L., Alcoba-Florez,J., Rubio-Rodriguez,L.A., Rodriguez-Perez,H., Gil-Campesino,H., Garcia-Martinez de Artola,D., Salas-Hernandez,J., Rodriguez-Nunez,J., Inigo-Campos,A., Garcia-Olivares,V., Diez-Gil,O.,

EPI_ISL_17201441	Renovables (ITER)	Renovables (ITER)	Gonzalez-Montelongo,R., Valenzuela-Fernandez,A., Lorenzo-Salazar,J.M. and Flores,C.
EPI_ISL_17206607, EPI_ISL_17206608, EPI_ISL_17206609, EPI_ISL_17206610, EPI_ISL_17206612, EPI_ISL_17206613, EPI_ISL_17206614, EPI_ISL_17206615, EPI_ISL_17206616, EPI_ISL_17206618, EPI_ISL_17206619, EPI_ISL_17206620			
see above	California Department of Public Health	California Department of Public Health	Haw,M., Kath,C., Espinosa,A., O'Neil,R., and Hacker,J.
EPI_ISL_17206621, EPI_ISL_17206622	California Department of Public Health	California Department of Public Health	Kath, C., Haw, M., Espinosa, A., and Hacker, J.
EPI_ISL_17211324, EPI_ISL_17211325, EPI_ISL_17211326, EPI_ISL_17211327, EPI_ISL_17211328, EPI_ISL_17211329	Kaiser Permanente Chino Hills Regional Reference Laboratories	Los Angeles County Public Health Laboratories	P. Hemarajata et al.
EPI_ISL_17211331	Los Angeles County Public Health Laboratories	Los Angeles County Public Health Laboratories	P. Hemarajata et al.
EPI_ISL_17211332	Quest Diagnostics Nichols Institute	Los Angeles County Public Health Laboratories	P. Hemarajata et al.
EPI_ISL_17211333	Los Angeles County Public Health Laboratories	Los Angeles County Public Health Laboratories	P. Hemarajata et al.
EPI_ISL_17222811, EPI_ISL_17222813, EPI_ISL_17222814, EPI_ISL_17222817, EPI_ISL_17222818, EPI_ISL_17222819, EPI_ISL_17222820, EPI_ISL_17222822, EPI_ISL_17222823, EPI_ISL_17222824, EPI_ISL_17222825, EPI_ISL_17222827, EPI_ISL_17222828			
see above	Viral and Rickettsial Disease Laboratory (VRDL) California Department of Public Health (CDPH)	Viral and Rickettsial Disease Laboratory (VRDL) California Department of Public Health (CDPH)	Haw,M., Kath,C., Espinosa,A., O'Neil,R. and Hacker,J.
EPI_ISL_17246657, EPI_ISL_17246659	Fumi Kasuya Tokyo Metropolitan Institute of Public Health, Department of Microbiology	Fumi Kasuya Tokyo Metropolitan Institute of Public Health, Department of Microbiology	Kasuya,F., Negishi,A., Kumagai,R., Hasegawa,M., Fujiwara,T., Miyake,H., Nagashima,M. and Sadamasu,K.
EPI_ISL_17269833, EPI_ISL_17269834, EPI_ISL_17269835, EPI_ISL_17269836, EPI_ISL_17269837, EPI_ISL_17269838, EPI_ISL_17269839	Environmental, Agricultural, and Occupational Health, University of Nebraska Medical Center	Environmental, Agricultural, and Occupational Health, University of Nebraska Medical Center	Tegomoh,B., Cross,S.T., Chapman,R.C., Bernhard,K., McCutchen,E.L., Fauver,J.R., Pratt,C.B., Warden,D.E., Iwen,P.C., Donahue,M. and Wiley,M.R.
EPI_ISL_17319547, EPI_ISL_17319548, EPI_ISL_17319550, EPI_ISL_17319551	Department of Clinical Sciences, Institute of Tropical Medicine	Department of Clinical Sciences, Institute of Tropical Medicine	Mertes,H., Rezende,A.M., Naesens,R., de Block,T., Michiels,J., Coppens,J., Van Dijck,C., Bomans,P., Arien,K., Bottieau,E., Van Esbroeck,M., Liesenborghs,L. and Vercauteren,K.
EPI_ISL_17383630, EPI_ISL_17383632, EPI_ISL_17383634, EPI_ISL_17383635, EPI_ISL_17383636, EPI_ISL_17383637, EPI_ISL_17383639, EPI_ISL_17383641	Laboratorio Departamental de Salud Publica de Antioquia	Laboratorio Departamental de Salud Publica de Antioquia	Betancur,I.I.B., Velarde Hoyos,C.A.C.V., Gomez,R.R.G. and Mercado-Reyes,M.M.R.
EPI_ISL_17406093, EPI_ISL_17406094, EPI_ISL_17406095, EPI_ISL_17406096, EPI_ISL_17406097, EPI_ISL_17406098, EPI_ISL_17406099, EPI_ISL_17406100, EPI_ISL_17406101, EPI_ISL_17406102, EPI_ISL_17406103, EPI_ISL_17406104, EPI_ISL_17406105, EPI_ISL_17406106, EPI_ISL_17406107, EPI_ISL_17406108, EPI_ISL_17406109, EPI_ISL_17406110, EPI_ISL_17406111, EPI_ISL_17406112, EPI_ISL_17406113, EPI_ISL_17406115, EPI_ISL_17406116, EPI_ISL_17406117, EPI_ISL_17406118, EPI_ISL_17406119, EPI_ISL_17406120, EPI_ISL_17406121, EPI_ISL_17406122, EPI_ISL_17406123, EPI_ISL_17406124			
see above	CDCT/CEVS/SES-RS	CDCT/CEVS/SES-RS	Richard Steiner Salvato, Fernanda Marques Godinho, Regina Bones Barcellos, Patricia Sesterheim, Amanda Pellenz Ruivo, Viviane Horn de Melo, Júlio Augusto Schroder
EPI_ISL_17424657, EPI_ISL_17424658, EPI_ISL_17424659, EPI_ISL_17424660, EPI_ISL_17424661, EPI_ISL_17424662, EPI_ISL_17424663, EPI_ISL_17424664, EPI_ISL_17424665, EPI_ISL_17424666, EPI_ISL_17424667, EPI_ISL_17424668, EPI_ISL_17424669, EPI_ISL_17424670, EPI_ISL_17424671, EPI_ISL_17424673, EPI_ISL_17424674, EPI_ISL_17424675, EPI_ISL_17424676, EPI_ISL_17424677, EPI_ISL_17424678			
see above	Molecular Microbiology Laboratory, Department of Pathology, Molecular and Cell-Based Medicine, Icahn School of Medicine at Mount Sinai,	Molecular Microbiology Laboratory, Department of Pathology, Molecular and Cell-Based Medicine, Icahn School of Medicine at Mount Sinai,	Luz H. Patiño, Susana Guerra, Marina Muñoz, Nicolas Luna , Keith Farrugia, Adriana van de Guchte, Zain Khalil , Ana Silvia Gonzalez-Reiche, Matthew M. Hernandez ,Radhika Banu, Paras Shrestha, Bernadette Liggayu, Adolfo Firpo Betancourt, David Reich, Carlos Cordon-Cardo, Randy Albrechtf, Rebecca Pearlf, Viviana Simona, Aria Rookera, Emilia Mia Sordillo, Harm van Bakeld, Adolfo García-Sastre, Dusan Bogunovic, Gustavo Palacios, Alberto Paniz Mondolfi, Juan David Ramírez
EPI_ISL_17445514, EPI_ISL_17445515, EPI_ISL_17445516, EPI_ISL_17445517, EPI_ISL_17445518, EPI_ISL_17445519	Tokyo Metropolitan Institute of Public Health	Tokyo Metropolitan Institute of Public Health	Fumi Kasuya, Wakaba Okada, Ryota Kumagai, Sachiko Harada, Arisa Amano, Michiya Hasegawa, Mami Nagashima, Kenji Sadamasu
EPI_ISL_17471100, EPI_ISL_17471101, EPI_ISL_17471102, EPI_ISL_17471103, EPI_ISL_17471104, EPI_ISL_17471106, EPI_ISL_17471108, EPI_ISL_17471110	Laboratorio de Enterovirus, Instituto Oswaldo Cruz, Fiocruz	Laboratory of Respiratory Viruses and Measles, Oswaldo Cruz Institute, FIOCRUZ	Paola Resende, Elisa Cavalcante Pereira, Bruna Mendonça da Silva, Jéssica Graça Macedo de Carvalho, Larissa Macedo Pinto, Victor Guimaraes, Marilda Siqueira, Renan da Silva Faustino, Marilia Santini, Beatriz Grinsztejn, Mayara Secco Torres da Silva, Edson Elias da Silva on behalf of the Fiocruz Genomic Surveillance Network
EPI_ISL_17485343	Laboratorio de Enterovirus, Instituto Oswaldo Cruz, Fiocruz	Instituto Oswaldo Cruz FIOCRUZ - Laboratory of Respiratory Viruses and Measles (LVRS)	Paola Resende, Elisa Cavalcante Pereira, Bruna Mendonça da Silva, Jéssica Graça Macedo de Carvalho, Larissa Macedo Pinto, Victor Guimaraes, Marilda Siqueira, Renan da Silva Faustino, Marilia Santini, Beatriz Grinsztejn, Mayara Secco Torres da Silva, Edson Elias da Silva on behalf of the Fiocruz Genomic Surveillance Network
EPI_ISL_17502583	Public Health Laboratory, Public Health Service Amsterdam, The Netherlands	Department of Medical Microbiology & Infection prevention, Amsterdam University Medical Centers location AMC	Matthijs Welkers, Jelle Koopsen, Robin van Houdt, Marcel Jonges, Sebastien Matamoros, Sjoerd Rebers, Fokla Zorgdrager, Sylvia Bruisten, Akke Cornelissen, Janke Schinkel, Ewout Fanoy, Roisin Bavalia, Menno de Jong and Mariken van der Lubben on behalf of the Amsterdam Regional Genomic epidemiology and Outbreak Surveillance (ARGOS) consortium
EPI_ISL_17518107	Virology Section, Division of Microbiology,Osaka Institute of Public Health	Virology Section, Division of Microbiology,Osaka Institute of Public Health	Daiki Kanbayashi, Takako Kurata, Takuya Kawahata, Fumiya Bannno, Minami Hama, Kazushi Motomuta
EPI_ISL_17525484	Division de Microbiologia, Hospital Nacional de Niños Carlos Saenz Herrera	Inciensa, Investigación y Enseñanza en Nutrición y Salud Centro Nacional de Referencia de Virología	Cristian Perez Corrales, Christopher Mairena Acuña, Diana Cantillo, Hillary Serrano, Ana Isela Ruiz, Gustavo Vega, Claudio Soto-Garita, Adriana Godinez, Estela Cordero, Melany Calderon, Francisco Duarte
EPI_ISL_17529368	Laboratorio de Virus Exantematicos, Gastroentéricos y Otros Transmitidos por Vectores	Centro de Referencia Nacional de Genomica, Secuenciacion y Bioinformatica GENSBIO, INSPI-CZ9	Andrés Carrazco*, SIlvia Salgado, Diana Gutiérrez, Damaris Alarcón, Andrés Tinizaray, Ruth Gómez, Martha Sánchez, Johanna Parrales, Eva Nicola, Jorge Bejarano, Leandro Patiño.
EPI_ISL_17536780	Department of Virology, National Institute of Health, Islamabad, Pakistan	Department of Virology, National Institute of Health, Islamabad, Pakistan	Massab Umair, Muhammad Ammar, Syed Adnan Haider, Rabia Hakim, Qasim Malik, Muhammad Salman, Ghazala Parveen, and Naseem Akhtar
EPI_ISL_17536782, EPI_ISL_17536783, EPI_ISL_17536784, EPI_ISL_17536785	Laboratorio de Enterovirus, Instituto Oswaldo Cruz, Fiocruz	Instituto Oswaldo Cruz FIOCRUZ - Laboratory of Respiratory Viruses and Measles (LVRS)	Paola Resende, Elisa Cavalcante Pereira, Bruna Mendonça da Silva, Jéssica Graça Macedo de Carvalho, Larissa Macedo Pinto, Victor Guimaraes, Marilda Siqueira, Renan da Silva Faustino, Marilia Santini, Edson Elias da Silva on behalf of the Fiocruz Genomic Surveillance Network
EPI_ISL_17584292	Centro Medico ABC	Instituto Nacional de Medicina Genomica	Cedro Tanda Alberto, Roxana Trejo González, Laura Gomez-Romero, Alfredo Mendoza-Vargas, Dora Garnica-Lopez, Alfredo Hidalgo-Miranda, Luis A Herrera.
EPI_ISL_17592665, EPI_ISL_17592666, EPI_ISL_17592667, EPI_ISL_17592668, EPI_ISL_17592669, EPI_ISL_17592670	Tokyo Metropolitan Institute of Public Health	Tokyo Metropolitan Institute of Public Health	Fumi Kasuya, Wakaba Okada, Ryota Kumagai, Sachiko Harada, Arisa Amano, Michiya Hasegawa, Mami Nagashima, Kenji Sadamasu
EPI_ISL_17614017, EPI_ISL_17614018, EPI_ISL_17614019, EPI_ISL_17614020, EPI_ISL_17614021, EPI_ISL_17614022, EPI_ISL_17614024, EPI_ISL_17614025, EPI_ISL_17614026, EPI_ISL_17614027, EPI_ISL_17614028, EPI_ISL_17614029, EPI_ISL_17614030, EPI_ISL_17614031, EPI_ISL_17614032, EPI_ISL_17614033, EPI_ISL_17614034, EPI_ISL_17614035, EPI_ISL_17614036, EPI_ISL_17614037, EPI_ISL_17614039, EPI_ISL_17614040, EPI_ISL_17614041, EPI_ISL_17614042, EPI_ISL_17614043, EPI_ISL_17614044, EPI_ISL_17614045, EPI_ISL_17614046, EPI_ISL_17614047, EPI_ISL_17614048, EPI_ISL_17614049			
see above	Laboratorio de Enterovirus, Instituto Oswaldo Cruz, Fiocruz	Instituto Oswaldo Cruz FIOCRUZ - Laboratory of Respiratory Viruses and Measles (LVRS)	Paola Resende, Elisa Cavalcante Pereira, Bruna Mendonça da Silva, Jéssica Graça Macedo de Carvalho, Larissa Macedo Pinto, Victor Guimaraes, Marilda Siqueira, Renan da Silva Faustino, Marilia Santini, Edson Elias da Silva on behalf of the Fiocruz Genomic Surveillance Network
EPI_ISL_17665624, EPI_ISL_17665625, EPI_ISL_17665626, EPI_ISL_17665627	Tokyo Metropolitan Institute of Public Health	Tokyo Metropolitan Institute of Public Health	Fumi Kasuya, Wakaba Okada, Ryota Kumagai, Sachiko Harada, Arisa Amano, Michiya Hasegawa, Mami Nagashima, Kenji Sadamasu
EPI_ISL_17672206	LESP State of Mexico	Instituto de Diagnostico y Referencia Epidemiologicos (INDRE)	Abril Rodríguez-Maldonado; Claudia Wong-Arámbula; Silvia Rivero-Arredondo; Ruth Madera-Sandoval; Joaquín Quiroz-Mercado; Fernando González-Domínguez; Lucia Hernández-Rivas, Irma López-Martínez; Ernesto Ramírez-González; Maribel González-Villa
EPI_ISL_17672207	LESP Jalisco	Instituto de Diagnostico y Referencia Epidemiologicos (INDRE)	Abril Rodríguez-Maldonado; Claudia Wong-Arámbula; Silvia Rivero-Arredondo; Ruth Madera-Sandoval; Joaquín Quiroz-Mercado; Fernando González-Domínguez; Lucia Hernández-Rivas, Irma López-Martínez; Ernesto Ramírez-González; Maribel González-Villa
EPI_ISL_17672208	LESP Queretaro	Instituto de Diagnostico y Referencia Epidemiologicos (INDRE)	Abril Rodríguez-Maldonado; Claudia Wong-Arámbula; Silvia Rivero-Arredondo; Ruth Madera-Sandoval; Joaquín Quiroz-Mercado; Fernando González-Domínguez; Lucia Hernández-Rivas, Irma López-Martínez; Ernesto Ramírez-González; Maribel González-Villa
EPI_ISL_17672209	LESP Yucatan	Instituto de Diagnostico y Referencia Epidemiologicos (INDRE)	Abril Rodríguez-Maldonado; Claudia Wong-Arámbula; Silvia Rivero-Arredondo; Ruth Madera-Sandoval; Joaquín Quiroz-Mercado; Fernando González-Domínguez; Lucia Hernández-Rivas, Irma López-Martínez; Ernesto Ramírez-González; Maribel González-Villa
EPI_ISL_17672210	LESP Quintana Roo	Instituto de Diagnostico y Referencia Epidemiologicos (INDRE)	Abril Rodríguez-Maldonado; Claudia Wong-Arámbula; Silvia Rivero-Arredondo; Ruth Madera-Sandoval; Joaquín Quiroz-Mercado; Fernando González-Domínguez; Lucia Hernández-Rivas, Irma López-Martínez; Ernesto Ramírez-González; Maribel González-Villa
EPI_ISL_17672211	LESP Mexico City	Instituto de Diagnostico y Referencia Epidemiologicos (INDRE)	Abril Rodríguez-Maldonado; Claudia Wong-Arámbula; Silvia Rivero-Arredondo; Ruth Madera-Sandoval; Joaquín Quiroz-Mercado; Fernando González-Domínguez; Lucia Hernández-Rivas, Irma López-Martínez; Ernesto Ramírez-González; Maribel González-Villa
EPI_ISL_17672212	LESP Tamaulipas	Instituto de Diagnostico y Referencia Epidemiologicos (INDRE)	Abril Rodríguez-Maldonado; Claudia Wong-Arámbula; Silvia Rivero-Arredondo; Ruth Madera-Sandoval; Joaquín Quiroz-Mercado; Fernando González-Domínguez; Lucia Hernández-Rivas, Irma López-Martínez; Ernesto Ramírez-González; Maribel González-Villa
EPI_ISL_17672213	LESP Puebla	Instituto de Diagnostico y Referencia Epidemiologicos (INDRE)	Abril Rodríguez-Maldonado; Claudia Wong-Arámbula; Silvia Rivero-Arredondo; Ruth Madera-Sandoval; Joaquín Quiroz-Mercado; Fernando González-Domínguez; Lucia Hernández-Rivas, Irma López-Martínez; Ernesto Ramírez-González; Maribel González-Villa
EPI_ISL_17672214	LESP Guerrero	Instituto de Diagnostico y Referencia Epidemiologicos (INDRE)	Abril Rodríguez-Maldonado; Claudia Wong-Arámbula; Silvia Rivero-Arredondo; Ruth Madera-Sandoval; Joaquín Quiroz-Mercado; Fernando González-Domínguez; Lucia Hernández-Rivas, Irma López-Martínez; Ernesto Ramírez-González; Maribel González-Villa
EPI_ISL_17672215	LESP Oaxaca	Instituto de Diagnostico y Referencia Epidemiologicos (INDRE)	Abril Rodríguez-Maldonado; Claudia Wong-Arámbula; Silvia Rivero-Arredondo; Ruth Madera-Sandoval; Joaquín Quiroz-Mercado; Fernando González-Domínguez; Lucia Hernández-Rivas, Irma López-Martínez; Ernesto Ramírez-González; Maribel González-Villa
EPI_ISL_17672216	LESP Hidalgo	Instituto de Diagnostico y Referencia Epidemiologicos (INDRE)	Abril Rodríguez-Maldonado; Claudia Wong-Arámbula; Silvia Rivero-Arredondo; Ruth Madera-Sandoval; Joaquín Quiroz-Mercado; Fernando González-Domínguez; Lucia Hernández-Rivas, Irma López-Martínez; Ernesto Ramírez-González; Maribel González-Villa
EPI_ISL_17672217	LESP Zacatecas	Instituto de Diagnostico y Referencia Epidemiologicos (INDRE)	Abril Rodríguez-Maldonado; Claudia Wong-Arámbula; Silvia Rivero-Arredondo; Ruth Madera-Sandoval; Joaquín Quiroz-Mercado; Fernando González-Domínguez; Lucia Hernández-Rivas, Irma López-Martínez; Ernesto Ramírez-González; Maribel González-Villa
EPI_ISL_17672218	LESP Tabasco	Instituto de Diagnostico y Referencia Epidemiologicos	Abril Rodríguez-Maldonado; Claudia Wong-Arámbula; Silvia Rivero-Arredondo; Ruth Madera-Sandoval; Joaquín Quiroz-Mercado; Fernando González-Domínguez; Lucia Hernández-Rivas, Irma López-Martínez; Ernesto Ramírez-González;

		(INDRE)	Maribel González-Villa
EPI_ISL_17672219	LESP Aguascalientes	Instituto de Diagnostico y Referencia Epidemiologicos (INDRE)	Abril Rodríguez-Maldonado; Claudia Wong-Arámbula; Silvia Rivero-Arredondo; Ruth Madera-Sandoval; Joaquín Quiroz-Mercado; Fernando González-Domínguez; Lucia Hernández-Rivas, Irma López-Martínez; Ernesto Ramírez-González; Maribel González-Villa
EPI_ISL_17672220	LESP Tlaxcala	Instituto de Diagnostico y Referencia Epidemiologicos (INDRE)	Abril Rodríguez-Maldonado; Claudia Wong-Arámbula; Silvia Rivero-Arredondo; Ruth Madera-Sandoval; Joaquín Quiroz-Mercado; Fernando González-Domínguez; Lucia Hernández-Rivas, Irma López-Martínez; Ernesto Ramírez-González; Maribel González-Villa
EPI_ISL_17672221	LESP Chihuahua	Instituto de Diagnostico y Referencia Epidemiologicos (INDRE)	Abril Rodríguez-Maldonado; Claudia Wong-Arámbula; Silvia Rivero-Arredondo; Ruth Madera-Sandoval; Joaquín Quiroz-Mercado; Fernando González-Domínguez; Lucia Hernández-Rivas, Irma López-Martínez; Ernesto Ramírez-González; Maribel González-Villa
EPI_ISL_17672222	LESP Veracruz	Instituto de Diagnostico y Referencia Epidemiologicos (INDRE)	Abril Rodríguez-Maldonado; Claudia Wong-Arámbula; Silvia Rivero-Arredondo; Ruth Madera-Sandoval; Joaquín Quiroz-Mercado; Fernando González-Domínguez; Lucia Hernández-Rivas, Irma López-Martínez; Ernesto Ramírez-González; Maribel González-Villa
EPI_ISL_17672223	LESP Baja California	Instituto de Diagnostico y Referencia Epidemiologicos (INDRE)	Abril Rodríguez-Maldonado; Claudia Wong-Arámbula; Silvia Rivero-Arredondo; Ruth Madera-Sandoval; Joaquín Quiroz-Mercado; Fernando González-Domínguez; Lucia Hernández-Rivas, Irma López-Martínez; Ernesto Ramírez-González; Maribel González-Villa
EPI_ISL_17672224	LESP Tamaulipas	Instituto de Diagnostico y Referencia Epidemiologicos (INDRE)	Abril Rodríguez-Maldonado; Claudia Wong-Arámbula; Silvia Rivero-Arredondo; Ruth Madera-Sandoval; Joaquín Quiroz-Mercado; Fernando González-Domínguez; Lucia Hernández-Rivas, Irma López-Martínez; Ernesto Ramírez-González; Maribel González-Villa
EPI_ISL_17672225	LESP Campeche	Instituto de Diagnostico y Referencia Epidemiologicos (INDRE)	Abril Rodríguez-Maldonado; Claudia Wong-Arámbula; Silvia Rivero-Arredondo; Ruth Madera-Sandoval; Joaquín Quiroz-Mercado; Fernando González-Domínguez; Lucia Hernández-Rivas, Irma López-Martínez; Ernesto Ramírez-González; Maribel González-Villa
EPI_ISL_17672226	LESP Hidalgo	Instituto de Diagnostico y Referencia Epidemiologicos (INDRE)	Abril Rodríguez-Maldonado; Claudia Wong-Arámbula; Silvia Rivero-Arredondo; Ruth Madera-Sandoval; Joaquín Quiroz-Mercado; Fernando González-Domínguez; Lucia Hernández-Rivas, Irma López-Martínez; Ernesto Ramírez-González; Maribel González-Villa
EPI_ISL_17672227	LESP Mexico City	Instituto de Diagnostico y Referencia Epidemiologicos (INDRE)	Abril Rodríguez-Maldonado; Claudia Wong-Arámbula; Silvia Rivero-Arredondo; Ruth Madera-Sandoval; Joaquín Quiroz-Mercado; Fernando González-Domínguez; Lucia Hernández-Rivas, Irma López-Martínez; Ernesto Ramírez-González; Maribel González-Villa
EPI_ISL_17672228	LESP Guerrero	Instituto de Diagnostico y Referencia Epidemiologicos (INDRE)	Abril Rodríguez-Maldonado; Claudia Wong-Arámbula; Silvia Rivero-Arredondo; Ruth Madera-Sandoval; Joaquín Quiroz-Mercado; Fernando González-Domínguez; Lucia Hernández-Rivas, Irma López-Martínez; Ernesto Ramírez-González; Maribel González-Villa
EPI_ISL_17672229	LESP Coahuila	Instituto de Diagnostico y Referencia Epidemiologicos (INDRE)	Abril Rodríguez-Maldonado; Claudia Wong-Arámbula; Silvia Rivero-Arredondo; Ruth Madera-Sandoval; Joaquín Quiroz-Mercado; Fernando González-Domínguez; Lucia Hernández-Rivas, Irma López-Martínez; Ernesto Ramírez-González; Maribel González-Villa
EPI_ISL_17672230	LESP Tabasco	Instituto de Diagnostico y Referencia Epidemiologicos (INDRE)	Abril Rodríguez-Maldonado; Claudia Wong-Arámbula; Silvia Rivero-Arredondo; Ruth Madera-Sandoval; Joaquín Quiroz-Mercado; Fernando González-Domínguez; Lucia Hernández-Rivas, Irma López-Martínez; Ernesto Ramírez-González; Maribel González-Villa
EPI_ISL_17672231	LESP Chiapas	Instituto de Diagnostico y Referencia Epidemiologicos (INDRE)	Abril Rodríguez-Maldonado; Claudia Wong-Arámbula; Silvia Rivero-Arredondo; Ruth Madera-Sandoval; Joaquín Quiroz-Mercado; Fernando González-Domínguez; Lucia Hernández-Rivas, Irma López-Martínez; Ernesto Ramírez-González; Maribel González-Villa
EPI_ISL_17672232	LESP Yucatan	Instituto de Diagnostico y Referencia Epidemiologicos (INDRE)	Abril Rodríguez-Maldonado; Claudia Wong-Arámbula; Silvia Rivero-Arredondo; Ruth Madera-Sandoval; Joaquín Quiroz-Mercado; Fernando González-Domínguez; Lucia Hernández-Rivas, Irma López-Martínez; Ernesto Ramírez-González; Maribel González-Villa
EPI_ISL_17672233	LESP Quintana Roo	Instituto de Diagnostico y Referencia Epidemiologicos (INDRE)	Abril Rodríguez-Maldonado; Claudia Wong-Arámbula; Silvia Rivero-Arredondo; Ruth Madera-Sandoval; Joaquín Quiroz-Mercado; Fernando González-Domínguez; Lucia Hernández-Rivas, Irma López-Martínez; Ernesto Ramírez-González; Maribel González-Villa
EPI_ISL_17672234	LESP Jalisco	Instituto de Diagnostico y Referencia Epidemiologicos (INDRE)	Abril Rodríguez-Maldonado; Claudia Wong-Arámbula; Silvia Rivero-Arredondo; Ruth Madera-Sandoval; Joaquín Quiroz-Mercado; Fernando González-Domínguez; Lucia Hernández-Rivas, Irma López-Martínez; Ernesto Ramírez-González; Maribel González-Villa
EPI_ISL_17672235	LESP State of Mexico	Instituto de Diagnostico y Referencia Epidemiologicos (INDRE)	Abril Rodríguez-Maldonado; Claudia Wong-Arámbula; Silvia Rivero-Arredondo; Ruth Madera-Sandoval; Joaquín Quiroz-Mercado; Fernando González-Domínguez; Lucia Hernández-Rivas, Irma López-Martínez; Ernesto Ramírez-González; Maribel González-Villa
EPI_ISL_17672236	Laboratorio Estatal de Salud Pública Puebla	Instituto de Diagnostico y Referencia Epidemiologicos (INDRE)	Abril Rodríguez-Maldonado; Claudia Wong-Arámbula; Silvia Rivero-Arredondo; Ruth Madera-Sandoval; Joaquín Quiroz-Mercado; Fernando González-Domínguez; Lucia Hernández-Rivas, Irma López-Martínez; Ernesto Ramírez-González; Maribel González-Villa
EPI_ISL_17672237	LESP Campeche	Instituto de Diagnostico y Referencia Epidemiologicos (INDRE)	Abril Rodríguez-Maldonado; Claudia Wong-Arámbula; Silvia Rivero-Arredondo; Ruth Madera-Sandoval; Joaquín Quiroz-Mercado; Fernando González-Domínguez; Lucia Hernández-Rivas, Irma López-Martínez; Ernesto Ramírez-González; Maribel González-Villa
EPI_ISL_17672238	LESP State of Mexico	Instituto de Diagnostico y Referencia Epidemiologicos (INDRE)	Abril Rodríguez-Maldonado; Claudia Wong-Arámbula; Silvia Rivero-Arredondo; Ruth Madera-Sandoval; Joaquín Quiroz-Mercado; Fernando González-Domínguez; Lucia Hernández-Rivas, Irma López-Martínez; Ernesto Ramírez-González; Maribel González-Villa
EPI_ISL_17672239	LESP Chiapas	Instituto de Diagnostico y Referencia Epidemiologicos (INDRE)	Abril Rodríguez-Maldonado; Claudia Wong-Arámbula; Silvia Rivero-Arredondo; Ruth Madera-Sandoval; Joaquín Quiroz-Mercado; Fernando González-Domínguez; Lucia Hernández-Rivas, Irma López-Martínez; Ernesto Ramírez-González; Maribel González-Villa
EPI_ISL_17672240	LESP Coahuila	Instituto de Diagnostico y Referencia Epidemiologicos (INDRE)	Abril Rodríguez-Maldonado; Claudia Wong-Arámbula; Silvia Rivero-Arredondo; Ruth Madera-Sandoval; Joaquín Quiroz-Mercado; Fernando González-Domínguez; Lucia Hernández-Rivas, Irma López-Martínez; Ernesto Ramírez-González; Maribel González-Villa
EPI_ISL_17672241	LESP Jalisco	Instituto de Diagnostico y Referencia Epidemiologicos (INDRE)	Abril Rodríguez-Maldonado; Claudia Wong-Arámbula; Silvia Rivero-Arredondo; Ruth Madera-Sandoval; Joaquín Quiroz-Mercado; Fernando González-Domínguez; Lucia Hernández-Rivas, Irma López-Martínez; Ernesto Ramírez-González; Maribel González-Villa
EPI_ISL_17672242	LESP Nuevo Leon	Instituto de Diagnostico y Referencia Epidemiologicos (INDRE)	Abril Rodríguez-Maldonado; Claudia Wong-Arámbula; Silvia Rivero-Arredondo; Ruth Madera-Sandoval; Joaquín Quiroz-Mercado; Fernando González-Domínguez; Lucia Hernández-Rivas, Irma López-Martínez; Ernesto Ramírez-González; Maribel González-Villa
EPI_ISL_17672243	LESP Queretaro	Instituto de Diagnostico y Referencia Epidemiologicos (INDRE)	Abril Rodríguez-Maldonado; Claudia Wong-Arámbula; Silvia Rivero-Arredondo; Ruth Madera-Sandoval; Joaquín Quiroz-Mercado; Fernando González-Domínguez; Lucia Hernández-Rivas, Irma López-Martínez; Ernesto Ramírez-González; Maribel González-Villa
EPI_ISL_17672244	LESP Hidalgo	Instituto de Diagnostico y Referencia Epidemiologicos (INDRE)	Abril Rodríguez-Maldonado; Claudia Wong-Arámbula; Silvia Rivero-Arredondo; Ruth Madera-Sandoval; Joaquín Quiroz-Mercado; Fernando González-Domínguez; Lucia Hernández-Rivas, Irma López-Martínez; Ernesto Ramírez-González; Maribel González-Villa
EPI_ISL_17672245	LESP Tamaulipas	Instituto de Diagnostico y Referencia Epidemiologicos (INDRE)	Abril Rodríguez-Maldonado; Claudia Wong-Arámbula; Silvia Rivero-Arredondo; Ruth Madera-Sandoval; Joaquín Quiroz-Mercado; Fernando González-Domínguez; Lucia Hernández-Rivas, Irma López-Martínez; Ernesto Ramírez-González; Maribel González-Villa
EPI_ISL_17672246	LESP Veracruz	Instituto de Diagnostico y Referencia Epidemiologicos (INDRE)	Abril Rodríguez-Maldonado; Claudia Wong-Arámbula; Silvia Rivero-Arredondo; Ruth Madera-Sandoval; Joaquín Quiroz-Mercado; Fernando González-Domínguez; Lucia Hernández-Rivas, Irma López-Martínez; Ernesto Ramírez-González; Maribel González-Villa
EPI_ISL_17672247	LESP Quintana Roo	Instituto de Diagnostico y Referencia Epidemiologicos (INDRE)	Abril Rodríguez-Maldonado; Claudia Wong-Arámbula; Silvia Rivero-Arredondo; Ruth Madera-Sandoval; Joaquín Quiroz-Mercado; Fernando González-Domínguez; Lucia Hernández-Rivas, Irma López-Martínez; Ernesto Ramírez-González; Maribel González-Villa
EPI_ISL_17672248	LESP Queretaro	Instituto de Diagnostico y Referencia Epidemiologicos (INDRE)	Abril Rodríguez-Maldonado; Claudia Wong-Arámbula; Silvia Rivero-Arredondo; Ruth Madera-Sandoval; Joaquín Quiroz-Mercado; Fernando González-Domínguez; Lucia Hernández-Rivas, Irma López-Martínez; Ernesto Ramírez-González; Maribel González-Villa
EPI_ISL_17672249	LESP Oaxaca	Instituto de Diagnostico y Referencia Epidemiologicos (INDRE)	Abril Rodríguez-Maldonado; Claudia Wong-Arámbula; Silvia Rivero-Arredondo; Ruth Madera-Sandoval; Joaquín Quiroz-Mercado; Fernando González-Domínguez; Lucia Hernández-Rivas, Irma López-Martínez; Ernesto Ramírez-González; Maribel González-Villa
EPI_ISL_17672250	LESP Yucatan	Instituto de Diagnostico y Referencia Epidemiologicos (INDRE)	Abril Rodríguez-Maldonado; Claudia Wong-Arámbula; Silvia Rivero-Arredondo; Ruth Madera-Sandoval; Joaquín Quiroz-Mercado; Fernando González-Domínguez; Lucia Hernández-Rivas, Irma López-Martínez; Ernesto Ramírez-González; Maribel González-Villa
EPI_ISL_17672251	LESP Guanajuato	Instituto de Diagnostico y Referencia Epidemiologicos (INDRE)	Abril Rodríguez-Maldonado; Claudia Wong-Arámbula; Silvia Rivero-Arredondo; Ruth Madera-Sandoval; Joaquín Quiroz-Mercado; Fernando González-Domínguez; Lucia Hernández-Rivas, Irma López-Martínez; Ernesto Ramírez-González; Maribel González-Villa
EPI_ISL_17672252	LESP Veracruz	Instituto de Diagnostico y Referencia Epidemiologicos (INDRE)	Abril Rodríguez-Maldonado; Claudia Wong-Arámbula; Silvia Rivero-Arredondo; Ruth Madera-Sandoval; Joaquín Quiroz-Mercado; Fernando González-Domínguez; Lucia Hernández-Rivas, Irma López-Martínez; Ernesto Ramírez-González; Maribel González-Villa
EPI_ISL_17672253	LESP Mexico City	Instituto de Diagnostico y Referencia Epidemiologicos (INDRE)	Abril Rodríguez-Maldonado; Claudia Wong-Arámbula; Silvia Rivero-Arredondo; Ruth Madera-Sandoval; Joaquín Quiroz-Mercado; Fernando González-Domínguez; Lucia Hernández-Rivas, Irma López-Martínez; Ernesto Ramírez-González; Maribel González-Villa
EPI_ISL_17672254	LESP Durango	Instituto de Diagnostico y Referencia Epidemiologicos (INDRE)	Abril Rodríguez-Maldonado; Claudia Wong-Arámbula; Silvia Rivero-Arredondo; Ruth Madera-Sandoval; Joaquín Quiroz-Mercado; Fernando González-Domínguez; Lucia Hernández-Rivas, Irma López-Martínez; Ernesto Ramírez-González; Maribel González-Villa
EPI_ISL_17672255	LESP San Luis Potosi	Instituto de Diagnostico y Referencia Epidemiologicos (INDRE)	Abril Rodríguez-Maldonado; Claudia Wong-Arámbula; Silvia Rivero-Arredondo; Ruth Madera-Sandoval; Joaquín Quiroz-Mercado; Fernando González-Domínguez; Lucia Hernández-Rivas, Irma López-Martínez; Ernesto Ramírez-González; Maribel González-Villa
EPI_ISL_17672256	LESP Nuevo Leon	Instituto de Diagnostico y Referencia Epidemiologicos (INDRE)	Abril Rodríguez-Maldonado; Claudia Wong-Arámbula; Silvia Rivero-Arredondo; Ruth Madera-Sandoval; Joaquín Quiroz-Mercado; Fernando González-Domínguez; Lucia Hernández-Rivas, Irma López-Martínez; Ernesto Ramírez-González; Maribel González-Villa
EPI_ISL_17672257, EPI_ISL_17672258	LESP Quintana Roo	Instituto de Diagnostico y Referencia Epidemiologicos (INDRE)	Abril Rodríguez-Maldonado; Claudia Wong-Arámbula; Silvia Rivero-Arredondo; Ruth Madera-Sandoval; Joaquín Quiroz-Mercado; Fernando González-Domínguez; Lucia Hernández-Rivas, Irma López-Martínez; Ernesto Ramírez-González; Maribel González-Villa
EPI_ISL_17672259	LESP Mexico City	Instituto de Diagnostico y Referencia Epidemiologicos (INDRE)	Abril Rodríguez-Maldonado; Claudia Wong-Arámbula; Silvia Rivero-Arredondo; Ruth Madera-Sandoval; Joaquín Quiroz-Mercado; Fernando González-Domínguez; Lucia Hernández-Rivas, Irma López-Martínez; Ernesto Ramírez-González; Maribel González-Villa
EPI_ISL_17672260	LESP Veracruz	Instituto de Diagnostico y Referencia Epidemiologicos (INDRE)	Abril Rodríguez-Maldonado; Claudia Wong-Arámbula; Silvia Rivero-Arredondo; Ruth Madera-Sandoval; Joaquín Quiroz-Mercado; Fernando González-Domínguez; Lucia Hernández-Rivas, Irma López-Martínez; Ernesto Ramírez-González; Maribel González-Villa
EPI_ISL_17692268, EPI_ISL_17692269	Tokyo Metropolitan Institute of Public Health	Tokyo Metropolitan Institute of Public Health	Fumi Kasuya, Wakaba Okada, Ryota Kumagai, Sachiko Harada, Arisa Amano, Michiya Hasegawa, Mami Nagashima, Kenji Sadamasu
EPI_ISL_17703564, EPI_ISL_17703565	LESP State of Mexico	Instituto de Diagnostico y Referencia Epidemiologicos (INDRE)	Abril Rodríguez-Maldonado; Claudia Wong-Arámbula; Felipe Arguijo-Perez; Helios Cárdenas-Hernández; Carmen Castro-Méndez; Lidia García-Torres; Ruth Madera-Sandoval; América Mandujano-Martínez; Nancy Martínez-Velázquez; Mireya Mederos-Michel; Angélica Pedraza-Meléndez; Joaquín Quiroz-Mercado; Daniel Regalado-Santiago; Silvia Rivero-Arredondo; Erika Sierra-Atanacio; Fernando González-Domínguez; Lucia Hernández-Rivas, Irma López-Martínez; Ernesto Ramírez-González; Maribel González-Villa
EPI_ISL_17703566	LESP Jalisco	Instituto de Diagnostico y Referencia Epidemiologicos (INDRE)	Abril Rodríguez-Maldonado; Claudia Wong-Arámbula; Felipe Arguijo-Perez; Helios Cárdenas-Hernández; Carmen Castro-Méndez; Lidia García-Torres; Ruth Madera-Sandoval; América Mandujano-Martínez; Nancy Martínez-Velázquez; Mireya Mederos-Michel; Angélica Pedraza-Meléndez; Joaquín Quiroz-Mercado; Daniel Regalado-Santiago; Silvia Rivero-Arredondo; Erika Sierra-Atanacio; Fernando González-Domínguez; Lucia Hernández-Rivas, Irma López-Martínez; Ernesto Ramírez-González; Maribel González-Villa

EPI_ISL_17703567	LESP State of Mexico	Instituto de Diagnostico y Referencia Epidemiologicos (INDRE)	Abril Rodríguez-Maldonado; Claudia Wong-Arámbula; Felipe Arguijo-Perez; Helios Cárdenas-Hernández; Carmen Castro-Méndez; Lidia García-Torres; Ruth Madera-Sandoval; América Mandujano-Martínez; Nancy Martínez-Velázquez; Mireya Mederos-Michel; Angélica Pedraza-Meléndez; Joaquín Quiroz-Mercado; Daniel Regalado-Santiago; Silvia Rivero-Arredondo; Erika Sierra-Atanacio; Fernando González-Domínguez; Lucia Hernández-Rivas, Irma López-Martínez; Ernesto Ramírez-González; Maribel González-Villa
EPI_ISL_17703568	LESP Quintana Roo	Instituto de Diagnostico y Referencia Epidemiologicos (INDRE)	Abril Rodríguez-Maldonado; Claudia Wong-Arámbula; Felipe Arguijo-Perez; Helios Cárdenas-Hernández; Carmen Castro-Méndez; Lidia García-Torres; Ruth Madera-Sandoval; América Mandujano-Martínez; Nancy Martínez-Velázquez; Mireya Mederos-Michel; Angélica Pedraza-Meléndez; Joaquín Quiroz-Mercado; Daniel Regalado-Santiago; Silvia Rivero-Arredondo; Erika Sierra-Atanacio; Fernando González-Domínguez; Lucia Hernández-Rivas, Irma López-Martínez; Ernesto Ramírez-González; Maribel González-Villa
EPI_ISL_17703569, EPI_ISL_17703570	LESP Mexico City	Instituto de Diagnostico y Referencia Epidemiologicos (INDRE)	Abril Rodríguez-Maldonado; Claudia Wong-Arámbula; Felipe Arguijo-Perez; Helios Cárdenas-Hernández; Carmen Castro-Méndez; Lidia García-Torres; Ruth Madera-Sandoval; América Mandujano-Martínez; Nancy Martínez-Velázquez; Mireya Mederos-Michel; Angélica Pedraza-Meléndez; Joaquín Quiroz-Mercado; Daniel Regalado-Santiago; Silvia Rivero-Arredondo; Erika Sierra-Atanacio; Fernando González-Domínguez; Lucia Hernández-Rivas, Irma López-Martínez; Ernesto Ramírez-González; Maribel González-Villa
EPI_ISL_17703571	LESP Sinaloa	Instituto de Diagnostico y Referencia Epidemiologicos (INDRE)	Abril Rodríguez-Maldonado; Claudia Wong-Arámbula; Felipe Arguijo-Perez; Helios Cárdenas-Hernández; Carmen Castro-Méndez; Lidia García-Torres; Ruth Madera-Sandoval; América Mandujano-Martínez; Nancy Martínez-Velázquez; Mireya Mederos-Michel; Angélica Pedraza-Meléndez; Joaquín Quiroz-Mercado; Daniel Regalado-Santiago; Silvia Rivero-Arredondo; Erika Sierra-Atanacio; Fernando González-Domínguez; Lucia Hernández-Rivas, Irma López-Martínez; Ernesto Ramírez-González; Maribel González-Villa
EPI_ISL_17703572	LESP State of Mexico	Instituto de Diagnostico y Referencia Epidemiologicos (INDRE)	Abril Rodríguez-Maldonado; Claudia Wong-Arámbula; Felipe Arguijo-Perez; Helios Cárdenas-Hernández; Carmen Castro-Méndez; Lidia García-Torres; Ruth Madera-Sandoval; América Mandujano-Martínez; Nancy Martínez-Velázquez; Mireya Mederos-Michel; Angélica Pedraza-Meléndez; Joaquín Quiroz-Mercado; Daniel Regalado-Santiago; Silvia Rivero-Arredondo; Erika Sierra-Atanacio; Fernando González-Domínguez; Lucia Hernández-Rivas, Irma López-Martínez; Ernesto Ramírez-González; Maribel González-Villa
EPI_ISL_17703573	LESP Mexico City	Instituto de Diagnostico y Referencia Epidemiologicos (INDRE)	Abril Rodríguez-Maldonado; Claudia Wong-Arámbula; Felipe Arguijo-Perez; Helios Cárdenas-Hernández; Carmen Castro-Méndez; Lidia García-Torres; Ruth Madera-Sandoval; América Mandujano-Martínez; Nancy Martínez-Velázquez; Mireya Mederos-Michel; Angélica Pedraza-Meléndez; Joaquín Quiroz-Mercado; Daniel Regalado-Santiago; Silvia Rivero-Arredondo; Erika Sierra-Atanacio; Fernando González-Domínguez; Lucia Hernández-Rivas, Irma López-Martínez; Ernesto Ramírez-González; Maribel González-Villa
EPI_ISL_17703574	LESP State of Mexico	Instituto de Diagnostico y Referencia Epidemiologicos (INDRE)	Abril Rodríguez-Maldonado; Claudia Wong-Arámbula; Felipe Arguijo-Perez; Helios Cárdenas-Hernández; Carmen Castro-Méndez; Lidia García-Torres; Ruth Madera-Sandoval; América Mandujano-Martínez; Nancy Martínez-Velázquez; Mireya Mederos-Michel; Angélica Pedraza-Meléndez; Joaquín Quiroz-Mercado; Daniel Regalado-Santiago; Silvia Rivero-Arredondo; Erika Sierra-Atanacio; Fernando González-Domínguez; Lucia Hernández-Rivas, Irma López-Martínez; Ernesto Ramírez-González; Maribel González-Villa
EPI_ISL_17703575	LESP Mexico City	Instituto de Diagnostico y Referencia Epidemiologicos (INDRE)	Abril Rodríguez-Maldonado; Claudia Wong-Arámbula; Felipe Arguijo-Perez; Helios Cárdenas-Hernández; Carmen Castro-Méndez; Lidia García-Torres; Ruth Madera-Sandoval; América Mandujano-Martínez; Nancy Martínez-Velázquez; Mireya Mederos-Michel; Angélica Pedraza-Meléndez; Joaquín Quiroz-Mercado; Daniel Regalado-Santiago; Silvia Rivero-Arredondo; Erika Sierra-Atanacio; Fernando González-Domínguez; Lucia Hernández-Rivas, Irma López-Martínez; Ernesto Ramírez-González; Maribel González-Villa
EPI_ISL_17703576	LESP Quintana Roo	Instituto de Diagnostico y Referencia Epidemiologicos (INDRE)	Abril Rodríguez-Maldonado; Claudia Wong-Arámbula; Felipe Arguijo-Perez; Helios Cárdenas-Hernández; Carmen Castro-Méndez; Lidia García-Torres; Ruth Madera-Sandoval; América Mandujano-Martínez; Nancy Martínez-Velázquez; Mireya Mederos-Michel; Angélica Pedraza-Meléndez; Joaquín Quiroz-Mercado; Daniel Regalado-Santiago; Silvia Rivero-Arredondo; Erika Sierra-Atanacio; Fernando González-Domínguez; Lucia Hernández-Rivas, Irma López-Martínez; Ernesto Ramírez-González; Maribel González-Villa
EPI_ISL_17703577	LESP Mexico City	Instituto de Diagnostico y Referencia Epidemiologicos (INDRE)	Abril Rodríguez-Maldonado; Claudia Wong-Arámbula; Felipe Arguijo-Perez; Helios Cárdenas-Hernández; Carmen Castro-Méndez; Lidia García-Torres; Ruth Madera-Sandoval; América Mandujano-Martínez; Nancy Martínez-Velázquez; Mireya Mederos-Michel; Angélica Pedraza-Meléndez; Joaquín Quiroz-Mercado; Daniel Regalado-Santiago; Silvia Rivero-Arredondo; Erika Sierra-Atanacio; Fernando González-Domínguez; Lucia Hernández-Rivas, Irma López-Martínez; Ernesto Ramírez-González; Maribel González-Villa
EPI_ISL_17703578	LESP Yucatan	Instituto de Diagnostico y Referencia Epidemiologicos (INDRE)	Abril Rodríguez-Maldonado; Claudia Wong-Arámbula; Felipe Arguijo-Perez; Helios Cárdenas-Hernández; Carmen Castro-Méndez; Lidia García-Torres; Ruth Madera-Sandoval; América Mandujano-Martínez; Nancy Martínez-Velázquez; Mireya Mederos-Michel; Angélica Pedraza-Meléndez; Joaquín Quiroz-Mercado; Daniel Regalado-Santiago; Silvia Rivero-Arredondo; Erika Sierra-Atanacio; Fernando González-Domínguez; Lucia Hernández-Rivas, Irma López-Martínez; Ernesto Ramírez-González; Maribel González-Villa
EPI_ISL_17703579	LESP Quintana Roo	Instituto de Diagnostico y Referencia Epidemiologicos (INDRE)	Abril Rodríguez-Maldonado; Claudia Wong-Arámbula; Felipe Arguijo-Perez; Helios Cárdenas-Hernández; Carmen Castro-Méndez; Lidia García-Torres; Ruth Madera-Sandoval; América Mandujano-Martínez; Nancy Martínez-Velázquez; Mireya Mederos-Michel; Angélica Pedraza-Meléndez; Joaquín Quiroz-Mercado; Daniel Regalado-Santiago; Silvia Rivero-Arredondo; Erika Sierra-Atanacio; Fernando González-Domínguez; Lucia Hernández-Rivas, Irma López-Martínez; Ernesto Ramírez-González; Maribel González-Villa
EPI_ISL_17703580	LESP Hidalgo	Instituto de Diagnostico y Referencia Epidemiologicos (INDRE)	Abril Rodríguez-Maldonado; Claudia Wong-Arámbula; Felipe Arguijo-Perez; Helios Cárdenas-Hernández; Carmen Castro-Méndez; Lidia García-Torres; Ruth Madera-Sandoval; América Mandujano-Martínez; Nancy Martínez-Velázquez; Mireya Mederos-Michel; Angélica Pedraza-Meléndez; Joaquín Quiroz-Mercado; Daniel Regalado-Santiago; Silvia Rivero-Arredondo; Erika Sierra-Atanacio; Fernando González-Domínguez; Lucia Hernández-Rivas, Irma López-Martínez; Ernesto Ramírez-González; Maribel González-Villa
EPI_ISL_17703581	LESP Yucatan	Instituto de Diagnostico y Referencia Epidemiologicos (INDRE)	Abril Rodríguez-Maldonado; Claudia Wong-Arámbula; Felipe Arguijo-Perez; Helios Cárdenas-Hernández; Carmen Castro-Méndez; Lidia García-Torres; Ruth Madera-Sandoval; América Mandujano-Martínez; Nancy Martínez-Velázquez; Mireya Mederos-Michel; Angélica Pedraza-Meléndez; Joaquín Quiroz-Mercado; Daniel Regalado-Santiago; Silvia Rivero-Arredondo; Erika Sierra-Atanacio; Fernando González-Domínguez; Lucia Hernández-Rivas, Irma López-Martínez; Ernesto Ramírez-González; Maribel González-Villa
EPI_ISL_17703582	LESP Nuevo Leon	Instituto de Diagnostico y Referencia Epidemiologicos (INDRE)	Abril Rodríguez-Maldonado; Claudia Wong-Arámbula; Felipe Arguijo-Perez; Helios Cárdenas-Hernández; Carmen Castro-Méndez; Lidia García-Torres; Ruth Madera-Sandoval; América Mandujano-Martínez; Nancy Martínez-Velázquez; Mireya Mederos-Michel; Angélica Pedraza-Meléndez; Joaquín Quiroz-Mercado; Daniel Regalado-Santiago; Silvia Rivero-Arredondo; Erika Sierra-Atanacio; Fernando González-Domínguez; Lucia Hernández-Rivas, Irma López-Martínez; Ernesto Ramírez-González; Maribel González-Villa
EPI_ISL_17703583	LESP Quintana Roo	Instituto de Diagnostico y Referencia Epidemiologicos (INDRE)	Abril Rodríguez-Maldonado; Claudia Wong-Arámbula; Felipe Arguijo-Perez; Helios Cárdenas-Hernández; Carmen Castro-Méndez; Lidia García-Torres; Ruth Madera-Sandoval; América Mandujano-Martínez; Nancy Martínez-Velázquez; Mireya Mederos-Michel; Angélica Pedraza-Meléndez; Joaquín Quiroz-Mercado; Daniel Regalado-Santiago; Silvia Rivero-Arredondo; Erika Sierra-Atanacio; Fernando González-Domínguez; Lucia Hernández-Rivas, Irma López-Martínez; Ernesto Ramírez-González; Maribel González-Villa
EPI_ISL_17703584	LESP Chihuahua	Instituto de Diagnostico y Referencia Epidemiologicos (INDRE)	Abril Rodríguez-Maldonado; Claudia Wong-Arámbula; Felipe Arguijo-Perez; Helios Cárdenas-Hernández; Carmen Castro-Méndez; Lidia García-Torres; Ruth Madera-Sandoval; América Mandujano-Martínez; Nancy Martínez-Velázquez; Mireya Mederos-Michel; Angélica Pedraza-Meléndez; Joaquín Quiroz-Mercado; Daniel Regalado-Santiago; Silvia Rivero-Arredondo; Erika Sierra-Atanacio; Fernando González-Domínguez; Lucia Hernández-Rivas, Irma López-Martínez; Ernesto Ramírez-González; Maribel González-Villa
EPI_ISL_17703585	LESP Yucatan	Instituto de Diagnostico y Referencia Epidemiologicos (INDRE)	Abril Rodríguez-Maldonado; Claudia Wong-Arámbula; Felipe Arguijo-Perez; Helios Cárdenas-Hernández; Carmen Castro-Méndez; Lidia García-Torres; Ruth Madera-Sandoval; América Mandujano-Martínez; Nancy Martínez-Velázquez; Mireya Mederos-Michel; Angélica Pedraza-Meléndez; Joaquín Quiroz-Mercado; Daniel Regalado-Santiago; Silvia Rivero-Arredondo; Erika Sierra-Atanacio; Fernando González-Domínguez; Lucia Hernández-Rivas, Irma López-Martínez; Ernesto Ramírez-González; Maribel González-Villa
EPI_ISL_17703586, EPI_ISL_17703587	LESP Nuevo Leon	Instituto de Diagnostico y Referencia Epidemiologicos (INDRE)	Abril Rodríguez-Maldonado; Claudia Wong-Arámbula; Felipe Arguijo-Perez; Helios Cárdenas-Hernández; Carmen Castro-Méndez; Lidia García-Torres; Ruth Madera-Sandoval; América Mandujano-Martínez; Nancy Martínez-Velázquez; Mireya Mederos-Michel; Angélica Pedraza-Meléndez; Joaquín Quiroz-Mercado; Daniel Regalado-Santiago; Silvia Rivero-Arredondo; Erika Sierra-Atanacio; Fernando González-Domínguez; Lucia Hernández-Rivas, Irma López-Martínez; Ernesto Ramírez-González; Maribel González-Villa
EPI_ISL_17703588, EPI_ISL_17703589	LESP Quintana Roo	Instituto de Diagnostico y Referencia Epidemiologicos (INDRE)	Abril Rodríguez-Maldonado; Claudia Wong-Arámbula; Felipe Arguijo-Perez; Helios Cárdenas-Hernández; Carmen Castro-Méndez; Lidia García-Torres; Ruth Madera-Sandoval; América Mandujano-Martínez; Nancy Martínez-Velázquez; Mireya Mederos-Michel; Angélica Pedraza-Meléndez; Joaquín Quiroz-Mercado; Daniel Regalado-Santiago; Silvia Rivero-Arredondo; Erika Sierra-Atanacio; Fernando González-Domínguez; Lucia Hernández-Rivas, Irma López-Martínez; Ernesto Ramírez-González; Maribel González-Villa
EPI_ISL_17703590	LESP State of Mexico	Instituto de Diagnostico y Referencia Epidemiologicos (INDRE)	Abril Rodríguez-Maldonado; Claudia Wong-Arámbula; Felipe Arguijo-Perez; Helios Cárdenas-Hernández; Carmen Castro-Méndez; Lidia García-Torres; Ruth Madera-Sandoval; América Mandujano-Martínez; Nancy Martínez-Velázquez; Mireya Mederos-Michel; Angélica Pedraza-Meléndez; Joaquín Quiroz-Mercado; Daniel Regalado-Santiago; Silvia Rivero-Arredondo; Erika Sierra-Atanacio; Fernando González-Domínguez; Lucia Hernández-Rivas, Irma López-Martínez; Ernesto Ramírez-González; Maribel González-Villa
EPI_ISL_17703591	LESP Campeche	Instituto de Diagnostico y Referencia Epidemiologicos (INDRE)	Abril Rodríguez-Maldonado; Claudia Wong-Arámbula; Felipe Arguijo-Perez; Helios Cárdenas-Hernández; Carmen Castro-Méndez; Lidia García-Torres; Ruth Madera-Sandoval; América Mandujano-Martínez; Nancy Martínez-Velázquez; Mireya Mederos-Michel; Angélica Pedraza-Meléndez; Joaquín Quiroz-Mercado; Daniel Regalado-Santiago; Silvia Rivero-Arredondo; Erika Sierra-Atanacio; Fernando González-Domínguez; Lucia Hernández-Rivas, Irma López-Martínez; Ernesto Ramírez-González; Maribel González-Villa
EPI_ISL_17703592	LESP Nuevo Leon	Instituto de Diagnostico y Referencia Epidemiologicos (INDRE)	Abril Rodríguez-Maldonado; Claudia Wong-Arámbula; Felipe Arguijo-Perez; Helios Cárdenas-Hernández; Carmen Castro-Méndez; Lidia García-Torres; Ruth Madera-Sandoval; América Mandujano-Martínez; Nancy Martínez-Velázquez; Mireya Mederos-Michel; Angélica Pedraza-Meléndez; Joaquín Quiroz-Mercado; Daniel Regalado-Santiago; Silvia Rivero-Arredondo; Erika Sierra-Atanacio; Fernando González-Domínguez; Lucia Hernández-Rivas, Irma López-Martínez; Ernesto Ramírez-González; Maribel González-Villa
EPI_ISL_17703593, EPI_ISL_17703594	LESP Jalisco	Instituto de Diagnostico y Referencia Epidemiologicos (INDRE)	Abril Rodríguez-Maldonado; Claudia Wong-Arámbula; Felipe Arguijo-Perez; Helios Cárdenas-Hernández; Carmen Castro-Méndez; Lidia García-Torres; Ruth Madera-Sandoval; América Mandujano-Martínez; Nancy Martínez-Velázquez; Mireya Mederos-Michel; Angélica Pedraza-Meléndez; Joaquín Quiroz-Mercado; Daniel Regalado-Santiago; Silvia Rivero-Arredondo; Erika Sierra-Atanacio; Fernando González-Domínguez; Lucia Hernández-Rivas, Irma López-Martínez; Ernesto Ramírez-González; Maribel González-Villa
EPI_ISL_17703595, EPI_ISL_17703596	LESP Nuevo Leon	Instituto de Diagnostico y Referencia Epidemiologicos (INDRE)	Abril Rodríguez-Maldonado; Claudia Wong-Arámbula; Felipe Arguijo-Perez; Helios Cárdenas-Hernández; Carmen Castro-Méndez; Lidia García-Torres; Ruth Madera-Sandoval; América Mandujano-Martínez; Nancy Martínez-Velázquez; Mireya Mederos-Michel; Angélica Pedraza-Meléndez; Joaquín Quiroz-Mercado; Daniel Regalado-Santiago; Silvia Rivero-Arredondo; Erika Sierra-Atanacio; Fernando González-Domínguez; Lucia Hernández-Rivas, Irma López-Martínez; Ernesto Ramírez-González; Maribel González-Villa
EPI_ISL_17703597	Laboratorio Estatal de Salud Pública Nuevo Leon	Instituto de Diagnostico y Referencia Epidemiologicos (INDRE)	Abril Rodríguez-Maldonado; Claudia Wong-Arámbula; Felipe Arguijo-Perez; Helios Cárdenas-Hernández; Carmen Castro-Méndez; Lidia García-Torres; Ruth Madera-Sandoval; América Mandujano-Martínez; Nancy Martínez-Velázquez; Mireya Mederos-Michel; Angélica Pedraza-Meléndez; Joaquín Quiroz-Mercado; Daniel Regalado-Santiago; Silvia Rivero-Arredondo; Erika Sierra-Atanacio; Fernando González-Domínguez; Lucia Hernández-Rivas, Irma López-Martínez; Ernesto Ramírez-González; Maribel González-Villa
EPI_ISL_17703598	LESP Quintana Roo	Instituto de Diagnostico y Referencia Epidemiologicos (INDRE)	Abril Rodríguez-Maldonado; Claudia Wong-Arámbula; Felipe Arguijo-Perez; Helios Cárdenas-Hernández; Carmen Castro-Méndez; Lidia García-Torres; Ruth Madera-Sandoval; América Mandujano-Martínez; Nancy Martínez-Velázquez; Mireya Mederos-Michel; Angélica Pedraza-Meléndez; Joaquín Quiroz-Mercado; Daniel Regalado-Santiago; Silvia Rivero-Arredondo; Erika Sierra-Atanacio; Fernando González-Domínguez; Lucia Hernández-Rivas, Irma López-Martínez; Ernesto Ramírez-González; Maribel González-Villa
EPI_ISL_17703599, EPI_ISL_17703600, EPI_ISL_17703601, EPI_ISL_17703602, EPI_ISL_17703603, EPI_ISL_17703604	LESP Nuevo Leon	Instituto de Diagnostico y Referencia Epidemiologicos (INDRE)	Abril Rodríguez-Maldonado; Claudia Wong-Arámbula; Felipe Arguijo-Perez; Helios Cárdenas-Hernández; Carmen Castro-Méndez; Lidia García-Torres; Ruth Madera-Sandoval; América Mandujano-Martínez; Nancy Martínez-Velázquez; Mireya Mederos-Michel; Angélica Pedraza-Meléndez; Joaquín Quiroz-Mercado; Daniel Regalado-Santiago; Silvia Rivero-Arredondo; Erika Sierra-Atanacio; Fernando González-Domínguez; Lucia Hernández-Rivas, Irma López-Martínez; Ernesto Ramírez-González; Maribel González-Villa
EPI_ISL_17703605	LESP Chihuahua	Instituto de Diagnostico y Referencia Epidemiologicos (INDRE)	Abril Rodríguez-Maldonado; Claudia Wong-Arámbula; Felipe Arguijo-Perez; Helios Cárdenas-Hernández; Carmen Castro-Méndez; Lidia García-Torres; Ruth Madera-Sandoval; América Mandujano-Martínez; Nancy Martínez-Velázquez; Mireya Mederos-Michel; Angélica Pedraza-Meléndez; Joaquín Quiroz-Mercado; Daniel Regalado-Santiago; Silvia Rivero-Arredondo; Erika Sierra-Atanacio; Fernando González-Domínguez; Lucia Hernández-Rivas, Irma López-Martínez; Ernesto Ramírez-González; Maribel González-Villa
EPI_ISL_17703606	LESP Nuevo Leon	Instituto de Diagnostico y Referencia Epidemiologicos (INDRE)	Abril Rodríguez-Maldonado; Claudia Wong-Arámbula; Felipe Arguijo-Perez; Helios Cárdenas-Hernández; Carmen Castro-Méndez; Lidia García-Torres; Ruth Madera-Sandoval; América Mandujano-Martínez; Nancy Martínez-Velázquez; Mireya Mederos-Michel; Angélica Pedraza-Meléndez; Joaquín Quiroz-Mercado; Daniel Regalado-Santiago; Silvia Rivero-Arredondo; Erika Sierra-Atanacio; Fernando González-Domínguez; Lucia Hernández-Rivas, Irma López-Martínez; Ernesto Ramírez-González; Maribel González-Villa
EPI_ISL_17703607, EPI_ISL_17703608, EPI_ISL_17703609, EPI_ISL_17703610	LESP Mexico City	Instituto de Diagnostico y Referencia Epidemiologicos (INDRE)	Abril Rodríguez-Maldonado; Claudia Wong-Arámbula; Felipe Arguijo-Perez; Helios Cárdenas-Hernández; Carmen Castro-Méndez; Lidia García-Torres; Ruth Madera-Sandoval; América Mandujano-Martínez; Nancy Martínez-Velázquez; Mireya Mederos-Michel; Angélica Pedraza-Meléndez; Joaquín Quiroz-Mercado; Daniel Regalado-Santiago; Silvia Rivero-Arredondo; Erika Sierra-Atanacio; Fernando González-Domínguez; Lucia Hernández-Rivas, Irma López-Martínez; Ernesto Ramírez-González; Maribel González-Villa
EPI_ISL_17703611	LESP Tlaxcala	Instituto de Diagnostico y Referencia Epidemiologicos (INDRE)	Abril Rodríguez-Maldonado; Claudia Wong-Arámbula; Felipe Arguijo-Perez; Helios Cárdenas-Hernández; Carmen Castro-Méndez; Lidia García-Torres; Ruth Madera-Sandoval; América Mandujano-Martínez; Nancy Martínez-Velázquez; Mireya Mederos-Michel; Angélica Pedraza-Meléndez; Joaquín Quiroz-Mercado; Daniel Regalado-Santiago; Silvia Rivero-Arredondo; Erika Sierra-Atanacio; Fernando González-Domínguez; Lucia Hernández-Rivas, Irma López-Martínez; Ernesto Ramírez-González; Maribel González-Villa

EPI_ISL_17703612	LESP Nuevo Leon	Instituto de Diagnostico y Referencia Epidemiologicos (INDRE)	Abril Rodríguez-Maldonado; Claudia Wong-Arámbula; Felipe Arguijo-Perez; Helios Cárdenas-Hernández; Carmen Castro-Méndez; Lidia García-Torres; Ruth Madera-Sandoval; América Mandujano-Martínez; Nancy Martínez-Velázquez; Mireya Mederos-Michel; Angélica Pedraza-Meléndez; Joaquín Quiroz-Mercado; Daniel Regalado-Santiago; Silvia Rivero-Arredondo; Erika Sierra-Atanacio; Fernando González-Domínguez; Lucia Hernández-Rivas, Irma López-Martínez; Ernesto Ramírez-González; Maribel González-Villa
EPI_ISL_17703744	Parasitology Laboratory, Institute of Tropical Medicine of Sao Paulo, School of Medicine, University of Sao Paulo	Parasitology Laboratory	Raissa Heloisa de Araujo Eliodoro, Ingra Claro Morales, Ester Cerdeira Sabino
EPI_ISL_17703745	Parasitology Laboratory, Institute of Tropical Medicine of Sao Paulo, School of Medicine, University of Sao Paulo	Parasitology Laboratory	Raissa Heloisa de Araujo Eliodoro, Ingra Morales Claro, Ester Cerdeira Sabino
EPI_ISL_17703746	Parasitology Laboratory, Institute of Tropical Medicine of Sao Paulo, School of Medicine, University of Sao Paulo	Parasitology Laboratory	Raissa Heloisa de Araujo Eliodoro, Ingra Claro Morales, Ester Cerdeira Sabino
EPI_ISL_17718458	Department of Virology, National Institute of Health, Islamabad, Pakistan	Department of Virology, National Institute of Health, Islamabad, Pakistan	Massab Umair, Muhammad Ammar, Syed Adnan Haider, Rabia Hakim, Qasim Malik, Muhammad Salman, Ghazala Parveen, and Naseem Akhtar
EPI_ISL_17722468	Tokyo Metropolitan Institute of Public Health	Tokyo Metropolitan Institute of Public Health	Fumi Kasuya, Wakaba Okada, Ryota Kumagai, Sachiko Harada, Arisa Amano, Michiya Hasegawa, Mami Nagashima, Kenji Sadamasu
EPI_ISL_17736865, EPI_ISL_17736866, EPI_ISL_17736867, EPI_ISL_17736868, EPI_ISL_17736869, EPI_ISL_17736870, EPI_ISL_17736871, EPI_ISL_17736872, EPI_ISL_17736873, EPI_ISL_17736874, EPI_ISL_17736875, EPI_ISL_17736876, EPI_ISL_17736877, EPI_ISL_17736878, EPI_ISL_17736879, EPI_ISL_17736880, EPI_ISL_17736881, EPI_ISL_17736882, EPI_ISL_17736883, EPI_ISL_17736884, EPI_ISL_17736885, EPI_ISL_17736886, EPI_ISL_17736887, EPI_ISL_17736888, EPI_ISL_17736889, EPI_ISL_17736890, EPI_ISL_17736891			
see above	Charité Universitätsmedizin Berlin, Institute for Virology/Laboratory Berlin	Charité Universitätsmedizin Berlin, Institute for Virology	Terry C. Jones, Julia Melchert, Barbara Mühlemann, Talitha Veith, Jörn Beheim-Schwarzbach, Julia Tesch, Marie Luisa Schmidt, Felix Walper, Tobias Bleicker, Caroline Isner, Frieder Pfäfflin, Ricardo Niklas Werner, Victor M. Corman, Christian Drosten
EPI_ISL_17737466, EPI_ISL_17737467, EPI_ISL_17737468, EPI_ISL_17737469, EPI_ISL_17737470, EPI_ISL_17737471, EPI_ISL_17737472, EPI_ISL_17737473, EPI_ISL_17737474, EPI_ISL_17737475, EPI_ISL_17737476, EPI_ISL_17737477, EPI_ISL_17737478, EPI_ISL_17737479, EPI_ISL_17737480, EPI_ISL_17737481, EPI_ISL_17737482, EPI_ISL_17737483, EPI_ISL_17737484, EPI_ISL_17737485, EPI_ISL_17737486, EPI_ISL_17737488, EPI_ISL_17737489, EPI_ISL_17737490, EPI_ISL_17737491, EPI_ISL_17737492, EPI_ISL_17737493, EPI_ISL_17737495, EPI_ISL_17737496, EPI_ISL_17737497, EPI_ISL_17737498, EPI_ISL_17737499, EPI_ISL_17737500, EPI_ISL_17737501, EPI_ISL_17737502, EPI_ISL_17737503, EPI_ISL_17737504, EPI_ISL_17737505, EPI_ISL_17737506, EPI_ISL_17737507, EPI_ISL_17737508, EPI_ISL_17737509, EPI_ISL_17737510, EPI_ISL_17737511, EPI_ISL_17737512, EPI_ISL_17737513, EPI_ISL_17737514, EPI_ISL_17737515, EPI_ISL_17737516, EPI_ISL_17737517, EPI_ISL_17737518, EPI_ISL_17737519, EPI_ISL_17737520, EPI_ISL_17737521, EPI_ISL_17737522, EPI_ISL_17737523, EPI_ISL_17737524, EPI_ISL_17737525, EPI_ISL_17737526, EPI_ISL_17737527, EPI_ISL_17737528, EPI_ISL_17737529, EPI_ISL_17737530, EPI_ISL_17737531, EPI_ISL_17737532, EPI_ISL_17737533, EPI_ISL_17737534, EPI_ISL_17737535, EPI_ISL_17737536, EPI_ISL_17737537, EPI_ISL_17737538, EPI_ISL_17737539, EPI_ISL_17737540, EPI_ISL_17737541, EPI_ISL_17737542, EPI_ISL_17737543, EPI_ISL_17737544, EPI_ISL_17737545, EPI_ISL_17737546, EPI_ISL_17737547, EPI_ISL_17737548, EPI_ISL_17737549, EPI_ISL_17737550, EPI_ISL_17737551, EPI_ISL_17737553, EPI_ISL_17737555, EPI_ISL_17737556			
see above	Public Health Ontario	Public Health Ontario	Isabel S, Eshaghi A, Duvvuri VR, Gubbay JB, Cronin K, Li A, Hasso M, Clark ST, Hopkins JP, Patel SN, Braukmann TWA
EPI_ISL_17762484, EPI_ISL_17762485	Tokyo Metropolitan Institute of Public Health	Tokyo Metropolitan Institute of Public Health	Fumi Kasuya, Wakaba Okada, Ryota Kumagai, Sachiko Harada, Arisa Amano, Michiya Hasegawa, Mami Nagashima, Kenji Sadamasu
EPI_ISL_17779992	Servicio de Microbiologia Hospital Ramon y Cajal	Servicio de Microbiologia Hospital Ramon y Cajal	Ponce-Alonso M, Martinez-García L, Olavarrieta L, Galan JC
EPI_ISL_17793219	Microbiology, Immunology and Transplantation, KU Leuven	Microbiology, Immunology and Transplantation, KU Leuven	Wawina-Bokalanga,T., Vanmechelen,B., Logist,A.-S., Bloemen,M. and Maes,P.
EPI_ISL_17793220	Microbiology, Immunology and Transplantation, KU Leuven	Microbiology, Immunology and Transplantation, KU Leuven	Vanmechelen,B., Wawina-Bokalanga,T., Logist,A.-S., Bloemen,M. and Maes,P.
EPI_ISL_17793221, EPI_ISL_17793222	Microbiology, Immunology and Transplantation, KU Leuven	Microbiology, Immunology and Transplantation, KU Leuven	Wawina-Bokalanga,T., Vanmechelen,B., Logist,A.-S., Bloemen,M. and Maes,P.
EPI_ISL_17793223, EPI_ISL_17793224, EPI_ISL_17793225	Microbiology, Immunology and Transplantation, KU Leuven	Microbiology, Immunology and Transplantation, KU Leuven	Vanmechelen,B., Wawina-Bokalanga,T., Logist,A.-S., Bloemen,M. and Maes,P.
EPI_ISL_17793226	Microbiology, Immunology and Transplantation, KU Leuven	Microbiology, Immunology and Transplantation, KU Leuven	Wawina-Bokalanga,T., Vanmechelen,B., Logist,A.-S., Bloemen,M. and Maes,P.
EPI_ISL_17793227	Microbiology, Immunology and Transplantation, KU Leuven	Microbiology, Immunology and Transplantation, KU Leuven	Vanmechelen,B., Wawina-Bokalanga,T., Logist,A.-S., Bloemen,M. and Maes,P.
EPI_ISL_17793230	Microbiology, Immunology and Transplantation, KU Leuven	Microbiology, Immunology and Transplantation, KU Leuven	Wawina-Bokalanga,T., Vanmechelen,B., Logist,A.-S., Sinnesael,R., Ysebaert,L., Verlinden,J., Van Holm,B., Bloemen,M. and Maes,P.
EPI_ISL_17793231	Microbiology, Immunology and Transplantation, KU Leuven	Microbiology, Immunology and Transplantation, KU Leuven	Vanmechelen,B., Wawina-Bokalanga,T., Logist,A.-S., Bloemen,M. and Maes,P.
EPI_ISL_17793232	Microbiology, Immunology and Transplantation, KU Leuven	Microbiology, Immunology and Transplantation, KU Leuven	Wawina-Bokalanga,T., Vanmechelen,B., Logist,A.-S., Bloemen,M. and Maes,P.
EPI_ISL_17793233, EPI_ISL_17793234	Microbiology, Immunology and Transplantation, KU Leuven	Microbiology, Immunology and Transplantation, KU Leuven	Vanmechelen,B., Wawina-Bokalanga,T., Logist,A.-S., Bloemen,M. and Maes,P.
EPI_ISL_17793235	Microbiology, Immunology and Transplantation, KU Leuven	Microbiology, Immunology and Transplantation, KU Leuven	Wawina-Bokalanga,T., Vanmechelen,B., Logist,A.-S., Bloemen,M. and Maes,P.
EPI_ISL_17793236, EPI_ISL_17793239	Microbiology, Immunology and Transplantation, KU Leuven	Microbiology, Immunology and Transplantation, KU Leuven	Vanmechelen,B., Wawina-Bokalanga,T., Logist,A.-S., Bloemen,M. and Maes,P.
EPI_ISL_17793241	Microbiology, Immunology and Transplantation, KU Leuven	Microbiology, Immunology and Transplantation, KU Leuven	Wawina-Bokalanga,T., Vanmechelen,B., Logist,A.-S., Bloemen,M. and Maes,P.
EPI_ISL_17793242, EPI_ISL_17793243	Microbiology, Immunology and Transplantation, KU Leuven	Microbiology, Immunology and Transplantation, KU Leuven	Vanmechelen,B., Wawina-Bokalanga,T., Logist,A.-S., Bloemen,M. and Maes,P.
EPI_ISL_17793244	Microbiology, Immunology and Transplantation, KU Leuven	Microbiology, Immunology and Transplantation, KU Leuven	Wawina-Bokalanga,T., Vanmechelen,B., Logist,A.-S., Bloemen,M. and Maes,P.
EPI_ISL_17793247, EPI_ISL_17793248, EPI_ISL_17793250	Microbiology, Immunology and Transplantation, KU Leuven	Microbiology, Immunology and Transplantation, KU Leuven	Vanmechelen,B., Wawina-Bokalanga,T., Logist,A.-S., Bloemen,M. and Maes,P.
EPI_ISL_17793251	Microbiology, Immunology and Transplantation, KU Leuven	Microbiology, Immunology and Transplantation, KU Leuven	Wawina-Bokalanga,T., Vanmechelen,B., Logist,A.-S., Bloemen,M. and Maes,P.
EPI_ISL_17793252	Microbiology, Immunology and Transplantation, KU Leuven	Microbiology, Immunology and Transplantation, KU Leuven	Vanmechelen,B., Wawina-Bokalanga,T., Logist,A.-S., Bloemen,M. and Maes,P.
EPI_ISL_17793254	Microbiology, Immunology and Transplantation, KU Leuven	Microbiology, Immunology and Transplantation, KU Leuven	Wawina-Bokalanga,T., Vanmechelen,B., Logist,A.-S., Ysebaert,L., Horemans,M., Verlinden,J., Van Holm,B., Bloemen,M. and Maes,P.
EPI_ISL_17793255	Microbiology, Immunology and Transplantation, KU Leuven	Microbiology, Immunology and Transplantation, KU Leuven	Wawina-Bokalanga,T., Vanmechelen,B., Logist,A.-S., Sinnesael,R., Ysebaert,L., Verlinden,J., Van Holm,B., Bloemen,M. and Maes,P.
EPI_ISL_17793256	Microbiology, Immunology and Transplantation, KU Leuven	Microbiology, Immunology and Transplantation, KU Leuven	Vanmechelen,B., Wawina-Bokalanga,T., Logist,A.-S., Bloemen,M. and Maes,P.
EPI_ISL_17793257	Microbiology, Immunology and Transplantation, KU Leuven	Microbiology, Immunology and Transplantation, KU Leuven	Wawina-Bokalanga,T., Vanmechelen,B., Logist,A.-S., Bloemen,M. and Maes,P.
EPI_ISL_17793258	Microbiology, Immunology and Transplantation, KU Leuven	Microbiology, Immunology and Transplantation, KU Leuven	Vanmechelen,B., Wawina-Bokalanga,T., Logist,A.-S., Bloemen,M. and Maes,P.
EPI_ISL_17793259, EPI_ISL_17793260, EPI_ISL_17793261	Microbiology, Immunology and Transplantation, KU Leuven	Microbiology, Immunology and Transplantation, KU Leuven	Wawina-Bokalanga,T., Vanmechelen,B., Logist,A.-S., Bloemen,M. and Maes,P.
EPI_ISL_17793262, EPI_ISL_17793263, EPI_ISL_17793264, EPI_ISL_17793265, EPI_ISL_17793267	Microbiology, Immunology and Transplantation, KU Leuven	Microbiology, Immunology and Transplantation, KU Leuven	Vanmechelen,B., Wawina-Bokalanga,T., Logist,A.-S., Bloemen,M. and Maes,P.
EPI_ISL_17793268	Microbiology, Immunology and Transplantation, KU Leuven	Microbiology, Immunology and Transplantation, KU Leuven	Wawina-Bokalanga,T., Vanmechelen,B., Logist,A.-S., Ysebaert,L., Verlinden,J., Sinnesael,R., Van Holm,B., Bloemen,M. and Maes,P.
EPI_ISL_17793269	Microbiology, Immunology and Transplantation, KU Leuven	Microbiology, Immunology and Transplantation, KU Leuven	Wawina-Bokalanga,T., Vanmechelen,B., Logist,A.-S., Bloemen,M. and Maes,P.
EPI_ISL_17793270	Microbiology, Immunology and Transplantation, KU Leuven	Microbiology, Immunology and Transplantation, KU Leuven	Vanmechelen,B., Wawina-Bokalanga,T., Logist,A.-S., Bloemen,M. and Maes,P.
EPI_ISL_17793271	Microbiology, Immunology and Transplantation, KU Leuven	Microbiology, Immunology and Transplantation, KU Leuven	Wawina-Bokalanga,T., Vanmechelen,B., Logist,A.-S., Bloemen,M., Van Holm,B. and Maes,P.
EPI_ISL_17793273, EPI_ISL_17793275	Microbiology, Immunology and Transplantation, KU Leuven	Microbiology, Immunology and Transplantation, KU Leuven	Wawina-Bokalanga,T., Vanmechelen,B., Logist,A.-S., Bloemen,M. and Maes,P.
EPI_ISL_17793277	Microbiology, Immunology and Transplantation, KU Leuven	Microbiology, Immunology and Transplantation, KU Leuven	Vanmechelen,B., Wawina-Bokalanga,T., Logist,A.-S., Bloemen,M. and Maes,P.
EPI_ISL_17793278	Microbiology, Immunology and Transplantation, KU	Microbiology, Immunology and Transplantation, KU	Wawina-Bokalanga,T., Mechelen,B., Logist,A.-S., Bloemen,M. and Maes,P.

	Leuven	Leuven	
EPI_ISL_17793280, EPI_ISL_17793281	Microbiology, Immunology and Transplantation, KU Leuven	Microbiology, Immunology and Transplantation, KU Leuven	Vanmechelen,B., Wawina-Bokalanga,T., Logist,A.-S., Bloemen,M. and Maes,P.
EPI_ISL_17793282	Microbiology, Immunology and Transplantation, KU Leuven	Microbiology, Immunology and Transplantation, KU Leuven	Vanmechelen,B., Wawina-Bokalanga,T., Logist,A.-S., Van Holm,B., Bloemen,M. and Maes,P.
EPI_ISL_17793283	Microbiology, Immunology and Transplantation, KU Leuven	Microbiology, Immunology and Transplantation, KU Leuven	Wawina-Bokalanga,T., Vanmechelen,B., Logist,A.-S., Bloemen,M. and Maes,P.
EPI_ISL_17793284, EPI_ISL_17793286	Microbiology, Immunology and Transplantation, KU Leuven	Microbiology, Immunology and Transplantation, KU Leuven	Vanmechelen,B., Wawina-Bokalanga,T., Logist,A.-S., Bloemen,M. and Maes,P.
EPI_ISL_17793287	Microbiology, Immunology and Transplantation, KU Leuven	Microbiology, Immunology and Transplantation, KU Leuven	Vanmechelen,B., Wawona-Bokalanga,T., Logist,A.-S., Bloemen,M. and Maes,P.
EPI_ISL_17793288	Microbiology, Immunology and Transplantation, KU Leuven	Microbiology, Immunology and Transplantation, KU Leuven	Wawina-Bokalanga,T., Vanmechelen,B., Logist,A.-S., Bloemen,M. and Maes,P.
EPI_ISL_17793290	Microbiology, Immunology and Transplantation, KU Leuven	Microbiology, Immunology and Transplantation, KU Leuven	Vanmechelen,B., Wawina-Bokalanga,T., Logist,A.-S., Bloemen,M. and Maes,P.
EPI_ISL_17793292, EPI_ISL_17793293, EPI_ISL_17793295, EPI_ISL_17797716	Microbiology, Immunology and Transplantation, KU Leuven	Microbiology, Immunology and Transplantation, KU Leuven	Wawina-Bokalanga,T., Vanmechelen,B., Logist,A.-S., Bloemen,M. and Maes,P.
EPI_ISL_17797717	Microbiology, Immunology and Transplantation, KU Leuven	Microbiology, Immunology and Transplantation, KU Leuven	Vanmechelen,B., Wawina-Bokalanga,T., Logist,A.-S., Van Holm,B., Bloemen,M. and Maes,P.
EPI_ISL_17797718, EPI_ISL_17797720, EPI_ISL_17797721	Microbiology, Immunology and Transplantation, KU Leuven	Microbiology, Immunology and Transplantation, KU Leuven	Wawina-Bokalanga,T., Vanmechelen,B., Logist,A.-S., Bloemen,M. and Maes,P.
EPI_ISL_17797722	Microbiology, Immunology and Transplantation, KU Leuven	Microbiology, Immunology and Transplantation, KU Leuven	Vanmechelen,B., Wawina-Bokalanga,T., Logist,A.-S., Bloemen,M., Van Holm,B. and Maes,P.
EPI_ISL_17797723	Microbiology, Immunology and Transplantation, KU Leuven	Microbiology, Immunology and Transplantation, KU Leuven	Vanmechelen,B., Wawina-Bokalanga,T., Logist,A.-S., Bloemen,M. and Maes,P.
EPI_ISL_17797725	Microbiology, Immunology and Transplantation, KU Leuven	Microbiology, Immunology and Transplantation, KU Leuven	Wawina-Bokalanga,T., Vanmechelen,B., Logist,A.-S., Bloemen,M. and Maes,P.
EPI_ISL_17797726	Microbiology, Immunology and Transplantation, KU Leuven	Microbiology, Immunology and Transplantation, KU Leuven	Wawina-Bokalanga,T., Vanmechelen,B., Logist,A.-S., Van Holm,B., Bloemen,M. and Maes,P.
EPI_ISL_17797728	Microbiology, Immunology and Transplantation, KU Leuven	Microbiology, Immunology and Transplantation, KU Leuven	Wawina-Bokalanga,T., Vanmechelen,B., Logist,A.-s., Van Holm,B., Bloemen,M. and Maes,P.
EPI_ISL_17797729, EPI_ISL_17797730, EPI_ISL_17797731	Microbiology, Immunology and Transplantation, KU Leuven	Microbiology, Immunology and Transplantation, KU Leuven	Wawina-Bokalanga,T., Vanmechelen,B., Logist,A.-S., Bloemen,M. and Maes,P.
EPI_ISL_17797732	Microbiology, Immunology and Transplantation, KU Leuven	Microbiology, Immunology and Transplantation, KU Leuven	Vanmechelen,B., Wawina-Bokalanga,T., Logist,A.-S., Van Holm,B., Bloemen,M. and Maes,P.
EPI_ISL_17797734, EPI_ISL_17797735, EPI_ISL_17797736	Microbiology, Immunology and Transplantation, KU Leuven	Microbiology, Immunology and Transplantation, KU Leuven	Wawina-Bokalanga,T., Vanmechelen,B., Logist,A.-S., Bloemen,M. and Maes,P.
EPI_ISL_17797738	Microbiology, Immunology and Transplantation, KU Leuven	Microbiology, Immunology and Transplantation, KU Leuven	Wawina-Bokalanga,T., Vanmechelen,B., Logist,A.-S., Van Holm,B., Bloemen,M. and Maes,P.
EPI_ISL_17797739	Microbiology, Immunology and Transplantation, KU Leuven	Microbiology, Immunology and Transplantation, KU Leuven	Vanmechelen,B., Wawina-Bokalanga,T., Logist,A.-S., Van Holm,B., Bloemen,M. and Maes,P.
EPI_ISL_17797740	Microbiology, Immunology and Transplantation, KU Leuven	Microbiology, Immunology and Transplantation, KU Leuven	Vanmechelen,B., Wawina-Bokalanga,T., Logist,A.-S., Bloemen,M. and Maes,P.
EPI_ISL_17797742	Microbiology, Immunology and Transplantation, KU Leuven	Microbiology, Immunology and Transplantation, KU Leuven	Wawina-Bokalanga,T., Vanmechelen,B., Logist,A.-S., Bloemen,M. and Maes,P.
EPI_ISL_17797744	Microbiology, Immunology and Transplantation, KU Leuven	Microbiology, Immunology and Transplantation, KU Leuven	Vanmechelen,B., Wawina-Bokalanga,T., Logist,A.-S., Bloemen,M., Van Holm,B. and Maes,P.
EPI_ISL_17797745, EPI_ISL_17797746	Microbiology, Immunology and Transplantation, KU Leuven	Microbiology, Immunology and Transplantation, KU Leuven	Wawina-Bokalanga,T., Vanmechelen,B., Logist,A.-S., Bloemen,M. and Maes,P.
EPI_ISL_17797747	Microbiology, Immunology and Transplantation, KU Leuven	Microbiology, Immunology and Transplantation, KU Leuven	Wawina-Bokalanga,T., Vanmechelen,B., Logist,A.-S., Van Holm,B., Bloemen,M. and Maes,P.
EPI_ISL_17797750	Microbiology, Immunology and Transplantation, KU Leuven	Microbiology, Immunology and Transplantation, KU Leuven	Vanmechelen,B., Wawina-Bokalanga,T., Logist,A.-S., Van Holm,B., Bloemen,M. and Maes,P.
EPI_ISL_17797751	Microbiology, Immunology and Transplantation, KU Leuven	Microbiology, Immunology and Transplantation, KU Leuven	Wawina-Bokalanga,T., Vanmechelen,B., Logist,A.-S., Van Holm,B., Bloemen,M. and Maes,P.
EPI_ISL_17797752	Microbiology, Immunology and Transplantation, KU Leuven	Microbiology, Immunology and Transplantation, KU Leuven	Vanmechelen,B., Wawina-Bokalanga,T., Logist,A.-S., Van Holm,B., Bloemen,M. and Maes,P.
EPI_ISL_17797754	Microbiology, Immunology and Transplantation, KU Leuven	Microbiology, Immunology and Transplantation, KU Leuven	Wawina-Bokalanga,T., Vanmechelen,B., Logist,A.-S., Bloemen,M. and Maes,P.
EPI_ISL_17809521	Hangzhou Center for Disease Control and Prevention	Hangzhou Center for Disease Control and Prevention	Lijiao Ao , Jun Li , Yue Yu
EPI_ISL_17817239, EPI_ISL_17817240, EPI_ISL_17817241	Tokyo Metropolitan Institute of Public Health	Tokyo Metropolitan Institute of Public Health	Fumi Kasuya, Wakaba Okada, Ryota Kumagai, Sachiko Harada, Arisa Amano, Michiya Hasegawa, Mami Nagashima, Kenji Sadamasu
EPI_ISL_17821096, EPI_ISL_17821097, EPI_ISL_17821098	ACL Laboratories	RIPHL at Rush University Medical Center	Stefan Green, Kevin Kunstman, Hannah Barbian, Felix Araujo Perez, Edith Perez, Sofiya Bobrovska, Alyse Kittner, Cecilia Chau, Giancarlo Balangue, Lok Yiu Ashley Wu
EPI_ISL_17821099, EPI_ISL_17821100, EPI_ISL_17821101	Quest Diagnostics	RIPHL at Rush University Medical Center	Stefan Green, Kevin Kunstman, Hannah Barbian, Felix Araujo Perez, Edith Perez, Sofiya Bobrovska, Alyse Kittner, Cecilia Chau, Giancarlo Balangue, Lok Yiu Ashley Wu
EPI_ISL_17834476	California Department of Public Health	California Department of Public Health	Kath, C., Haw, M., Espinosa, A., and Hacker, J.
EPI_ISL_17837266, EPI_ISL_17837267, EPI_ISL_17837268, EPI_ISL_17959214, EPI_ISL_17959215, EPI_ISL_17959216	Tokyo Metropolitan Institute of Public Health	Tokyo Metropolitan Institute of Public Health	Fumi Kasuya, Wakaba Okada, Ryota Kumagai, Sachiko Harada, Arisa Amano, Michiya Hasegawa, Mami Nagashima, Kenji Sadamasu
EPI_ISL_17960864, EPI_ISL_17960865, EPI_ISL_17960866	Laboratorio Nacional de Salud Pública Dr. Defilló	Laboratorio Nacional de Salud Pública Dr. Defilló	Isaac Miguel Sánchez, Carlos Vergara Castillo, Edwin Féliz, Anny Peña, Pedro Martinez, Yeny E. Lara Perez, Robinson Agramonte
EPI_ISL_17972012, EPI_ISL_17972014, EPI_ISL_17972015	California Department of Public Health (CDPH)	California Department of Public Health (CDPH)	Kath,C., Haw,M., Espinosa,A. and Hacker,J.
EPI_ISL_17977751	Centro Medico ABC	Instituto Nacional de Medicina Genomica	Cedro Tanda Alberto, Roxana Trejo Gonzalez, Laura Gomez-Romero, Alfredo Mendoza-Vargas, Dora Garnica-Lopez, Alfredo Hidalgo-Miranda.
EPI_ISL_17979000, EPI_ISL_17979001, EPI_ISL_17979002	Quest Diagnostics	Regional Innovative Public Health Laboratory at Rush University Medical Center	Stefan Green, Kevin Kunstman, Hannah Barbian, Sofiya Bobrovska, Felix Araujo Perez, Edith Perez, Cecilia Chau, Giancarlo Balangue, Lok Yiu Ashley Wu, Trisha Jeon, Marisol Dominguez, Latifah Boyd
EPI_ISL_17980808	Hospital Ramon y Cajal	Hospital Ramon y Cajal	Ponce-Alonso,M., Martinez-Garcia,L., Olavarrieta,L. and Galan,J.C.
EPI_ISL_17988349, EPI_ISL_17988350, EPI_ISL_17988351, EPI_ISL_17988352, EPI_ISL_17988353, EPI_ISL_17988354, EPI_ISL_17988355, EPI_ISL_17988356, EPI_ISL_17988357, EPI_ISL_17988358, EPI_ISL_17988359, EPI_ISL_17988360, EPI_ISL_17988361, EPI_ISL_17988362, EPI_ISL_17988363, EPI_ISL_17988364, EPI_ISL_17988365, EPI_ISL_17988366, EPI_ISL_17988367, EPI_ISL_17988368, EPI_ISL_17988369, EPI_ISL_17988370, EPI_ISL_17988371, EPI_ISL_17988372, EPI_ISL_17988373, EPI_ISL_17988374, EPI_ISL_17988375, EPI_ISL_17988376, EPI_ISL_17988377, EPI_ISL_17988378			
see above	Laboratorio Central de Salud Publica	Laboratorio Central de Salud Publica	Cynthia Vazquez, Vagner Fonseca, Andrea Gomez de la Fuente, Sandra Gonzalez, Fatima Fleitas, Mauricio Lima, Natalia R. Guimaraes, Felipe C. M. Iani, Analia Rojas, Tania Alfonso, Cesar Cantero, Julio Barrios, Shirley Villalba, Maria Jose Ortega, Juan Torales, Maria Liz Gamarra, Carolina Aquino, Jairo Mendez Rico, Luiz Carlos Junior Alcantara, Marta Giovanetti
EPI_ISL_18044983, EPI_ISL_18044987	Center for Vectors and Infectious Diseases Research (CEVDI), National Health Institute Doutor Ricardo Jorge, IP (INSA),	Center for Vectors and Infectious Diseases Research (CEVDI), National Health Institute Doutor Ricardo Jorge, IP (INSA),	Isidro,J., Borges,V., Pinto,M., Sobral,D., Santos,J., Nunes,A., Mixao,V., Ferreira,R., Santos,D., Duarte,S., Vieira,L., Borrego,M.J., Nuncio,S., Lopes de Carvalho,I., Pelerito,A., Cordeiro,R. and Gomes,J.P.
EPI_ISL_18055899, EPI_ISL_18055900	Tokyo Metropolitan Institute of Public Health	Tokyo Metropolitan Institute of Public Health	Fumi Kasuya, Wakaba Okada, Ryota Kumagai, Sachiko Harada, Arisa Amano, Michiya Hasegawa, Mami Nagashima, Kenji Sadamasu
EPI_ISL_18059182, EPI_ISL_18059183, EPI_ISL_18059184	Department of Acute Infectious Diseases Control and Prevention, Yunnan Center for Disease Control and Prevention	Department of Acute Infectious Diseases Control and Prevention, Yunnan Center for Disease Control and Prevention	Meiling Zhang, Ruize Ni, Xiaoqing Fu

EPI_ISL_18064640, EPI_ISL_18064641, EPI_ISL_18064642, EPI_ISL_18064643, EPI_ISL_18064644, EPI_ISL_18064645, EPI_ISL_18064648	Laboratory of Microbiology and Virology, Ospedale Amedeo di Savoia, ASL "Città di Torino"	Laboratory of Microbiology and Virology, Ospedale Amedeo di Savoia, ASL "Città di Torino"	Francesco Cerutti, Tiziano Allice, Maria Grazia Milia, Gabriella Gregori, Elisa Burdino, Sara Monteleone, Marisa Cazzadore, Valeria Ghisetti
EPI_ISL_18075506, EPI_ISL_18075507, EPI_ISL_18075508	Tokyo Metropolitan Institute of Public Health	Tokyo Metropolitan Institute of Public Health	Fumi Kasuya, Wakaba Okada, Ryota Kumagai, Sachiko Harada, Arisa Amano, Michiya Hasegawa, Mami Nagashima, Kenji Sadamasu
EPI_ISL_18076380, EPI_ISL_18076383, EPI_ISL_18076384, EPI_ISL_18076389	Center for Vectors and Infectious Diseases Research (CEVDI), National Health Institute Doutor Ricardo Jorge, IP (INSA)	Center for Vectors and Infectious Diseases Research (CEVDI), National Health Institute Doutor Ricardo Jorge, IP (INSA)	Isidro,J., Borges,V., Pinto,M., Sobral,D., Santos,J., Nunes,A., Mixao,V., Ferreira,R., Santos,D., Duarte,S., Vieira,L., Borrego,M.J., Nuncio,S., Lopes de Carvalho,I., Pelerito,A., Cordeiro,R. and Gomes,J.P.
EPI_ISL_18097375	Tokyo Metropolitan Institute of Public Health	Tokyo Metropolitan Institute of Public Health	Fumi Kasuya, Wakaba Okada, Ryota Kumagai, Sachiko Harada, Arisa Amano, Michiya Hasegawa, Mami Nagashima, Kenji Sadamasu
EPI_ISL_18128768	Bichat-Claude Bernard Hospital, Paris France	Institut Pasteur	Aurelia Kwasiborski, Véronique Hourdel, Charlotte Balière, Damien Hoinard, Quentin Grassin, Maxence Feher, Clémentine De La Porte Des Vaux, Mélanie Cresta, Jessica Vanhomwegen, Jean-Claude Manuguerra, Christophe Batéjat, Valérie Caro
EPI_ISL_18137801, EPI_ISL_18137802, EPI_ISL_18137803	Northwestern Medicine	RIPHL at Rush University Medical Center	Stefan Green, Kevin Kunstman, Hannah Barbian, Sofiya Bobrovska, Felix Araujo Perez, Edith Perez, Cecilia Chau, Giancarlo Balangue, Lok Yiu Ashley Wu, Trisha Jeon, Marisol Dominguez, Latifah Boyd, Lacy Simons
EPI_ISL_18137805	ACL Laboratories	RIPHL at Rush University Medical Center	Stefan Green, Kevin Kunstman, Hannah Barbian, Sofiya Bobrovska, Felix Araujo Perez, Edith Perez, Cecilia Chau, Giancarlo Balangue, Lok Yiu Ashley Wu, Trisha Jeon, Marisol Dominguez, Latifah Boyd
EPI_ISL_18137807, EPI_ISL_18137808	Northwestern Medicine	RIPHL at Rush University Medical Center	Stefan Green, Kevin Kunstman, Hannah Barbian, Sofiya Bobrovska, Felix Araujo Perez, Edith Perez, Cecilia Chau, Giancarlo Balangue, Lok Yiu Ashley Wu, Trisha Jeon, Marisol Dominguez, Latifah Boyd, Lacy Simons
EPI_ISL_18137812	Quest Diagnostics	RIPHL at Rush University Medical Center	Stefan Green, Kevin Kunstman, Hannah Barbian, Sofiya Bobrovska, Felix Araujo Perez, Edith Perez, Cecilia Chau, Giancarlo Balangue, Lok Yiu Ashley Wu, Trisha Jeon, Marisol Dominguez, Latifah Boyd
EPI_ISL_18137814, EPI_ISL_18137815, EPI_ISL_18137816, EPI_ISL_18137817, EPI_ISL_18137818, EPI_ISL_18137819, EPI_ISL_18137820	Northwestern Medicine	RIPHL at Rush University Medical Center	Stefan Green, Kevin Kunstman, Hannah Barbian, Sofiya Bobrovska, Felix Araujo Perez, Edith Perez, Cecilia Chau, Giancarlo Balangue, Lok Yiu Ashley Wu, Trisha Jeon, Marisol Dominguez, Latifah Boyd, Lacy Simons
EPI_ISL_18147334, EPI_ISL_18147335, EPI_ISL_18147336, EPI_ISL_18147337, EPI_ISL_18147338, EPI_ISL_18147339, EPI_ISL_18147340, EPI_ISL_18147341, EPI_ISL_18147342, EPI_ISL_18147343, EPI_ISL_18147344, EPI_ISL_18147345, EPI_ISL_18147346, EPI_ISL_18147347, EPI_ISL_18147348, EPI_ISL_18147349, EPI_ISL_18147350, EPI_ISL_18147351, EPI_ISL_18147352, EPI_ISL_18147353, EPI_ISL_18147354, EPI_ISL_18147355, EPI_ISL_18147356, EPI_ISL_18147357, EPI_ISL_18147358, EPI_ISL_18147359, EPI_ISL_18147360, EPI_ISL_18147361, EPI_ISL_18147362, EPI_ISL_18147363			
see above	Korea Disease Control and Prevention Agency	Korea Disease Control and Prevention Agency	Chung,Y.-S., Yi,H., Choi,M.-M., Kim,J.-W., Lee,M., Lee,S., Sim,G., Lee,J.H., Shin,H. and Choi,C.
EPI_ISL_18161269, EPI_ISL_18161271, EPI_ISL_18161276, EPI_ISL_18161279	Quest Diagnostics Nichols Institute	Los Angeles County Public Health Laboratories	J. Garrigues et al.
EPI_ISL_18161289, EPI_ISL_18161291	Los Angeles County Public Health Laboratories	Los Angeles County Public Health Laboratories	J. Garrigues et al.
EPI_ISL_18161297, EPI_ISL_18161301, EPI_ISL_18161303, EPI_ISL_18161304	Quest Diagnostics Nichols Institute	Los Angeles County Public Health Laboratories	J. Garrigues et al.
EPI_ISL_18161306	Los Angeles County Public Health Laboratories	Los Angeles County Public Health Laboratories	J. Garrigues et al.
EPI_ISL_18161312	Quest Diagnostics Nichols Institute	Los Angeles County Public Health Laboratories	J. Garrigues et al.
EPI_ISL_18161320	Laboratory Corporation of America	Los Angeles County Public Health Laboratories	J. Garrigues et al.
EPI_ISL_18168623	Quest Diagnostics	RIPHL at Rush University Medical Center	Stefan Green, Kevin Kunstman, Hannah Barbian, Sofiya Bobrovska, Felix Araujo Perez, Edith Perez, Cecilia Chau, Giancarlo Balangue, Lok Yiu Ashley Wu, Trisha Jeon, Marisol Dominguez, Latifah Boyd
EPI_ISL_18213374, EPI_ISL_18213375	Institute for Hepatology,Shenzhen Third People's Hospital	Institute for Hepatology,Shenzhen Third People's Hospital	Lin Cheng,Zheng Zhang
EPI_ISL_18228621, EPI_ISL_18228630, EPI_ISL_18228631, EPI_ISL_18228633, EPI_ISL_18228634, EPI_ISL_18228637, EPI_ISL_18228640, EPI_ISL_18228645	Center for Vectors and Infectious Diseases Research (CEVDI), National Health Institute Doutor Ricardo Jorge, IP (INSA)	Center for Vectors and Infectious Diseases Research (CEVDI), National Health Institute Doutor Ricardo Jorge, IP (INSA)	Isidro,J., Borges,V., Pinto,M., Sobral,D., Santos,J., Nunes,A., Mixao,V., Ferreira,R., Santos,D., Duarte,S., Vieira,L., Borrego,M.J., Nuncio,S., Lopes de Carvalho,I., Pelerito,A., Cordeiro,R. and Gomes,J.P.
EPI_ISL_18241786, EPI_ISL_18241788, EPI_ISL_18241789, EPI_ISL_18241790, EPI_ISL_18241791	Unidade de Genômica - UFRJ	Unidade de Genômica - UFRJ	Carolina Moreira Voloch, Filipe Romero Rebello Moreira, Diana Mariani, Rafael Mello Galliez, Debora Souza Faffe, Terezinha Marta Pereira Pinto Castiñeiras, Clarissa Damaso, Amílcar Tanuri.
EPI_ISL_18245407, EPI_ISL_18245408, EPI_ISL_18245409, EPI_ISL_18245410, EPI_ISL_18245411, EPI_ISL_18245412, EPI_ISL_18245413	Indian Council of Medical Research-National Institute of Virology	Indian Council of Medical Research-National Institute of Virology	Pragya Yadav, Rima Sahay, Anita Aich Shete, Sreelekshmy Mohandas
EPI_ISL_18285962, EPI_ISL_18285963, EPI_ISL_18285965, EPI_ISL_18285966	Quest Diagnostics Nichols Institute	Los Angeles County Public Health Laboratories	J. Garrigues et al.
EPI_ISL_18285971, EPI_ISL_18285972	Cedars-Sinai Medical Center	Los Angeles County Public Health Laboratories	J. Garrigues et al.
EPI_ISL_18285985	Quest Diagnostics	RIPHL at Rush University Medical Center	Stefan Green, Kevin Kunstman, Hannah Barbian, Sofiya Bobrovska, Felix Araujo Perez, Edith Perez, Cecilia Chau, Giancarlo Balangue, Lok Yiu Ashley Wu, Trisha Jeon, Marisol Dominguez, Latifah Boyd
EPI_ISL_18308395, EPI_ISL_18308396, EPI_ISL_18308397, EPI_ISL_18308398, EPI_ISL_18308399	National Virus Reference Laboratory	National Virus Reference Laboratory	Gabriel Gonzalez, Michael Carr, Emer O'Byrne, Weronika Banka, Brian Keogan, Jonathan Dean, Daniel Hare, Cillian F De Gascun
EPI_ISL_18323779, EPI_ISL_18323780, EPI_ISL_18323781, EPI_ISL_18323784, EPI_ISL_18323786, EPI_ISL_18323794, EPI_ISL_18324980, EPI_ISL_18324981, EPI_ISL_18324984, EPI_ISL_18324989, EPI_ISL_18324992, EPI_ISL_18324993, EPI_ISL_18324994, EPI_ISL_18324995, EPI_ISL_18324996, EPI_ISL_18324997, EPI_ISL_18324998, EPI_ISL_18324999, EPI_ISL_18325000, EPI_ISL_18325003, EPI_ISL_18325007, EPI_ISL_18325008, EPI_ISL_18325010			
see above	California Department of Public Health	California Department of Public Health	Kath, C., Haw, M., Espinosa, A., and Hacker, J.
EPI_ISL_18352302, EPI_ISL_18352303, EPI_ISL_18352304, EPI_ISL_18352305, EPI_ISL_18352306	Tokyo Metropolitan Institute of Public Health	Tokyo Metropolitan Institute of Public Health	Fumi Kasuya, Wakaba Okada, Ryota Kumagai, Sachiko Harada, Arisa Amano, Michiya Hasegawa, Mami Nagashima, Kenji Sadamasu
EPI_ISL_18354483	Shenzhen Key Laboratory of Pathogen and Immunity	Shenzhen Key Laboratory of Pathogen and Immunity	Yang Yang, Shengjie Zhang, Yun Peng, Fuxiang Wang, Yingxia Liu, Hongzhou Lu
EPI_ISL_18386999, EPI_ISL_18387001, EPI_ISL_18387005, EPI_ISL_18387010, EPI_ISL_18387011	NC - Los Angeles County Public Health Laboratories	NC - Los Angeles County Public Health Laboratories	Garrigues,J.M. and Green,N.M.
EPI_ISL_18399136, EPI_ISL_18399142, EPI_ISL_18399143, EPI_ISL_18399144, EPI_ISL_18399145	California Department of Public Health	California Department of Public Health	Kath, C., Haw, M., Espinosa, A., and Hacker, J.
EPI_ISL_18436040	PKC Mampang Prapatan	National Institute of Health Research and Development	Fajar Nur Sulistyahadi, Arie Ardiansyah Nugraha, Hana Apsari Pawestri, Kartika Dewi Puspa, Herna, Subangkit, IGM Wirabrata
EPI_ISL_18436041	PKC Jatinegara	National Institute of Health Research and Development	Fajar Nur Sulistyahadi, Arie Ardiansyah Nugraha, Hana Apsari Pawestri, Kartika Dewi Puspa, Herna, Subangkit, IGM Wirabrata
EPI_ISL_18443030, EPI_ISL_18443032, EPI_ISL_18443033, EPI_ISL_18443035, EPI_ISL_18443036, EPI_ISL_18443038, EPI_ISL_18443040, EPI_ISL_18443041, EPI_ISL_18443042, EPI_ISL_18443045, EPI_ISL_18452334, EPI_ISL_18452335, EPI_ISL_18452336, EPI_ISL_18452337, EPI_ISL_18452341, EPI_ISL_18452343, EPI_ISL_18452347, EPI_ISL_18458948, EPI_ISL_18458949, EPI_ISL_18458952, EPI_ISL_18458953, EPI_ISL_18458955, EPI_ISL_18458956, EPI_ISL_18458957, EPI_ISL_18458958, EPI_ISL_18458959, EPI_ISL_18458960, EPI_ISL_18458961, EPI_ISL_18458962, EPI_ISL_18458963, EPI_ISL_18458964, EPI_ISL_18458965, EPI_ISL_18460494, EPI_ISL_18460495, EPI_ISL_18460496, EPI_ISL_18460497, EPI_ISL_18460498, EPI_ISL_18460499, EPI_ISL_18460500, EPI_ISL_18460501, EPI_ISL_18460502, EPI_ISL_18460504, EPI_ISL_18460505			
see above	California Department of Public Health	California Department of Public Health	Kath, C., Haw, M., Espinosa, A., and Hacker, J.
EPI_ISL_18463158	PKM Kembangan	National Institute of Health Research and Development	Hana Apsari Pawestri, Arie Ardiansyah Nugraha, Fajar Nur Sulistyahadi, Hartanti Dian Ikawati, Kartika Dewi Puspa, Markus Evan Anggia, Subangkit, Nelis Imaningsih, IGM Wirabrata
EPI_ISL_18463159	PKC Setiabudi	National Institute of Health Research and Development	Hana Apsari Pawestri, Arie Ardiansyah Nugraha, Fajar Nur Sulistyahadi, Hartanti Dian Ikawati, Kartika Dewi Puspa, Markus Evan Anggia, Subangkit, Nelis Imaningsih, IGM Wirabrata
EPI_ISL_18463160	RSUPN Dr Cipto Mangunkusumo	National Institute of Health Research and Development	Hana Apsari Pawestri, Arie Ardiansyah Nugraha, Fajar Nur Sulistyahadi, Hartanti Dian Ikawati, Kartika Dewi Puspa, Markus Evan Anggia, Subangkit, Nelis Imaningsih, IGM Wirabrata
EPI_ISL_18463161	RSUD Kembangan	National Institute of Health Research and Development	Hana Apsari Pawestri, Arie Ardiansyah Nugraha, Fajar Nur Sulistyahadi, Hartanti Dian Ikawati, Kartika Dewi Puspa, Markus Evan Anggia, Subangkit, Nelis Imaningsih, IGM Wirabrata
EPI_ISL_18467794	Eka Hospital BSD	National Institute of Health Research and Development	Fajar Nur Sulistyahadi, Hana Apsari Pawestri, Arie Ardiansyah Nugraha, Hartanti Dian Ikawati, Kartika Dewi Puspa, Subangkit, IGM Wirabrata
EPI_ISL_18467795, EPI_ISL_18467796	PKM Kembangan	National Institute of Health Research and Development	Hana Apsari Pawestri, Arie Ardiansyah Nugraha, Fajar Nur Sulistyahadi, Hartanti Dian Ikawati, Kartika Dewi Puspa, Subangkit, IGM Wirabrata
EPI_ISL_18467797	PKC Cengkareng	National Institute of Health Research and Development	Arie Ardiansyah Nugraha, Fajar Nur Sulistyahadi, Hartanti Dian Ikawati, Kartika Dewi Puspa, Hana Apsari Pawestri, Subangkit, IGM Wirabrata
EPI_ISL_18467798	PKC Grogol Petamburan	National Institute of Health Research and Development	Fajar Nur Sulistyahadi, Hana Apsari Pawestri, Arie Ardiansyah Nugraha, Hartanti Dian Ikawati, Kartika Dewi Puspa, Subangkit, IGM Wirabrata

EPI_ISL_18467799	PKC Setiabudi	National Institute of Health Research and Development	Hana Apsari Pawestri, Arie Ardiansyah Nugraha, Fajar Nur Sulistyahadi, Hartanti Dian Ikawati, Kartika Dewi Puspa, Subangkit, IGM Wirabrata
EPI_ISL_18467800	PKC Pancoran	National Institute of Health Research and Development	Arie Ardiansyah Nugraha, Fajar Nur Sulistyahadi, Hartanti Dian Ikawati, Kartika Dewi Puspa, Hana Apsari Pawestri, Subangkit, IGM Wirabrata
EPI_ISL_18467801	PKM Mampang Prapatan	National Institute of Health Research and Development	Arie Ardiansyah Nugraha, Fajar Nur Sulistyahadi, Hartanti Dian Ikawati, Kartika Dewi Puspa, Hana Apsari Pawestri, Subangkit, IGM Wirabrata
EPI_ISL_18467803	RS Brawijaya Saharjo	National Institute of Health Research and Development	Fajar Nur Sulistyahadi, Hana Apsari Pawestri, Arie Ardiansyah Nugraha, Hartanti Dian Ikawati, Kartika Dewi Puspa, Subangkit, IGM Wirabrata
EPI_ISL_18467805	PKC Kramat Jati	National Institute of Health Research and Development	Arie Ardiansyah Nugraha, Fajar Nur Sulistyahadi, Hartanti Dian Ikawati, Kartika Dewi Puspa, Hana Apsari Pawestri, Subangkit, IGM Wirabrata
EPI_ISL_18467806	PKM Tanjung Priuk	National Institute of Health Research and Development	Fajar Nur Sulistyahadi, Hana Apsari Pawestri, Arie Ardiansyah Nugraha, Hartanti Dian Ikawati, Kartika Dewi Puspa, Subangkit, IGM Wirabrata
EPI_ISL_18467807	PKC Kelapa Gading	National Institute of Health Research and Development	Fajar Nur Sulistyahadi, Hana Apsari Pawestri, Arie Ardiansyah Nugraha, Hartanti Dian Ikawati, Kartika Dewi Puspa, Subangkit, IGM Wirabrata
EPI_ISL_18467808	RSUP Dr Hasan Sadikin	National Institute of Health Research and Development	Arie Ardiansyah Nugraha, Fajar Nur Sulistyahadi, Hartanti Dian Ikawati, Kartika Dewi Puspa, Hana Apsari Pawestri, Subangkit, IGM Wirabrata
EPI_ISL_18486349, EPI_ISL_18486350, EPI_ISL_18486351, EPI_ISL_18486352	U.O. Microbiologia Laboratorio Unico Centro Servizi - Azienda Unità Sanitarie Locali della Romagna	U.O. Microbiologia Laboratorio Unico Centro Servizi - Azienda Unità Sanitarie Locali della Romagna - DIMEC - Università di Bologna	Alessandra Scagliarini, Vittorio Sambri, Maria Elena Turba, Fabio Gentilini, Francesca Taddei, Giorgio Dirani, Silvia Zannoli, Giulia Gatti, Martina Brandolini, Alessandra Mistral De Pascali, Monica Cricca
EPI_ISL_18539018	Microbiology and Laboratory Science, Public Health Ontario	Microbiology and Laboratory Science, Public Health Ontario	Isabel,S., Eshaghi,A., Duvvuri,V.R., Gubbay,J.B., Cronin,K., Li,A., Hasso,M., Clark,S.T., Hopkins,J.P., Patel,S.N. and Braukmann,T.W.A.
EPI_ISL_18553811, EPI_ISL_18553812, EPI_ISL_18553813, EPI_ISL_18553816, EPI_ISL_18553820, EPI_ISL_18553824, EPI_ISL_18553826, EPI_ISL_18553829, EPI_ISL_18553832, EPI_ISL_18553833, EPI_ISL_18553835, EPI_ISL_18553837, EPI_ISL_18553846, EPI_ISL_18553847, EPI_ISL_18553850			
see above	Center for Vectors and Infectious Diseases Research (CEVDI), National Health Institute Doutor Ricardo Jorge, IP (INSA)	Center for Vectors and Infectious Diseases Research (CEVDI), National Health Institute Doutor Ricardo Jorge, IP (INSA)	Isidro,J., Borges,V., Pinto,M., Sobral,D., Santos,J., Nunes,A., Mixao,V., Ferreira,R., Santos,D., Duarte,S., Vieira,L., Borrego,M.J., Nuncio,S., Lopes de Carvalho,I., Pelerito,A., Cordeiro,R. and Gomes,J.P.
EPI_ISL_18557816, EPI_ISL_18557817, EPI_ISL_18557818	Tokyo Metropolitan Institute of Public Health	Tokyo Metropolitan Institute of Public Health	Fumi Kasuya, Wakaba Okada, Ryota Kumagai, Sachiko Harada, Arisa Amano, Michiya Hasegawa, Mami Nagashima, Kenji Sadamasu
EPI_ISL_18567806, EPI_ISL_18567807	Southern Nevada Public Health Laboratory	Southern Nevada Public Health Laboratory	Horng-Yuan Kan
EPI_ISL_18627266	Division of Infectious Diseases and Tropical Medicine, University Hospital, Ludwig-Maximilians-Universitaet (LMU) Munich, Munich, Germany	Bundeswehr Institute of Microbiology	MH Antwerpen, D Lang, S Zange, R Woelfel
EPI_ISL_18642356	PKC Senen	National Institute of Health Research and Development	Hana Apsari Pawestri, Arie Ardiansyah Nugraha, Fajar Nur Sulistyahadi, Markus Evan Anggia, Subangkit, Herna, IGM Wirabrata
EPI_ISL_18642357	PKC Cakung	National Institute of Health Research and Development	Hana Apsari Pawestri, Arie Ardiansyah Nugraha, Fajar Nur Sulistyahadi, Markus Evan Anggia, Subangkit, Herna, IGM Wirabrata
EPI_ISL_18642358	RS Mitra Keluarga Gading	National Institute of Health Research and Development	Hana Apsari Pawestri, Arie Ardiansyah Nugraha, Fajar Nur Sulistyahadi, Markus Evan Anggia, Subangkit, Herna, IGM Wirabrata
EPI_ISL_18642359	RSUP Persahabatan	National Institute of Health Research and Development	Hana Apsari Pawestri, Arie Ardiansyah Nugraha, Fajar Nur Sulistyahadi, Markus Evan Anggia, Subangkit, Herna, IGM Wirabrata
EPI_ISL_18642360	PKC Cilandak	National Institute of Health Research and Development	Hana Apsari Pawestri, Arie Ardiansyah Nugraha, Fajar Nur Sulistyahadi, Markus Evan Anggia, Subangkit, Herna, IGM Wirabrata
EPI_ISL_18642361	PKC Mampang Prapatan	National Institute of Health Research and Development	Hana Apsari Pawestri, Arie Ardiansyah Nugraha, Fajar Nur Sulistyahadi, Markus Evan Anggia, Subangkit, Herna, IGM Wirabrata
EPI_ISL_18642362	Dinkes Kabupaten Cirebon	National Institute of Health Research and Development	Hana Apsari Pawestri, Arie Ardiansyah Nugraha, Fajar Nur Sulistyahadi, Markus Evan Anggia, Subangkit, Herna, IGM Wirabrata
EPI_ISL_18642363	PKC Setiabudi	National Institute of Health Research and Development	Hana Apsari Pawestri, Arie Ardiansyah Nugraha, Fajar Nur Sulistyahadi, Markus Evan Anggia, Subangkit, Herna, IGM Wirabrata
EPI_ISL_18642364	PKM Pancoran	National Institute of Health Research and Development	Hana Apsari Pawestri, Arie Ardiansyah Nugraha, Fajar Nur Sulistyahadi, Markus Evan Anggia, Subangkit, Herna, IGM Wirabrata
EPI_ISL_18642365	Puskesmas Bambu Apus	National Institute of Health Research and Development	Hana Apsari Pawestri, Arie Ardiansyah Nugraha, Fajar Nur Sulistyahadi, Markus Evan Anggia, Subangkit, Herna, IGM Wirabrata
EPI_ISL_18659828	Erasmus Medical Center Department of Virology	Erasmus Medical Center Department of Virology	Leonard Schuele, Bas Oude Munnink, Marjan Boter, Babette Weller, Babs Verstrepen, Richard Molenkamp, Reina Sikkema, Marion Koopmans
EPI_ISL_18659829, EPI_ISL_18659846	Erasmus Medical Center Department of Virology	Erasmus Medical Center Department of Virology	Leonard Schuele, Marjan Boter, Hayley Cassidy, Babette Weller, Babs Verstrepen, Richard Molenkamp, Marion Koopmans, Bas Oude Munnink
EPI_ISL_18689511	Bundeswehr Institute of Microbiology	Bundeswehr Institute of Microbiology	Antwerpen,M.H., Lang,D., Sabine,S. and Woelfel,R.
EPI_ISL_18697752	National Institute of Public Health	Institut Pasteur du Cambodge, Virology Unit	Janin Nouhin, Leakhena Pum, Jurre Y Siegers, Chin Savuth, Chau Darapheak, Veasna Duong, Erik A Karlsson
EPI_ISL_18702208, EPI_ISL_18702209, EPI_ISL_18702212, EPI_ISL_18702213, EPI_ISL_18702219, EPI_ISL_18702221, EPI_ISL_18702222, EPI_ISL_18702223, EPI_ISL_18702224, EPI_ISL_18702225, EPI_ISL_18702226, EPI_ISL_18702227, EPI_ISL_18702229, EPI_ISL_18702230, EPI_ISL_18702231, EPI_ISL_18702234, EPI_ISL_18702235, EPI_ISL_18702236, EPI_ISL_18702237, EPI_ISL_18702238, EPI_ISL_18702240, EPI_ISL_18702241, EPI_ISL_18702243, EPI_ISL_18702247, EPI_ISL_18702248, EPI_ISL_18702249, EPI_ISL_18702250, EPI_ISL_18702251, EPI_ISL_18702252, EPI_ISL_18702253, EPI_ISL_18702255, EPI_ISL_18702256, EPI_ISL_18702257			
see above	Center for Vectors and Infectious Diseases Research (CEVDI), National Health Institute Doutor Ricardo Jorge, IP (INSA)	Center for Vectors and Infectious Diseases Research (CEVDI), National Health Institute Doutor Ricardo Jorge, IP (INSA)	Isidro,J., Borges,V., Pinto,M., Sobral,D., Santos,J., Nunes,A., Mixao,V., Ferreira,R., Santos,D., Duarte,S., Vieira,L., Borrego,M., Nuncio,S., Lopes de Carvalho,I., Pelerito,A., Cordeiro,R. and Gomes,J.P.
EPI_ISL_18719999	CT Department of Public Health	CT Department of Public Health	Claire Pearson, Tu N. Nguyen, Kutluhan Incekara, Neranjan V. Perera
EPI_ISL_18722047	Dallas Regional Medical Center	Dallas County Health and Human Services Public Health Laboratory	Kabir, Farruk; Plaisance, Erin; Stringer, Joey; Short, Luke.
EPI_ISL_18722048	DCHHS Sexual Health Clinic	Dallas County Health and Human Services Public Health Laboratory	Kabir, Farruk; Plaisance, Erin; Stringer, Joey; Short, Luke.
EPI_ISL_18722049, EPI_ISL_18722051	Parkland Health and Hospital System	Dallas County Health and Human Services Public Health Laboratory	Kabir, Farruk; Plaisance, Erin; Stringer, Joey; Short, Luke.
EPI_ISL_18723947, EPI_ISL_18723948	DCHHS Sexual Health Clinic	Dallas County Health & Human Services Public Health Laboratory	Kabir, Farruk; Plaisance, Erin; Stringer, Joey; Short, Luke.
EPI_ISL_18723949	Texas Health Presbyterian at Dallas	Dallas County Health & Human Services Public Health Laboratory	Kabir, Farruk; Plaisance, Erin; Stringer, Joey; Short, Luke.
EPI_ISL_18723959, EPI_ISL_18723960, EPI_ISL_18723967, EPI_ISL_18723975, EPI_ISL_18737443, EPI_ISL_18737446, EPI_ISL_18737448, EPI_ISL_18737523, EPI_ISL_18737524, EPI_ISL_18737544, EPI_ISL_18737546, EPI_ISL_18737553			
see above	Parkland Health and Hospital System	Dallas County Health & Human Services Public Health Laboratory	Kabir, Farruk; Plaisance, Erin; Stringer, Joey; Short, Luke.
EPI_ISL_18739594, EPI_ISL_18739595	Tokyo Metropolitan Institute of Public Health	Tokyo Metropolitan Institute of Public Health	Fumi Kasuya, Wakaba Okada, Ryota Kumagai, Sachiko Harada, Arisa Amano, Michiya Hasegawa, Mami Nagashima, Kenji Sadamasu
EPI_ISL_18744048, EPI_ISL_18744049, EPI_ISL_18744050	National Medical Center	National Medical Center	Jun-sun Park, Hongsoon Yim, Jihye Um, Hyang Su Kim, BumSik Chin, Jaehyun Jeon, Yeonjae Kim, Min-Kyung Kim
EPI_ISL_18746884, EPI_ISL_18746885	Erasmus Medical Center Department of Virology	Erasmus Medical Center Department of Virology	Leonard Schuele, Marjan Boter, Babs Verstrepen, Richard Molenkamp, Marion Koopmans, Bas Oude Munnink
EPI_ISL_18746967, EPI_ISL_18746991, EPI_ISL_18746995	Parkland Health and Hospital System	Dallas County Health & Human Services Public Health Laboratory	Kabir, Farruk; Plaisance, Erin; Stringer, Joey; Short, Luke.
EPI_ISL_18746999	DCHHS Sexual Health Clinic	Dallas County Health & Human Services Public Health Laboratory	Kabir, Farruk; Plaisance, Erin; Stringer, Joey; Short, Luke.
EPI_ISL_18747002, EPI_ISL_18747010, EPI_ISL_18747025	Parkland Health and Hospital System	Dallas County Health & Human Services Public Health Laboratory	Kabir, Farruk; Plaisance, Erin; Stringer, Joey; Short, Luke.
EPI_ISL_18755970, EPI_ISL_18755971, EPI_ISL_18755972, EPI_ISL_18755973, EPI_ISL_18755974, EPI_ISL_18755975, EPI_ISL_18755976, EPI_ISL_18755977, EPI_ISL_18755978, EPI_ISL_18755979	Erasmus Medical Center Department of Virology	Erasmus Medical Center Department of Virology	Leonard Schuele, Marjan Boter, Babs Verstrepen, Richard Molenkamp, Marion Koopmans, Bas Oude Munnink

EPI_ISL_18781626, EPI_ISL_18781627, EPI_ISL_18781628, EPI_ISL_18781629, EPI_ISL_18781630, EPI_ISL_18781631, EPI_ISL_18781635, EPI_ISL_18781636, EPI_ISL_18781637, EPI_ISL_18781786, EPI_ISL_18781787, EPI_ISL_18781788, EPI_ISL_18781791, EPI_ISL_18781792, EPI_ISL_18781793, EPI_ISL_18781806, EPI_ISL_18781807, EPI_ISL_18781808, EPI_ISL_18781809, EPI_ISL_18781810, EPI_ISL_18781811, EPI_ISL_18781812, EPI_ISL_18781815, EPI_ISL_18781816, EPI_ISL_18781817, EPI_ISL_18781818, EPI_ISL_18781819, EPI_ISL_18781820, EPI_ISL_18781833, EPI_ISL_18781836, EPI_ISL_18781837, EPI_ISL_18781840, EPI_ISL_18781844, EPI_ISL_18781848, EPI_ISL_18781849, EPI_ISL_18781913, EPI_ISL_18781915, EPI_ISL_18781918, EPI_ISL_18781922, EPI_ISL_18781923, EPI_ISL_18781924, EPI_ISL_18781925, EPI_ISL_18781926, EPI_ISL_18781928, EPI_ISL_18781929, EPI_ISL_18786340, EPI_ISL_18786341, EPI_ISL_18786342, EPI_ISL_18786347, EPI_ISL_18786348, EPI_ISL_18786349, EPI_ISL_18786350, EPI_ISL_18786351, EPI_ISL_18786357, EPI_ISL_18786358			
see above	California Department of Public Health	California Department of Public Health	Kath, C., Haw, M., Espinosa, A., and Hacker, J.
EPI_ISL_18798834	PKM Mampang Prapatan	National Institute of Health Research and Development	Hana Apsari Pawestri, Arie Ardiansyah Nugraha, Fajar Nur Sulistyahadi, Markus Evan Anggia, Subangkit
EPI_ISL_18798835	PKC Kebayoran Lama	National Institute of Health Research and Development	Hana Apsari Pawestri, Arie Ardiansyah Nugraha, Fajar Nur Sulistyahadi, Markus Evan Anggia, Subangkit
EPI_ISL_18798836	RS Grha Kedoya Jakarta	National Institute of Health Research and Development	Hana Apsari Pawestri, Arie Ardiansyah Nugraha, Fajar Nur Sulistyahadi, Markus Evan Anggia, Subangkit
EPI_ISL_18798837	PKC Kebayoran Baru	National Institute of Health Research and Development	Hana Apsari Pawestri, Arie Ardiansyah Nugraha, Fajar Nur Sulistyahadi, Markus Evan Anggia, Subangkit
EPI_ISL_18798838	PKC Tanah Abang	National Institute of Health Research and Development	Hana Apsari Pawestri, Arie Ardiansyah Nugraha, Fajar Nur Sulistyahadi, Markus Evan Anggia, Subangkit
EPI_ISL_18798839	PKM Bogor Timur	National Institute of Health Research and Development	Hana Apsari Pawestri, Arie Ardiansyah Nugraha, Fajar Nur Sulistyahadi, Markus Evan Anggia, Subangkit
EPI_ISL_18798840	PKM Warung Jambu	National Institute of Health Research and Development	Hana Apsari Pawestri, Arie Ardiansyah Nugraha, Fajar Nur Sulistyahadi, Markus Evan Anggia, Subangkit
EPI_ISL_18798841	PKC Pademangan	National Institute of Health Research and Development	Hana Apsari Pawestri, Arie Ardiansyah Nugraha, Fajar Nur Sulistyahadi, Markus Evan Anggia, Subangkit
EPI_ISL_18798842	PKC Kebayoran Baru	National Institute of Health Research and Development	Hana Apsari Pawestri, Arie Ardiansyah Nugraha, Fajar Nur Sulistyahadi, Markus Evan Anggia, Subangkit
EPI_ISL_18809376	California Department of Public Health	California Department of Public Health	Kath, C., Haw, M., Espinosa, A., and Hacker, J.
EPI_ISL_18822108	Quest Diagnostics Nichols Institute	Los Angeles County Public Health Laboratories	S. McCann et al
EPI_ISL_18879931	Central Public Health Laboratories, Ministry of Health Egypt	Center of Scientific Excellence for Influenza Viruses, National Research Centre (NRC), Egypt.	Wael H. Roshdy, Rabeh El-Shesheny, Yassmin Moatasim, Mina N. Kamel, Shaymaa Shawky, Mokhtar Gomaa, Galal Mahmoud, Amer Sayed, Amel Naguib, Nancy El Guindy, Ahmed Kandeil, Mohamed A. Ali, Amr Kandeel
EPI_ISL_18952716	Quest Diagnostics Nichols Institute	Los Angeles County Public Health Laboratory	S. McCann et. al.
EPI_ISL_18959309, EPI_ISL_18959311, EPI_ISL_18959312, EPI_ISL_18959314, EPI_ISL_18959316, EPI_ISL_18959318, EPI_ISL_18959319, EPI_ISL_18959320, EPI_ISL_18959321, EPI_ISL_18959322, EPI_ISL_18959323, EPI_ISL_18959324			
see above	California Department of Public Health	California Department of Public Health	Kath, C., Haw, M., Espinosa, A., and Hacker, J.
EPI_ISL_18971016, EPI_ISL_18971017	Central Public Health Laboratory, State Health Surveillance Center of the Rio Grande do Sul State Health Department (LACEN/CEVS/SES-RS)	Center for Scientific and Technological Development, State Center for Health Surveillance of the Secretary of Health of the State of Rio Grande do Sul (CDCT/CEVS/SES-RS)	Fernanda Godinho, Richard Steiner Salvato
EPI_ISL_18993187, EPI_ISL_18993188, EPI_ISL_18993191	Quest Diagnostics Nichols Institute	Los Angeles County Public Health Laboratories	J. Garrigues et. al.
EPI_ISL_18993192, EPI_ISL_18993195	Laboratory Corporation Of America	Los Angeles County Public Health Laboratories	J. Garrigues et. al.
EPI_ISL_18993197, EPI_ISL_18993198	Los Angeles County Public Health Laboratory	Los Angeles County Public Health Laboratories	J. Garrigues et. al.
EPI_ISL_18993199, EPI_ISL_18993200	Quest Diagnostics Nichols Institute	Los Angeles County Public Health Laboratories	J. Garrigues et. al.
EPI_ISL_18993201	Los Angeles County Public Health Laboratory	Los Angeles County Public Health Laboratories	J. Garrigues et. al.
EPI_ISL_18993203	Quest Diagnostics Nichols Institute	Los Angeles County Public Health Laboratories	J. Garrigues et. al.
EPI_ISL_18993204	Laboratory Corporation Of America	Los Angeles County Public Health Laboratories	J. Garrigues et. al.
EPI_ISL_18993206	Quest Diagnostics West Hills	Los Angeles County Public Health Laboratories	J. Garrigues et. al.
EPI_ISL_18993207	Laboratory Corporation Of America	Los Angeles County Public Health Laboratories	J. Garrigues et. al.
EPI_ISL_18993210	Arup Laboratories	Los Angeles County Public Health Laboratories	J. Garrigues et. al.
EPI_ISL_18993214	Quest Diagnostics Nichols Institute	Los Angeles County Public Health Laboratories	J. Garrigues et. al.
EPI_ISL_18993959, EPI_ISL_18993961, EPI_ISL_18993964, EPI_ISL_18993966, EPI_ISL_18993967	Center for Vectors and Infectious Diseases Research (CEVDI), National Health Institute Doutor Ricardo Jorge, IP (INSA)	Center for Vectors and Infectious Diseases Research (CEVDI), National Health Institute Doutor Ricardo Jorge, IP (INSA)	Isidro,J., Borges,V., Pinto,M., Sobral,D., Santos,J., Nunes,A., Mixao,V., Ferreira,R., Santos,D., Duarte,S., Vieira,L., Borrego,M.J., Nuncio,S., Lopes de Carvalho,I., Pelerito,A., Cordeiro,R. and Gomes,J.P.
EPI_ISL_19001887	Tokyo Metropolitan Institute of Public Health	Tokyo Metropolitan Institute of Public Health	Fumi Kasuya, Wakaba Okada, Ryota Kumagai, Sachiko Harada, Arisa Amano, Michiya Hasegawa, Mami Nagashima, Kenji Sadamasu
EPI_ISL_19002300	Erasmus Medical Center Department of Virology	Erasmus Medical Center Department of Virology	Leonard Schuele, Marjan Boter, Babs Verstrepen, Richard Molenkamp, Marion Koopmans, Bas Oude Munnink
EPI_ISL_19012435, EPI_ISL_19012436	Laboratorio de Enterovirus, Instituto Oswaldo Cruz, Fiocruz	Oswaldo Cruz Foundation Laboratory of Respiratory Virus and Measles	Paola Resende, Elisa Cavalcante Pereira, Bruna Mendonça da Silva, Jéssica Graça Macedo de Carvalho, Larissa Macedo Pinto, Victor Guimaraes, Marilda Siqueira, Renan da Silva Faustino, Marilia Santini, Edson Elias da Silva on behalf of the Fiocruz Genomic Surveillance Network
EPI_ISL_19016746, EPI_ISL_19016764, EPI_ISL_19016768	Quest Diagnostics	RIPHL at Rush University Medical Center	Stefan Green, Kevin Kunstman, Hannah Barbian, Sofiya Bobrovska, Felix Araujo Perez, Erin Newcomer
EPI_ISL_19016819	Quest Diagnostics	Regional Innovative Public Health Laboratory (RIPHL) at Rush University Medical Center	Stefan Green, Kevin Kunstman, Hannah Barbian, Sofiya Bobrovska, Felix Araujo Perez, Erin Newcomer
EPI_ISL_19022858, EPI_ISL_19022859, EPI_ISL_19022860, EPI_ISL_19022861, EPI_ISL_19022862	Osaka Metropolitan University, Graduate School of Medicine, Department of Virology and Parasitology	Osaka Metropolitan University, Graduate School of Medicine, Department of Virology and Parasitology	Evariste Tshibangu-Kabamba, Natsuko Kaku, Eisuke Adachi, Mayo Yasugi, Takuya Yamamoto, Takuto Nogimori, Yoshiyuki Wakabayashi, Yasutoshi Kido
EPI_ISL_19031631, EPI_ISL_19031633, EPI_ISL_19031635, EPI_ISL_19031636, EPI_ISL_19031637	Quest Diagnostics	RIPHL at Rush University Medical Center	Stefan Green, Kevin Kunstman, Hannah Barbian, Sofiya Bobrovska, Felix Araujo Perez, Erin Newcomer, Alyse Kitttner
EPI_ISL_19053766	Laboratory Medicine and Pathology, University of Washington	Laboratory Medicine and Pathology, University of Washington	Roychoudhury,P., Xie,X., Sereewit,J., Ellis,S. and Greninger,A.
EPI_ISL_19058864, EPI_ISL_19058865, EPI_ISL_19058866, EPI_ISL_19058867, EPI_ISL_19058868, EPI_ISL_19058869, EPI_ISL_19058870, EPI_ISL_19058871, EPI_ISL_19058872, EPI_ISL_19058873	Guangdong Provincial Center for Disease Control and Prevention, Institute of Pathogenic Microbiology	Guangdong Provincial Center for Disease Control and Prevention, Institute of Pathogenic Microbiology	Li,B., Zhao,W. and Shen,C.
EPI_ISL_19093415	ARUP Laboratories	Los Angeles County Public Health Laboratories	N. Heibeck et al.
EPI_ISL_19093789, EPI_ISL_19093793, EPI_ISL_19093798, EPI_ISL_19093801, EPI_ISL_19093802, EPI_ISL_19093804, EPI_ISL_19093806, EPI_ISL_19093807, EPI_ISL_19093813, EPI_ISL_19093818, EPI_ISL_19093819, EPI_ISL_19093820, EPI_ISL_19093822, EPI_ISL_19093823, EPI_ISL_19093825, EPI_ISL_19093826, EPI_ISL_19093828, EPI_ISL_19093829, EPI_ISL_19093832			
see above	Pathogen Genomic Laboratory, Institut National de Recherche Biomedicale	Pathogen Genomic Laboratory, Institut National de Recherche Biomedicale	Vakaniaki,E.H., Kaciat,C., Kinganda - Lusamaki,E., O'Toole,A., Wawina -Bokalanga,T., Mukadi - Bamuleka,D., Amuri,A.A., Parker,E., Muswamba-Kayembe,P.-C., Makangara - Cigolo,J.-C., Mulopo - Mukanya,N., Pukuta - Simbu,E., Mujula,Y., Nundu,S.S., Akil - Bandali,P., Kavunga,H., Lushima,R.S., Vercauteren,K., Sam-Agudu,N.A., Mills,E.J., Tshiani - Mbaya,O., Hoff,N., Rimoin,A.W., Hensley,L.E., Kundrachuk,J., Ayouba,A., Peeters,M., Delaporte,E., Nachega,J.B., Ahuka - Mundeke,S., Muyembe - Tamfum,J.-J., Rambaut,A., Liesenborghs,L. and Mbala - Kingebeni,P.
EPI_ISL_19108154, EPI_ISL_19108157, EPI_ISL_19108158, EPI_ISL_19108159, EPI_ISL_19108160, EPI_ISL_19108161, EPI_ISL_19108165, EPI_ISL_19108167, EPI_ISL_19108169, EPI_ISL_19108170	Oxford University Clinical Research Unt	Oxford University Clinical Research Unt	Huynh Thi Thuy Hoa, Nguyen Thanh Dung, Le Manh Hung, Nguyen Thi Thu Hong, Vo Truong Quy, Hoang Truong, Nguyen Trong Duy, Tran Minh Hoang, Nguyen Thi Thanh, Mai Hong Phuoc, Nguyen Nhut Thong, Nguyen Duc Huy, Vu Thi Kim Thoa, Nghiem My Ngoc, Vo Trong Vuong, Ngo Tan Tai, Huynh Kim Nhung, Dao Phuong Linh, Pham Thi Ngoc Thoa, Lam Minh Yen, Nguyen Thi Thao, Tran Ba Thien, Truong Hoang Chau Truc, Le Kim Thanh, Vo Tan Hoang, Nguyen Thanh Ngoc, Tran Tan Thanh, Louise Thwaites, Nguyen Van Vinh Chau, Guy Thwaites, Nguyen To Anh, Le Van Tan
EPI_ISL_19109197, EPI_ISL_19131278, EPI_ISL_19131279	Laboratory Medicine and Pathology, University of Washington	Laboratory Medicine and Pathology, University of Washington	Roychoudhury,P., Xie,X., Sereewit,J., Ellis,S. and Greninger,A.
EPI_ISL_19131334, EPI_ISL_19131335, EPI_ISL_19131336, EPI_ISL_19131337, EPI_ISL_19131338, EPI_ISL_19131339, EPI_ISL_19131340, EPI_ISL_19131341, EPI_ISL_19131342, EPI_ISL_19131343, EPI_ISL_19131344, EPI_ISL_19131345			
see above	Indian Council of Medical Research-National Institute of Virology, Microbial Containment Complex	Indian Council of Medical Research-National Institute of Virology, Microbial Containment Complex	Pragya D. Yadav
EPI_ISL_19139151	QUEST DIAGNOSTICS WEST HILLS	Los Angeles County Public Health Laboratories	N. Heibeck et al.
EPI_ISL_19143445, EPI_ISL_19143447, EPI_ISL_19143448, EPI_ISL_19143449, EPI_ISL_19143450, EPI_ISL_19143451, EPI_ISL_19143452, EPI_ISL_19143453, EPI_ISL_19143454, EPI_ISL_19143455, EPI_ISL_19143458, EPI_ISL_19143459, EPI_ISL_19143460, EPI_ISL_19143462, EPI_ISL_19143464, EPI_ISL_19143467, EPI_ISL_19143468, EPI_ISL_19143469, EPI_ISL_19143470, EPI_ISL_19143471, EPI_ISL_19143473, EPI_ISL_19143475, EPI_ISL_19143476			
see above	California Department of Public Health	California Department of Public Health	Kath, C., Haw, M., Espinosa, A., and Hacker, J.
EPI_ISL_19151800, EPI_ISL_19151801	Tokyo Metropolitan Institute of Public Health	Tokyo Metropolitan Institute of Public Health	Wakaba Okada, Ryota Kumagai, Sachiko Harada, Yu Yaoita, Arisa Amano, Kumiko Takahashi, Mami Nagashima, Kenji Sadamasu

EPI_ISL_19158945, EPI_ISL_19158946, EPI_ISL_19158948, EPI_ISL_19158949, EPI_ISL_19158950	Northwestern Medicine	RIPHL at Rush University Medical Center	Stefan Green, Kevin Kunstman, Hannah Barbian, Sofiya Bobrovska, Felix Araujo Perez, Erin Newcomer, Alyse Kitttner
EPI_ISL_19159108	RSPI Sulianti Saroso	Balai Besar Laboratorium Biologi Kesehatan	Hana Apsari Pawestri, Arie Ardiansyah Nugraha, Fajar Nur Sulistyahadi, Markus Evan Anggia, Subangkit
EPI_ISL_19159109	RSUP Dr Hasan Sadikin	Balai Besar Laboratorium Biologi Kesehatan	Hana Apsari Pawestri, Arie Ardiansyah Nugraha, Fajar Nur Sulistyahadi, Markus Evan Anggia, Subangkit
EPI_ISL_19159110	Puskesmas Cilodong	Balai Besar Laboratorium Biologi Kesehatan	Hana Apsari Pawestri, Arie Ardiansyah Nugraha, Fajar Nur Sulistyahadi, Markus Evan Anggia, Subangkit
EPI_ISL_19159111	RSUD Cengkareng	Balai Besar Laboratorium Biologi Kesehatan	Hana Apsari Pawestri, Arie Ardiansyah Nugraha, Fajar Nur Sulistyahadi, Markus Evan Anggia, Subangkit
EPI_ISL_19159112	Eka Hospital BSD	Balai Besar Laboratorium Biologi Kesehatan	Hana Apsari Pawestri, Arie Ardiansyah Nugraha, Fajar Nur Sulistyahadi, Markus Evan Anggia, Subangkit
EPI_ISL_19159113	PKC Tebet	Balai Besar Laboratorium Biologi Kesehatan	Hana Apsari Pawestri, Arie Ardiansyah Nugraha, Fajar Nur Sulistyahadi, Markus Evan Anggia, Subangkit
EPI_ISL_19159114	PKC Menteng	Balai Besar Laboratorium Biologi Kesehatan	Hana Apsari Pawestri, Arie Ardiansyah Nugraha, Fajar Nur Sulistyahadi, Markus Evan Anggia, Subangkit
EPI_ISL_19159115, EPI_ISL_19159116	PKC Tanjung Priok	Balai Besar Laboratorium Biologi Kesehatan	Hana Apsari Pawestri, Arie Ardiansyah Nugraha, Fajar Nur Sulistyahadi, Markus Evan Anggia, Subangkit
EPI_ISL_19159119	RSPI Sulianti Saroso	Balai Besar Laboratorium Biologi Kesehatan	Hana Apsari Pawestri, Arie Ardiansyah Nugraha, Fajar Nur Sulistyahadi, Markus Evan Anggia, Subangkit
EPI_ISL_19159120	RSUP Fatmawati	Balai Besar Laboratorium Biologi Kesehatan	Hana Apsari Pawestri, Arie Ardiansyah Nugraha, Fajar Nur Sulistyahadi, Markus Evan Anggia, Subangkit
EPI_ISL_19170459, EPI_ISL_19170460, EPI_ISL_19170462, EPI_ISL_19170465, EPI_ISL_19170468, EPI_ISL_19170469, EPI_ISL_19170470, EPI_ISL_19170471, EPI_ISL_19170472, EPI_ISL_19170473, EPI_ISL_19170475, EPI_ISL_19170476, EPI_ISL_19170478, EPI_ISL_19170479, EPI_ISL_19170480, EPI_ISL_19170481, EPI_ISL_19170483, EPI_ISL_19170486			
see above	California Department of Public Health	California Department of Public Health	Kath, C., Haw, M., Espinosa, A., and Hacker, J.
EPI_ISL_19193028	California Department of Public Health, Viral and Rickettsial Disease Laboratory	California Department of Public Health, Viral and Rickettsial Disease Laboratory	Kath,C., Haw,M., Espinosa,A. and Hacker,J.
EPI_ISL_19196357	Laboratory Corporation of America	Los Angeles County Public Health Laboratories	S. McCann et. al.
EPI_ISL_19196359	Quest Diagnostic Nichols Institute	Los Angeles County Public Health Laboratories	S. McCann et. al.
EPI_ISL_19204034	QUEST DIAGNOSTIC NICHOLS INSTITUTE	Los Angeles County Public Health Laboratories	S. McCann et. al.
EPI_ISL_19204035	QUEST DIAGNOSTICS WEST HILLS	Los Angeles County Public Health Laboratories	S. McCann et. al.
EPI_ISL_19205399, EPI_ISL_19205401, EPI_ISL_19205402, EPI_ISL_19205403, EPI_ISL_19205404, EPI_ISL_19205405, EPI_ISL_19205407	Laboratorio de Enterovirus, Instituto Oswaldo Cruz, Fiocruz	Instituto Oswaldo Cruz FIOCRUZ - Laboratory of Respiratory Viruses and Measles (LVRS)	Paola Resende, Elisa Cavalcante Pereira, Bruna Mendonça da Silva, Jéssica Graça Macedo de Carvalho, Larissa Macedo Pinto, Victor Guimaraes, Luciana Appolinario, Alice Sampaio, Marilda Siqueira, Renan da Silva Faustino, Marilia Santini, Edson Elias da Silva on behalf of the Fiocruz Genomic Surveillance Network
EPI_ISL_19230668	Virologia, INEI- ANLIS Dr. Carlos G. Malbran	Virologia, INEI- ANLIS Dr. Carlos G. Malbran	Lewis,A., Josiowicz,A., Poklepovich,T., Mallou,F., Cuba,F., Haim,M. and Cisterna,D.
EPI_ISL_19243972, EPI_ISL_19243973	Laboratory Medicine and Pathology, University of Washington	Laboratory Medicine and Pathology, University of Washington	Roychoudhury,P., Xie,X., Sereewit,J., Ellis,S. and Greninger,A.
EPI_ISL_19256183, EPI_ISL_19256184, EPI_ISL_19256185, EPI_ISL_19256186, EPI_ISL_19256187, EPI_ISL_19256188, EPI_ISL_19256189, EPI_ISL_19256190, EPI_ISL_19256191, EPI_ISL_19256192, EPI_ISL_19256193, EPI_ISL_19256194, EPI_ISL_19256195, EPI_ISL_19256196, EPI_ISL_19256197, EPI_ISL_19256198, EPI_ISL_19256199, EPI_ISL_19256200, EPI_ISL_19256201, EPI_ISL_19256203, EPI_ISL_19256205, EPI_ISL_19256206, EPI_ISL_19256207, EPI_ISL_19256208, EPI_ISL_19256209, EPI_ISL_19256210, EPI_ISL_19256211, EPI_ISL_19256212, EPI_ISL_19256213, EPI_ISL_19256214, EPI_ISL_19256215, EPI_ISL_19256216, EPI_ISL_19256217, EPI_ISL_19256218, EPI_ISL_19256219, EPI_ISL_19256220, EPI_ISL_19256221, EPI_ISL_19256222, EPI_ISL_19256223, EPI_ISL_19256224, EPI_ISL_19256225, EPI_ISL_19256226, EPI_ISL_19256227, EPI_ISL_19256228, EPI_ISL_19256229, EPI_ISL_19256230, EPI_ISL_19256232, EPI_ISL_19256233, EPI_ISL_19256234, EPI_ISL_19256235, EPI_ISL_19256236, EPI_ISL_19256237, EPI_ISL_19256240, EPI_ISL_19256242, EPI_ISL_19256243, EPI_ISL_19256247, EPI_ISL_19256248, EPI_ISL_19256249, EPI_ISL_19256250, EPI_ISL_19256251, EPI_ISL_19256252, EPI_ISL_19256253, EPI_ISL_19256254, EPI_ISL_19256255, EPI_ISL_19256256, EPI_ISL_19256257, EPI_ISL_19256258, EPI_ISL_19256259, EPI_ISL_19256260, EPI_ISL_19256261, EPI_ISL_19256262, EPI_ISL_19256263, EPI_ISL_19256264, EPI_ISL_19256265, EPI_ISL_19256266, EPI_ISL_19256267, EPI_ISL_19256268, EPI_ISL_19256269, EPI_ISL_19256270, EPI_ISL_19256271, EPI_ISL_19256272, EPI_ISL_19256273, EPI_ISL_19256274, EPI_ISL_19256275, EPI_ISL_19256276, EPI_ISL_19256277, EPI_ISL_19256278, EPI_ISL_19256280, EPI_ISL_19256281, EPI_ISL_19256282, EPI_ISL_19256283, EPI_ISL_19256284, EPI_ISL_19256285, EPI_ISL_19256286, EPI_ISL_19256287, EPI_ISL_19256288, EPI_ISL_19256289, EPI_ISL_19256290, EPI_ISL_19256291, EPI_ISL_19256292, EPI_ISL_19256294, EPI_ISL_19256295			
see above	Nigeria Centre for Disease Control and Prevention	Institute of Ecology and Evolution, University of Edinburgh	Parker,E., Omah,I.F., Varilly,P., Magee,A., Ayinla,A.O., Sijuwola,A.E., Ahmed,M.I., Ope-ewe,O.O., Ogunsanya,O.A., Olono,A., Eromon,P., Tomkins-Tinch,C.H., Otieno,J.R., Akanbi,O., Egwuenu,A., Ehiakhamen,O., Chukwu,C., Suleiman,K., Akinpelu,A., Ahmad,A., Imam,K.I., Ojedele,R., Oripenaye,V., Ikeata,K., Adelakun,S., Olajumoke,B., Djuicy,D.D., Messanga Essengue,L.L., Moumbeket Yifomnjou,M.H., Zeller,M., Gangavarapu,K., O'Toole,A., Park,D.J., Mboowa,G., Tessema,S.K., Tebeje,Y.K., Folarin,O., Happi,A., Lemey,P., Suchard,M.A., Andersen,K.G., Sabeti,P., Rambaut,A., Njoum,R., Ihekweazu,C., Jide,I., Adetifa,I. and Happi,C.T.
EPI_ISL_19256299, EPI_ISL_19256300, EPI_ISL_19256301, EPI_ISL_19256302, EPI_ISL_19256303, EPI_ISL_19256304	Centre Pasteur of Cameroon	Institute of Ecology and Evolution, University of Edinburgh	Djuicy,D.D., Omah,I.F., Parker,E., Tomkins-Tinch,C.H., Otieno,J.R., Yifomnjou,M.H.M., Essengue,L.L.M., Ayinla,A.O., Sijuwola,A.E., Ahmed,M.I., Ope-ewe,O.O., Ogunsanya,O.A., Olono,A., Eromon,P., Yonga,M.G.W., Essima,G.D., Touoyem,I.P., Mounchili,L.J.M., Eyangoh,S.I., Esso,L., Nguidjol,I.M.E., Metomb,S.F., Chebo,C., Agwe,S.M., Mossi,H.M., Bilounga,C.N., Etoundi,A.G.M., Akanbi,O., Egwuenu,A., Ehiakhamen,O., Chukwu,C., Suleiman,K., Akinpelu,A., Ahmad,A., Imam,K.I., Ojedele,R., Oripenaye,V., Ikeata,K., Adelakun,S., Olajumoke,B., O'Toole,A., Magee,A., Zeller,M., Gangavarapu,K., Varilly,P., Park,D.J., Mboowa,G., Kifle Tessema,S., Tebeje,Y.K., Folarin,O., Happi,A., Lemey,P., Suchard,M.A., Andersen,K.G., Sabeti,P., Rambaut,A., Ihekweazu,C., Jide,I., Adetifa,I., Njoum,R. and Happi,C.T.
EPI_ISL_19273374	Labcorp	Los Angeles County Public Health Laboratories	J. Garrigues et. al.
EPI_ISL_19273375, EPI_ISL_19273376	Quest Diagnostics Nichols Institute	Los Angeles County Public Health Laboratories	J. Garrigues et. al.
EPI_ISL_19287109	Pathogen Genomics Lab, National Institute for Biomedical Research (INRB)	Pathogen Genomics Lab, National Institute for Biomedical Research (INRB)	Placide Mbala-Kingebeni, Eddy Kinganda-Lusamaki, Adrienne Amuri-Aziza, Elisabeth Pukuta, Emmanuel Lokilo Lofiko, Gradi Luakanda Ndelemo, Francisca Muyembe Mawete, Jean Claude Makangara Cigolo, Elisabeth Muyamuna, Sydney Merritt, Raphaël Lumembe Numbi, Gabriel Kabamba Lungenyi, Prince Akil Bandali, Pauline Musuamba Kayembe, Rilia Ola Mpumbe, Princesse Paku Tshambu, Sifa Kavira Bpitani, Emile Malembi, Emmanuel Hasivirwe Vakaniaki, Francois Kasongo, Innocent Kanda, Emile Malembi, Lygie Lunyanga , Sylvie Linsuke, Yvon Anta, Pierrot Bolunza, Raoul Nganzobo, Sophie Gabia, Ahidjo Ayouba, Nicole A. Hoff, Lisa E. Hensley, Jason Kindrachuk, Anne W. Rimoin, Nicola Low, Steve Ahuka-Mundeke, Eric Delaporte, Martine Peeters, Jean-Jacques Muyembe Tamfum
EPI_ISL_19293742	National Center for Global Health and Medicine	National Center for Global Health and Medicine	Higashi-Kuwata,N., Okada,W., Takahashi,K., Nagashima,M., Morioka,S., Iwamoto,N., Sadamasu,K., Yoshimura,K., Ohmagari,N. and Mitsuya,H.
EPI_ISL_19295744, EPI_ISL_19295750, EPI_ISL_19295752, EPI_ISL_19295754, EPI_ISL_19295757, EPI_ISL_19295758, EPI_ISL_19295760, EPI_ISL_19295761, EPI_ISL_19295762, EPI_ISL_19295763, EPI_ISL_19295764, EPI_ISL_19295765, EPI_ISL_19295767, EPI_ISL_19295768, EPI_ISL_19295769, EPI_ISL_19295770, EPI_ISL_19295771, EPI_ISL_19295772, EPI_ISL_19295778, EPI_ISL_19295782			
see above	California Department of Public Health	California Department of Public Health	Kath, C., Haw, M., Espinosa, A., and Hacker, J.
EPI_ISL_19305614, EPI_ISL_19305615	MRC/UVRI & LSHTM Uganda Research Unit, Uganda Virus Research Institute	MRC/UVRI & LSHTM Uganda Research Unit, Uganda Virus Research Institute	Nicholas Bbosa, Stella E. Nabirye, Hamidah S. Namagembe, Ronald Kiiza, Alfred Ssekagiri, Mary Munyagwa, Arafat Bwambale, Stephen Bagonza, Henry Kyobe Bosa, Mary Rodgers, Francisco Averhoff, Michael Berg, Robert Downing, Gavin Cloherty, Julius Lutwama, Pontiano Kaleebu, Deogratius Ssemwanga
