# Supplementary material for: Update of the Genetic Variability of Monkeypox Virus Clade IIb Lineage B.1
Source: Microorganisms. 2024 Sep 11;12(9):1874. doi: 10.3390/microorganisms12091874 (PMC11434445; doi:10.3390/microorganisms12091874)
Supplement: Supplementary file 1 [file microorganisms-12-01874-s001.zip › Table_S2.pdf]

We gratefully acknowledge the following Authors from the Originating laboratories responsible for obtaining the specimens, as well as the Submitting laboratories where the genome data were generated and shared via GISAID, on which this research is based.

All Submitters of data may be contacted directly via www.gisaid.org

Authors are sorted alphabetically.

Accession ID	Originating Laboratory	Submitting Laboratory	Authors
EPI_ISL_13052263	Microbiol Genomics and Bioinformatics, Bundeswehr Institute of Microbiology	Microbiol Genomics and Bioinformatics, Bundeswehr Institute of Microbiology	Antwerpen,M.H., Lang,D., Zange,S., Walter,M.C. and Woelfel,R.
EPI_ISL_13052266, EPI_ISL_13052267, EPI_ISL_13052268, EPI_ISL_13052269, EPI_ISL_13052270, EPI_ISL_13052272, EPI_ISL_13052273	Instituto Nacional de Saude Doutor Ricardo Jorge (INSA)	Instituto Nacional de Saude Doutor Ricardo Jorge (INSA)	Joana Isidro, Vítor Borges, Miguel Pinto, Daniel Sobral, João Dourado Santos, Alexandra Nunes, Verónica Mixão, Rita Ferreira, Daniela Santos, Silvia Duarte, Luís Vieira, Maria José Borrego, Sofia Núncio, Isabel Lopes de Carvalho, Ana Pelerito, Rita Cordeiro, João Paulo Gomes
EPI_ISL_13052274	Laboratory of Virology, University Hospitals of Geneva	Laboratory of Virology, University Hospitals of Geneva	Laubscher,F., Chudzinski,V., Schibler,M., Kaiser,L. and Renzoni,A.
EPI_ISL_13052282	Microbiology, Immunology and Transplantation, KU Leuven, Rega Institute	Microbiology, Immunology and Transplantation, KU Leuven, Rega Institute	Vanmechelen,B., Wawina-Bokalanga,T., Logist,A.-S., Sinnesael,R., Ysebaert,L., Verlinden,J., Bloemen,M. and Maes,P.
EPI_ISL_13052283	Microbiology, Immunology and Transplantation, KU Leuven, Rega Institute	Microbiology, Immunology and Transplantation, KU Leuven, Rega Institute	Wawina-Bokalanga,T., Vanmechelen,B., Logist,A.-S., Sinnesael,R., Ysebaert,L., Verlinden,J., Bloemen,M. and Maes,P.
EPI_ISL_13052285	Laboratory of Virology, University Hospitals of Geneva	Laboratory of Virology, University Hospitals of Geneva	Laubscher,F., Schibler,M., Kaiser,L. and Renzoni,A.
EPI_ISL_13052288	Department of Health, Utah Public Health Laboratory	Department of Health, Utah Public Health Laboratory	Young,E.L., Hergert,J. and Oakeson,K.F.
EPI_ISL_13052290	Laboratory for Diagnostics of Zoonoses and WHO Centre, Institute of Microbiology and Immunology, Faculty of Medicine, University of Ljubljana	Laboratory for Diagnostics of Zoonoses and WHO Centre, Institute of Microbiology and Immunology, Faculty of Medicine, University of Ljubljana	Zakotnik,S., Vlaj,D., Suljic,A., Zorec,T.M., Korva,M., Poljak,M. and Avsic Zupanc,T.
EPI_ISL_13052295	SC (UCO) Igiene e Sanità Pubblica, ASUGI, Trieste	Genomics and Epigenomics, AREA Science Park	Licastro,D., DeGasperi,M., Negri,C., Piscianz,E., Koncan,R., Dal Monego,S., Segat,L. and D'Agaro,P.
EPI_ISL_13056892, EPI_ISL_13056893, EPI_ISL_13056896, EPI_ISL_13056901, EPI_ISL_13056902, EPI_ISL_13056903, EPI_ISL_13056904, EPI_ISL_13056905, EPI_ISL_13056906, EPI_ISL_13056907, EPI_ISL_13056908, EPI_ISL_13056909			
see above	Instituto Nacional de Saude Doutor Ricardo Jorge (INSA)	Instituto Nacional de Saude Doutor Ricardo Jorge (INSA)	Joana Isidro, Vítor Borges, Miguel Pinto, Daniel Sobral, João Dourado Santos, Alexandra Nunes, Verónica Mixão, Rita Ferreira, Daniela Santos, Silvia Duarte, Luís Vieira, Maria José Borrego, Sofia Núncio, Isabel Lopes de Carvalho, Ana Pelerito, Rita Cordeiro, João Paulo Gomes
EPI_ISL_13056910	Biochemistry and Molecular Genetics, Israel Institute for Biological Research	Biochemistry and Molecular Genetics, Israel Institute for Biological Research	Israeli,O., Guedj-Dana,Y., Lazar,S., Shifman,O., Erez,N., Weiss,S., Paran,N., Israely,T., Schuster,O., Zvi,A., Beth-Din,A. and Cohen Gihon,I.
EPI_ISL_13191438	Instituto de Infectologia Emilio Ribas	Instituto Adolfo Lutz Strategic Laboratory	Claudio Tavares Sacchi, Karoline Rodrigues Campos, Marlon Benedito Nascimento Santos, Alex Domingos Reis, Ariadne Ferreira Amarante, Adriano Abbud, Adriana Bugno, Walkiria Delnoro Almeida Prado, Regiane Cardoso de Paula
EPI_ISL_13194516	Alberta Precision Laboratories	Alberta Precision Laboratories	Matthew Croxen, Ashwin Deo, Paul Dieu, Xiaoli Dong, Kara Gill, David Granger, Christina Ferrato, Vanipriyadarsini Ikkurti, Jamil Kanji, Petya Koleva, Vincent Li, Colin Lloyd, Tarah Lynch, Raymond Ma, Kanti Pabbaraju, Silas Rotich, Hilary Sergeant, Steven Shideler, Todd Skitsko, Sandy Shokopoles, Graham Tipples, Johanna Thayer, Anita Wong
EPI_ISL_13234112	Laboratório Central de Saúde Pública do Estado do Rio Grande do Sul	Instituto Adolfo Lutz Strategic Laboratory	Claudio Tavares Sacchi, Karoline Rodrigues Campos, Adriano Abbud, Adriana Bugno
EPI_ISL_13244349	Erasmus Medical Center Department of Virology	Erasmus Medical Center Department of Virology	Bas Oude Munnink, Marjan Boter, Babette Weller, Richard Molenkamp, Janette Rahamat-Langendoen, Reina Sikkema, Marion Koopmans
EPI_ISL_13251120	Laboratory of Virology, INMI Lazzaro Spallanzani IRCCS	Laboratory of Virology, INMI Lazzaro Spallanzani IRCCS	Giombini,E., Gruber,C.E.M., Rueca,M., Gramigna,G., Vlta,S., Carletti,F., D'Abramo,A., Lapa,D., Puro,V., Fabeni,L., Butera,O., Colavita,F., Meschi,S., Matusali,G., Specchiarello,E., Vairo,F., Vaia,F., Garbuglia,A.R., Nicastri,E., Antinori,A., GIrardi,E. and Maggi,F.
EPI_ISL_13251723	checkin Zollhaus	Institute of Medical Virology, University of Zurich	Verena Kufner, Gabriela Ziltener, Maryam Zaheri, Stefan Schmutz, Annette Audigé, Odette Bernasconi, Kevin Steiner, Jon Huder, Cyril Shah, Riccarda Capaul, Guido Bloemberg, Jürg Böni, Michael Huber, Alexandra Trkola
EPI_ISL_13269478	Alberta Precision Laboratories	Alberta Precision Laboratories	Matthew Croxen, Ashwin Deo, Paul Dieu, Xiaoli Dong, Kara Gill, David Granger, Christina Ferrato, Vanipriyadarsini Ikkurti, Jamil Kanji, Petya Koleva, Vincent Li, Colin Lloyd, Tarah Lynch, Raymond Ma, Kanti Pabbaraju, Silas Rotich, Hilary Sergeant, Steven Shideler, Todd Skitsko, Sandy Shokopoles, Graham Tipples, Johanna Thayer, Anita Wong
EPI_ISL_13302316	Laboratory of Clinical Microbiology, Virology and Bioemergencies. ASST-Fatebenefratelli-Sacco, L.Sacco University Hospital	Army Medical and Veterinary Research Center	Silvia Fillo, Riccardo De Sanctis, Giovanni Faggioni, Andrea Ciammaruconi, Anna Anselmo, Vanessa Vera Fain, Simone Di Sabatino, Francesco Giordani, Antonella Fortunato, Rossella Brandi, Giulia Campoli, Marzia Cavalli, Anella Monte, Martina Lipari, Maria Di Spirito, Giorgia Grilli, Silvia Chimienti, Giandomenico Cerreto, Filippo Molinari, Giancarlo Petralito, Davide Mileto, Valeria Micheli, Maria Rita Gismondo, Florigio Lista
EPI_ISL_13308158, EPI_ISL_13308160	IRBA Research Institute Biomédicale Des Armées	IRBA Research Institute Biomédicale Des Armées	Jarjaval,F., Nolent,F., Criqui,A., Chapus,C., Lamer,O., Ferraris,O. and Gorge,O.
EPI_ISL_13314740	Laboratorio de Vigilancia em Saude de Vinhedo	Instituto Adolfo Lutz Strategic Laboratory	Claudio Tavares Sacchi, Karoline Rodrigues Campos, Adriano Abbud, Adriana Bugno
EPI_ISL_13331598	Department for Virology, Molecular Biology and Genome Research, R. G. Lugar Center for Public Health Research, National Center for Disease Control and Public Health (NCDC) of Georgia	Department for Virology, Molecular Biology and Genome Research, R. G. Lugar Center for Public Health Research, National Center for Disease Control and Public Health (NCDC) of Georgia	Giorgi Tomashvili, Salome Javashvili, Meri Pantsulaia, Gvantsa Brachveli, Ana Papkiauri, Giorgi Gogoladze, Gvantsa Chanturia, Adam Kotorashvili, Maia Alkhazashvili, Khatuna Zakhashvili, Paata Imnadze, Amiran Gamkrelidze.
EPI_ISL_13331713	Laboratory of Virology, INMI Lazzaro Spallanzani IRCCS	Laboratory of Virology, INMI Lazzaro Spallanzani IRCCS	Gramigna,G., Giombini,E., Gruber,C.E.M., Rueca,M., Carletti,F., Cicalini,S., Lapa,D., Puro,V., Marani,A., Fabeni,L., Butera,O., Colavita,F., Meschi,S., Matusali,G., Rivano Capparuccia,M., Specchiarello,E., Vairo,F., Vaia,F., Nicastri,E., Antinori,A., Girardi,E. and Maggi,F.
EPI_ISL_13339105	Microbiology Service, Hospital Universitario Clinico San Cecilio, Granada	Microbiology Service, Hospital Universitario Clinico San Cecilio, Granada	Chueca N, de Salazar A, Viñuela L, Fuentes A, Casimiro-Soriguer CS, Perez-Florido J, Dopazo J, Garcia F
EPI_ISL_13343634	Instituto de Infectologia Emilio Ribas	Instituto Adolfo Lutz Strategic Laboratory	Claudio Tavares Sacchi, Karoline Rodrigues Campos, Adriano Abbud, Adriana Bugno
EPI_ISL_13343697	Fleury Medicina Dignóstica	Instituto Adolfo Lutz Strategic Laboratory	Claudio Tavares Sacchi, Karoline Rodrigues Campos, Adriano Abbud, Adriana Bugno
EPI_ISL_13343718	Hospital Santa Ignes	Instituto Adolfo Lutz Strategic Laboratory	Claudio Tavares Sacchi, Karoline Rodrigues Campos, Adriano Abbud, Adriana Bugno
EPI_ISL_13362760	Laboratorio di Epidemiologia Molecolare e Sanità Pubblica-Policlinico Bari	Istituto Zooprofilattico Sperimentale della Puglia e della Basilicata	Parisi A, Simone D, Capozzi L, Del Sambro L, Bianco A, Chironna M, Loconsole D, Sallustio F, Galante D, Pace L, Manzulli V, Fasanella A.
EPI_ISL_13363142	Hospital Universitari Vall d'Hebron	Hospital Universitari Vall d'Hebron	Maria Piñana, Cristina Andrés, Alejandra González-Sánchez, Damir Garcia-Cehic, Ariadna Rando, Juliana Esperalba, Maria Gema Codina, Maria Carmen Martin, Carla Castillo, Karen García, Rodrigo Vásquez, Maria Piquer, Tomàs Pumarola, Josep Quer, Andrés Antón
EPI_ISL_13374487	National Public Health Center, National Biosafety Laboratory	National Public Health Center, National Biosafety Laboratory	Judit Henczkó, Dániel Déri, Fruzsina Petrovay, Lili Jármi, Bernadett Pályi, Eszter Balla, Zoltán Kis
EPI_ISL_13408799, EPI_ISL_13408801	Public Health Agency of Canada, National Microbiology Laboratory	Public Health Agency of Canada, National Microbiology Laboratory	Knox,N., Hole,D., Duggan,A., Yadav,C., Haidl,E., Chapel,M., Graham,M., Domselaar,G.V., Jolly,G., Audet,J., Fernando,L., Antonation,K., Hagan,M., Griffiths,E., Leung,A., Safronetz,D., Eshaghi,A., Gubbay,J.B., Hasso,M., Marchand-Austin,A., Olsha,R. and Patel,S.N.
EPI_ISL_13408807, EPI_ISL_13408809, EPI_ISL_13408811, EPI_ISL_13408813, EPI_ISL_13408815, EPI_ISL_13408817, EPI_ISL_13408819, EPI_ISL_13408821, EPI_ISL_13408823, EPI_ISL_13408827, EPI_ISL_13408831, EPI_ISL_13408833, EPI_ISL_13408835			
see above	Public Health Agency of Canada, National Microbiology Laboratory	Public Health Agency of Canada, National Microbiology Laboratory	ncknox
EPI_ISL_13408837, EPI_ISL_13408841, EPI_ISL_13408843, EPI_ISL_13408847, EPI_ISL_13408849, EPI_ISL_13408851, EPI_ISL_13408853, EPI_ISL_13408855, EPI_ISL_13408857, EPI_ISL_13408859, EPI_ISL_13408861			
see above	Public Health Agency of Canada, National Microbiology Laboratory	Public Health Agency of Canada, National Microbiology Laboratory	Knox,N., Duggan,A., Yadav,C., Hole,D., Haidl,E., Chapel,M., Jolly,G., Domselaar,G.V., Antonation,K., Leung,A., Fernando,L., Audet,J., Hagan,M., Graham,M., Griffiths,E., Safronetz,D., Charest,H., Levade,I. and Fafard,J.
EPI_ISL_13436658	Coordenadoria de Vigilancia em Saude - Sao Paulo	Instituto Adolfo Lutz Strategic Laboratory	Claudio Tavares Sacchi, Karoline Rodrigues Campos, Ariadne Fereira Amarante, Adriano Abbud, Adriana Bugno
EPI_ISL_13436792	Hospital Santa Ignes	Instituto Adolfo Lutz Strategic Laboratory	Claudio Tavares Sacchi, Karoline Rodrigues Campos, Adriano Abbud, Adriana Bugno
EPI_ISL_13437056	Hosp. Alemao Oswaldo Cruz	Instituto Adolfo Lutz Strategic Laboratory	Claudio Tavares Sacchi, Karoline Rodrigues Campos, Ariadne Ferreira Amarante, Adriano Abbud, Adriana Bugno
EPI_ISL_13449965, EPI_ISL_13449966	Hospital Universitario La Paz, Microbiology	Hospital Universitario La Paz, Microbiology	de la Hoz-Sanchez,B., Lopez-Ortiz,M., Gutierrez-Arroyo,A., Roces-Alvarez,P., Lazaro-Peona,F., Dahdouh,E., Bloise,I., Garcia-Rodriguez,J. and Mingorance,J.
EPI_ISL_13459346	CRT-DST-AIDS	Instituto Adolfo Lutz Strategic Laboratory	Claudio Tavares Sacchi, Karoline Rodrigues Campos, Ariadne Ferreira Amarante, Adriano Abbud, Adriana Bugno
EPI_ISL_13459347, EPI_ISL_13459482, EPI_ISL_13459483	Instituto de Infectologia Emilio Ribas	Instituto Adolfo Lutz Strategic Laboratory	Claudio Tavares Sacchi, Karoline Rodrigues Campos, Ariadne Ferreira Amarante, Adriano Abbud, Adriana Bugno
EPI_ISL_13466448, EPI_ISL_13466449, EPI_ISL_13466450, EPI_ISL_13466451, EPI_ISL_13466452, EPI_ISL_13466453, EPI_ISL_13466455, EPI_ISL_13466456, EPI_ISL_13466457, EPI_ISL_13466459, EPI_ISL_13466460, EPI_ISL_13466461, EPI_ISL_13466465			
see above	Department of Infectious Diseases, National Institute of Health Doutor Ricardo Jorge, Portugal (INSA)	Department of Infectious Diseases, National Institute of Health Doutor Ricardo Jorge, Portugal (INSA)	Isidro,J., Borges,V., Pinto,M., Sobral,D., Santos,J., Nunes,A., Mixao,V., Ferreira,R., Santos,D., Duarte,S., Vieira,L., Borrego,M.J., Nuncio,S., Lopes de Carvalho,I., Pelerito,A., Cordeiro,R., Gomes,J.P.
EPI_ISL_13484458	Laboratorio de Enterovirus, Instituto Oswaldo Cruz, Fiocruz	Instituto Oswaldo Cruz FIOCRUZ - Laboratory of Respiratory Viruses and Measles (LVRS)	Paola Resende, Elisa Cavalcante Pereira, Bruna Mendonça da Silva, Jéssica Graça Macedo de Carvalho, Larissa Macedo Pinto, Victor Guimaraes, Marilda Siqueira, Renan da Silva Faustino, Marilia Santini, Edson Elias da Silva on behalf of the Fiocruz Genomic Surveillance Network
EPI_ISL_13498265	National Institute for Communicable Diseases of the National Health Laboratory Service	National Institute for Communicable Diseases of the National Health Laboratory Service	Chan WY, Mtshali PS, Grobbelaar A, Moolla N, Mohale T, Du Plessis MG, Ismail A, Weyer J
EPI_ISL_13502582	Laboratory of Microbiology and Virology, Ospedale	Laboratory of Microbiology and Virology, Ospedale	Francesco Cerutti, Antonella Bottoni, Marisa Cazzadore, Tiziano Allice, Maria Grazia Milia, Gabriella Gregori, Elisa Burdino, Valeria Ghisetti

	Amedeo di Savoia, ASL "Città di Torino"	Amedeo di Savoia, ASL "Città di Torino"	
EPI_ISL_13508393	Hosp. Itacolomy Butanta	Instituto Adolfo Lutz Strategic Laboratory	Claudio Tavares Sacchi, Karoline Rodrigues Campos, Ariadne Ferreira Amarante, Adriano Abbud, Adriana Bugno
EPI_ISL_13508471	Instituto de Infectologia Emilio Ribas	Instituto Adolfo Lutz Strategic Laboratory	Claudio Tavares Sacchi, Karoline Rodrigues Campos, Ariadne Ferreira Amarante, Adriano Abbud, Adriana Bugno
EPI_ISL_13530881	Laboratorio de Referencia Nacional de Virus Respiratorios. Centro Nacional de Salud Publica. Instituto Nacional de Salud Peru.	Laboratorio de Referencia Nacional de Virus Respiratorios. Centro Nacional de Salud Publica. Instituto Nacional de Salud Peru.	Carlos Padilla Rojas, Veronica Hurtado Vela, Iris Silva Molina, Luren Sevilla Castañeda, Victor Jimenez Vasquez, Orson Mestanza Millones, Luis Barcena Flores, Wendy Lizarraga Olivares, Alicia Nuñez Llanos, Steve Acedo Lazo, Francisco Ascue Orosco, Kelly Izarra Rojas, Princesa Medrano Alhuay, Karla Vasquez Cajachahua, Estela Huaman Angeles, Jorge Giraldo Chavez, Lilian Huarca Balbin, Lisbet Roxana Inga Angulo, Maria Sandra Villar Saavedra, Henri Bailon Calderon, Lely Solari Zerpa, Gloria Arotinco Garayar. Equipo de vigilancia genomica del Instituto Nacional de Salud.
EPI_ISL_13537923	Microbiology, Immunology and Transplantation, KU Leuven, Rega Institute	Microbiology, Immunology and Transplantation, KU Leuven, Rega Institute	Wawina-Bokalanga,T., Vanmechelen,B., Logist,A.-S., Sinnesael,R., Ysebaert,L., Bloemen,M. and Maes,P.
EPI_ISL_13537924, EPI_ISL_13537925, EPI_ISL_13537926	Microbiology, Immunology and Transplantation, KU Leuven, Rega Institute	Microbiology, Immunology and Transplantation, KU Leuven, Rega Institute	Vanmechelen,B., Wawina-Bokalanga,T., Logist,A.-S., Sinnesael,R., Ysebaert,L., Verlinden,J., Van Holm,B., Bloemen,M. and Maes,P.
EPI_ISL_13544223, EPI_ISL_13544226, EPI_ISL_13544227, EPI_ISL_13544228, EPI_ISL_13544229, EPI_ISL_13544230, EPI_ISL_13544231, EPI_ISL_13544233, EPI_ISL_13544234, EPI_ISL_13544235	Public Health Agency of Canada, National Microbiology Laboratory	Public Health Agency of Canada, National Microbiology Laboratory	Duggan,A., Hole,D., Knox,N., Yadav,C., Haidl,E., Chapel,M., Domselaar,G.V., Jolly,G., Audet,J., Fernando,L., Antonation,K., Safronetz,D., Hagan,M., Griffiths,E., Leung,A., Graham,M., Peters,G., Go,A., Laminman,V., Kaplen,B., Eshaghi,A., Gubbay,J.B., Hasso,M., Marchand-Austin,A., Olsha,R. and Patel,S.N.
EPI_ISL_13544237, EPI_ISL_13544239, EPI_ISL_13544241, EPI_ISL_13544244, EPI_ISL_13544245, EPI_ISL_13544247, EPI_ISL_13544248, EPI_ISL_13544249, EPI_ISL_13544252, EPI_ISL_13544254, EPI_ISL_13544255, EPI_ISL_13544256, EPI_ISL_13544257, EPI_ISL_13544258, EPI_ISL_13544260, EPI_ISL_13544261, EPI_ISL_13544263, EPI_ISL_13544264, EPI_ISL_13544265, EPI_ISL_13544266, EPI_ISL_13544267			
see above	Public Health Agency of Canada, National Microbiology Laboratory	Public Health Agency of Canada, National Microbiology Laboratory	Duggan,A., Hole,D., Knox,N., Yadav,C., Haidl,E., Chapel,M., Domselaar,G.V., Fernando,L., Graham,M., Antonation,K., Audet,J., Hagan,M., Safronetz,D., Leung,A., Peters,G., Go,A., Laminman,V., Kaplen,B., Jolly,G., Charest,H., Levade,I. and Fafard,J.
EPI_ISL_13573943	Center for Virology, Medical University of Vienna	Medical University of Vienna Center for Virology	Jeremy V. Camp, Monika Redlberger-Fritz, Stephan W. Aberle
EPI_ISL_13584854, EPI_ISL_13586184	Institute for Virology, Philipps-University Marburg	Institute for Virology, Philipps-University Marburg	Eickmann, M., Lier, C., Kowalski, K., Kraft, F., Becker, S.
EPI_ISL_13607904	Servicio de Infectologia, Hospital Universitario Dr. José Eleuterio Gonzalez, Universidad Autonoma de Nuevo Leon	Centro de Investigacion e Innovacion en Virologia Medica, Departamento de Bioquimica y Medicina Molecular, Facultad de Medicina, Universidad Autonoma de Nuevo Leon	Kame A. Galan-Huerta, Manuel Paz Infanzon, Ali F. Ruiz Higareda, Laura Nuzzolo-Shihadeh, Adrian Camacho-Ortiz, Paola Bocanegra-Ibarias, Ana M. Rivas-Estilla, Daniel Zacarias-Villarreal, Luis A. Yamallel-Ortega, Maria D. Guerrero-Putz, Jorge Ocampo-Candiani
EPI_ISL_13624509	Instituto de Diagnóstico y Referencia Epidemiológicos/Jurisdicción Sanitaria Cuauhtémoc/Hospital Ángeles Roma	Instituto de Diagnóstico y Referencia Epidemiológicos/Instituto de Biotecnología UNAM	Adnan Araiza-Rodríguez, Adriana Salvador-Patiño, Alejandro Sánchez-Flores, América del Pilar Mandujano-Martínez, Blanca Taboada, Carlos Eduardo Hernández-Sánchez, Carlos F. Arias, Claudia Elena Wong-Arámbula, Daniel José Regalado-Santiago, David Esaú Fragoso-Fonseca, Elizabeth Andrade-Montiel, Fabiola Garcés-Ayala, Fernando González-Dominguez, Gabriel García-Rodríguez, Gloria Vázquez-Castro, Hugo López Gatell Ramírez, Irma López-Martínez, Jerome Verleyen, Jesús Trujillo, Jorge Ochoa, José Ernesto Ramírez-González, Karel Estrada-Guerra, Lucía Hernández-Rivas, Magaly Guadalupe Landa-Flores, Maribel González-Villa, Mireya Mederos-Michel, Nancy Martínez-Velázquez, Noé Escobar-Escamilla, Oliva López, Ricardo Cortés-Alcalá, Ricardo Grande, Verónica Jiménez-Jacinto
EPI_ISL_13632071	Center of Diagnostics and Vaccine Development, Centers for Disease Control, Taiwan	Center of Diagnostics and Vaccine Development, Centers for Disease Control, Taiwan	Jih-Hui Lin, Shu-Chun Chiu, Hsin-I, Huang, Wei-Lun Huang, Wen-Bin, Fann, Pei-Yu, Hsieh, Jyh-Yuan Yang
EPI_ISL_13632288	National Institute for Communicable Diseases of the National Health Laboratory Service	National Institute for Communicable Diseases of the National Health Laboratory Service	Chan WY, Mtshali PS, Grobbelaar A, Moolla N, Mohale T, Lowe M, Du Plessis MG, Ismail A, Weyer J
EPI_ISL_13651348, EPI_ISL_13651349, EPI_ISL_13651350	Laboratorio de Referencia Nacional de Virus Respiratorio. Centro Nacional de Salud Publica. Instituto Nacional de Salud.	Laboratorio de Referencia Nacional de Virus Respiratorio. Centro Nacional de Salud Publica. Instituto Nacional de Salud.	Carlos Padilla Rojas, Veronica Hurtado Vela, Iris Silva Molina, Luren Sevilla Castañeda, Victor Jimenez Vasquez, Orson Mestanza Millones, Luis Barcena Flores, Wendy Lizarraga Olivares, Alicia Nuñez Llanos, Steve Acedo Lazo, Francisco Ascue Orosco, Kelly Izarra Rojas, Princesa Medrano Alhuay, Karla Vasquez Cajachahua, Estela Huaman Angeles, Jorge Giraldo Chavez, Lilian Huarca Balbin, Lisbet Roxana Inga Angulo, Maria Sandra Villar Saavedra, Henri Bailon Calderon, Lely Solari Zerpa, Gloria Arotinco Garayar. Equipo de vigilancia genomica del Instituto Nacional de Salud.
EPI_ISL_13658019, EPI_ISL_13658021	Erasmus Medical Center Department of Virology	Erasmus Medical Center Department of Virology	Bas Oude Munnink, Marjan Boter, Babette Weller, Richard Molenkamp, Janette Rahamat-Langendoen, Reina Sikkema, Marion Koopmans
EPI_ISL_13705358	Hosp. Alemao Oswaldo Cruz	Instituto Adolfo Lutz Strategic Laboratory	Claudio Tavares Sacchi, Karoline Rodrigues Campos, Ariadne Ferreira Amarante, Marlon Benedito Nascimento Santos, Alex Domingos Reis, Adriano Abbud, Adriana Bugno
EPI_ISL_13705407	Hosp. Sirio-Libanes	Instituto Adolfo Lutz Strategic Laboratory	Claudio Tavares Sacchi, Karoline Rodrigues Campos, Ariadne Ferreira Amarante, Marlon Benedito Nascimento Santos, Alex Domingos Reis, Adriano Abbud, Adriana Bugno
EPI_ISL_13728303	Department of Medical Microbiology & Infection prevention, Amsterdam University Medical Centers location AMC	Department of Medical Microbiology & Infection prevention, Amsterdam University Medical Centers location AMC	Matthijs Welkers, Jelle Koopsen, Robin van Houdt, Marcel Jonges, Sebastian Matamoros, Sjoerd Rebers, Fokla Zorgdrager, Sylvia Bruisten, Judith den Uil, Akke Cornelissen, Janke Schinkel, Menno de Jong, Gini van Rijckevorsel and Mariken van der Lubben on behalf of the Amsterdam Regional Genomic epidemiology and Outbreak Surveillance (ARGOS) consortium
EPI_ISL_13732932	Hosp. Sao Joaquim - Beneficiencia Portuguesa	Instituto Adolfo Lutz Strategic Laboratory	Claudio Tavares Sacchi, Karoline Rodrigues Campos, Ariadne Ferreira Amarante, Marlon Benedito Nascimento Santos, Alex Domingos Reis, Adriano Abbud, Adriana Bugno
EPI_ISL_13744902	Department of Virology, Faculty of Medicine, University of Helsinki	Department of Virology, Faculty of Medicine, University of Helsinki	Kant,R., Smura,T., Vauhkonen,H. and Vapalahti,O.
EPI_ISL_13822667, EPI_ISL_13822668, EPI_ISL_13822669, EPI_ISL_13822718	Erasmus Medical Center Department of Virology	Erasmus Medical Center Department of Virology	Bas Oude Munnink, Marjan Boter, Babette Weller, Richard Molenkamp, Janette Rahamat-Langendoen, Reina Sikkema, Marion Koopmans
EPI_ISL_13827274, EPI_ISL_13827275, EPI_ISL_13827277, EPI_ISL_13827278, EPI_ISL_13827279, EPI_ISL_13827280, EPI_ISL_13827282	Public Health Agency of Canada, National Microbiology Laboratory	Public Health Agency of Canada, National Microbiology Laboratory	Duggan,A., Hole,D., Yadav,C., Knox,N., Haidl,E., Chapel,M., Domselaar,G.V., Fernando,L., Graham,M., Antonation,K., Audet,J., Hagan,M., Safronetz,D., Leung,A., Peters,G., Go,A., Laminman,V., Kaplen,B., Jolly,G., Marchand-Austin,A., Eshaghi,A., Patel,S.N., Hasso,M., Gubbay,J.B. and Olsha,R.
EPI_ISL_13833194, EPI_ISL_13833195, EPI_ISL_13833196, EPI_ISL_13833197	Laboratorio de Referencia Nacional de Virus Respiratorio. Centro Nacional de Salud Publica. Instituto Nacional de Salud.	Laboratorio de Referencia Nacional de Virus Respiratorio. Centro Nacional de Salud Publica. Instituto Nacional de Salud.	Carlos Padilla Rojas, Veronica Hurtado Vela, Iris Silva Molina, Luren Sevilla Castañeda, Victor Jimenez Vasquez, Orson Mestanza Millones, Luis Barcena Flores, Wendy Lizarraga Olivares, Alicia Nuñez Llanos, Steve Acedo Lazo, Francisco Ascue Orosco, Kelly Izarra Rojas, Princesa Medrano Alhuay, Karla Vasquez Cajachahua, Estela Huaman Angeles, Jorge Giraldo Chavez, Lilian Huarca Balbin, Lisbet Roxana Inga Angulo, Maria Sandra Villar Saavedra, Henri Bailon Calderon, Lely Solari Zerpa, Gloria Arotinco Garayar. Equipo de vigilancia genomica del Instituto Nacional de Salud.
EPI_ISL_13842269, EPI_ISL_13842548	Center for Virology, Medical University of Vienna	Medical University of Vienna Center for Virology	Jeremy V. Camp, Monika Redlberger-Fritz, Stephan W. Aberle
EPI_ISL_13889435, EPI_ISL_13889436, EPI_ISL_13889438, EPI_ISL_13889439, EPI_ISL_13889440, EPI_ISL_13889441, EPI_ISL_13889442, EPI_ISL_13889443, EPI_ISL_13889444, EPI_ISL_13889445, EPI_ISL_13889446, EPI_ISL_13889447, EPI_ISL_13889448, EPI_ISL_13889449, EPI_ISL_13889450, EPI_ISL_13889515, EPI_ISL_13889590, EPI_ISL_13889660, EPI_ISL_13889729, EPI_ISL_13889796, EPI_ISL_13889908, EPI_ISL_13889977, EPI_ISL_13890048, EPI_ISL_13890135, EPI_ISL_13890204, EPI_ISL_13890273, EPI_ISL_13890464, EPI_ISL_13890465, EPI_ISL_13890466, EPI_ISL_13890467, EPI_ISL_13890468, EPI_ISL_13890469, EPI_ISL_13890471, EPI_ISL_13890472, EPI_ISL_13890473, EPI_ISL_13890474, EPI_ISL_13890475, EPI_ISL_13890478, EPI_ISL_13890479, EPI_ISL_13890481			
see above	Charité Universitätsmedizin Berlin, Institut für Virologie/Labor Berlin	Charité Universitätsmedizin Berlin, Institut für Virologie	Terry C. Jones, Julia Schneider, Barbara Mühlemann, Talitha Veith, Jörn Beheim-Schwarzbach, Julia Tesch, Marie Luisa Schmidt, Felix Walper, Tobias Bleicker, Caroline Isner, Frieder Pfäfflin, Ricardo Niklas Werner, Victor M. Corman, Christian Drosten
EPI_ISL_13908332, EPI_ISL_13908333, EPI_ISL_13908334, EPI_ISL_13908335, EPI_ISL_13908336, EPI_ISL_13908337, EPI_ISL_13908338, EPI_ISL_13908339, EPI_ISL_13908340, EPI_ISL_13908341, EPI_ISL_13908342, EPI_ISL_13908343			
see above	Public Health Agency of Canada, National Microbiology Laboratory	Public Health Agency of Canada, National Microbiology Laboratory	Duggan,A., Hole,D., Yadav,C., Knox,N., Chapel,M., Tyler,A., Haidl,E., Domselaar,G.V., Antonation,K., Audet,J., Fernando,L., Hagan,M., Safronetz,D., Graham,M., Peters,G., Go,A., Laminman,V., Kaplen,B., Leung,A., Jolly,G., Fafard,J., Charest,H. and Levade,I.
EPI_ISL_13958697	Research and Evaluation, UKHSA	Research and Evaluation, UKHSA	Groves,N., Osman,K.L., Lewandowski,K.S., Carter,D.P., Pullan,S.T., Myers,R., Vipond,R. and Chand,M.
EPI_ISL_13983356	INSPI-Centro de Referencia Nacional de Virus Exantemáticos, Gastroentéricos y Transmitido por Vectores.	INSPI-Dirección Técnica de Investigación, Desarrollo e Innovación INSPI-Centro de Referencia Nacional de Genómica, Secuenciación y Bioinformática	Andrés Carrazco-Montalvo, Diana Gutiérrez, Naomi Mora, Silvia Salgado-Cisneros, Johana Parrales-Valdiviezo, Martha Sánchez-Domenech, Diego Morales, Gulnara Borja-Cabrera, Leandro Patiño*.
EPI_ISL_13993735, EPI_ISL_13993737, EPI_ISL_13993738, EPI_ISL_13993739	California Department of Public Health	California Department of Public Health	Viral and Rickettsial Disease Laboratory
EPI_ISL_14021725	Hosp. Municipal Enf. Antonio Policarpo de Oliveira	Instituto Adolfo Lutz Strategic Laboratory	Claudio Tavares Sacchi, Karoline Rodrigues Campos, Ariadne Ferreira Amarante, Marlon Benedito Nascimento Santos, Alex Domingos Reis, Adriano Abbud, Adriana Bugno
EPI_ISL_14050451, EPI_ISL_14050453, EPI_ISL_14050454, EPI_ISL_14050458	Public Health Agency of Canada, National Microbiology Laboratory	Public Health Agency of Canada, National Microbiology Laboratory	Duggan,A., Hole,D., Yadav,C., Knox,N., Tyler,A., Haidl,E., Chapel,M., Domselaar,G.V., Graham,M., Audet,J., Fernando,L., Hagan,M., Safronetz,D., Leung,A., Peters,G., Go,A., Laminman,V., Kaplen,B., Antonation,K., Jolly,G., Griffiths,E., Charest,H., Levade,I. and Fafard,J.
EPI_ISL_14070493, EPI_ISL_14070852, EPI_ISL_14070855	Instituto de Infectologia Emilio Ribas	Instituto Adolfo Lutz Strategic Laboratory	Claudio Tavares Sacchi, Karoline Rodrigues Campos, Ariadne Ferreira Amarante, Marlon Benedito Nascimento Santos, Alex Domingos Reis, Adriano Abbud, Adriana Bugno
EPI_ISL_14153982	Vajira Hospital	National Institute of Health, Department of Medical Sciences, Ministry of Public Health, Thailand	Pilailuk Okada; Siripaporn Phuygun; Nuttida Thongpramul; Thanutsapa Thanadachakul; Kazuhisa Okada; Archawin Rojanawiwat; Chakkarat Pitayawonganon; Supakit Sirilak
EPI_ISL_14166709	Medical University of Vienna Center for Virology	Medical University of Vienna Center for Virology	Jeremy V Camp, Monika Redlberger-Fritz, Stephan W. Aberle
EPI_ISL_14167248, EPI_ISL_14167573, EPI_ISL_14167574, EPI_ISL_14167575	Medical University of Vienna Center for Virology	Medical University of Vienna Center for Virology	Jeremy V. Camp, Monika Redlberger-Fritz, Stephan W. Aberle
EPI_ISL_14207724, EPI_ISL_14207725, EPI_ISL_14207726, EPI_ISL_14207730, EPI_ISL_14207731, EPI_ISL_14207733, EPI_ISL_14207734, EPI_ISL_14207735, EPI_ISL_14207738, EPI_ISL_14207739, EPI_ISL_14207740, EPI_ISL_14207741			
see above	Laboratorio de Referencia Nacional de Virus Respiratorio. Centro Nacional de Salud Publica. Instituto Nacional de Salud.	Laboratorio de Referencia Nacional de Virus Respiratorio. Centro Nacional de Salud Publica. Instituto Nacional de Salud.	Carlos Padilla Rojas, Veronica Hurtado Vela, Iris Silva Molina, Luren Sevilla Castañeda, Victor Jimenez Vasquez, Orson Mestanza Millones, Luis Barcena Flores, Wendy Lizarraga Olivares, Alicia Nuñez Llanos, Steve Acedo Lazo, Francisco Ascue Orosco, Kelly Izarra Rojas, Princesa Medrano Alhuay, Karla Vasquez Cajachahua, Estela Huaman Angeles, Jorge Giraldo Chavez, Lilian Huarca Balbin, Lisbet Roxana Inga Angulo, Maria Sandra Villar Saavedra, Henri Bailon Calderon, Lely Solari Zerpa, Gloria Arotinco Garayar. Equipo de vigilancia genomica del Instituto Nacional de Salud.
EPI_ISL_14211644, EPI_ISL_14211645	Public Health Authority of the Slovak Republic	Laboratory of Genomics and Bioinformatics, Comenius University Science Park	Tomáš Szemes, Edita Staroňová, Elena Tichá, Lucia Ševčíková, Terézia Vrabľová, Tatiana Sedláčková, Miroslav Böhmer, Jaroslav Budiš, Pavol Mišenko
EPI_ISL_14224334	Genetica Molecular and Subdepartamento de Virologia ISP Chile	Instituto de Salud Publica de Chile	Paulo C. Covarrubias, Andrés E. Castillo, Constanza Campano, Mariela Guajardo, Bárbara Parra, Rodrigo Fasce Pineda, Jorge Fernández
EPI_ISL_14254435, EPI_ISL_14254436, EPI_ISL_14254437, EPI_ISL_14254438	Erasmus Medical Center Department of Virology	Erasmus Medical Center Department of Virology	Bas Oude Munnink, Marjan Boter, Babette Weller, Babs Verstrepen, Richard Molenkamp, Janette Rahamat-Langendoen, Reina Sikkema, Marion Koopmans
EPI_ISL_14326638, EPI_ISL_14326639, EPI_ISL_14326640, EPI_ISL_14326641,	Environmental, Agricultural, and Occupational Health, University of Nebraska Medical Center, 984388	Environmental, Agricultural, and Occupational Health, University of Nebraska Medical Center, 984388	Tegomoh,B., Cross,S.T., Chapman,R.C., Bernhard,K., McCutchen,E.L., Fauver,J.R., Pratt,C.B., Warden,D.E., Iwen,P.C., Donahue,M. and Wiley,M.R.

EPI_ISL_14326642, EPI_ISL_14326643	Nebraska Medical Center	Nebraska Medical Center	
EPI_ISL_14326644	Environmental, Agricultural, and Occupational Health, University of Nebraska Medical Center, 984388 Nebraska Medical Center	Environmental, Agricultural, and Occupational Health, University of Nebraska Medical Center, 984388 Nebraska Medical Center	Tegomoh,B., Cross,S.T., Chapman,R.C., Bernhard,K., McCutchen,E.L., Fauver,J.R., Pratt,C.B., Warden,D.E., Iwen,P.C., Donahue,M. and Wiley,M.R
EPI_ISL_14414948	UMS Parque Industrial Curitiba	Instituto Adolfo Lutz Strategic Laboratory	Claudio Tavares Sacchi, Karoline Rodrigues Campos, Ariadne Ferreira Amarante, Marlon Benedito Nascimento Santos, Alex Domingos Reis, Adriano Abbud, Adriana Bugno
EPI_ISL_14415810	CTA Sao Miguel	Instituto Adolfo Lutz Strategic Laboratory	Claudio Tavares Sacchi, Karoline Rodrigues Campos, Ariadne Ferreira Amarante, Marlon Benedito Nascimento Santos, Alex Domingos Reis, Adriano Abbud, Adriana Bugno
EPI_ISL_14439713, EPI_ISL_14439714, EPI_ISL_14439715, EPI_ISL_14439716, EPI_ISL_14439717, EPI_ISL_14439718, EPI_ISL_14439719, EPI_ISL_14439720, EPI_ISL_14439721, EPI_ISL_14439722, EPI_ISL_14439723, EPI_ISL_14439724, EPI_ISL_14439725, EPI_ISL_14439726, EPI_ISL_14439728, EPI_ISL_14439729, EPI_ISL_14439730, EPI_ISL_14439731, EPI_ISL_14439732, EPI_ISL_14439733, EPI_ISL_14439734, EPI_ISL_14439735, EPI_ISL_14439736, EPI_ISL_14439737, EPI_ISL_14439738, EPI_ISL_14439739, EPI_ISL_14439740, EPI_ISL_14439741, EPI_ISL_14439742, EPI_ISL_14439743, EPI_ISL_14439745, EPI_ISL_14439746, EPI_ISL_14439747, EPI_ISL_14439748, EPI_ISL_14439749, EPI_ISL_14439750, EPI_ISL_14439751, EPI_ISL_14439752, EPI_ISL_14439753, EPI_ISL_14439754, EPI_ISL_14439755, EPI_ISL_14439756, EPI_ISL_14439757, EPI_ISL_14439758, EPI_ISL_14439759, EPI_ISL_14439760, EPI_ISL_14439761, EPI_ISL_14439762, EPI_ISL_14439763, EPI_ISL_14439764, EPI_ISL_14439765, EPI_ISL_14439766, EPI_ISL_14439767, EPI_ISL_14439768, EPI_ISL_14439769, EPI_ISL_14439770, EPI_ISL_14439771, EPI_ISL_14439772, EPI_ISL_14439773, EPI_ISL_14439774, EPI_ISL_14439775, EPI_ISL_14439776, EPI_ISL_14439777, EPI_ISL_14439779, EPI_ISL_14439780, EPI_ISL_14439781, EPI_ISL_14439782, EPI_ISL_14439784, EPI_ISL_14439785			
see above	Research and Evaluation, UKHSA	Research and Evaluation, UKHSA	Groves,N., Osman,K.L., Lewandowski,K.S., Carter,D.P., Pullan,S.T., Myers,R., Vipond,R. and Chand,M.
EPI_ISL_14445098, EPI_ISL_14445101, EPI_ISL_14445102, EPI_ISL_14445103, EPI_ISL_14445109, EPI_ISL_14445116, EPI_ISL_14445118, EPI_ISL_14445119, EPI_ISL_14445120, EPI_ISL_14445121, EPI_ISL_14445122, EPI_ISL_14445123, EPI_ISL_14445124, EPI_ISL_14445125, EPI_ISL_14445126, EPI_ISL_14445127, EPI_ISL_14445128, EPI_ISL_14445129, EPI_ISL_14445130, EPI_ISL_14445131, EPI_ISL_14445132, EPI_ISL_14445133, EPI_ISL_14445134, EPI_ISL_14445135, EPI_ISL_14445136, EPI_ISL_14445137, EPI_ISL_14445138, EPI_ISL_14445139, EPI_ISL_14445140, EPI_ISL_14445141, EPI_ISL_14445146, EPI_ISL_14445147, EPI_ISL_14445150, EPI_ISL_14445152, EPI_ISL_14445153			
see above	Laboratorio de Referencia Nacional de Virus Respiratorio. Centro Nacional de Salud Publica. Instituto Nacional de Salud.	Laboratorio de Referencia Nacional de Virus Respiratorio. Centro Nacional de Salud Publica. Instituto Nacional de Salud.	Carlos Padilla Rojas, Veronica Hurtado Vela, Iris Silva Molina, Luren Sevilla Castañeda, Victor Jimenez Vasquez, Orson Mestanza Millones, Luis Barcena Flores, Wendy Lizarraga Olivares, Alicia Nuñez Llanos, Steve Acedo Lazo, Francisco Ascue Orosco, Kelly Izarra Rojas, Princesa Medrano Alhuay, Karla Vasquez Cajachahua, Estela Huaman Angeles, Jorge Giraldo Chavez, Lilian Huarca Balbin, Lisbet Roxana Inga Angulo, Maria Sandra Villar Saavedra, Henri Bailon Calderon, Lely Solari Zerpa, Gloria Arotinco Garayar. Equipo de vigilancia genomica del Instituto Nacional de Salud.
EPI_ISL_14465517	Centro de Desenvolvimento Científico e Tecnológico (CDCT), Centro Estadual de Vigilância em Saúde (CEVS) da Secretaria Estadual da Saúde (SES-RS)	Centro de Desenvolvimento Científico e Tecnológico (CDCT), Centro Estadual de Vigilância em Saúde (CEVS) da Secretaria Estadual da Saúde (SES-RS)	Richard Steiner Salvato, Regina Bones Barcellos, Fernanda Marques Godinho
EPI_ISL_14467428, EPI_ISL_14467429	Laboratório Central de Saúde Pública do Amazonas - LACEN-AM	Laboratório de Ecologia de Doenças Transmissíveis na Amazônia, Instituto Leônidas e Maria Deane - Fiocruz Amazônia	Victor Souza, Fernanda Nascimento, Matilde Mejía, Dejanane Silva, Luciana Gonçalves, Tatyana Costa Amorim Ramos, Ana Ruth Lima Arcanjo, Valdinete Nascimento, Felipe Naveca on behalf of the Fiocruz COVID-19 Genomic Surveillance Network
EPI_ISL_14515201	Department of Infectious Diseases, National Institute of Health Doutor Ricardo Jorge, Portugal (INSA)	Department of Infectious Diseases, National Institute of Health Doutor Ricardo Jorge, Portugal (INSA)	Isidro,J., Borges,V., Pinto,M., Sobral,D., Santos,J., Nunes,A., Mixao,V., Ferreira,R., Santos,D., Duarte,S., Vieira,L., Borrego,M.J., Nuncio,S., Lopes de Carvalho,I., Pelerito,A., Cordeiro,R. and Gomes,J.P.
EPI_ISL_14526939, EPI_ISL_14526942, EPI_ISL_14526944, EPI_ISL_14526945, EPI_ISL_14526948, EPI_ISL_14526949, EPI_ISL_14526950, EPI_ISL_14526952, EPI_ISL_14526955, EPI_ISL_14526956	Connecticut Department of Public Health	Grubaugh Lab - Yale School of Public Health	Nicholas F. G. Chen, Chrispin Chaguza, Kien Pham, Nathan D. Grubaugh, Christina Nishimura, Claire Pearson, Kutluhan Incekara, Jian Ping Huang, Emily Gagnon, Ethan Reever, Jafar Razeq, Anthony Muyombwe, Chantal B. F. Vogels
EPI_ISL_14541645, EPI_ISL_14541654	Public Health Authority of the Slovak Republic	Laboratory of Genomics and Bioinformatics, Comenius University Science Park	Tomáš Szemes, Edita Staroňová, Elena Tichá, Lucia Ševčíková, Terézia Vrabľová, Tatiana Sedláčková, Miroslav Böhmer, Jaroslav Budiš, Pavol Mišenko
EPI_ISL_14571429	Hosp. Municipal Dr. Jose de Carvalho Florence	Instituto Adolfo Lutz Strategic Laboratory	Claudio Tavares Sacchi, Karoline Rodrigues Campos, Ariadne Ferreira Amarante, Marlon Benedito Nascimento Santos, Alex Domingos Reis, Adriano Abbud, Adriana Bugno
EPI_ISL_14571433	Casa de Saude Stella Maris	Instituto Adolfo Lutz Strategic Laboratory	Claudio Tavares Sacchi, Karoline Rodrigues Campos, Ariadne Ferreira Amarante, Marlon Benedito Nascimento Santos, Alex Domingos Reis, Adriano Abbud, Adriana Bugno
EPI_ISL_14571435	Secrtetaria Municipal de Saude de Sertaozinho	Instituto Adolfo Lutz Strategic Laboratory	Claudio Tavares Sacchi, Karoline Rodrigues Campos, Ariadne Ferreira Amarante, Marlon Benedito Nascimento Santos, Alex Domingos Reis, Adriano Abbud, Adriana Bugno
EPI_ISL_14571439	Secrtetaria Municipal de Saude de Sata Barbara D Oeste	Instituto Adolfo Lutz Strategic Laboratory	Claudio Tavares Sacchi, Karoline Rodrigues Campos, Ariadne Ferreira Amarante, Marlon Benedito Nascimento Santos, Alex Domingos Reis, Adriano Abbud, Adriana Bugno
EPI_ISL_14571441	Hosp. Municipal Dr. Waldemar Tebaldi	Instituto Adolfo Lutz Strategic Laboratory	Claudio Tavares Sacchi, Karoline Rodrigues Campos, Ariadne Ferreira Amarante, Marlon Benedito Nascimento Santos, Alex Domingos Reis, Adriano Abbud, Adriana Bugno
EPI_ISL_14571442	Instituto de Infectologia Emilio Ribas II Baixada Santista	Instituto Adolfo Lutz Strategic Laboratory	Claudio Tavares Sacchi, Karoline Rodrigues Campos, Ariadne Ferreira Amarante, Marlon Benedito Nascimento Santos, Alex Domingos Reis, Adriano Abbud, Adriana Bugno
EPI_ISL_14571444	UBDS DR. Italo Baruffi Castelo Branco	Instituto Adolfo Lutz Strategic Laboratory	Claudio Tavares Sacchi, Karoline Rodrigues Campos, Ariadne Ferreira Amarante, Marlon Benedito Nascimento Santos, Alex Domingos Reis, Adriano Abbud, Adriana Bugno
EPI_ISL_14584274, EPI_ISL_14584275, EPI_ISL_14584277, EPI_ISL_14584279, EPI_ISL_14584281, EPI_ISL_14584282, EPI_ISL_14584283, EPI_ISL_14584284, EPI_ISL_14584286, EPI_ISL_14584287, EPI_ISL_14584289, EPI_ISL_14584290, EPI_ISL_14584291, EPI_ISL_14584292, EPI_ISL_14584293, EPI_ISL_14584294, EPI_ISL_14584295, EPI_ISL_14584296, EPI_ISL_14584297, EPI_ISL_14584298, EPI_ISL_14584299, EPI_ISL_14584300, EPI_ISL_14584302, EPI_ISL_14584303, EPI_ISL_14584304, EPI_ISL_14584305, EPI_ISL_14584306, EPI_ISL_14584308, EPI_ISL_14584309, EPI_ISL_14584310, EPI_ISL_14584311			
see above	Laboratorio de Referencia Nacional de Virus Respiratorio. Centro Nacional de Salud Publica. Instituto Nacional de Salud.	Laboratorio de Referencia Nacional de Virus Respiratorio. Centro Nacional de Salud Publica. Instituto Nacional de Salud.	Carlos Padilla Rojas, Veronica Hurtado Vela, Iris Silva Molina, Luren Sevilla Castañeda, Victor Jimenez Vasquez, Orson Mestanza Millones, Luis Barcena Flores, Wendy Lizarraga Olivares, Alicia Nuñez Llanos, Steve Acedo Lazo, Francisco Ascue Orosco, Kelly Izarra Rojas, Princesa Medrano Alhuay, Karla Vasquez Cajachahua, Estela Huaman Angeles, Jorge Giraldo Chavez, Lilian Huarca Balbin, Lisbet Roxana Inga Angulo, Maria Sandra Villar Saavedra, Henri Bailon Calderon, Lely Solari Zerpa, Gloria Arotinco Garayar. Equipo de vigilancia genomica del Instituto Nacional de Salud.
EPI_ISL_14586688	Public Health Authority of the Slovak Republic	Laboratory of Genomics and Bioinformatics, Comenius University Science Park	Tomáš Szemes, Edita Staroňová, Elena Tichá, Lucia Ševčíková, Terézia Vrabľová, Tatiana Sedláčková, Miroslav Böhmer, Jaroslav Budiš, Pavol Mišenko
EPI_ISL_14587544, EPI_ISL_14587545, EPI_ISL_14587546, EPI_ISL_14587548, EPI_ISL_14587549, EPI_ISL_14587550	Public Health Agency of Canada, National Microbiology Laboratory	Public Health Agency of Canada, National Microbiology Laboratory	Duggan,A., Hole,D., Yadav,C., Knox,N., Tyler,A., Haidl,E., Chapel,M., Domselaar,G.V., Graham,M., Audet,J., Fernando,L., Hagan,M., Safronetz,D., Leung,A., Peters,G., Go,A., Laminman,V., Kaplen,B., Antonation,K., Griffiths,E., Jolly,G., Charest,H., Levade,I. and Fafard,J.
EPI_ISL_14594043, EPI_ISL_14594051, EPI_ISL_14594054, EPI_ISL_14594056	Public Health Agency of Canada, National Microbiology Laboratory	Public Health Agency of Canada, National Microbiology Laboratory	Duggan,A., Hole,D., Yadav,C., Knox,N., Tyler,A., Haidl,E., Chapel,M., Domselaar,G.V., Graham,M., Audet,J., Fernando,L., Antonation,K., Safronetz,D., Hagan,M., Peters,G., Go,A., Laminman,V., Kaplen,B., Leung,A., Griffiths,E., Jolly,G., Eshaghi,A., Gubbay,J.B., Hasso,M., Marchand-Austin,A., Olsha,R. and Patel,S.N.
EPI_ISL_14615579	RSUPN dr. Cipto Mangunkusumo	National Institute of Health Research and Development	Hana Apsari Pawestri, Arie Ardiansyah Nugraha, Fajar Nur Sulistiyohadi, Subangkit, Krisna NA Pangesti, Tze Minn Mak, I Gede Made Wirabrata
EPI_ISL_14621526	Virology, APHP Pitie Salpetriere SU	Virology, APHP Pitie Salpetriere SU	Seang,S., Burrel,S., Todesco,E., Leducq,V., Monsel,G., Le Pluart,D., Cordevant,C., Pourcher,V. and Palich,R.
EPI_ISL_14622055	Instituto de Infectologia Emilio Ribas	Instituto Adolfo Lutz Strategic Laboratory	Claudio Tavares Sacchi, Karoline Rodrigues Campos, Ariadne Ferreira Amarante, Marlon Benedito Nascimento Santos, Alex Domingos Reis, Adriano Abbud, Adriana Bugno
EPI_ISL_14622520	UBS Jovaia	Instituto Adolfo Lutz Strategic Laboratory	Claudio Tavares Sacchi, Karoline Rodrigues Campos, Ariadne Ferreira Amarante, Marlon Benedito Nascimento Santos, Alex Domingos Reis, Adriano Abbud, Adriana Bugno
EPI_ISL_14622705	UBS Jardim Santista	Instituto Adolfo Lutz Strategic Laboratory	Claudio Tavares Sacchi, Karoline Rodrigues Campos, Ariadne Ferreira Amarante, Marlon Benedito Nascimento Santos, Alex Domingos Reis, Adriano Abbud, Adriana Bugno
EPI_ISL_14622706	Centro de Referencia Modulo I SAE II Bauru	Instituto Adolfo Lutz Strategic Laboratory	Claudio Tavares Sacchi, Karoline Rodrigues Campos, Ariadne Ferreira Amarante, Marlon Benedito Nascimento Santos, Alex Domingos Reis, Adriano Abbud, Adriana Bugno
EPI_ISL_14622707	USF Boicucanga I Sao Sebastiao	Instituto Adolfo Lutz Strategic Laboratory	Claudio Tavares Sacchi, Karoline Rodrigues Campos, Ariadne Ferreira Amarante, Marlon Benedito Nascimento Santos, Alex Domingos Reis, Adriano Abbud, Adriana Bugno
EPI_ISL_14622913	Secretaria Municipal de Saude de Caxias do Sul	Instituto Adolfo Lutz Strategic Laboratory	Claudio Tavares Sacchi, Karoline Rodrigues Campos, Ariadne Ferreira Amarante, Marlon Benedito Nascimento Santos, Alex Domingos Reis, Adriano Abbud, Adriana Bugno
EPI_ISL_14622953	Sistema de Vigilancia em Saude Viamao	Instituto Adolfo Lutz Strategic Laboratory	Claudio Tavares Sacchi, Karoline Rodrigues Campos, Ariadne Ferreira Amarante, Marlon Benedito Nascimento Santos, Alex Domingos Reis, Adriano Abbud, Adriana Bugno
EPI_ISL_14622960	Vigilancia Epidemiologica Municipal	Instituto Adolfo Lutz Strategic Laboratory	Claudio Tavares Sacchi, Karoline Rodrigues Campos, Ariadne Ferreira Amarante, Marlon Benedito Nascimento Santos, Alex Domingos Reis, Adriano Abbud, Adriana Bugno
EPI_ISL_14623175	Centro de Referencia em Especialidades Central Rib Preto	Instituto Adolfo Lutz Strategic Laboratory	Claudio Tavares Sacchi, Karoline Rodrigues Campos, Ariadne Ferreira Amarante, Marlon Benedito Nascimento Santos, Alex Domingos Reis, Adriano Abbud, Adriana Bugno
EPI_ISL_14623523	Laboratorio Municipal de Piracicaba	Instituto Adolfo Lutz Strategic Laboratory	Claudio Tavares Sacchi, Karoline Rodrigues Campos, Ariadne Ferreira Amarante, Marlon Benedito Nascimento Santos, Alex Domingos Reis, Adriano Abbud, Adriana Bugno
EPI_ISL_14623704	Unidade Basica de Saude Esplanada	Instituto Adolfo Lutz Strategic Laboratory	Claudio Tavares Sacchi, Karoline Rodrigues Campos, Ariadne Ferreira Amarante, Marlon Benedito Nascimento Santos, Alex Domingos Reis, Adriano Abbud, Adriana Bugno
EPI_ISL_14624411	Hospital Albert Sabin Atibaia	Instituto Adolfo Lutz Strategic Laboratory	Claudio Tavares Sacchi, Karoline Rodrigues Campos, Ariadne Ferreira Amarante, Marlon Benedito Nascimento Santos, Alex Domingos Reis, Adriano Abbud, Adriana Bugno
EPI_ISL_14624610	USAFA Forte	Instituto Adolfo Lutz Strategic Laboratory	Claudio Tavares Sacchi, Karoline Rodrigues Campos, Ariadne Ferreira Amarante, Marlon Benedito Nascimento Santos, Alex Domingos Reis, Adriano Abbud, Adriana Bugno
EPI_ISL_14624698	Centro de Referencia em AIDS SECRAIDS	Instituto Adolfo Lutz Strategic Laboratory	Claudio Tavares Sacchi, Karoline Rodrigues Campos, Ariadne Ferreira Amarante, Marlon Benedito Nascimento Santos, Alex Domingos Reis, Adriano Abbud, Adriana Bugno
EPI_ISL_14624832	Servico de Vigilancia Epidemiologica e de Zoonoses do Guaruja	Instituto Adolfo Lutz Strategic Laboratory	Claudio Tavares Sacchi, Karoline Rodrigues Campos, Ariadne Ferreira Amarante, Marlon Benedito Nascimento Santos, Alex Domingos Reis, Adriano Abbud, Adriana Bugno
EPI_ISL_14625156	Secretaria Municipal de Saude de Suzano	Instituto Adolfo Lutz Strategic Laboratory	Claudio Tavares Sacchi, Karoline Rodrigues Campos, Ariadne Ferreira Amarante, Marlon Benedito Nascimento Santos, Alex Domingos Reis, Adriano Abbud, Adriana Bugno
EPI_ISL_14625157	PSF Vila Nossa Senhora de Fatima Fartura	Instituto Adolfo Lutz Strategic Laboratory	Claudio Tavares Sacchi, Karoline Rodrigues Campos, Ariadne Ferreira Amarante, Marlon Benedito Nascimento Santos, Alex Domingos Reis, Adriano Abbud, Adriana Bugno
EPI_ISL_14625190	Ambulatorio de Atendimentode DST de Guariba	Instituto Adolfo Lutz Strategic Laboratory	Claudio Tavares Sacchi, Karoline Rodrigues Campos, Ariadne Ferreira Amarante, Marlon Benedito Nascimento Santos, Alex Domingos Reis, Adriano Abbud, Adriana Bugno
EPI_ISL_14625230	UBS Centro Clair Aparecida Pavan	Instituto Adolfo Lutz Strategic Laboratory	Claudio Tavares Sacchi, Karoline Rodrigues Campos, Ariadne Ferreira Amarante, Marlon Benedito Nascimento Santos, Alex Domingos Reis, Adriano Abbud, Adriana Bugno
EPI_ISL_14625256	UMS Campina do Siqueira	Instituto Adolfo Lutz Strategic Laboratory	Claudio Tavares Sacchi, Karoline Rodrigues Campos, Ariadne Ferreira Amarante, Marlon Benedito Nascimento Santos, Alex Domingos Reis, Adriano Abbud, Adriana Bugno
EPI_ISL_14625282	Hospital Edmundo Vasconcelos	Instituto Adolfo Lutz Strategic Laboratory	Claudio Tavares Sacchi, Karoline Rodrigues Campos, Ariadne Ferreira Amarante, Marlon Benedito Nascimento Santos, Alex Domingos Reis, Adriano Abbud, Adriana Bugno
EPI_ISL_14666780	Public Health Authority of the Slovak Republic	Laboratory of Genomics and Bioinformatics, Comenius University Science Park	Tomáš Szemes, Edita Staroňová, Elena Tichá, Lucia Ševčíková, Terézia Vrabľová, Tatiana Sedláčková, Miroslav Böhmer, Jaroslav Budiš, Pavol Mišenko
EPI_ISL_14721255, EPI_ISL_14721259, EPI_ISL_14721262, EPI_ISL_14721265	National Public Health Laboratory, National Centre for Infectious Diseases	National Public Health Laboratory, National Centre for Infectious Diseases	Yichen Ding, Benny Yeo, Daniel Lim, Zhenyang Zhou, Royce Ang, Samuel Loo, Lin Cui, Raymond Tzer Pin Lin
EPI_ISL_14736400, EPI_ISL_14736402, EPI_ISL_14736403	California Department of Public Health	California Department of Public Health	Viral and Rickettsial Disease Laboratory
EPI_ISL_14752090, EPI_ISL_14752091, EPI_ISL_14752093, EPI_ISL_14752094, EPI_ISL_14752096	Environmental, Agricultural, and Occupational Health, University of Nebraska Medical Center	Environmental, Agricultural, and Occupational Health, University of Nebraska Medical Center	Tegomoh,B., Cross,S.T., Chapman,R.C., Bernhard,K., McCutchen,E.L., Fauver,J.R., Pratt,C.B., Warden,D.E., Iwen,P.C., Donahue,M. and Wiley,M.R.

EPI_ISL_14752216	Department of Infectious Diseases, National Institute of Health Doutor Ricardo Jorge (INSA)	Department of Infectious Diseases, National Institute of Health Doutor Ricardo Jorge (INSA)	Isidro,J., Borges,V., Pinto,M., Sobral,D., Santos,J., Nunes,A., Mixao,V., Ferreira,R., Santos,D., Duarte,S., Vieira,L., Borrego,M.J., Nuncio,S., Lopes de Carvalho,I., Pelerito,A., Cordeiro,R. and Gomes,J.P.
EPI_ISL_14752284	Research and Evaluation, UKHSA	Research and Evaluation, UKHSA	Grove,N., Osman,K.L., Lewandowski,K.S., Carter,D.P., Pullan,S.T., Myers,R., Vipond,R. and Chand,M.
EPI_ISL_14752290	Research and Evaluation, UKHSA	Research and Evaluation, UKHSA	Groves,N., Osman,K.L., Lewandowski,K.S., Carter,D.P., Pullan,S.T., Myers,R., Vipond,R. and Chand,M.
EPI_ISL_14752293	Medical Microbiology & Infection Prevention, Amsterdam Medical Centres location AMC	Medical Microbiology & Infection Prevention, Amsterdam Medical Centres location AMC	Welkers,M., Jonges,M., de Regt,M., Ooijevaar,R. and Wagemakers,A.
EPI_ISL_14772912	USF Jardim Oratorio	Instituto Adolfo Lutz Strategic Laboratory	Claudio Tavares Sacchi, Karoline Rodrigues Campos, Ariadne Ferreira Amarante, Marlon Benedito Nascimento Santos, Alex Domingos Reis, Adriano Abbud, Adriana Bugno
EPI_ISL_14772913	Vigilancia Epidemiologica Jardinopolis - SP	Instituto Adolfo Lutz Strategic Laboratory	Claudio Tavares Sacchi, Karoline Rodrigues Campos, Ariadne Ferreira Amarante, Marlon Benedito Nascimento Santos, Alex Domingos Reis, Adriano Abbud, Adriana Bugno
EPI_ISL_14772914	Pronto Atendimento Infantil e entral de Quimioterapia SJrpreto	Instituto Adolfo Lutz Strategic Laboratory	Claudio Tavares Sacchi, Karoline Rodrigues Campos, Ariadne Ferreira Amarante, Marlon Benedito Nascimento Santos, Alex Domingos Reis, Adriano Abbud, Adriana Bugno
EPI_ISL_14773001	CEDIC CTA	Instituto Adolfo Lutz Strategic Laboratory	Claudio Tavares Sacchi, Karoline Rodrigues Campos, Ariadne Ferreira Amarante, Marlon Benedito Nascimento Santos, Alex Domingos Reis, Adriano Abbud, Adriana Bugno
EPI_ISL_14783237	Sicilian Regional Laboratory - AOUP "P. Giaccone" - University of Palermo	Sicilian Regional Laboratory - AOUP "P. Giaccone" - University of Palermo	Fabio Tramuto, Carmelo Massimo Maida, Giulia Randazzo, Valeria Guzzetta, Walter Mazzucco, Giorgio Graziano, Vincenzo Restivo, Claudio Costantino, Francesco Vitale
EPI_ISL_14793992	Erasmus Medical Center Department of Virology	Erasmus Medical Center Department of Virology	Bas Oude Munnink, Leonard Schuele, Marjan Boter, Babette Weller, Babs Verstrepen, Richard Molenkamp, Janette Rahamat-Langendoen, Reina Sikkema, Marion Koopmans
EPI_ISL_14804638, EPI_ISL_14804639, EPI_ISL_14804640, EPI_ISL_14804641, EPI_ISL_14804642, EPI_ISL_14804643, EPI_ISL_14804644, EPI_ISL_14804645, EPI_ISL_14804646, EPI_ISL_14804647	Nebraska Public Health Laboratory	University of Nebraska Medical Center, Oklahoma Pathogen Genomics Consortium	Chapman,R.C., Bernhard,K., McCutchen,E.L., Fauver,J.R., O'Dell,J.X., Mannell,M., Wiley,M.R., Cross,S.T.
EPI_ISL_14809096	AMA Capao Redondo	Instituto Adolfo Lutz Strategic Laboratory	Claudio Tavares Sacchi, Karoline Rodrigues Campos, Ariadne Ferreira Amarante, Marlon Benedito Nascimento Santos, Alex Domingos Reis, Adriano Abbud, Adriana Bugno
EPI_ISL_14809098	Laboratorio Municipal de Piracicaba	Instituto Adolfo Lutz Strategic Laboratory	Claudio Tavares Sacchi, Karoline Rodrigues Campos, Ariadne Ferreira Amarante, Marlon Benedito Nascimento Santos, Alex Domingos Reis, Adriano Abbud, Adriana Bugno
EPI_ISL_14809099	Centro de Saude Gabriel de Lara	Instituto Adolfo Lutz Strategic Laboratory	Claudio Tavares Sacchi, Karoline Rodrigues Campos, Ariadne Ferreira Amarante, Marlon Benedito Nascimento Santos, Alex Domingos Reis, Adriano Abbud, Adriana Bugno
EPI_ISL_14809100	Secretaria Municipal da Saude de Joanopolis	Instituto Adolfo Lutz Strategic Laboratory	Claudio Tavares Sacchi, Karoline Rodrigues Campos, Ariadne Ferreira Amarante, Marlon Benedito Nascimento Santos, Alex Domingos Reis, Adriano Abbud, Adriana Bugno
EPI_ISL_14810404, EPI_ISL_14810405, EPI_ISL_14810406, EPI_ISL_14810407	Erasmus Medical Center Department of Virology	Erasmus Medical Center Department of Virology	Leonard Schuele, Bas Oude Munnink, Marjan Boter, Babette Weller, Babs Verstrepen, Richard Molenkamp, Janette Rahamat-Langendoen, Reina Sikkema, Marion Koopmans
EPI_ISL_14818585	Los Angeles County Public Health Laboratories	Los Angeles County Public Health Laboratories	P. Hemarajata et al.
EPI_ISL_14818783, EPI_ISL_14818784, EPI_ISL_14818785, EPI_ISL_14818786, EPI_ISL_14818787, EPI_ISL_14818788, EPI_ISL_14818789, EPI_ISL_14818790, EPI_ISL_14818791, EPI_ISL_14818792, EPI_ISL_14818793, EPI_ISL_14818794, EPI_ISL_14818795, EPI_ISL_14818796, EPI_ISL_14818797, EPI_ISL_14818798, EPI_ISL_14818799, EPI_ISL_14818800, EPI_ISL_14818801, EPI_ISL_14818802, EPI_ISL_14818803, EPI_ISL_14818804, EPI_ISL_14818805, EPI_ISL_14818806, EPI_ISL_14818807, EPI_ISL_14818808, EPI_ISL_14818809, EPI_ISL_14818810, EPI_ISL_14818811, EPI_ISL_14818812, EPI_ISL_14818813, EPI_ISL_14818814, EPI_ISL_14818815, EPI_ISL_14818817, EPI_ISL_14818818, EPI_ISL_14818819, EPI_ISL_14818820, EPI_ISL_14818821, EPI_ISL_14818822			
see above	Laboratorio de Referencia Nacional de Virus Inmunoprevenibles. Centro Nacional de Salud Publica. Instituto Nacional de Salud.	Laboratorio de Referencia Nacional de Virus Inmunoprevenibles. Centro Nacional de Salud Publica. Instituto Nacional de Salud.	Carlos Padilla Rojas, Veronica Hurtado Vela, Iris Silva Molina, Luren Sevilla Castañeda, Victor Jimenez Vasquez, Luis Barcena Flores, Alicia Nuñez Llanos, Kelly Izarra Rojas, Karla Vasquez Cajachahua, Estela Huaman Angeles, Jorge Giraldo Chavez, Lilian Huarca Balbin, Maria Sandra Villar Saavedra, Henri Bailon Calderon, Lely Solari Zerpa, Gloria Arotinco Garayar. Equipo de vigilancia genomica del Instituto Nacional de Salud.
EPI_ISL_14863041, EPI_ISL_14863042	Molecular Epidemiology, Idaho Bureau of Laboratories	Molecular Epidemiology, Idaho Bureau of Laboratories	Ceniseros,A.
EPI_ISL_14863048	MEPHI, IHU - Mediterranee Infection	MEPHI, IHU - Mediterranee Infection	Colson,P.
EPI_ISL_14863049	Molecular Epidemiology, Idaho Bureau of Laboratories	Molecular Epidemiology, Idaho Bureau of Laboratories	Ceniseros,A.
EPI_ISL_14863050, EPI_ISL_14863051, EPI_ISL_14863052, EPI_ISL_14863053, EPI_ISL_14863055, EPI_ISL_14863057, EPI_ISL_14863059	MEPHI, IHU - Mediterranee Infection	MEPHI, IHU - Mediterranee Infection	Colson,P.
EPI_ISL_14865785	UBS J COPA	Instituto Adolfo Lutz Strategic Laboratory	Claudio Tavares Sacchi, Karoline Rodrigues Campos, Ariadne Ferreira Amarante, Marlon Benedito Nascimento Santos, Alex Domingos Reis, Adriano Abbud, Adriana Bugno
EPI_ISL_14866481	PR S da Familia Unidade de Saude Adalberto Rocha	Instituto Adolfo Lutz Strategic Laboratory	Claudio Tavares Sacchi, Karoline Rodrigues Campos, Ariadne Ferreira Amarante, Marlon Benedito Nascimento Santos, Alex Domingos Reis, Adriano Abbud, Adriana Bugno
EPI_ISL_14866751	Pronto Socorro da Vila Dirce	Instituto Adolfo Lutz Strategic Laboratory	Claudio Tavares Sacchi, Karoline Rodrigues Campos, Ariadne Ferreira Amarante, Marlon Benedito Nascimento Santos, Alex Domingos Reis, Adriano Abbud, Adriana Bugno
EPI_ISL_14866752	Secretaria Municipal de Saude Sao Carlos	Instituto Adolfo Lutz Strategic Laboratory	Claudio Tavares Sacchi, Karoline Rodrigues Campos, Ariadne Ferreira Amarante, Marlon Benedito Nascimento Santos, Alex Domingos Reis, Adriano Abbud, Adriana Bugno
EPI_ISL_14910886	Research and Evaluation, UKHSA	Research and Evaluation, UKHSA	Burton,J., Easterbrook,L., Drinkwater,E., Groves,N., Osman,K.L., Lewandowski,K.S., Carter,D., Pullan,S.T., Myers,R., Vipond,R. and Chand,M.
EPI_ISL_14917580, EPI_ISL_14917581	Los Angeles County Public Health Laboratories	Los Angeles County Public Health Laboratories	P. Hemarajata et al.
EPI_ISL_14934116	Medical Center of Vienna Center for Virology	Medical University of Vienna Center for Virology	Jeremy V. Camp, Monika Redlberger-Fritz, Stephan W. Aberle
EPI_ISL_14934140	Center for Virology Medical Unviersity of Vienna	Medical University of Vienna Center for Virology	Jeremy V. Camp, Monika Redlberger-Fritz, Stephan W. Aberle
EPI_ISL_14934382	Medical University of Vienna Center for Virology	Medical University of Vienna Center for Virology	Jeremy V. Camp, Monika Redlberger-Fritz, Stephan W. Aberle
EPI_ISL_14934478	Medical University of Vienna Center for Virology	Medical University of Vienna Center for Virology	Jeremy V. Camp, Monika Redlberg-Fritz, Stephan W. Aberle
EPI_ISL_14934529, EPI_ISL_14934538, EPI_ISL_14934540, EPI_ISL_14934543, EPI_ISL_14934550, EPI_ISL_14934574, EPI_ISL_14934587	Department of Infectious Diseases, National Institute of Health Doutor Ricardo Jorge, Portugal (INSA)	Department of Infectious Diseases, National Institute of Health Doutor Ricardo Jorge, Portugal (INSA)	Isidro,J., Borges,V., Pinto,M., Sobral,D., Santos,J., Nunes,A., Mixao,V., Ferreira,R., Santos,D., Duarte,S., Vieira,L., Borrego,M.J., Nuncio,S., Lopes de Carvalho,I., Pelerito,A., Cordeiro,R. and Gomes,J.P.
EPI_ISL_14961089, EPI_ISL_14961090	Public Health Authority of the Slovak Republic	Laboratory of Genomics and Bioinformatics, Comenius University Science Park	Tomáš Szemes, Edita Staroňová, Elena Tichá, Lucia Ševčíková, Terézia Vrabľová, Tatiana Sedláčková, Miroslav Böhmer, Jaroslav Budiš, Pavol Mišenko
EPI_ISL_14977306, EPI_ISL_14977307	Environmental, Agricultural, and Occupational Health, University of Nebraska Medical Center	Environmental, Agricultural, and Occupational Health, University of Nebraska Medical Center	Tegomoh,B., Cross,S.T., Chapman,R.C., Bernhard,K., McCutchen,E.L., Fauver,J.R., Pratt,C.B., Warden,D.E., Iwen,P.C., Donahue,M. and Wiley,M.R.
EPI_ISL_14995206	Pronto Socorro Municiplal de Cravinhos	Instituto Adolfo Lutz Strategic Laboratory	Claudio Tavares Sacchi, Karoline Rodrigues Campos, Ariadne Ferreira Amarante, Marlon Benedito Nascimento Santos, Alex Domingos Reis, Adriano Abbud, Adriana Bugno
EPI_ISL_14995578	Hosp. Municipal de Ilhabela Gov. Mario Covas Jr.	Instituto Adolfo Lutz Strategic Laboratory	Claudio Tavares Sacchi, Karoline Rodrigues Campos, Ariadne Ferreira Amarante, Marlon Benedito Nascimento Santos, Alex Domingos Reis, Adriano Abbud, Adriana Bugno
EPI_ISL_14995579	Secretaria Municipal de Saude de Feira de Santana	Instituto Adolfo Lutz Strategic Laboratory	Claudio Tavares Sacchi, Karoline Rodrigues Campos, Ariadne Ferreira Amarante, Marlon Benedito Nascimento Santos, Alex Domingos Reis, Adriano Abbud, Adriana Bugno
EPI_ISL_14995580	UBS Alexander Fleming Simioni	Instituto Adolfo Lutz Strategic Laboratory	Claudio Tavares Sacchi, Karoline Rodrigues Campos, Ariadne Ferreira Amarante, Marlon Benedito Nascimento Santos, Alex Domingos Reis, Adriano Abbud, Adriana Bugno
EPI_ISL_14995582	Hosp. Municipla. Dr. Jose de Carvalho Florence	Instituto Adolfo Lutz Strategic Laboratory	Claudio Tavares Sacchi, Karoline Rodrigues Campos, Ariadne Ferreira Amarante, Marlon Benedito Nascimento Santos, Alex Domingos Reis, Adriano Abbud, Adriana Bugno
EPI_ISL_14995585	Pronto Socorro Municipal do Promorar	Instituto Adolfo Lutz Strategic Laboratory	Claudio Tavares Sacchi, Karoline Rodrigues Campos, Ariadne Ferreira Amarante, Marlon Benedito Nascimento Santos, Alex Domingos Reis, Adriano Abbud, Adriana Bugno
EPI_ISL_14995586	UPA Centro	Instituto Adolfo Lutz Strategic Laboratory	Claudio Tavares Sacchi, Karoline Rodrigues Campos, Ariadne Ferreira Amarante, Marlon Benedito Nascimento Santos, Alex Domingos Reis, Adriano Abbud, Adriana Bugno
EPI_ISL_14995587	Centro de Saude 24 horas	Instituto Adolfo Lutz Strategic Laboratory	Claudio Tavares Sacchi, Karoline Rodrigues Campos, Ariadne Ferreira Amarante, Marlon Benedito Nascimento Santos, Alex Domingos Reis, Adriano Abbud, Adriana Bugno
EPI_ISL_14995591	Instituto de Infectologia Emilio Ribas	Instituto Adolfo Lutz Strategic Laboratory	Claudio Tavares Sacchi, Karoline Rodrigues Campos, Ariadne Ferreira Amarante, Marlon Benedito Nascimento Santos, Alex Domingos Reis, Adriano Abbud, Adriana Bugno
EPI_ISL_14995593	SAE DST / Aids Ipiranga Jose Francisco Araujo	Instituto Adolfo Lutz Strategic Laboratory	Claudio Tavares Sacchi, Karoline Rodrigues Campos, Ariadne Ferreira Amarante, Marlon Benedito Nascimento Santos, Alex Domingos Reis, Adriano Abbud, Adriana Bugno
EPI_ISL_14995611	UBS Horto Florestal	Instituto Adolfo Lutz Strategic Laboratory	Claudio Tavares Sacchi, Karoline Rodrigues Campos, Ariadne Ferreira Amarante, Marlon Benedito Nascimento Santos, Alex Domingos Reis, Adriano Abbud, Adriana Bugno
EPI_ISL_14995612	Secretaria Minicipal de Saude de IRECE	Instituto Adolfo Lutz Strategic Laboratory	Claudio Tavares Sacchi, Karoline Rodrigues Campos, Ariadne Ferreira Amarante, Marlon Benedito Nascimento Santos, Alex Domingos Reis, Adriano Abbud, Adriana Bugno
EPI_ISL_14995619	Hosp. Tereza de Lisieux	Instituto Adolfo Lutz Strategic Laboratory	Claudio Tavares Sacchi, Karoline Rodrigues Campos, Ariadne Ferreira Amarante, Marlon Benedito Nascimento Santos, Alex Domingos Reis, Adriano Abbud, Adriana Bugno
EPI_ISL_14995622	UBS Parque Meia Lua	Instituto Adolfo Lutz Strategic Laboratory	Claudio Tavares Sacchi, Karoline Rodrigues Campos, Ariadne Ferreira Amarante, Marlon Benedito Nascimento Santos, Alex Domingos Reis, Adriano Abbud, Adriana Bugno
EPI_ISL_14995653	Unidade Basica de Saude Vila Cristina	Instituto Adolfo Lutz Strategic Laboratory	Claudio Tavares Sacchi, Karoline Rodrigues Campos, Ariadne Ferreira Amarante, Marlon Benedito Nascimento Santos, Alex Domingos Reis, Adriano Abbud, Adriana Bugno
EPI_ISL_14995723	Unidade Mista de Atendimento Infantil Carapicuiba	Instituto Adolfo Lutz Strategic Laboratory	Claudio Tavares Sacchi, Karoline Rodrigues Campos, Ariadne Ferreira Amarante, Marlon Benedito Nascimento Santos, Alex Domingos Reis, Adriano Abbud, Adriana Bugno
EPI_ISL_14995724	Hosp. Carlos Chagas	Instituto Adolfo Lutz Strategic Laboratory	Claudio Tavares Sacchi, Karoline Rodrigues Campos, Ariadne Ferreira Amarante, Marlon Benedito Nascimento Santos, Alex Domingos Reis, Adriano Abbud, Adriana Bugno
EPI_ISL_15005641	Chongqing Municipal Center for Disease Control and Prevention	Chongqing Municipal Center for Disease Control and Prevention	Sheng Ye, Yun Tang, Shuang Chen, Mingyue Wang, Zhangping Tan, Zhen Yu
EPI_ISL_15055820	Sicilian Regional Laboratory - AOUP "P. Giaccone" - University of Palermo	Sicilian Regional Laboratory - AOUP "P. Giaccone" - University of Palermo	Fabio Tramuto, Carmelo Massimo Maida, Giulia Randazzo, Valeria Guzzetta, Walter Mazzucco, Giorgio Graziano, Vincenzo Restivo, Claudio Costantino, Francesco Vitale
EPI_ISL_15076130, EPI_ISL_15076131	Environmental, Agricultural, and Occupational Health, University of Nebraska Medical Center	Environmental, Agricultural, and Occupational Health, University of Nebraska Medical Center	Chapman,R.C., Bernhard,K., McCutchen,E.L., Fauver,J.R., O'Dell,J.X., Mannell,M., Wiley,M.R. and Cross,S.T.
EPI_ISL_15076180, EPI_ISL_15076183, EPI_ISL_15076188	Department of Genetics, University of North Carolina at Chapel Hill	Department of Genetics, University of North Carolina at Chapel Hill	Deanhardt,B., Miller,M. and Wang,J.R.
EPI_ISL_15104903	Institute for Virology, Philipps-University Marburg	Institute for Virology, Philipps-University Marburg	Eickmann, M., Lier, C., Kowalski, K., Kraft, F., Becker, S.
EPI_ISL_15120452, EPI_ISL_15120480, EPI_ISL_15120496	Los Angeles County Public Health Laboratories	Los Angeles County Public Health Laboratories	P. Hemarajata et al.

EPI_ISL_15158315, EPI_ISL_15158316, EPI_ISL_15158336, EPI_ISL_15158341, EPI_ISL_15158358, EPI_ISL_15158360, EPI_ISL_15158361, EPI_ISL_15158363, EPI_ISL_15158364, EPI_ISL_15158367, EPI_ISL_15158369, EPI_ISL_15158371, EPI_ISL_15158374, EPI_ISL_15158384, EPI_ISL_15158385, EPI_ISL_15158387, EPI_ISL_15158390, EPI_ISL_15158391, EPI_ISL_15158394, EPI_ISL_15158395, EPI_ISL_15158398			
see above	Molecular Biology, Microbiology, and Biochemistry, Southern Illinois University	Molecular Biology, Microbiology, and Biochemistry, Southern Illinois University	Gagnon,K.T.
EPI_ISL_15165603, EPI_ISL_15165604, EPI_ISL_15165608, EPI_ISL_15165614, EPI_ISL_15165618	Centro de Desenvolvimento Científico e Tecnológico (CDCT), Centro Estadual de Vigilância em Saúde (CEVS) da Secretaria Estadual da Saúde (SES-RS)	Centro de Desenvolvimento Científico e Tecnológico (CDCT), Centro Estadual de Vigilância em Saúde (CEVS) da Secretaria Estadual da Saúde (SES-RS)	Richard Steiner Salvato, Fernanda Marques Godinho, Regina Bones Barcellos, Patricia Sesterheim, Amanda Pellenz Ruivo, Viviane Horn de Melo, Júlio Augusto Schroder
EPI_ISL_15199671, EPI_ISL_15199672, EPI_ISL_15199682, EPI_ISL_15199706, EPI_ISL_15199727, EPI_ISL_15199738, EPI_ISL_15199739, EPI_ISL_15199743, EPI_ISL_15199776, EPI_ISL_15199795, EPI_ISL_15199796			
see above	Department of Infectious Diseases, National Institute of Health Doutor Ricardo Jorge, Portugal (INSA)	Department of Infectious Diseases, National Institute of Health Doutor Ricardo Jorge, Portugal (INSA)	Isidro,J., Borges,V., Pinto,M., Sobral,D., Santos,J., Nunes,A., Mixao,V., Ferreira,R., Santos,D., Duarte,S., Vieira,L., Borrego,M.J., Nuncio,S., Lopes de Carvalho,I., Pelerito,A., Cordeiro,R. and Gomes,J.P.
EPI_ISL_15247221	Medical University of Vienna Center for Virology	Medical University of Vienna Center for Virology	Jeremy V. Camp, Monika Redlberger-Fritz, Stephan W. Aberle
EPI_ISL_15257669	Public Health Agency of Canada, National Microbiology Laboratory	Public Health Agency of Canada, National Microbiology Laboratory	Duggan,A., Hole,D., Yadav,C., Knox,N., Haidl,E., Chapel,M., Tyler,A.D., Domselaar,G.V., Graham,M., Audet,J., Fernando,L., Hagan,M., Safronetz,D., Leung,A., Peters,G., Go,A., Kaplen,B., Antonation,K., Laminman,V., Jolly,G., Croxen,M., Deo,A., Dieu,P., Dong,X., Gill,K., Granger,D., Ferrato,C., Ikkurti,V., Kanji,J., Koleva,P., Li,V., Lloyd,C., Lynch,T., Ma,R., Pabbaraju,K., Rotich,S., Sergeant,H., Skitsko,T., Tipples,G., Thayer,J., Shideler,S. and Wong,A.
EPI_ISL_15257681, EPI_ISL_15257682, EPI_ISL_15257687	Viral and Rickettsial Disease Laboratory, California Department of Public Health	Viral and Rickettsial Disease Laboratory, California Department of Public Health	Probert,W., Espinosa,A., Kath,C., Haw,M., O'Neil,R., Bell,J. and Hacker,J.
EPI_ISL_15263355	Sicilian Regional Laboratory - AOUP "P. Giaccone" - University of Palermo	Sicilian Regional Laboratory - AOUP "P. Giaccone" - University of Palermo	Fabio Tramuto, Carmelo Massimo Maida, Giulia Randazzo, Valeria Guzzetta, Walter Mazzucco, Giorgio Graziano, Vincenzo Restivo, Claudio Costantino, Francesco Vitale
EPI_ISL_15269699, EPI_ISL_15269702, EPI_ISL_15269704	Erasmus Medical Center Department of Virology	Erasmus Medical Center Department of Virology	Leonard Schuele, Bas Oude Munnink, Marjan Boter, Babette Weller, Babs Verstrepen, Richard Molenkamp, Janette Rahamat-Langendoen, Reina Sikkema, Marion Koopmans
EPI_ISL_15293815	National Institute for Viral Disease Control and Prevention (IVDC), Chinese Center for Disease Control and Prevention , Beijing, China	National Institute for Viral Disease Control and Prevention (IVDC), Chinese Center for Disease Control and Prevention , Beijing, China	Wenjie Tan, Changcheng Wu, Ruhan A, Wenling Wang, Roujian Lu, Li Zhao, Baoying Huang, Fei Ye, Wenbo Xu
EPI_ISL_15332338, EPI_ISL_15332339, EPI_ISL_15332340	Environmental, Agricultural, and Occupational Health, University of Nebraska Medical Center	Environmental, Agricultural, and Occupational Health, University of Nebraska Medical Center	Chapman,R.C., Bernhard,K., McCutchen,E.L., Fauver,J.R., O'Dell,J.X., Mannell,M., Wiley,M.R. and Cross,S.T.
EPI_ISL_15380492	Eastwood Medical City	Molecular Biology Laboratory, Research Institute for Tropical Medicine	Samantha Louise P. Bado, Niquitta B. Galap, Bea C. Mateo, Chelsea Mae M. Reyes, Amalea Dulcene Nicolasora, Miguel Francisco B. Abulencia, Francisco Gerardo M. Polotan on behalf of the Research Institute for Tropical Medicine
EPI_ISL_15419131	Instituto de Infectologia Emilio Ribas	Instituto Adolfo Lutz Strategic Laboratory	Claudio Tavares Sacchi, Karoline Rodrigues Campos, Ariadne Ferreira Amarante, Marlon Benedito Nascimento Santos, Adriano Abbud, Adriana Bugno
EPI_ISL_15419133	UBS II COHAB Presidente Prudente	Instituto Adolfo Lutz Strategic Laboratory	Claudio Tavares Sacchi, Karoline Rodrigues Campos, Ariadne Ferreira Amarante, Marlon Benedito Nascimento Santos, Adriano Abbud, Adriana Bugno
EPI_ISL_15419134	CTA Centro de Testagem e Aconselhamento de Caieiras	Instituto Adolfo Lutz Strategic Laboratory	Claudio Tavares Sacchi, Karoline Rodrigues Campos, Ariadne Ferreira Amarante, Marlon Benedito Nascimento Santos, Adriano Abbud, Adriana Bugno
EPI_ISL_15419135	SAE DST AIDS Cidade Dutra	Instituto Adolfo Lutz Strategic Laboratory	Claudio Tavares Sacchi, Karoline Rodrigues Campos, Ariadne Ferreira Amarante, Marlon Benedito Nascimento Santos, Adriano Abbud, Adriana Bugno
EPI_ISL_15419136	UBS J Nordeste	Instituto Adolfo Lutz Strategic Laboratory	Claudio Tavares Sacchi, Karoline Rodrigues Campos, Ariadne Ferreira Amarante, Marlon Benedito Nascimento Santos, Adriano Abbud, Adriana Bugno
EPI_ISL_15419137	CTA Centro de Testagem e Aconselhamento Favo de Mel	Instituto Adolfo Lutz Strategic Laboratory	Claudio Tavares Sacchi, Karoline Rodrigues Campos, Ariadne Ferreira Amarante, Marlon Benedito Nascimento Santos, Adriano Abbud, Adriana Bugno
EPI_ISL_15419138	Vigilancia Epidemiologica e Controle de Vetores de Pirassununga	Instituto Adolfo Lutz Strategic Laboratory	Claudio Tavares Sacchi, Karoline Rodrigues Campos, Ariadne Ferreira Amarante, Marlon Benedito Nascimento Santos, Adriano Abbud, Adriana Bugno
EPI_ISL_15419140	Secretaria Municipal de Saude de Batatais SP	Instituto Adolfo Lutz Strategic Laboratory	Claudio Tavares Sacchi, Karoline Rodrigues Campos, Ariadne Ferreira Amarante, Marlon Benedito Nascimento Santos, Adriano Abbud, Adriana Bugno
EPI_ISL_15419141	UBS J Nordeste	Instituto Adolfo Lutz Strategic Laboratory	Claudio Tavares Sacchi, Karoline Rodrigues Campos, Ariadne Ferreira Amarante, Marlon Benedito Nascimento Santos, Adriano Abbud, Adriana Bugno
EPI_ISL_15419142	Instituto de Infectologia Emilio Ribas	Instituto Adolfo Lutz Strategic Laboratory	Claudio Tavares Sacchi, Karoline Rodrigues Campos, Ariadne Ferreira Amarante, Marlon Benedito Nascimento Santos, Adriano Abbud, Adriana Bugno
EPI_ISL_15419143	Santa Casa de Barretos	Instituto Adolfo Lutz Strategic Laboratory	Claudio Tavares Sacchi, Karoline Rodrigues Campos, Ariadne Ferreira Amarante, Marlon Benedito Nascimento Santos, Adriano Abbud, Adriana Bugno
EPI_ISL_15419144	Hospital Vera Cruz	Instituto Adolfo Lutz Strategic Laboratory	Claudio Tavares Sacchi, Karoline Rodrigues Campos, Ariadne Ferreira Amarante, Marlon Benedito Nascimento Santos, Adriano Abbud, Adriana Bugno
EPI_ISL_15419145	NotreDame Intermedica Saude	Instituto Adolfo Lutz Strategic Laboratory	Claudio Tavares Sacchi, Karoline Rodrigues Campos, Ariadne Ferreira Amarante, Marlon Benedito Nascimento Santos, Adriano Abbud, Adriana Bugno
EPI_ISL_15419146	Hospital e Maternidade Santa Maria Cruz Azul	Instituto Adolfo Lutz Strategic Laboratory	Claudio Tavares Sacchi, Karoline Rodrigues Campos, Ariadne Ferreira Amarante, Marlon Benedito Nascimento Santos, Adriano Abbud, Adriana Bugno
EPI_ISL_15419147	Pronto Socorro Central de Diadema	Instituto Adolfo Lutz Strategic Laboratory	Claudio Tavares Sacchi, Karoline Rodrigues Campos, Ariadne Ferreira Amarante, Marlon Benedito Nascimento Santos, Adriano Abbud, Adriana Bugno
EPI_ISL_15419148	NotreDame Intermedica Saude Santo Andre	Instituto Adolfo Lutz Strategic Laboratory	Claudio Tavares Sacchi, Karoline Rodrigues Campos, Ariadne Ferreira Amarante, Marlon Benedito Nascimento Santos, Adriano Abbud, Adriana Bugno
EPI_ISL_15419151	UPA 24H Brotas	Instituto Adolfo Lutz Strategic Laboratory	Claudio Tavares Sacchi, Karoline Rodrigues Campos, Ariadne Ferreira Amarante, Marlon Benedito Nascimento Santos, Adriano Abbud, Adriana Bugno
EPI_ISL_15419152	Hospital Nossa Senhora de Lourdes	Instituto Adolfo Lutz Strategic Laboratory	Claudio Tavares Sacchi, Karoline Rodrigues Campos, Ariadne Ferreira Amarante, Marlon Benedito Nascimento Santos, Adriano Abbud, Adriana Bugno
EPI_ISL_15419153	AMA Paraisopolis	Instituto Adolfo Lutz Strategic Laboratory	Claudio Tavares Sacchi, Karoline Rodrigues Campos, Ariadne Ferreira Amarante, Marlon Benedito Nascimento Santos, Adriano Abbud, Adriana Bugno
EPI_ISL_15419154	Pronto Atendimento Infantil e Central de Quimioterapia de Sao Jose do Rio Preto	Instituto Adolfo Lutz Strategic Laboratory	Claudio Tavares Sacchi, Karoline Rodrigues Campos, Ariadne Ferreira Amarante, Marlon Benedito Nascimento Santos, Adriano Abbud, Adriana Bugno
EPI_ISL_15419155	Santa Casa de Atibaia Pro Saude	Instituto Adolfo Lutz Strategic Laboratory	Claudio Tavares Sacchi, Karoline Rodrigues Campos, Ariadne Ferreira Amarante, Marlon Benedito Nascimento Santos, Adriano Abbud, Adriana Bugno
EPI_ISL_15419156	UPA Vila Mariana	Instituto Adolfo Lutz Strategic Laboratory	Claudio Tavares Sacchi, Karoline Rodrigues Campos, Ariadne Ferreira Amarante, Marlon Benedito Nascimento Santos, Adriano Abbud, Adriana Bugno
EPI_ISL_15419157	Secretaria de Saude de Mogi das Cruzes	Instituto Adolfo Lutz Strategic Laboratory	Claudio Tavares Sacchi, Karoline Rodrigues Campos, Ariadne Ferreira Amarante, Marlon Benedito Nascimento Santos, Adriano Abbud, Adriana Bugno
EPI_ISL_15419158	Unidade Mista de saude Mariano Gayoso Castelo Branco	Instituto Adolfo Lutz Strategic Laboratory	Claudio Tavares Sacchi, Karoline Rodrigues Campos, Ariadne Ferreira Amarante, Marlon Benedito Nascimento Santos, Adriano Abbud, Adriana Bugno
EPI_ISL_15419161	Centro de Saude I Albertino Affonso Jaboticabal	Instituto Adolfo Lutz Strategic Laboratory	Claudio Tavares Sacchi, Karoline Rodrigues Campos, Ariadne Ferreira Amarante, Marlon Benedito Nascimento Santos, Adriano Abbud, Adriana Bugno
EPI_ISL_15419162	SAE DST AIDS M Boi Mirim Servico de Atencao Especializada	Instituto Adolfo Lutz Strategic Laboratory	Claudio Tavares Sacchi, Karoline Rodrigues Campos, Ariadne Ferreira Amarante, Marlon Benedito Nascimento Santos, Adriano Abbud, Adriana Bugno
EPI_ISL_15458903, EPI_ISL_15458904, EPI_ISL_15458905, EPI_ISL_15458906	Nebraska Public Health Laboratory (NPHL)	Environmental, Agricultural, and Occupational Health, University of Nebraska Medical Center	Chapman,R.C., Bernhard,K., McCutchen,E.L., Fauver,J.R., O'Dell,J.X., Mannell,M., Wiley,M.R. and Cross,S.T.
EPI_ISL_15528148	Department of Clinical Sciences, Institute of Tropica Medicine	Department of Clinical Sciences, Institute of Tropica Medicine	Berens-Riha,N., De Block,T., Rutgers,J., Van Gestel,L., Hens,M., Kenyon,C., Soentjens,P., Van Griensven,J., Brosius,I., Arien,K., Van Esbroeck,M., Rezende,A.M., Vercauteren,K. and Liesenborghs,L.
EPI_ISL_15528151	Department of Clinical Sciences, Institute of Tropica Medicine	Department of Clinical Sciences, Institute of Tropica Medicine	Berens-Riha,N., De Block,T., Rutgers,J., Van Gestel,L., Hens,M., Kenyon,C., Soentjens,P., Van Griensven,J., Brosius,I., Arien,K., Van Esbroeck,M., Rezende,A.M. and Vercauteren,K.
EPI_ISL_15593717	LESP State of Mexico	Instituto de Diagnostico y Referencia Epidemiologicos (INDRE)	Abril Rodríguez-Maldonado; Claudia Wong-Arámbula; Felipe Arguijo-Perez; Helios Cárdenas-Hernández; Carmen Castro-Méndez; Lidia García-Torres; Ruth Madera-Sandoval; América Mandujano-Martínez; Nancy Martínez-Velázquez; Mireya Mederos-Michel; Angélica Pedraza-Meléndez; Joaquín Quiroz-Mercado; Daniel Regalado-Santiago; Silvia Rivero-Arredondo; Erika Sierra-Atanacio; Fernando González-Domínguez; Lucia Hernández-Rivas, Irma López-Martínez; Ernesto Ramírez-González; Maribel González-Villa
EPI_ISL_15593719	LESP Puebla	Instituto de Diagnostico y Referencia Epidemiologicos (INDRE)	Abril Rodríguez-Maldonado; Claudia Wong-Arámbula; Felipe Arguijo-Perez; Helios Cárdenas-Hernández; Carmen Castro-Méndez; Lidia García-Torres; Ruth Madera-Sandoval; América Mandujano-Martínez; Nancy Martínez-Velázquez; Mireya Mederos-Michel; Angélica Pedraza-Meléndez; Joaquín Quiroz-Mercado; Daniel Regalado-Santiago; Silvia Rivero-Arredondo; Erika Sierra-Atanacio; Fernando González-Domínguez; Lucia Hernández-Rivas, Irma López-Martínez; Ernesto Ramírez-González; Maribel González-Villa
EPI_ISL_15593720	LESP Tamaulipas	Instituto de Diagnostico y Referencia Epidemiologicos (INDRE)	Abril Rodríguez-Maldonado; Claudia Wong-Arámbula; Felipe Arguijo-Perez; Helios Cárdenas-Hernández; Carmen Castro-Méndez; Lidia García-Torres; Ruth Madera-Sandoval; América Mandujano-Martínez; Nancy Martínez-Velázquez; Mireya Mederos-Michel; Angélica Pedraza-Meléndez; Joaquín Quiroz-Mercado; Daniel Regalado-Santiago; Silvia Rivero-Arredondo; Erika Sierra-Atanacio; Fernando González-Domínguez; Lucia Hernández-Rivas, Irma López-Martínez; Ernesto Ramírez-González; Maribel González-Villa
EPI_ISL_15593721	LESP Baja California	Instituto de Diagnostico y Referencia Epidemiologicos (INDRE)	Abril Rodríguez-Maldonado; Claudia Wong-Arámbula; Felipe Arguijo-Perez; Helios Cárdenas-Hernández; Carmen Castro-Méndez; Lidia García-Torres; Ruth Madera-Sandoval; América Mandujano-Martínez; Nancy Martínez-Velázquez; Mireya Mederos-Michel; Angélica Pedraza-Meléndez; Joaquín Quiroz-Mercado; Daniel Regalado-Santiago; Silvia Rivero-Arredondo; Erika Sierra-Atanacio; Fernando González-Domínguez; Lucia Hernández-Rivas, Irma López-Martínez; Ernesto Ramírez-González; Maribel González-Villa
EPI_ISL_15593722	LESP Nuevo Leon	Instituto de Diagnostico y Referencia Epidemiologicos (INDRE)	Abril Rodríguez-Maldonado; Claudia Wong-Arámbula; Felipe Arguijo-Perez; Helios Cárdenas-Hernández; Carmen Castro-Méndez; Lidia García-Torres; Ruth Madera-Sandoval; América Mandujano-Martínez; Nancy Martínez-Velázquez; Mireya Mederos-Michel; Angélica Pedraza-Meléndez; Joaquín Quiroz-Mercado; Daniel Regalado-Santiago; Silvia Rivero-Arredondo; Erika Sierra-Atanacio; Fernando González-Domínguez; Lucia Hernández-Rivas, Irma López-Martínez; Ernesto Ramírez-González; Maribel González-Villa
EPI_ISL_15608909	Southern Nevada Public Health Laboratory	Southern Nevada Public Health Laboratory	Michael Picker
EPI_ISL_15641543, EPI_ISL_15641550, EPI_ISL_15641551, EPI_ISL_15641553, EPI_ISL_15641555, EPI_ISL_15641557, EPI_ISL_15641560, EPI_ISL_15641561, EPI_ISL_15641562, EPI_ISL_15641564, EPI_ISL_15641565, EPI_ISL_15641566, EPI_ISL_15641568, EPI_ISL_15641570, EPI_ISL_15641571, EPI_ISL_15641572, EPI_ISL_15641574, EPI_ISL_15641578, EPI_ISL_15641579, EPI_ISL_15641580, EPI_ISL_15641581, EPI_ISL_15641582, EPI_ISL_15641583, EPI_ISL_15641584, EPI_ISL_15641585, EPI_ISL_15641586, EPI_ISL_15641588, EPI_ISL_15641589, EPI_ISL_15641590, EPI_ISL_15641591, EPI_ISL_15641592, EPI_ISL_15641593, EPI_ISL_15641595, EPI_ISL_15641596, EPI_ISL_15641598, EPI_ISL_15641599, EPI_ISL_15641601, EPI_ISL_15641603, EPI_ISL_15641605, EPI_ISL_15641606, EPI_ISL_15641608			
see above	Public Health Laboratory, Public Health Service Amsterdam, The Netherlands	Department of Medical Microbiology & Infection prevention, Amsterdam University Medical Centers location AMC	Matthijs Welkers, Jelle Koopsen, Robin van Houdt, Marcel Jonges, Sebastien Matamoros, Sjoerd Rebers, Fokla Zorgdrager, Sylvia Bruisten, Akke Cornelissen, Janke Schinkel, Ewout Fanoy, Roisin Bavalia, Menno de Jong and Mariken van der Lubben on behalf of the Amsterdam Regional Genomic epidemiology and Outbreak Surveillance (ARGOS) consortium
EPI_ISL_15655944	Erasmus Medical Center Department of Virology	Erasmus Medical Center Department of Virology	Leonard Schuele, Bas Oude Munnink, Marjan Boter, Babette Weller, Babs Verstrepen, Richard Molenkamp, Janette Rahamat-Langendoen, Reina Sikkema, Marion Koopmans

EPI_ISL_15684645	Laboratory of Virology, University Hospitals of Geneva	Laboratory of Virology, University Hospitals of Geneva	Laubscher,F., Marques-Melancia,S., Cordey,S., Schibler,M.,Kaiser,L. and Renzoni,A.
EPI_ISL_15714288, EPI_ISL_15715292	Erasmus Medical Center Department of Virology	Erasmus Medical Center Department of Virology	Leonard Schuele, Bas Oude Munnink, Marjan Boter, Babette Weller, Babs Verstrepen, Richard Molenkamp, Janette Rahamat-Langendoen, Reina Sikkema, Marion Koopmans
EPI_ISL_15722681, EPI_ISL_15722682, EPI_ISL_15722684, EPI_ISL_15722685, EPI_ISL_15722686, EPI_ISL_15722687	Rush University Medical Center	RIPHL at Rush University Medical Center	Stefan Green, Kevin Kunstman, Hannah Barbian, Felix Araujo Perez, Edith Perez, Sofiya Bobrovska, Alyse Kittner, Cecilia Chau, Giancarlo Balangue, Lok Yiu Ashley Wu, Mary Hayden, Joyce Houlihan, Diane Springer, Nicholas Moore
EPI_ISL_15763810, EPI_ISL_15763811, EPI_ISL_15763812, EPI_ISL_15763813, EPI_ISL_15763814, EPI_ISL_15763815, EPI_ISL_15763817, EPI_ISL_15763818, EPI_ISL_15763819, EPI_ISL_15763820, EPI_ISL_15763822, EPI_ISL_15763823, EPI_ISL_15763825, EPI_ISL_15763826, EPI_ISL_15763829, EPI_ISL_15763830, EPI_ISL_15763831, EPI_ISL_15763832			
see above	National Virus Reference Laboratory	National Virus Reference Laboratory	Gabriel Gonzalez, Michael Carr, Brian Keogan, Jose Maria Urtasun Elizari, Jonathan Dean, Daniel Hare, Cillian F De Gascun
EPI_ISL_15802695, EPI_ISL_15802697, EPI_ISL_15802700, EPI_ISL_15802704, EPI_ISL_15802710	Direccion de Investigacion en Salud Publica, Instituto Nacional de Salud	Direccion de Investigacion en Salud Publica, Instituto Nacional de Salud	Laiton-Donato,K.D., Franco,C.E., Alvarez-Diaz,D.A., Ruiz-Moreno,H.A., Prada,D.A., Martinez,D. and Mercado-Reyes,M.M.
EPI_ISL_15802722, EPI_ISL_15802723, EPI_ISL_15802724, EPI_ISL_15802725, EPI_ISL_15802726, EPI_ISL_15802727, EPI_ISL_15802728, EPI_ISL_15802729, EPI_ISL_15802730, EPI_ISL_15802731, EPI_ISL_15802732, EPI_ISL_15802733, EPI_ISL_15802734, EPI_ISL_15802735, EPI_ISL_15802736, EPI_ISL_15802737, EPI_ISL_15802738, EPI_ISL_15802739, EPI_ISL_15802740, EPI_ISL_15802741, EPI_ISL_15802742, EPI_ISL_15802743			
see above	Centre for Biological Threats, Highly Pathogenic Viruses, Robert Koch Institute	Centre for Biological Threats, Highly Pathogenic Viruses, Robert Koch Institute	Brinkmann,A., Kohl,C., Pape,K., Schrick,L., Michel,J., Schaade,L. and Nitsche,A.
EPI_ISL_15819629	California Department of Public Health	California Department of Public Health	Viral and Rickettsial Disease Laboratory
EPI_ISL_15831211	División Diagnóstico Molecular Hospital México	División Diagnóstico Molecular Hospital México	Juan Carlos Villalobos Ugalde, Vanessa Villalobos Alfaro, Carlos Ramirez Chavarría
EPI_ISL_15831212	División Diagnóstico Molecular Hospital México	División Diagnóstico Molecular Hospital México	Juan Carlos Villalobos Ugalde, Vanessa Villalobos Alfaro, Sofía Villalobos Abarca
EPI_ISL_15896303, EPI_ISL_15896342, EPI_ISL_15896350, EPI_ISL_15896351	Los Angeles County Public Health Laboratories	Los Angeles County Public Health Laboratories	P. Hemarajata et al.
EPI_ISL_15942296	Sexually Transmitted Diseases (STDs) outpatient service of Dermatology Unit, Fondazione IRCCS Ca' Granda Ospedale Maggiore Policlinico of Milan	Bioinformatic lab, Scientific Institute IRCCS E. Medea	Diego Forni, Rachele Cagliani, Manuela Sironi, Chiara Moltrasio, Luigia Venegoni, Eleonora Quattri, Angelo Marzano
EPI_ISL_15942637, EPI_ISL_15942638, EPI_ISL_15942886	Sexually Transmitted Diseases (STDs) outpatient service of Dermatology Unit, Fondazione IRCCS Ca' Granda Ospedale Maggiore Policlinico of Milan	Bioinformatic Lab, Scientific Institute IRCCS E. Medea	Diego Forni, Rachele Cagliani, Manuela Sironi, Chiara Moltrasio, Luigia Venegoni, Eleonora Quattri, Angelo Marzano
EPI_ISL_15955333, EPI_ISL_15955334	Kaiser Permanente Chino Hills Regional Reference Laboratories	Los Angeles County Public Health Laboratories	P. Hemarajata et al.
EPI_ISL_15955339	Los Angeles County Public Health Laboratories	Los Angeles County Public Health Laboratories	P. Hemarajata et al.
EPI_ISL_15955345, EPI_ISL_15955349, EPI_ISL_15955352	Kaiser Permanente Chino Hills Regional Reference Laboratories	Los Angeles County Public Health Laboratories	P. Hemarajata et al.
EPI_ISL_15972406, EPI_ISL_15972407, EPI_ISL_15972409	Laboratorio Central, Ministerio de Salud Córdoba	Laboratorio Central, Ministerio de Salud Córdoba	Castro, G.; Sicilia, P.; Poklepovich, T.; Campos, J.; Barbas, G.
EPI_ISL_15992095	LESP State of Mexico	Instituto de Diagnostico y Referencia Epidemiologicos (INDRE)	Abril Rodríguez-Maldonado; Claudia Wong-Arámbula; Felipe Arguijo-Perez; Helios Cárdenas-Hernández; Carmen Castro-Méndez; Lidia García-Torres; Ruth Madera-Sandoval; América Mandujano-Martínez; Nancy Martínez-Velázquez; Mireya Mederos-Michel; Angélica Pedraza-Meléndez; Joaquín Quiroz-Mercado; Daniel Regalado-Santiago; Silvia Rivero-Arredondo; Erika Sierra-Atanacio; Fernando González-Domínguez; Lucia Hernández-Rivas, Irma López-Martínez; Ernesto Ramírez-González; Maribel González-Villa
EPI_ISL_15992096	LESP Jalisco	Instituto de Diagnostico y Referencia Epidemiologicos (INDRE)	Abril Rodríguez-Maldonado; Claudia Wong-Arámbula; Felipe Arguijo-Perez; Helios Cárdenas-Hernández; Carmen Castro-Méndez; Lidia García-Torres; Ruth Madera-Sandoval; América Mandujano-Martínez; Nancy Martínez-Velázquez; Mireya Mederos-Michel; Angélica Pedraza-Meléndez; Joaquín Quiroz-Mercado; Daniel Regalado-Santiago; Silvia Rivero-Arredondo; Erika Sierra-Atanacio; Fernando González-Domínguez; Lucia Hernández-Rivas, Irma López-Martínez; Ernesto Ramírez-González; Maribel González-Villa
EPI_ISL_15992097	LESP Morelos	Instituto de Diagnostico y Referencia Epidemiologicos (INDRE)	Abril Rodríguez-Maldonado; Claudia Wong-Arámbula; Felipe Arguijo-Perez; Helios Cárdenas-Hernández; Carmen Castro-Méndez; Lidia García-Torres; Ruth Madera-Sandoval; América Mandujano-Martínez; Nancy Martínez-Velázquez; Mireya Mederos-Michel; Angélica Pedraza-Meléndez; Joaquín Quiroz-Mercado; Daniel Regalado-Santiago; Silvia Rivero-Arredondo; Erika Sierra-Atanacio; Fernando González-Domínguez; Lucia Hernández-Rivas, Irma López-Martínez; Ernesto Ramírez-González; Maribel González-Villa
EPI_ISL_15992098	LESP Nuevo Leon	Instituto de Diagnostico y Referencia Epidemiologicos (INDRE)	Abril Rodríguez-Maldonado; Claudia Wong-Arámbula; Felipe Arguijo-Perez; Helios Cárdenas-Hernández; Carmen Castro-Méndez; Lidia García-Torres; Ruth Madera-Sandoval; América Mandujano-Martínez; Nancy Martínez-Velázquez; Mireya Mederos-Michel; Angélica Pedraza-Meléndez; Joaquín Quiroz-Mercado; Daniel Regalado-Santiago; Silvia Rivero-Arredondo; Erika Sierra-Atanacio; Fernando González-Domínguez; Lucia Hernández-Rivas, Irma López-Martínez; Ernesto Ramírez-González; Maribel González-Villa
EPI_ISL_15992099	LESP Hidalgo	Instituto de Diagnostico y Referencia Epidemiologicos (INDRE)	Abril Rodríguez-Maldonado; Claudia Wong-Arámbula; Felipe Arguijo-Perez; Helios Cárdenas-Hernández; Carmen Castro-Méndez; Lidia García-Torres; Ruth Madera-Sandoval; América Mandujano-Martínez; Nancy Martínez-Velázquez; Mireya Mederos-Michel; Angélica Pedraza-Meléndez; Joaquín Quiroz-Mercado; Daniel Regalado-Santiago; Silvia Rivero-Arredondo; Erika Sierra-Atanacio; Fernando González-Domínguez; Lucia Hernández-Rivas, Irma López-Martínez; Ernesto Ramírez-González; Maribel González-Villa
EPI_ISL_15992100	LESP Campeche	Instituto de Diagnostico y Referencia Epidemiologicos (INDRE)	Abril Rodríguez-Maldonado; Claudia Wong-Arámbula; Felipe Arguijo-Perez; Helios Cárdenas-Hernández; Carmen Castro-Méndez; Lidia García-Torres; Ruth Madera-Sandoval; América Mandujano-Martínez; Nancy Martínez-Velázquez; Mireya Mederos-Michel; Angélica Pedraza-Meléndez; Joaquín Quiroz-Mercado; Daniel Regalado-Santiago; Silvia Rivero-Arredondo; Erika Sierra-Atanacio; Fernando González-Domínguez; Lucia Hernández-Rivas, Irma López-Martínez; Ernesto Ramírez-González; Maribel González-Villa
EPI_ISL_15992101	LESP Tlaxcala	Instituto de Diagnostico y Referencia Epidemiologicos (INDRE)	Abril Rodríguez-Maldonado; Claudia Wong-Arámbula; Felipe Arguijo-Perez; Helios Cárdenas-Hernández; Carmen Castro-Méndez; Lidia García-Torres; Ruth Madera-Sandoval; América Mandujano-Martínez; Nancy Martínez-Velázquez; Mireya Mederos-Michel; Angélica Pedraza-Meléndez; Joaquín Quiroz-Mercado; Daniel Regalado-Santiago; Silvia Rivero-Arredondo; Erika Sierra-Atanacio; Fernando González-Domínguez; Lucia Hernández-Rivas, Irma López-Martínez; Ernesto Ramírez-González; Maribel González-Villa
EPI_ISL_15992102	LESP Aguascalientes	Instituto de Diagnostico y Referencia Epidemiologicos (INDRE)	Abril Rodríguez-Maldonado; Claudia Wong-Arámbula; Felipe Arguijo-Perez; Helios Cárdenas-Hernández; Carmen Castro-Méndez; Lidia García-Torres; Ruth Madera-Sandoval; América Mandujano-Martínez; Nancy Martínez-Velázquez; Mireya Mederos-Michel; Angélica Pedraza-Meléndez; Joaquín Quiroz-Mercado; Daniel Regalado-Santiago; Silvia Rivero-Arredondo; Erika Sierra-Atanacio; Fernando González-Domínguez; Lucia Hernández-Rivas, Irma López-Martínez; Ernesto Ramírez-González; Maribel González-Villa
EPI_ISL_15992103, EPI_ISL_15992104	LESP San Luis Potosi	Instituto de Diagnostico y Referencia Epidemiologicos (INDRE)	Abril Rodríguez-Maldonado; Claudia Wong-Arámbula; Felipe Arguijo-Perez; Helios Cárdenas-Hernández; Carmen Castro-Méndez; Lidia García-Torres; Ruth Madera-Sandoval; América Mandujano-Martínez; Nancy Martínez-Velázquez; Mireya Mederos-Michel; Angélica Pedraza-Meléndez; Joaquín Quiroz-Mercado; Daniel Regalado-Santiago; Silvia Rivero-Arredondo; Erika Sierra-Atanacio; Fernando González-Domínguez; Lucia Hernández-Rivas, Irma López-Martínez; Ernesto Ramírez-González; Maribel González-Villa
EPI_ISL_16012468, EPI_ISL_16012469, EPI_ISL_16012470, EPI_ISL_16012471, EPI_ISL_16012472, EPI_ISL_16012475, EPI_ISL_16012476, EPI_ISL_16012477, EPI_ISL_16012479, EPI_ISL_16012480, EPI_ISL_16012481, EPI_ISL_16012482, EPI_ISL_16012483, EPI_ISL_16012484, EPI_ISL_16012486, EPI_ISL_16012488, EPI_ISL_16012489, EPI_ISL_16012490, EPI_ISL_16012491, EPI_ISL_16012492, EPI_ISL_16012493, EPI_ISL_16012494, EPI_ISL_16012495, EPI_ISL_16012496, EPI_ISL_16012497, EPI_ISL_16012499, EPI_ISL_16012500, EPI_ISL_16012501, EPI_ISL_16012503, EPI_ISL_16012504, EPI_ISL_16012505, EPI_ISL_16012506, EPI_ISL_16012507, EPI_ISL_16012508, EPI_ISL_16012509, EPI_ISL_16012510, EPI_ISL_16012511, EPI_ISL_16012512, EPI_ISL_16012514, EPI_ISL_16012515, EPI_ISL_16012516, EPI_ISL_16012517, EPI_ISL_16012518, EPI_ISL_16012520, EPI_ISL_16012521, EPI_ISL_16012522, EPI_ISL_16012523, EPI_ISL_16012524, EPI_ISL_16012526, EPI_ISL_16012527, EPI_ISL_16012529, EPI_ISL_16012531, EPI_ISL_16012532, EPI_ISL_16012533, EPI_ISL_16012535, EPI_ISL_16012536			
see above	National Virus Reference Laboratory	National Virus Reference Laboratory	Gabriel Gonzalez, Michael Carr, Brian Keogan, Jose Maria Urtasun Elizari, Jonathan Dean, Daniel Hare, Cillian F De Gascun
EPI_ISL_16104842, EPI_ISL_16104843, EPI_ISL_16104844, EPI_ISL_16104845, EPI_ISL_16104846, EPI_ISL_16104847, EPI_ISL_16104848, EPI_ISL_16104849, EPI_ISL_16104850, EPI_ISL_16104851, EPI_ISL_16104852, EPI_ISL_16104853, EPI_ISL_16104854, EPI_ISL_16104855, EPI_ISL_16104856, EPI_ISL_16104857, EPI_ISL_16104858, EPI_ISL_16104859, EPI_ISL_16104860, EPI_ISL_16104861, EPI_ISL_16104862, EPI_ISL_16104863, EPI_ISL_16104864, EPI_ISL_16104865, EPI_ISL_16104869, EPI_ISL_16104870, EPI_ISL_16104871, EPI_ISL_16104872, EPI_ISL_16104873, EPI_ISL_16104874, EPI_ISL_16104875, EPI_ISL_16104876, EPI_ISL_16104877, EPI_ISL_16104878, EPI_ISL_16104879, EPI_ISL_16104880, EPI_ISL_16104881, EPI_ISL_16104882, EPI_ISL_16104883, EPI_ISL_16104884, EPI_ISL_16104885, EPI_ISL_16104886, EPI_ISL_16104887, EPI_ISL_16104888, EPI_ISL_16104889, EPI_ISL_16104890, EPI_ISL_16104891, EPI_ISL_16104892, EPI_ISL_16104893, EPI_ISL_16104894, EPI_ISL_16104895, EPI_ISL_16104896, EPI_ISL_16104897, EPI_ISL_16104899, EPI_ISL_16104900, EPI_ISL_16104901, EPI_ISL_16104902, EPI_ISL_16104903, EPI_ISL_16104904, EPI_ISL_16104905, EPI_ISL_16104906, EPI_ISL_16104907, EPI_ISL_16104908, EPI_ISL_16104909, EPI_ISL_16104910, EPI_ISL_16104912, EPI_ISL_16104913, EPI_ISL_16104914, EPI_ISL_16104916, EPI_ISL_16104917, EPI_ISL_16104918, EPI_ISL_16104919, EPI_ISL_16104920, EPI_ISL_16104921, EPI_ISL_16104922, EPI_ISL_16116727, EPI_ISL_16116728, EPI_ISL_16116729, EPI_ISL_16116730, EPI_ISL_16116733, EPI_ISL_16116734, EPI_ISL_16116735, EPI_ISL_16116736, EPI_ISL_16116737, EPI_ISL_16116738, EPI_ISL_16116739, EPI_ISL_16116740, EPI_ISL_16116741, EPI_ISL_16116742, EPI_ISL_16116743, EPI_ISL_16116744, EPI_ISL_16116745, EPI_ISL_16116746, EPI_ISL_16116747, EPI_ISL_16116749, EPI_ISL_16116750, EPI_ISL_16116752, EPI_ISL_16116754, EPI_ISL_16116755, EPI_ISL_16116756, EPI_ISL_16116757, EPI_ISL_16116758, EPI_ISL_16116759, EPI_ISL_16116760, EPI_ISL_16116761, EPI_ISL_16116762, EPI_ISL_16116763, EPI_ISL_16116765			
see above	Laboratorio de Referencia Nacional de Viruas Inmunoprevenibles. Centro Nacional de Salud Publica. Instiuto Nacional de Salud	Laboratorio de Referencia Nacional de Viruas Inmunoprevenibles. Centro Nacional de Salud Publica. Instiuto Nacional de Salud	Carlos Patricio Padilla Rojas, Carmen Verónica Hurtado Vela, Juana Iris Silva Molina, Luis Bárcena Flores, Víctor Jiménez Vásquez, Alicia Elizabeth Núñez Llanos, Wendy Lizarraga Olivares, Luren Nieves Sevilla Catañeda, Kelly Vanessa Izarra Rojas, Karla Vasquez Cajachahua, Steve Vladimir Acedo Lazo, Omar Alberto Cáceres Rey, Henri Bailón Calderón, Priscila Nayu Lope Pari, Nancy Rojas Serrano, Gloria Arotinco Garayar. Equipo de vigilancia genomica del Instituto Nacional de Salud.
EPI_ISL_16138921	California Department of Public Health	California Department of Public Health	Kath, C., Haw, M., Espinosa, A., and Hacker, J.
EPI_ISL_16190089, EPI_ISL_16190090, EPI_ISL_16190092, EPI_ISL_16190094, EPI_ISL_16190099	Rush University Medical Center	RIPHL at Rush University Medical Center	Stefan Green, Kevin Kunstman, Hannah Barbian, Felix Araujo Perez, Edith Perez, Sofiya Bobrovska, Alyse Kittner, Cecilia Chau, Giancarlo Balangue, Lok Yiu Ashley Wu, Mary Hayden, Joyce Houlihan, Diane Springer, Nicholas Moore
EPI_ISL_16233781	Complejo Hospitalario Universitario de Pontevedra	Microbiology Department. Complexo Hospitalario Universitario de Vigo	Daviña C, Pizcueta J, Trigo M, Perez-Castro S
EPI_ISL_16233783, EPI_ISL_16233784, EPI_ISL_16233785, EPI_ISL_16233786, EPI_ISL_16233787, EPI_ISL_16233788	Microbiology Department. Complexo Hospitalario Universitario de Vigo	Microbiology Department. Complexo Hospitalario Universitario de Vigo	Daviña C, Pizcueta J, Perez-Castro S
EPI_ISL_16260351	Centre Médical de l'Institut Pasteur	Cellule d'Intervention Biologique d'Urgence, Institut Pasteur	Charlotte Balière, Véronique Hourdel, Aurelia Kwasiborski, Quentin Grassin, Maxence Feher, Damien Hoinard, Jessica Vanhomwegen, Fabien Taieb, Paul-Henri Consigny, Jean-Claude Manuguerra, India Leclercq, Christophe Batéjat, Valérie Caro
EPI_ISL_16260402	Cellule d'Intervention Biologique d'Urgence, Institut Pasteur	Cellule d'Intervention Biologique d'Urgence, Institut Pasteur	Charlotte Balière, Véronique Hourdel, Aurelia Kwasiborski, Quentin Grassin, Maxence Feher, Damien Hoinard, Jessica Vanhomwegen, Fabien Taieb, Paul-Henri Consigny, Jean-Claude Manuguerra, India Leclercq, Christophe Batéjat, Valérie Caro
EPI_ISL_16467111	IRCCS Sacro Cuore Don Calabria Hospital, Department of Infectious, Tropical Diseases & Microbiology	IRCCS Sacro Cuore Don Calabria Hospital, Department of Infectious, Tropical Diseases & Microbiology	Michela Deiana, Denise Lavezzari, Silvia Accordini, Concetta Castilletti, Antonio Mori, Elena Pomari, Chiara Piubelli
EPI_ISL_16510132, EPI_ISL_16510134, EPI_ISL_16510138, EPI_ISL_16510140, EPI_ISL_16510141, EPI_ISL_16510143, EPI_ISL_16510145, EPI_ISL_16510147, EPI_ISL_16510155, EPI_ISL_16510156, EPI_ISL_16510157, EPI_ISL_16510158, EPI_ISL_16510159, EPI_ISL_16510162, EPI_ISL_16510163, EPI_ISL_16510168, EPI_ISL_16510169, EPI_ISL_16510170, EPI_ISL_16510172, EPI_ISL_16510173, EPI_ISL_16510174, EPI_ISL_16510175, EPI_ISL_16510177, EPI_ISL_16510178, EPI_ISL_16510179, EPI_ISL_16510180, EPI_ISL_16510182, EPI_ISL_16510183, EPI_ISL_16510184, EPI_ISL_16510185			
see above	National Virus Reference Laboratory	National Virus Reference Laboratory	Gabriel Gonzalez, Michael Carr, Brian Keogan, Jose Maria Urtasun Elizari, Jonathan Dean, Daniel Hare, Cillian F De Gascun
EPI_ISL_16645221	Antioquia, Laboratorio Departamental de Salud Publica de Antioquia	Antioquia, Laboratorio Departamental de Salud Publica de Antioquia	Betancur,I.I.B., Velarde-Hoyos,C.-A.C.V., Gomez,R.R.G. and Mercado-Reyes,M.M.R.

EPI_ISL_16645229	Environment and Infectious Risks Unit, Insitut Pasteur	Environment and Infectious Risks Unit, Insitut Pasteur	Baliere,C., Hourdel,V., Kwasiborski,A., Grassin,Q., Feher,M., Hoinard,D., Vanhomwegen,J., Taieb,F., Consigny,P.-H., Manuguerra,J.-C., Leclercq,I., Batejat,C. and Caro,V.
EPI_ISL_16679206	Los Angeles County Public Health Laboratories	Los Angeles County Public Health Laboratories	P. Hemarajata et al.
EPI_ISL_16679222, EPI_ISL_16679225, EPI_ISL_16679235	Kaiser Permanente Chino Hills Regional Reference Laboratories	Los Angeles County Public Health Laboratories	P. Hemarajata et al.
EPI_ISL_16679240	Los Angeles County Public Health Laboratories	Los Angeles County Public Health Laboratories	P. Hemarajata et al.
EPI_ISL_16679258	California Department of Public Health	California Department of Public Health	Probert,W., Espinosa,A., Kath,C., Haw,M., O'Neil,R., Bell,J. and Hacker,J.
EPI_ISL_16871160, EPI_ISL_16871161	Laboratorio de Enterovirus, Instituto Oswaldo Cruz, Fiocruz	Instituto Oswaldo Cruz FIOCRUZ - Laboratory of Respiratory Viruses and Measles (LVRS)	Paola Resende, Elisa Cavalcante Pereira, Bruna Mendonça da Silva, Jéssica Graça Macedo de Carvalho, Larissa Macedo Pinto, Victor Guimaraes, Marilda Siqueira, Renan da Silva Faustino, Marilia Santini, Edson Elias da Silva on behalf of the Fiocruz Genomic Surveillance Network
EPI_ISL_16926988, EPI_ISL_16926991, EPI_ISL_16926994, EPI_ISL_16926997, EPI_ISL_16927000, EPI_ISL_16927007, EPI_ISL_16927018, EPI_ISL_16927021	Naval Infectious Diseases Diagnostic Laboratory	Naval Medical Research Center Biological Defense Research Directorate	Logan J. Voegtly, Gregory K. Rice, Adrian Pakey, Andrea E. Luquette, Maren C. Fitzpatrick, Hannah M. Drumm, Victor Sugiharto, Hua-Wei Chen, Francisco Malagon, Regina Z. Cer, Kimberly A. Bishop-Lilly
EPI_ISL_16946400	Division de Microbiologia, Hospital Nacional de Niños Carlos Saenz Herrera	Instituto Costarricense de Investigacion y Enseñanza en Nutricion y Salud, Inciensa	Diana Cantillo, Hillary Serrano, Ana Isela Ruiz, Gustavo Vega, Claudio Soto-Garita, Adriana Godinez, Estela Cordero, Melany Calderon, Francisco Duarte
EPI_ISL_16955243, EPI_ISL_16955272, EPI_ISL_16955278, EPI_ISL_16955279, EPI_ISL_16955289, EPI_ISL_16955294, EPI_ISL_16955299, EPI_ISL_16955300, EPI_ISL_16955305	Public Health Laboratory, NYC Department of Health and Mental Hygiene	Public Health Laboratory, NYC Department of Health and Mental Hygiene	Wang,J.C., Amin,H.S., Clabby,T.T., Taki,F., Su,M., Rahat,A., De La Cruz,N., Olsen,A., Thi,C., Silver,S., Akther,S., Chowdhury,M., Omoregie,E. and Hughes,S.
EPI_ISL_16985951, EPI_ISL_16985952, EPI_ISL_16985956, EPI_ISL_16985957, EPI_ISL_16985958, EPI_ISL_16985959, EPI_ISL_16985960, EPI_ISL_16985961, EPI_ISL_16985962, EPI_ISL_16985964, EPI_ISL_16985965, EPI_ISL_16985966, EPI_ISL_16985967, EPI_ISL_16985969, EPI_ISL_16985970, EPI_ISL_16985971, EPI_ISL_16985972			
see above	National Virus Reference Laboratory	National Virus Reference Laboratory	Gabriel Gonzalez, Michael Carr, Emer O'Byrne, Weronika Banka, Brian Keogan, Jose Maria Urtasun Elizari, Jonathan Dean, Daniel Hare, Cillian F De Gascun
EPI_ISL_16997418, EPI_ISL_16997419, EPI_ISL_16997421, EPI_ISL_16997425, EPI_ISL_16997427, EPI_ISL_16997432, EPI_ISL_16997433, EPI_ISL_16997435, EPI_ISL_16997437, EPI_ISL_16997442, EPI_ISL_16997445			
see above	Kaiser Permanente Chino Hills Regional Reference Laboratories	Los Angeles County Public Health Laboratories	P. Hemarajata et al.
EPI_ISL_17012023, EPI_ISL_17012036	Laboratorio de Virus Exantematicos, Gastroentéricos y Otros Transmitidos por Vectores	Centro de Referencia Nacional de Genomica, Secuenciacion y Bioinformatica GENSBIO, INSPI-CZ9	Andrés Carrazco, Silvia Salgado, Diana Gutiérrez, Damaris Alarcón, Andrés Herrera, Andrés Tinizaray, Martha Sánchez, Johanna Parrales, Diego Morales, Jorge Bejarano, Leandro Patiño.
EPI_ISL_17012084, EPI_ISL_17012090, EPI_ISL_17012091, EPI_ISL_17012092, EPI_ISL_17012094, EPI_ISL_17012097, EPI_ISL_17012102, EPI_ISL_17012109	Laboratorio de Virus Exantematicos, Gastroentéricos y Otros Transmitidos por Vectores	Centro de Referencia Nacional de Genomica, Secuenciacion y Bioinformatica GENSBIO, INSPI-CZ9	Andrés Carrazco-Motalvo, Silvia Salgado, Diana Gutiérrez, Damaris Alarcón, Andrés Herrera, Andrés Tinizaray, Ruth Gómez, Martha Sánchez, Johanna Parrales, Diego Morales, Jorge Bejarano, Leandro Patiño.
EPI_ISL_17019462	Parkland Health and Hospital System	Dallas County Health & Human Services Public Health Laboratory	Kabir, Farruk; Plaisance, Erin; Stringer, Joey; Short, Luke.
EPI_ISL_17048205, EPI_ISL_17048206, EPI_ISL_17048207	Laboratorio Central de Saude Publica do Estado da Bahia (LACEN/BA)	Laboratory of Respiratory Viruses and Measles, Oswaldo Cruz Institute, FIOCRUZ	Paola Resende, Fernando Motta, Elisa Cavalcante Pereira, Bruna Mendonça da Silva, Jéssica Graça Macedo de Carvalho, Larissa Macedo Pinto, Victor Guimaraes, Felicidade Pereira, Marilda Siqueira, Renan da Silva Faustino, Marilia Santini, Edson Elias da Silva on behalf of the Fiocruz COVID-19 Genomic Surveillance Network
EPI_ISL_17085706	Public Health Laboratory, NYC Department of Health and Mental Hygiene	Public Health Laboratory, NYC Department of Health and Mental Hygiene	Wang,J.C., Amin,H.S., Clabby,T.T., Taki,F., Su,M., Rahat,A., De La Cruz,N., Olsen,A., Thi,C., Silver,S., Akther,S., Chowdhury,M., Omoregie,E. and Hughes,S.
EPI_ISL_17085746, EPI_ISL_17085748, EPI_ISL_17085782, EPI_ISL_17085784, EPI_ISL_17085795, EPI_ISL_17085922, EPI_ISL_17085925, EPI_ISL_17085926, EPI_ISL_17085927, EPI_ISL_17085948, EPI_ISL_17085955, EPI_ISL_17085961			
see above	Public Health Laboratory, NYC Department of Health and Mental Hygiene	Public Health Laboratory, NYC Department of Health and Mental Hygiene	Clabby,T.T., Amin,H.S., Wang,J.C., Taki,F., Su,M., Rahat,A., De La Cruz,N., Olsen,A., Thi,C., Silver,S., Akther,S., Chowdhury,M., Omoregie,E. and Hughes,S.
EPI_ISL_17085977, EPI_ISL_17085984, EPI_ISL_17085986, EPI_ISL_17085990, EPI_ISL_17085998, EPI_ISL_17086001, EPI_ISL_17086002, EPI_ISL_17086005, EPI_ISL_17086007, EPI_ISL_17086010, EPI_ISL_17086015, EPI_ISL_17086021			
see above	Public Health Laboratory, NYC Department of Health and Mental Hygiene	Public Health Laboratory, NYC Department of Health and Mental Hygiene	Amin,H.S., Clabby,T.T., Wang,J.C., Taki,F., Su,M., Rahat,A., De La Cruz,N., Olsen,A., Thi,C., Silver,S., Akther,S., Chowdhury,M., Omoregie,E. and Hughes,S.
EPI_ISL_17086066, EPI_ISL_17086078, EPI_ISL_17086111, EPI_ISL_17086112, EPI_ISL_17086114, EPI_ISL_17086115, EPI_ISL_17086116, EPI_ISL_17086117, EPI_ISL_17086119, EPI_ISL_17086120, EPI_ISL_17086121, EPI_ISL_17086124, EPI_ISL_17086125, EPI_ISL_17086131, EPI_ISL_17086138, EPI_ISL_17086139, EPI_ISL_17086142, EPI_ISL_17086144, EPI_ISL_17086145, EPI_ISL_17086146, EPI_ISL_17086147, EPI_ISL_17086148, EPI_ISL_17086151, EPI_ISL_17086155, EPI_ISL_17086263			
see above	Public Health Laboratory, NYC Department of Health and Mental Hygiene	Public Health Laboratory, NYC Department of Health and Mental Hygiene	Wang,J.C., Amin,H.S., Clabby,T.T., Taki,F., Su,M., Rahat,A., De La Cruz,N., Olsen,A., Thi,C., Silver,S., Akther,S., Chowdhury,M., Omoregie,E. and Hughes,S.
EPI_ISL_17104205, EPI_ISL_17104541, EPI_ISL_17104542, EPI_ISL_17104547, EPI_ISL_17104551, EPI_ISL_17104560, EPI_ISL_17104561, EPI_ISL_17104563, EPI_ISL_17104564, EPI_ISL_17104565, EPI_ISL_17104569, EPI_ISL_17104570, EPI_ISL_17104574, EPI_ISL_17104577, EPI_ISL_17104581, EPI_ISL_17104583, EPI_ISL_17104585, EPI_ISL_17104586, EPI_ISL_17104588, EPI_ISL_17104589, EPI_ISL_17104590, EPI_ISL_17104591, EPI_ISL_17104592, EPI_ISL_17104593, EPI_ISL_17104595, EPI_ISL_17104597, EPI_ISL_17104642, EPI_ISL_17104649			
see above	Public Health Laboratory, NYC Department of Health and Mental Hygiene	Public Health Laboratory, NYC Department of Health and Mental Hygiene	Clabby,T.T., Amin,H.S., Wang,J.C., Taki,F., Su,M., Rahat,A., De La Cruz,N., Olsen,A., Thi,C., Silver,S., Akther,S., Chowdhury,M., Omoregie,E. and Hughes,S.
EPI_ISL_17118738	Environmental, Agricultural, and Occupational Health, University of Nebraska Medical Center	Environmental, Agricultural, and Occupational Health, University of Nebraska Medical Center	Pentella,M., Chapman,R.C., Stapleton,J., Meier,J., Xiang,J., Li,M., Reeb,V., Benfer,J., Eveland,K., Wiley,M.R., Hottel,W. and Cross,S.T.
EPI_ISL_17165732, EPI_ISL_17165740	Public Health Laboratory, NYC Department of Health and Mental Hygiene (DOHMH)	Public Health Laboratory, NYC Department of Health and Mental Hygiene (DOHMH)	Clabby,T.T., Amin,H.S., Wang,J.C., Taki,F., Su,M., Rahat,A., De La Cruz,N., Olsen,A., Thi,C., Silver,S., Akther,S., Chowdhury,M., Omoregie,E. and Hughes,S.
EPI_ISL_17170656, EPI_ISL_17170658, EPI_ISL_17170659, EPI_ISL_17170661, EPI_ISL_17170663, EPI_ISL_17170664, EPI_ISL_17170665, EPI_ISL_17170666, EPI_ISL_17170667, EPI_ISL_17170668, EPI_ISL_17170669, EPI_ISL_17170670			
see above	California Department of Public Health	California Department of Public Health	Haw, M. , Kath, C., Espinosa, A., O'Neil, R., and Hacker, J.
EPI_ISL_17179628	St Jame's Hospital, Virology Department	National Virus Reference Laboratory	Patrice Keane, Yvonne Lynagh, Brendan Crowley, Gabriel Gonzalez, Michael Carr, Emer O'Byrne, Weronika Banka, Brian Keogan, Jose Maria Urtasun Elizari, Jonathan Dean, Daniel Hare, Cillian F De Gascun
EPI_ISL_17179629, EPI_ISL_17179630, EPI_ISL_17179631, EPI_ISL_17179632	National Virus Reference Laboratory	National Virus Reference Laboratory	Gabriel Gonzalez, Michael Carr, Emer O'Byrne, Weronika Banka, Brian Keogan, Jose Maria Urtasun Elizari, Jonathan Dean, Daniel Hare, Cillian F De Gascun
EPI_ISL_17179633	St Jame's Hospital, Virology Department	National Virus Reference Laboratory	Patrice Keane, Yvonne Lynagh, Brendan Crowley, Gabriel Gonzalez, Michael Carr, Emer O'Byrne, Weronika Banka, Brian Keogan, Jose Maria Urtasun Elizari, Jonathan Dean, Daniel Hare, Cillian F De Gascun
EPI_ISL_17179634, EPI_ISL_17179635, EPI_ISL_17179636, EPI_ISL_17179637	National Virus Reference Laboratory	National Virus Reference Laboratory	Gabriel Gonzalez, Michael Carr, Emer O'Byrne, Weronika Banka, Brian Keogan, Jose Maria Urtasun Elizari, Jonathan Dean, Daniel Hare, Cillian F De Gascun
EPI_ISL_17179638, EPI_ISL_17179639, EPI_ISL_17179640, EPI_ISL_17179641, EPI_ISL_17179642, EPI_ISL_17179643	St Jame's Hospital, Virology Department	National Virus Reference Laboratory	Patrice Keane, Yvonne Lynagh, Brendan Crowley, Gabriel Gonzalez, Michael Carr, Emer O'Byrne, Weronika Banka, Brian Keogan, Jose Maria Urtasun Elizari, Jonathan Dean, Daniel Hare, Cillian F De Gascun
EPI_ISL_17187497, EPI_ISL_17187498	Vajira Hospital	Thai Red Cross Emerging Infectious Diseases Clinical Center and Faculty of Medicine, Chulalongkorn University	Suppasit srisaeng, Praepoly Ruekmuang, Kusuma Swangpun, Arriya Panchaiyaphum, Pakita Salaeh, Natpusda Kongmaung, Pornsiri Limwattanawong, Noree Pholprasert, Montriya Unteamsom, Kanjana Jeknok, Withak Withaksabut, Sunisa Nilda, Artorn Niakul, Sopon Iamsirithaworn, Thitipong Yingyong, Rossaporn Kittiyaowamarn, Rome Buathong, Ratanaporn Tangwangvivat, Supaporn Wacharapluesadee, Sininat Petcharat, Ananporn Supataragul, Stefan Fernandez, Achawin Rojanaviwat, Chonticha Klungthong, Pilailuk Okada, Khajohn Joonlasak, Chakkarat Pitayawonganon, Opass Putcharoen
EPI_ISL_17187499	Department of Disease Control, Ministry of Public Health	Thai Red Cross Emerging Infectious Diseases Clinical Center and Faculty of Medicine, Chulalongkorn University	Suppasit srisaeng, Praepoly Ruekmuang, Kusuma Swangpun, Arriya Panchaiyaphum, Pakita Salaeh, Natpusda Kongmaung, Pornsiri Limwattanawong, Noree Pholprasert, Montriya Unteamsom, Kanjana Jeknok, Withak Withaksabut, Sunisa Nilda, Artorn Niakul, Sopon Iamsirithaworn, Thitipong Yingyong, Rossaporn Kittiyaowamarn, Rome Buathong, Ratanaporn Tangwangvivat, Supaporn Wacharapluesadee, Sininat Petcharat, Ananporn Supataragul, Stefan Fernandez, Achawin Rojanaviwat, Chonticha Klungthong, Pilailuk Okada, Khajohn Joonlasak, Chakkarat Pitayawonganon, Opass Putcharoen
EPI_ISL_17201439, EPI_ISL_17201440, EPI_ISL_17201441	Genomics Division, Instituto Tecnologico y de Energias Renovables (ITER)	Genomics Division, Instituto Tecnologico y de Energias Renovables (ITER)	Munoz-Barrera,A., Ciuffreda,L., Alcoba-Florez,J., Rubio-Rodriguez,L.A., Rodriguez-Perez,H., Gil-Campesino,H., Garcia-Martinez de Artola,D., Salas-Hernandez,J., Rodriguez-Nunez,J., Inigo-Campos,A., Garcia-Olivares,V., Diez-Gil,O., Gonzalez-Montelongo,R., Valenzuela-Fernandez,A., Lorenzo-Salazar,J.M. and Flores,C.
EPI_ISL_17206607, EPI_ISL_17206612, EPI_ISL_17206614, EPI_ISL_17206619	California Department of Public Health	California Department of Public Health	Haw,M., Kath,C., Espinosa,A., O'Neil,R., and Hacker,J.
EPI_ISL_17206622	California Department of Public Health	California Department of Public Health	Kath, C., Haw, M., Espinosa, A., and Hacker, J.
EPI_ISL_17211324, EPI_ISL_17211327, EPI_ISL_17211328, EPI_ISL_17211329	Kaiser Permanente Chino Hills Regional Reference Laboratories	Los Angeles County Public Health Laboratories	P. Hemarajata et al.
EPI_ISL_17211331	Los Angeles County Public Health Laboratories	Los Angeles County Public Health Laboratories	P. Hemarajata et al.
EPI_ISL_17222811, EPI_ISL_17222813, EPI_ISL_17222814, EPI_ISL_17222817, EPI_ISL_17222819, EPI_ISL_17222820, EPI_ISL_17222822, EPI_ISL_17222823, EPI_ISL_17222824, EPI_ISL_17222825, EPI_ISL_17222827, EPI_ISL_17222828			
see above	Viral and Rickettsial Disease Laboratory (VRDL) California Department of Public Health (CDPH)	Viral and Rickettsial Disease Laboratory (VRDL) California Department of Public Health (CDPH)	Haw,M., Kath,C., Espinosa,A., O'Neil,R. and Hacker,J.
EPI_ISL_17269834, EPI_ISL_17269835, EPI_ISL_17269836, EPI_ISL_17269837, EPI_ISL_17269839	Environmental, Agricultural, and Occupational Health, University of Nebraska Medical Center	Environmental, Agricultural, and Occupational Health, University of Nebraska Medical Center	Tegomoh,B., Cross,S.T., Chapman,R.C., Bernhard,K., McCutchen,E.L., Fauver,J.R., Pratt,C.B., Warden,D.E., Iwen,P.C., Donahue,M. and Wiley,M.R.
EPI_ISL_17406101, EPI_ISL_17406102, EPI_ISL_17406103, EPI_ISL_17406110, EPI_ISL_17406121	CDCT/CEVS/SES-RS	CDCT/CEVS/SES-RS	Richard Steiner Salvato, Fernanda Marques Godinho, Regina Bones Barcellos, Patricia Sesterheim, Amanda Pellenz Ruivo, Viviane Horn de Melo, Júlio Augusto Schroder
EPI_ISL_17424657, EPI_ISL_17424658, EPI_ISL_17424659, EPI_ISL_17424660, EPI_ISL_17424662, EPI_ISL_17424663, EPI_ISL_17424664, EPI_ISL_17424665, EPI_ISL_17424666, EPI_ISL_17424667, EPI_ISL_17424668, EPI_ISL_17424669, EPI_ISL_17424673, EPI_ISL_17424674, EPI_ISL_17424677			

see above	Molecular Microbiology Laboratory, Department of Pathology, Molecular and Cell-Based Medicine, Icahn School of Medicine at Mount Sinai,	Molecular Microbiology Laboratory, Department of Pathology, Molecular and Cell-Based Medicine, Icahn School of Medicine at Mount Sinai,	Luz H. Patiño, Susana Guerra, Marina Muñoz, Nicolas Luna , Keith Farrugia, Adriana van de Guchte, Zain Khalil , Ana Silvia Gonzalez-Reiche, Matthew M. Hernandez ,Radhika Banu, Paras Shrestha, Bernadette Liggayu, Adolfo Firpo Betancourt, David Reich, Carlos Cordon-Cardo, Randy Albrechtf, Rebecca Pearlf, Viviana Simona, Aria Rookera, Emilia Mia Sordillo, Harm van Bakeld, Adolfo García-Sastre, Dusan Bogunovic, Gustavo Palacios, Alberto Paniz Mondolfi, Juan David Ramírez
EPI_ISL_17445514, EPI_ISL_17445515, EPI_ISL_17445516, EPI_ISL_17445517, EPI_ISL_17445518, EPI_ISL_17445519	Tokyo Metropolitan Institute of Public Health	Tokyo Metropolitan Institute of Public Health	Fumi Kasuya, Wakaba Okada, Ryota Kumagai, Sachiko Harada, Arisa Amano, Michiya Hasegawa, Mami Nagashima, Kenji Sadamasu
EPI_ISL_17471100, EPI_ISL_17471101, EPI_ISL_17471102, EPI_ISL_17471103	Laboratorio de Enterovirus, Instituto Oswaldo Cruz, Fiocruz	Laboratory of Respiratory Viruses and Measles, Oswaldo Cruz Institute, FIOCRUZ	Paola Resende, Elisa Cavalcante Pereira, Bruna Mendonça da Silva, Jéssica Graça Macedo de Carvalho, Larissa Macedo Pinto, Victor Guimaraes, Marilda Siqueira, Renan da Silva Faustino, Marilia Santini, Beatriz Grinsztejn, Mayara Secco Torres da Silva, Edson Elias da Silva on behalf of the Fiocruz Genomic Surveillance Network
EPI_ISL_17502583	Public Health Laboratory, Public Health Service Amsterdam, The Netherlands	Department of Medical Microbiology & Infection prevention, Amsterdam University Medical Centers location AMC	Matthijs Welkers, Jelle Koopsen, Robin van Houdt, Marcel Jonges, Sebastien Matamoros, Sjoerd Rebers, Fokla Zorgdrager, Sylvia Bruisten, Akke Cornelissen, Janke Schinkel, Ewout Fanoy, Roisin Bavalia, Menno de Jong and Mariken van der Lubben on behalf of the Amsterdam Regional Genomic epidemiology and Outbreak Surveillance (ARGOS) consortium
EPI_ISL_17525484	Division de Microbiologia, Hospital Nacional de Niños Carlos Saenz Herrera	Inciensa, Investigación y Enseñanza en Nutrición y Salud Centro Nacional de Referencia de Virología	Cristian Perez Corrales, Christopher Mairena Acuña, Diana Cantillo, Hillary Serrano, Ana Isela Ruiz, Gustavo Vega, Claudio Soto-Garita, Adriana Godinez, Estela Cordero, Melany Calderon, Francisco Duarte
EPI_ISL_17536782, EPI_ISL_17536784, EPI_ISL_17536785, EPI_ISL_17614021, EPI_ISL_17614022, EPI_ISL_17614024, EPI_ISL_17614025, EPI_ISL_17614026, EPI_ISL_17614030, EPI_ISL_17614034, EPI_ISL_17614036, EPI_ISL_17614037, EPI_ISL_17614040, EPI_ISL_17614046, EPI_ISL_17614048, EPI_ISL_17614049			
see above	Laboratorio de Enterovirus, Instituto Oswaldo Cruz, Fiocruz	Instituto Oswaldo Cruz FIOCRUZ - Laboratory of Respiratory Viruses and Measles (LVRS)	Paola Resende, Elisa Cavalcante Pereira, Bruna Mendonça da Silva, Jéssica Graça Macedo de Carvalho, Larissa Macedo Pinto, Victor Guimaraes, Marilda Siqueira, Renan da Silva Faustino, Marilia Santini, Edson Elias da Silva on behalf of the Fiocruz Genomic Surveillance Network
EPI_ISL_17665624, EPI_ISL_17665625, EPI_ISL_17665626, EPI_ISL_17665627	Tokyo Metropolitan Institute of Public Health	Tokyo Metropolitan Institute of Public Health	Fumi Kasuya, Wakaba Okada, Ryota Kumagai, Sachiko Harada, Arisa Amano, Michiya Hasegawa, Mami Nagashima, Kenji Sadamasu
EPI_ISL_17672206	LESP State of Mexico	Instituto de Diagnostico y Referencia Epidemiologicos (INDRE)	Abril Rodríguez-Maldonado; Claudia Wong-Arámbula; Silvia Rivero-Arredondo; Ruth Madera-Sandoval; Joaquín Quiroz-Mercado; Fernando González-Domínguez; Lucia Hernández-Rivas, Irma López-Martínez; Ernesto Ramírez-González; Maribel González-Villa
EPI_ISL_17672208	LESP Queretaro	Instituto de Diagnostico y Referencia Epidemiologicos (INDRE)	Abril Rodríguez-Maldonado; Claudia Wong-Arámbula; Silvia Rivero-Arredondo; Ruth Madera-Sandoval; Joaquín Quiroz-Mercado; Fernando González-Domínguez; Lucia Hernández-Rivas, Irma López-Martínez; Ernesto Ramírez-González; Maribel González-Villa
EPI_ISL_17672209	LESP Yucatan	Instituto de Diagnostico y Referencia Epidemiologicos (INDRE)	Abril Rodríguez-Maldonado; Claudia Wong-Arámbula; Silvia Rivero-Arredondo; Ruth Madera-Sandoval; Joaquín Quiroz-Mercado; Fernando González-Domínguez; Lucia Hernández-Rivas, Irma López-Martínez; Ernesto Ramírez-González; Maribel González-Villa
EPI_ISL_17672210	LESP Quintana Roo	Instituto de Diagnostico y Referencia Epidemiologicos (INDRE)	Abril Rodríguez-Maldonado; Claudia Wong-Arámbula; Silvia Rivero-Arredondo; Ruth Madera-Sandoval; Joaquín Quiroz-Mercado; Fernando González-Domínguez; Lucia Hernández-Rivas, Irma López-Martínez; Ernesto Ramírez-González; Maribel González-Villa
EPI_ISL_17672211	LESP Mexico City	Instituto de Diagnostico y Referencia Epidemiologicos (INDRE)	Abril Rodríguez-Maldonado; Claudia Wong-Arámbula; Silvia Rivero-Arredondo; Ruth Madera-Sandoval; Joaquín Quiroz-Mercado; Fernando González-Domínguez; Lucia Hernández-Rivas, Irma López-Martínez; Ernesto Ramírez-González; Maribel González-Villa
EPI_ISL_17672212	LESP Tamaulipas	Instituto de Diagnostico y Referencia Epidemiologicos (INDRE)	Abril Rodríguez-Maldonado; Claudia Wong-Arámbula; Silvia Rivero-Arredondo; Ruth Madera-Sandoval; Joaquín Quiroz-Mercado; Fernando González-Domínguez; Lucia Hernández-Rivas, Irma López-Martínez; Ernesto Ramírez-González; Maribel González-Villa
EPI_ISL_17672213	LESP Puebla	Instituto de Diagnostico y Referencia Epidemiologicos (INDRE)	Abril Rodríguez-Maldonado; Claudia Wong-Arámbula; Silvia Rivero-Arredondo; Ruth Madera-Sandoval; Joaquín Quiroz-Mercado; Fernando González-Domínguez; Lucia Hernández-Rivas, Irma López-Martínez; Ernesto Ramírez-González; Maribel González-Villa
EPI_ISL_17672214	LESP Guerrero	Instituto de Diagnostico y Referencia Epidemiologicos (INDRE)	Abril Rodríguez-Maldonado; Claudia Wong-Arámbula; Silvia Rivero-Arredondo; Ruth Madera-Sandoval; Joaquín Quiroz-Mercado; Fernando González-Domínguez; Lucia Hernández-Rivas, Irma López-Martínez; Ernesto Ramírez-González; Maribel González-Villa
EPI_ISL_17672216	LESP Hidalgo	Instituto de Diagnostico y Referencia Epidemiologicos (INDRE)	Abril Rodríguez-Maldonado; Claudia Wong-Arámbula; Silvia Rivero-Arredondo; Ruth Madera-Sandoval; Joaquín Quiroz-Mercado; Fernando González-Domínguez; Lucia Hernández-Rivas, Irma López-Martínez; Ernesto Ramírez-González; Maribel González-Villa
EPI_ISL_17672217	LESP Zacatecas	Instituto de Diagnostico y Referencia Epidemiologicos (INDRE)	Abril Rodríguez-Maldonado; Claudia Wong-Arámbula; Silvia Rivero-Arredondo; Ruth Madera-Sandoval; Joaquín Quiroz-Mercado; Fernando González-Domínguez; Lucia Hernández-Rivas, Irma López-Martínez; Ernesto Ramírez-González; Maribel González-Villa
EPI_ISL_17672218	LESP Tabasco	Instituto de Diagnostico y Referencia Epidemiologicos (INDRE)	Abril Rodríguez-Maldonado; Claudia Wong-Arámbula; Silvia Rivero-Arredondo; Ruth Madera-Sandoval; Joaquín Quiroz-Mercado; Fernando González-Domínguez; Lucia Hernández-Rivas, Irma López-Martínez; Ernesto Ramírez-González; Maribel González-Villa
EPI_ISL_17672220	LESP Tlaxcala	Instituto de Diagnostico y Referencia Epidemiologicos (INDRE)	Abril Rodríguez-Maldonado; Claudia Wong-Arámbula; Silvia Rivero-Arredondo; Ruth Madera-Sandoval; Joaquín Quiroz-Mercado; Fernando González-Domínguez; Lucia Hernández-Rivas, Irma López-Martínez; Ernesto Ramírez-González; Maribel González-Villa
EPI_ISL_17672221	LESP Chihuahua	Instituto de Diagnostico y Referencia Epidemiologicos (INDRE)	Abril Rodríguez-Maldonado; Claudia Wong-Arámbula; Silvia Rivero-Arredondo; Ruth Madera-Sandoval; Joaquín Quiroz-Mercado; Fernando González-Domínguez; Lucia Hernández-Rivas, Irma López-Martínez; Ernesto Ramírez-González; Maribel González-Villa
EPI_ISL_17672222	LESP Veracruz	Instituto de Diagnostico y Referencia Epidemiologicos (INDRE)	Abril Rodríguez-Maldonado; Claudia Wong-Arámbula; Silvia Rivero-Arredondo; Ruth Madera-Sandoval; Joaquín Quiroz-Mercado; Fernando González-Domínguez; Lucia Hernández-Rivas, Irma López-Martínez; Ernesto Ramírez-González; Maribel González-Villa
EPI_ISL_17672223	LESP Baja California	Instituto de Diagnostico y Referencia Epidemiologicos (INDRE)	Abril Rodríguez-Maldonado; Claudia Wong-Arámbula; Silvia Rivero-Arredondo; Ruth Madera-Sandoval; Joaquín Quiroz-Mercado; Fernando González-Domínguez; Lucia Hernández-Rivas, Irma López-Martínez; Ernesto Ramírez-González; Maribel González-Villa
EPI_ISL_17672224	LESP Tamaulipas	Instituto de Diagnostico y Referencia Epidemiologicos (INDRE)	Abril Rodríguez-Maldonado; Claudia Wong-Arámbula; Silvia Rivero-Arredondo; Ruth Madera-Sandoval; Joaquín Quiroz-Mercado; Fernando González-Domínguez; Lucia Hernández-Rivas, Irma López-Martínez; Ernesto Ramírez-González; Maribel González-Villa
EPI_ISL_17672225	LESP Campeche	Instituto de Diagnostico y Referencia Epidemiologicos (INDRE)	Abril Rodríguez-Maldonado; Claudia Wong-Arámbula; Silvia Rivero-Arredondo; Ruth Madera-Sandoval; Joaquín Quiroz-Mercado; Fernando González-Domínguez; Lucia Hernández-Rivas, Irma López-Martínez; Ernesto Ramírez-González; Maribel González-Villa
EPI_ISL_17672226	LESP Hidalgo	Instituto de Diagnostico y Referencia Epidemiologicos (INDRE)	Abril Rodríguez-Maldonado; Claudia Wong-Arámbula; Silvia Rivero-Arredondo; Ruth Madera-Sandoval; Joaquín Quiroz-Mercado; Fernando González-Domínguez; Lucia Hernández-Rivas, Irma López-Martínez; Ernesto Ramírez-González; Maribel González-Villa
EPI_ISL_17672227	LESP Mexico City	Instituto de Diagnostico y Referencia Epidemiologicos (INDRE)	Abril Rodríguez-Maldonado; Claudia Wong-Arámbula; Silvia Rivero-Arredondo; Ruth Madera-Sandoval; Joaquín Quiroz-Mercado; Fernando González-Domínguez; Lucia Hernández-Rivas, Irma López-Martínez; Ernesto Ramírez-González; Maribel González-Villa
EPI_ISL_17672228	LESP Guerrero	Instituto de Diagnostico y Referencia Epidemiologicos (INDRE)	Abril Rodríguez-Maldonado; Claudia Wong-Arámbula; Silvia Rivero-Arredondo; Ruth Madera-Sandoval; Joaquín Quiroz-Mercado; Fernando González-Domínguez; Lucia Hernández-Rivas, Irma López-Martínez; Ernesto Ramírez-González; Maribel González-Villa
EPI_ISL_17672229	LESP Coahuila	Instituto de Diagnostico y Referencia Epidemiologicos (INDRE)	Abril Rodríguez-Maldonado; Claudia Wong-Arámbula; Silvia Rivero-Arredondo; Ruth Madera-Sandoval; Joaquín Quiroz-Mercado; Fernando González-Domínguez; Lucia Hernández-Rivas, Irma López-Martínez; Ernesto Ramírez-González; Maribel González-Villa
EPI_ISL_17672230	LESP Tabasco	Instituto de Diagnostico y Referencia Epidemiologicos (INDRE)	Abril Rodríguez-Maldonado; Claudia Wong-Arámbula; Silvia Rivero-Arredondo; Ruth Madera-Sandoval; Joaquín Quiroz-Mercado; Fernando González-Domínguez; Lucia Hernández-Rivas, Irma López-Martínez; Ernesto Ramírez-González; Maribel González-Villa
EPI_ISL_17672231	LESP Chiapas	Instituto de Diagnostico y Referencia Epidemiologicos (INDRE)	Abril Rodríguez-Maldonado; Claudia Wong-Arámbula; Silvia Rivero-Arredondo; Ruth Madera-Sandoval; Joaquín Quiroz-Mercado; Fernando González-Domínguez; Lucia Hernández-Rivas, Irma López-Martínez; Ernesto Ramírez-González; Maribel González-Villa
EPI_ISL_17672232	LESP Yucatan	Instituto de Diagnostico y Referencia Epidemiologicos (INDRE)	Abril Rodríguez-Maldonado; Claudia Wong-Arámbula; Silvia Rivero-Arredondo; Ruth Madera-Sandoval; Joaquín Quiroz-Mercado; Fernando González-Domínguez; Lucia Hernández-Rivas, Irma López-Martínez; Ernesto Ramírez-González; Maribel González-Villa
EPI_ISL_17672233	LESP Quintana Roo	Instituto de Diagnostico y Referencia Epidemiologicos (INDRE)	Abril Rodríguez-Maldonado; Claudia Wong-Arámbula; Silvia Rivero-Arredondo; Ruth Madera-Sandoval; Joaquín Quiroz-Mercado; Fernando González-Domínguez; Lucia Hernández-Rivas, Irma López-Martínez; Ernesto Ramírez-González; Maribel González-Villa
EPI_ISL_17672234	LESP Jalisco	Instituto de Diagnostico y Referencia Epidemiologicos (INDRE)	Abril Rodríguez-Maldonado; Claudia Wong-Arámbula; Silvia Rivero-Arredondo; Ruth Madera-Sandoval; Joaquín Quiroz-Mercado; Fernando González-Domínguez; Lucia Hernández-Rivas, Irma López-Martínez; Ernesto Ramírez-González; Maribel González-Villa
EPI_ISL_17672235	LESP State of Mexico	Instituto de Diagnostico y Referencia Epidemiologicos (INDRE)	Abril Rodríguez-Maldonado; Claudia Wong-Arámbula; Silvia Rivero-Arredondo; Ruth Madera-Sandoval; Joaquín Quiroz-Mercado; Fernando González-Domínguez; Lucia Hernández-Rivas, Irma López-Martínez; Ernesto Ramírez-González; Maribel González-Villa
EPI_ISL_17672236	Laboratorio Estatal de Salud Pública Puebla	Instituto de Diagnostico y Referencia Epidemiologicos (INDRE)	Abril Rodríguez-Maldonado; Claudia Wong-Arámbula; Silvia Rivero-Arredondo; Ruth Madera-Sandoval; Joaquín Quiroz-Mercado; Fernando González-Domínguez; Lucia Hernández-Rivas, Irma López-Martínez; Ernesto Ramírez-González; Maribel González-Villa
EPI_ISL_17672237	LESP Campeche	Instituto de Diagnostico y Referencia Epidemiologicos (INDRE)	Abril Rodríguez-Maldonado; Claudia Wong-Arámbula; Silvia Rivero-Arredondo; Ruth Madera-Sandoval; Joaquín Quiroz-Mercado; Fernando González-Domínguez; Lucia Hernández-Rivas, Irma López-Martínez; Ernesto Ramírez-González; Maribel González-Villa
EPI_ISL_17672238	LESP State of Mexico	Instituto de Diagnostico y Referencia Epidemiologicos (INDRE)	Abril Rodríguez-Maldonado; Claudia Wong-Arámbula; Silvia Rivero-Arredondo; Ruth Madera-Sandoval; Joaquín Quiroz-Mercado; Fernando González-Domínguez; Lucia Hernández-Rivas, Irma López-Martínez; Ernesto Ramírez-González; Maribel González-Villa
EPI_ISL_17672239	LESP Chiapas	Instituto de Diagnostico y Referencia Epidemiologicos (INDRE)	Abril Rodríguez-Maldonado; Claudia Wong-Arámbula; Silvia Rivero-Arredondo; Ruth Madera-Sandoval; Joaquín Quiroz-Mercado; Fernando González-Domínguez; Lucia Hernández-Rivas, Irma López-Martínez; Ernesto Ramírez-González; Maribel González-Villa
EPI_ISL_17672240	LESP Coahuila	Instituto de Diagnostico y Referencia Epidemiologicos (INDRE)	Abril Rodríguez-Maldonado; Claudia Wong-Arámbula; Silvia Rivero-Arredondo; Ruth Madera-Sandoval; Joaquín Quiroz-Mercado; Fernando González-Domínguez; Lucia Hernández-Rivas, Irma López-Martínez; Ernesto Ramírez-González; Maribel González-Villa
EPI_ISL_17672241	LESP Jalisco	Instituto de Diagnostico y Referencia Epidemiologicos (INDRE)	Abril Rodríguez-Maldonado; Claudia Wong-Arámbula; Silvia Rivero-Arredondo; Ruth Madera-Sandoval; Joaquín Quiroz-Mercado; Fernando González-Domínguez; Lucia Hernández-Rivas, Irma López-Martínez; Ernesto Ramírez-González; Maribel González-Villa
EPI_ISL_17672242	LESP Nuevo Leon	Instituto de Diagnostico y Referencia Epidemiologicos (INDRE)	Abril Rodríguez-Maldonado; Claudia Wong-Arámbula; Silvia Rivero-Arredondo; Ruth Madera-Sandoval; Joaquín Quiroz-Mercado; Fernando González-Domínguez; Lucia Hernández-Rivas, Irma López-Martínez; Ernesto Ramírez-González; Maribel González-Villa
EPI_ISL_17672243	LESP Queretaro	Instituto de Diagnostico y Referencia Epidemiologicos (INDRE)	Abril Rodríguez-Maldonado; Claudia Wong-Arámbula; Silvia Rivero-Arredondo; Ruth Madera-Sandoval; Joaquín Quiroz-Mercado; Fernando González-Domínguez; Lucia Hernández-Rivas, Irma López-Martínez; Ernesto Ramírez-González; Maribel González-Villa
EPI_ISL_17672244	LESP Hidalgo	Instituto de Diagnostico y Referencia Epidemiologicos (INDRE)	Abril Rodríguez-Maldonado; Claudia Wong-Arámbula; Silvia Rivero-Arredondo; Ruth Madera-Sandoval; Joaquín Quiroz-Mercado; Fernando González-Domínguez; Lucia Hernández-Rivas, Irma López-Martínez; Ernesto Ramírez-González; Maribel González-Villa
EPI_ISL_17672245	LESP Tamaulipas	Instituto de Diagnostico y Referencia Epidemiologicos	Abril Rodríguez-Maldonado; Claudia Wong-Arámbula; Silvia Rivero-Arredondo; Ruth Madera-Sandoval; Joaquín Quiroz-Mercado; Fernando González-Domínguez; Lucia Hernández-Rivas, Irma López-Martínez; Ernesto Ramírez-González;

		(INDRE)	Maribel González-Villa
EPI_ISL_17672246	LESP Veracruz	Instituto de Diagnostico y Referencia Epidemiologicos (INDRE)	Abril Rodríguez-Maldonado; Claudia Wong-Arámbula; Silvia Rivero-Arredondo; Ruth Madera-Sandoval; Joaquín Quiroz-Mercado; Fernando González-Domínguez; Lucia Hernández-Rivas, Irma López-Martínez; Ernesto Ramírez-González; Maribel González-Villa
EPI_ISL_17672247	LESP Quintana Roo	Instituto de Diagnostico y Referencia Epidemiologicos (INDRE)	Abril Rodríguez-Maldonado; Claudia Wong-Arámbula; Silvia Rivero-Arredondo; Ruth Madera-Sandoval; Joaquín Quiroz-Mercado; Fernando González-Domínguez; Lucia Hernández-Rivas, Irma López-Martínez; Ernesto Ramírez-González; Maribel González-Villa
EPI_ISL_17672248	LESP Queretaro	Instituto de Diagnostico y Referencia Epidemiologicos (INDRE)	Abril Rodríguez-Maldonado; Claudia Wong-Arámbula; Silvia Rivero-Arredondo; Ruth Madera-Sandoval; Joaquín Quiroz-Mercado; Fernando González-Domínguez; Lucia Hernández-Rivas, Irma López-Martínez; Ernesto Ramírez-González; Maribel González-Villa
EPI_ISL_17672249	LESP Oaxaca	Instituto de Diagnostico y Referencia Epidemiologicos (INDRE)	Abril Rodríguez-Maldonado; Claudia Wong-Arámbula; Silvia Rivero-Arredondo; Ruth Madera-Sandoval; Joaquín Quiroz-Mercado; Fernando González-Domínguez; Lucia Hernández-Rivas, Irma López-Martínez; Ernesto Ramírez-González; Maribel González-Villa
EPI_ISL_17672250	LESP Yucatan	Instituto de Diagnostico y Referencia Epidemiologicos (INDRE)	Abril Rodríguez-Maldonado; Claudia Wong-Arámbula; Silvia Rivero-Arredondo; Ruth Madera-Sandoval; Joaquín Quiroz-Mercado; Fernando González-Domínguez; Lucia Hernández-Rivas, Irma López-Martínez; Ernesto Ramírez-González; Maribel González-Villa
EPI_ISL_17672251	LESP Guanajuato	Instituto de Diagnostico y Referencia Epidemiologicos (INDRE)	Abril Rodríguez-Maldonado; Claudia Wong-Arámbula; Silvia Rivero-Arredondo; Ruth Madera-Sandoval; Joaquín Quiroz-Mercado; Fernando González-Domínguez; Lucia Hernández-Rivas, Irma López-Martínez; Ernesto Ramírez-González; Maribel González-Villa
EPI_ISL_17672252	LESP Veracruz	Instituto de Diagnostico y Referencia Epidemiologicos (INDRE)	Abril Rodríguez-Maldonado; Claudia Wong-Arámbula; Silvia Rivero-Arredondo; Ruth Madera-Sandoval; Joaquín Quiroz-Mercado; Fernando González-Domínguez; Lucia Hernández-Rivas, Irma López-Martínez; Ernesto Ramírez-González; Maribel González-Villa
EPI_ISL_17672253	LESP Mexico City	Instituto de Diagnostico y Referencia Epidemiologicos (INDRE)	Abril Rodríguez-Maldonado; Claudia Wong-Arámbula; Silvia Rivero-Arredondo; Ruth Madera-Sandoval; Joaquín Quiroz-Mercado; Fernando González-Domínguez; Lucia Hernández-Rivas, Irma López-Martínez; Ernesto Ramírez-González; Maribel González-Villa
EPI_ISL_17672254	LESP Durango	Instituto de Diagnostico y Referencia Epidemiologicos (INDRE)	Abril Rodríguez-Maldonado; Claudia Wong-Arámbula; Silvia Rivero-Arredondo; Ruth Madera-Sandoval; Joaquín Quiroz-Mercado; Fernando González-Domínguez; Lucia Hernández-Rivas, Irma López-Martínez; Ernesto Ramírez-González; Maribel González-Villa
EPI_ISL_17672255	LESP San Luis Potosi	Instituto de Diagnostico y Referencia Epidemiologicos (INDRE)	Abril Rodríguez-Maldonado; Claudia Wong-Arámbula; Silvia Rivero-Arredondo; Ruth Madera-Sandoval; Joaquín Quiroz-Mercado; Fernando González-Domínguez; Lucia Hernández-Rivas, Irma López-Martínez; Ernesto Ramírez-González; Maribel González-Villa
EPI_ISL_17672256	LESP Nuevo Leon	Instituto de Diagnostico y Referencia Epidemiologicos (INDRE)	Abril Rodríguez-Maldonado; Claudia Wong-Arámbula; Silvia Rivero-Arredondo; Ruth Madera-Sandoval; Joaquín Quiroz-Mercado; Fernando González-Domínguez; Lucia Hernández-Rivas, Irma López-Martínez; Ernesto Ramírez-González; Maribel González-Villa
EPI_ISL_17672257, EPI_ISL_17672258	LESP Quintana Roo	Instituto de Diagnostico y Referencia Epidemiologicos (INDRE)	Abril Rodríguez-Maldonado; Claudia Wong-Arámbula; Silvia Rivero-Arredondo; Ruth Madera-Sandoval; Joaquín Quiroz-Mercado; Fernando González-Domínguez; Lucia Hernández-Rivas, Irma López-Martínez; Ernesto Ramírez-González; Maribel González-Villa
EPI_ISL_17672259	LESP Mexico City	Instituto de Diagnostico y Referencia Epidemiologicos (INDRE)	Abril Rodríguez-Maldonado; Claudia Wong-Arámbula; Silvia Rivero-Arredondo; Ruth Madera-Sandoval; Joaquín Quiroz-Mercado; Fernando González-Domínguez; Lucia Hernández-Rivas, Irma López-Martínez; Ernesto Ramírez-González; Maribel González-Villa
EPI_ISL_17672260	LESP Veracruz	Instituto de Diagnostico y Referencia Epidemiologicos (INDRE)	Abril Rodríguez-Maldonado; Claudia Wong-Arámbula; Silvia Rivero-Arredondo; Ruth Madera-Sandoval; Joaquín Quiroz-Mercado; Fernando González-Domínguez; Lucia Hernández-Rivas, Irma López-Martínez; Ernesto Ramírez-González; Maribel González-Villa
EPI_ISL_17692268, EPI_ISL_17692269	Tokyo Metropolitan Institute of Public Health	Tokyo Metropolitan Institute of Public Health	Fumi Kasuya, Wakaba Okada, Ryota Kumagai, Sachiko Harada, Arisa Amano, Michiya Hasegawa, Mami Nagashima, Kenji Sadamasu
EPI_ISL_17703564, EPI_ISL_17703565	LESP State of Mexico	Instituto de Diagnostico y Referencia Epidemiologicos (INDRE)	Abril Rodríguez-Maldonado; Claudia Wong-Arámbula; Felipe Arguijo-Perez; Helios Cárdenas-Hernández; Carmen Castro-Méndez; Lidia García-Torres; Ruth Madera-Sandoval; América Mandujano-Martínez; Nancy Martínez-Velázquez; Mireya Mederos-Michel; Angélica Pedraza-Meléndez; Joaquín Quiroz-Mercado; Daniel Regalado-Santiago; Silvia Rivero-Arredondo; Erika Sierra-Atanacio; Fernando González-Domínguez; Lucia Hernández-Rivas, Irma López-Martínez; Ernesto Ramírez-González; Maribel González-Villa
EPI_ISL_17703566	LESP Jalisco	Instituto de Diagnostico y Referencia Epidemiologicos (INDRE)	Abril Rodríguez-Maldonado; Claudia Wong-Arámbula; Felipe Arguijo-Perez; Helios Cárdenas-Hernández; Carmen Castro-Méndez; Lidia García-Torres; Ruth Madera-Sandoval; América Mandujano-Martínez; Nancy Martínez-Velázquez; Mireya Mederos-Michel; Angélica Pedraza-Meléndez; Joaquín Quiroz-Mercado; Daniel Regalado-Santiago; Silvia Rivero-Arredondo; Erika Sierra-Atanacio; Fernando González-Domínguez; Lucia Hernández-Rivas, Irma López-Martínez; Ernesto Ramírez-González; Maribel González-Villa
EPI_ISL_17703567	LESP State of Mexico	Instituto de Diagnostico y Referencia Epidemiologicos (INDRE)	Abril Rodríguez-Maldonado; Claudia Wong-Arámbula; Felipe Arguijo-Perez; Helios Cárdenas-Hernández; Carmen Castro-Méndez; Lidia García-Torres; Ruth Madera-Sandoval; América Mandujano-Martínez; Nancy Martínez-Velázquez; Mireya Mederos-Michel; Angélica Pedraza-Meléndez; Joaquín Quiroz-Mercado; Daniel Regalado-Santiago; Silvia Rivero-Arredondo; Erika Sierra-Atanacio; Fernando González-Domínguez; Lucia Hernández-Rivas, Irma López-Martínez; Ernesto Ramírez-González; Maribel González-Villa
EPI_ISL_17703568	LESP Quintana Roo	Instituto de Diagnostico y Referencia Epidemiologicos (INDRE)	Abril Rodríguez-Maldonado; Claudia Wong-Arámbula; Felipe Arguijo-Perez; Helios Cárdenas-Hernández; Carmen Castro-Méndez; Lidia García-Torres; Ruth Madera-Sandoval; América Mandujano-Martínez; Nancy Martínez-Velázquez; Mireya Mederos-Michel; Angélica Pedraza-Meléndez; Joaquín Quiroz-Mercado; Daniel Regalado-Santiago; Silvia Rivero-Arredondo; Erika Sierra-Atanacio; Fernando González-Domínguez; Lucia Hernández-Rivas, Irma López-Martínez; Ernesto Ramírez-González; Maribel González-Villa
EPI_ISL_17703570	LESP Mexico City	Instituto de Diagnostico y Referencia Epidemiologicos (INDRE)	Abril Rodríguez-Maldonado; Claudia Wong-Arámbula; Felipe Arguijo-Perez; Helios Cárdenas-Hernández; Carmen Castro-Méndez; Lidia García-Torres; Ruth Madera-Sandoval; América Mandujano-Martínez; Nancy Martínez-Velázquez; Mireya Mederos-Michel; Angélica Pedraza-Meléndez; Joaquín Quiroz-Mercado; Daniel Regalado-Santiago; Silvia Rivero-Arredondo; Erika Sierra-Atanacio; Fernando González-Domínguez; Lucia Hernández-Rivas, Irma López-Martínez; Ernesto Ramírez-González; Maribel González-Villa
EPI_ISL_17703571	LESP Sinaloa	Instituto de Diagnostico y Referencia Epidemiologicos (INDRE)	Abril Rodríguez-Maldonado; Claudia Wong-Arámbula; Felipe Arguijo-Perez; Helios Cárdenas-Hernández; Carmen Castro-Méndez; Lidia García-Torres; Ruth Madera-Sandoval; América Mandujano-Martínez; Nancy Martínez-Velázquez; Mireya Mederos-Michel; Angélica Pedraza-Meléndez; Joaquín Quiroz-Mercado; Daniel Regalado-Santiago; Silvia Rivero-Arredondo; Erika Sierra-Atanacio; Fernando González-Domínguez; Lucia Hernández-Rivas, Irma López-Martínez; Ernesto Ramírez-González; Maribel González-Villa
EPI_ISL_17703572	LESP State of Mexico	Instituto de Diagnostico y Referencia Epidemiologicos (INDRE)	Abril Rodríguez-Maldonado; Claudia Wong-Arámbula; Felipe Arguijo-Perez; Helios Cárdenas-Hernández; Carmen Castro-Méndez; Lidia García-Torres; Ruth Madera-Sandoval; América Mandujano-Martínez; Nancy Martínez-Velázquez; Mireya Mederos-Michel; Angélica Pedraza-Meléndez; Joaquín Quiroz-Mercado; Daniel Regalado-Santiago; Silvia Rivero-Arredondo; Erika Sierra-Atanacio; Fernando González-Domínguez; Lucia Hernández-Rivas, Irma López-Martínez; Ernesto Ramírez-González; Maribel González-Villa
EPI_ISL_17703573	LESP Mexico City	Instituto de Diagnostico y Referencia Epidemiologicos (INDRE)	Abril Rodríguez-Maldonado; Claudia Wong-Arámbula; Felipe Arguijo-Perez; Helios Cárdenas-Hernández; Carmen Castro-Méndez; Lidia García-Torres; Ruth Madera-Sandoval; América Mandujano-Martínez; Nancy Martínez-Velázquez; Mireya Mederos-Michel; Angélica Pedraza-Meléndez; Joaquín Quiroz-Mercado; Daniel Regalado-Santiago; Silvia Rivero-Arredondo; Erika Sierra-Atanacio; Fernando González-Domínguez; Lucia Hernández-Rivas, Irma López-Martínez; Ernesto Ramírez-González; Maribel González-Villa
EPI_ISL_17703574	LESP State of Mexico	Instituto de Diagnostico y Referencia Epidemiologicos (INDRE)	Abril Rodríguez-Maldonado; Claudia Wong-Arámbula; Felipe Arguijo-Perez; Helios Cárdenas-Hernández; Carmen Castro-Méndez; Lidia García-Torres; Ruth Madera-Sandoval; América Mandujano-Martínez; Nancy Martínez-Velázquez; Mireya Mederos-Michel; Angélica Pedraza-Meléndez; Joaquín Quiroz-Mercado; Daniel Regalado-Santiago; Silvia Rivero-Arredondo; Erika Sierra-Atanacio; Fernando González-Domínguez; Lucia Hernández-Rivas, Irma López-Martínez; Ernesto Ramírez-González; Maribel González-Villa
EPI_ISL_17703575	LESP Mexico City	Instituto de Diagnostico y Referencia Epidemiologicos (INDRE)	Abril Rodríguez-Maldonado; Claudia Wong-Arámbula; Felipe Arguijo-Perez; Helios Cárdenas-Hernández; Carmen Castro-Méndez; Lidia García-Torres; Ruth Madera-Sandoval; América Mandujano-Martínez; Nancy Martínez-Velázquez; Mireya Mederos-Michel; Angélica Pedraza-Meléndez; Joaquín Quiroz-Mercado; Daniel Regalado-Santiago; Silvia Rivero-Arredondo; Erika Sierra-Atanacio; Fernando González-Domínguez; Lucia Hernández-Rivas, Irma López-Martínez; Ernesto Ramírez-González; Maribel González-Villa
EPI_ISL_17703576	LESP Quintana Roo	Instituto de Diagnostico y Referencia Epidemiologicos (INDRE)	Abril Rodríguez-Maldonado; Claudia Wong-Arámbula; Felipe Arguijo-Perez; Helios Cárdenas-Hernández; Carmen Castro-Méndez; Lidia García-Torres; Ruth Madera-Sandoval; América Mandujano-Martínez; Nancy Martínez-Velázquez; Mireya Mederos-Michel; Angélica Pedraza-Meléndez; Joaquín Quiroz-Mercado; Daniel Regalado-Santiago; Silvia Rivero-Arredondo; Erika Sierra-Atanacio; Fernando González-Domínguez; Lucia Hernández-Rivas, Irma López-Martínez; Ernesto Ramírez-González; Maribel González-Villa
EPI_ISL_17703577	LESP Mexico City	Instituto de Diagnostico y Referencia Epidemiologicos (INDRE)	Abril Rodríguez-Maldonado; Claudia Wong-Arámbula; Felipe Arguijo-Perez; Helios Cárdenas-Hernández; Carmen Castro-Méndez; Lidia García-Torres; Ruth Madera-Sandoval; América Mandujano-Martínez; Nancy Martínez-Velázquez; Mireya Mederos-Michel; Angélica Pedraza-Meléndez; Joaquín Quiroz-Mercado; Daniel Regalado-Santiago; Silvia Rivero-Arredondo; Erika Sierra-Atanacio; Fernando González-Domínguez; Lucia Hernández-Rivas, Irma López-Martínez; Ernesto Ramírez-González; Maribel González-Villa
EPI_ISL_17703578	LESP Yucatan	Instituto de Diagnostico y Referencia Epidemiologicos (INDRE)	Abril Rodríguez-Maldonado; Claudia Wong-Arámbula; Felipe Arguijo-Perez; Helios Cárdenas-Hernández; Carmen Castro-Méndez; Lidia García-Torres; Ruth Madera-Sandoval; América Mandujano-Martínez; Nancy Martínez-Velázquez; Mireya Mederos-Michel; Angélica Pedraza-Meléndez; Joaquín Quiroz-Mercado; Daniel Regalado-Santiago; Silvia Rivero-Arredondo; Erika Sierra-Atanacio; Fernando González-Domínguez; Lucia Hernández-Rivas, Irma López-Martínez; Ernesto Ramírez-González; Maribel González-Villa
EPI_ISL_17703579	LESP Quintana Roo	Instituto de Diagnostico y Referencia Epidemiologicos (INDRE)	Abril Rodríguez-Maldonado; Claudia Wong-Arámbula; Felipe Arguijo-Perez; Helios Cárdenas-Hernández; Carmen Castro-Méndez; Lidia García-Torres; Ruth Madera-Sandoval; América Mandujano-Martínez; Nancy Martínez-Velázquez; Mireya Mederos-Michel; Angélica Pedraza-Meléndez; Joaquín Quiroz-Mercado; Daniel Regalado-Santiago; Silvia Rivero-Arredondo; Erika Sierra-Atanacio; Fernando González-Domínguez; Lucia Hernández-Rivas, Irma López-Martínez; Ernesto Ramírez-González; Maribel González-Villa
EPI_ISL_17703580	LESP Hidalgo	Instituto de Diagnostico y Referencia Epidemiologicos (INDRE)	Abril Rodríguez-Maldonado; Claudia Wong-Arámbula; Felipe Arguijo-Perez; Helios Cárdenas-Hernández; Carmen Castro-Méndez; Lidia García-Torres; Ruth Madera-Sandoval; América Mandujano-Martínez; Nancy Martínez-Velázquez; Mireya Mederos-Michel; Angélica Pedraza-Meléndez; Joaquín Quiroz-Mercado; Daniel Regalado-Santiago; Silvia Rivero-Arredondo; Erika Sierra-Atanacio; Fernando González-Domínguez; Lucia Hernández-Rivas, Irma López-Martínez; Ernesto Ramírez-González; Maribel González-Villa
EPI_ISL_17703581	LESP Yucatan	Instituto de Diagnostico y Referencia Epidemiologicos (INDRE)	Abril Rodríguez-Maldonado; Claudia Wong-Arámbula; Felipe Arguijo-Perez; Helios Cárdenas-Hernández; Carmen Castro-Méndez; Lidia García-Torres; Ruth Madera-Sandoval; América Mandujano-Martínez; Nancy Martínez-Velázquez; Mireya Mederos-Michel; Angélica Pedraza-Meléndez; Joaquín Quiroz-Mercado; Daniel Regalado-Santiago; Silvia Rivero-Arredondo; Erika Sierra-Atanacio; Fernando González-Domínguez; Lucia Hernández-Rivas, Irma López-Martínez; Ernesto Ramírez-González; Maribel González-Villa
EPI_ISL_17703582	LESP Nuevo Leon	Instituto de Diagnostico y Referencia Epidemiologicos (INDRE)	Abril Rodríguez-Maldonado; Claudia Wong-Arámbula; Felipe Arguijo-Perez; Helios Cárdenas-Hernández; Carmen Castro-Méndez; Lidia García-Torres; Ruth Madera-Sandoval; América Mandujano-Martínez; Nancy Martínez-Velázquez; Mireya Mederos-Michel; Angélica Pedraza-Meléndez; Joaquín Quiroz-Mercado; Daniel Regalado-Santiago; Silvia Rivero-Arredondo; Erika Sierra-Atanacio; Fernando González-Domínguez; Lucia Hernández-Rivas, Irma López-Martínez; Ernesto Ramírez-González; Maribel González-Villa
EPI_ISL_17703583	LESP Quintana Roo	Instituto de Diagnostico y Referencia Epidemiologicos (INDRE)	Abril Rodríguez-Maldonado; Claudia Wong-Arámbula; Felipe Arguijo-Perez; Helios Cárdenas-Hernández; Carmen Castro-Méndez; Lidia García-Torres; Ruth Madera-Sandoval; América Mandujano-Martínez; Nancy Martínez-Velázquez; Mireya Mederos-Michel; Angélica Pedraza-Meléndez; Joaquín Quiroz-Mercado; Daniel Regalado-Santiago; Silvia Rivero-Arredondo; Erika Sierra-Atanacio; Fernando González-Domínguez; Lucia Hernández-Rivas, Irma López-Martínez; Ernesto Ramírez-González; Maribel González-Villa
EPI_ISL_17703584	LESP Chihuahua	Instituto de Diagnostico y Referencia Epidemiologicos (INDRE)	Abril Rodríguez-Maldonado; Claudia Wong-Arámbula; Felipe Arguijo-Perez; Helios Cárdenas-Hernández; Carmen Castro-Méndez; Lidia García-Torres; Ruth Madera-Sandoval; América Mandujano-Martínez; Nancy Martínez-Velázquez; Mireya Mederos-Michel; Angélica Pedraza-Meléndez; Joaquín Quiroz-Mercado; Daniel Regalado-Santiago; Silvia Rivero-Arredondo; Erika Sierra-Atanacio; Fernando González-Domínguez; Lucia Hernández-Rivas, Irma López-Martínez; Ernesto Ramírez-González; Maribel González-Villa
EPI_ISL_17703585	LESP Yucatan	Instituto de Diagnostico y Referencia Epidemiologicos (INDRE)	Abril Rodríguez-Maldonado; Claudia Wong-Arámbula; Felipe Arguijo-Perez; Helios Cárdenas-Hernández; Carmen Castro-Méndez; Lidia García-Torres; Ruth Madera-Sandoval; América Mandujano-Martínez; Nancy Martínez-Velázquez; Mireya Mederos-Michel; Angélica Pedraza-Meléndez; Joaquín Quiroz-Mercado; Daniel Regalado-Santiago; Silvia Rivero-Arredondo; Erika Sierra-Atanacio; Fernando González-Domínguez; Lucia Hernández-Rivas, Irma López-Martínez; Ernesto Ramírez-González; Maribel González-Villa
EPI_ISL_17703586, EPI_ISL_17703587	LESP Nuevo Leon	Instituto de Diagnostico y Referencia Epidemiologicos (INDRE)	Abril Rodríguez-Maldonado; Claudia Wong-Arámbula; Felipe Arguijo-Perez; Helios Cárdenas-Hernández; Carmen Castro-Méndez; Lidia García-Torres; Ruth Madera-Sandoval; América Mandujano-Martínez; Nancy Martínez-Velázquez; Mireya Mederos-Michel; Angélica Pedraza-Meléndez; Joaquín Quiroz-Mercado; Daniel Regalado-Santiago; Silvia Rivero-Arredondo; Erika Sierra-Atanacio; Fernando González-Domínguez; Lucia Hernández-Rivas, Irma López-Martínez; Ernesto Ramírez-González; Maribel González-Villa
EPI_ISL_17703588, EPI_ISL_17703589	LESP Quintana Roo	Instituto de Diagnostico y Referencia Epidemiologicos	Abril Rodríguez-Maldonado; Claudia Wong-Arámbula; Felipe Arguijo-Perez; Helios Cárdenas-Hernández; Carmen Castro-Méndez; Lidia García-Torres; Ruth Madera-Sandoval; América Mandujano-Martínez; Nancy Martínez-Velázquez;

		(INDRE)	Mireya Mederos-Michel; Angélica Pedraza-Meléndez; Joaquín Quiroz-Mercado; Daniel Regalado-Santiago; Silvia Rivero-Arredondo; Erika Sierra-Atanacio; Fernando González-Domínguez; Lucia Hernández-Rivas, Irma López-Martínez; Ernesto Ramírez-González; Maribel González-Villa
EPI_ISL_17703590	LESP State of Mexico	Instituto de Diagnostico y Referencia Epidemiologicos (INDRE)	Abril Rodríguez-Maldonado; Claudia Wong-Arámbula; Felipe Arguijo-Perez; Helios Cárdenas-Hernández; Carmen Castro-Méndez; Lidia García-Torres; Ruth Madera-Sandoval; América Mandujano-Martínez; Nancy Martínez-Velázquez; Mireya Mederos-Michel; Angélica Pedraza-Meléndez; Joaquín Quiroz-Mercado; Daniel Regalado-Santiago; Silvia Rivero-Arredondo; Erika Sierra-Atanacio; Fernando González-Domínguez; Lucia Hernández-Rivas, Irma López-Martínez; Ernesto Ramírez-González; Maribel González-Villa
EPI_ISL_17703591	LESP Campeche	Instituto de Diagnostico y Referencia Epidemiologicos (INDRE)	Abril Rodríguez-Maldonado; Claudia Wong-Arámbula; Felipe Arguijo-Perez; Helios Cárdenas-Hernández; Carmen Castro-Méndez; Lidia García-Torres; Ruth Madera-Sandoval; América Mandujano-Martínez; Nancy Martínez-Velázquez; Mireya Mederos-Michel; Angélica Pedraza-Meléndez; Joaquín Quiroz-Mercado; Daniel Regalado-Santiago; Silvia Rivero-Arredondo; Erika Sierra-Atanacio; Fernando González-Domínguez; Lucia Hernández-Rivas, Irma López-Martínez; Ernesto Ramírez-González; Maribel González-Villa
EPI_ISL_17703592	LESP Nuevo Leon	Instituto de Diagnostico y Referencia Epidemiologicos (INDRE)	Abril Rodríguez-Maldonado; Claudia Wong-Arámbula; Felipe Arguijo-Perez; Helios Cárdenas-Hernández; Carmen Castro-Méndez; Lidia García-Torres; Ruth Madera-Sandoval; América Mandujano-Martínez; Nancy Martínez-Velázquez; Mireya Mederos-Michel; Angélica Pedraza-Meléndez; Joaquín Quiroz-Mercado; Daniel Regalado-Santiago; Silvia Rivero-Arredondo; Erika Sierra-Atanacio; Fernando González-Domínguez; Lucia Hernández-Rivas, Irma López-Martínez; Ernesto Ramírez-González; Maribel González-Villa
EPI_ISL_17703593, EPI_ISL_17703594	LESP Jalisco	Instituto de Diagnostico y Referencia Epidemiologicos (INDRE)	Abril Rodríguez-Maldonado; Claudia Wong-Arámbula; Felipe Arguijo-Perez; Helios Cárdenas-Hernández; Carmen Castro-Méndez; Lidia García-Torres; Ruth Madera-Sandoval; América Mandujano-Martínez; Nancy Martínez-Velázquez; Mireya Mederos-Michel; Angélica Pedraza-Meléndez; Joaquín Quiroz-Mercado; Daniel Regalado-Santiago; Silvia Rivero-Arredondo; Erika Sierra-Atanacio; Fernando González-Domínguez; Lucia Hernández-Rivas, Irma López-Martínez; Ernesto Ramírez-González; Maribel González-Villa
EPI_ISL_17703595, EPI_ISL_17703596	LESP Nuevo Leon	Instituto de Diagnostico y Referencia Epidemiologicos (INDRE)	Abril Rodríguez-Maldonado; Claudia Wong-Arámbula; Felipe Arguijo-Perez; Helios Cárdenas-Hernández; Carmen Castro-Méndez; Lidia García-Torres; Ruth Madera-Sandoval; América Mandujano-Martínez; Nancy Martínez-Velázquez; Mireya Mederos-Michel; Angélica Pedraza-Meléndez; Joaquín Quiroz-Mercado; Daniel Regalado-Santiago; Silvia Rivero-Arredondo; Erika Sierra-Atanacio; Fernando González-Domínguez; Lucia Hernández-Rivas, Irma López-Martínez; Ernesto Ramírez-González; Maribel González-Villa
EPI_ISL_17703597	Laboratorio Estatal de Salud Pública Nuevo Leon	Instituto de Diagnostico y Referencia Epidemiologicos (INDRE)	Abril Rodríguez-Maldonado; Claudia Wong-Arámbula; Felipe Arguijo-Perez; Helios Cárdenas-Hernández; Carmen Castro-Méndez; Lidia García-Torres; Ruth Madera-Sandoval; América Mandujano-Martínez; Nancy Martínez-Velázquez; Mireya Mederos-Michel; Angélica Pedraza-Meléndez; Joaquín Quiroz-Mercado; Daniel Regalado-Santiago; Silvia Rivero-Arredondo; Erika Sierra-Atanacio; Fernando González-Domínguez; Lucia Hernández-Rivas, Irma López-Martínez; Ernesto Ramírez-González; Maribel González-Villa
EPI_ISL_17703598	LESP Quintana Roo	Instituto de Diagnostico y Referencia Epidemiologicos (INDRE)	Abril Rodríguez-Maldonado; Claudia Wong-Arámbula; Felipe Arguijo-Perez; Helios Cárdenas-Hernández; Carmen Castro-Méndez; Lidia García-Torres; Ruth Madera-Sandoval; América Mandujano-Martínez; Nancy Martínez-Velázquez; Mireya Mederos-Michel; Angélica Pedraza-Meléndez; Joaquín Quiroz-Mercado; Daniel Regalado-Santiago; Silvia Rivero-Arredondo; Erika Sierra-Atanacio; Fernando González-Domínguez; Lucia Hernández-Rivas, Irma López-Martínez; Ernesto Ramírez-González; Maribel González-Villa
EPI_ISL_17703599, EPI_ISL_17703600, EPI_ISL_17703601, EPI_ISL_17703602, EPI_ISL_17703603, EPI_ISL_17703604	LESP Nuevo Leon	Instituto de Diagnostico y Referencia Epidemiologicos (INDRE)	Abril Rodríguez-Maldonado; Claudia Wong-Arámbula; Felipe Arguijo-Perez; Helios Cárdenas-Hernández; Carmen Castro-Méndez; Lidia García-Torres; Ruth Madera-Sandoval; América Mandujano-Martínez; Nancy Martínez-Velázquez; Mireya Mederos-Michel; Angélica Pedraza-Meléndez; Joaquín Quiroz-Mercado; Daniel Regalado-Santiago; Silvia Rivero-Arredondo; Erika Sierra-Atanacio; Fernando González-Domínguez; Lucia Hernández-Rivas, Irma López-Martínez; Ernesto Ramírez-González; Maribel González-Villa
EPI_ISL_17703605	LESP Chihuahua	Instituto de Diagnostico y Referencia Epidemiologicos (INDRE)	Abril Rodríguez-Maldonado; Claudia Wong-Arámbula; Felipe Arguijo-Perez; Helios Cárdenas-Hernández; Carmen Castro-Méndez; Lidia García-Torres; Ruth Madera-Sandoval; América Mandujano-Martínez; Nancy Martínez-Velázquez; Mireya Mederos-Michel; Angélica Pedraza-Meléndez; Joaquín Quiroz-Mercado; Daniel Regalado-Santiago; Silvia Rivero-Arredondo; Erika Sierra-Atanacio; Fernando González-Domínguez; Lucia Hernández-Rivas, Irma López-Martínez; Ernesto Ramírez-González; Maribel González-Villa
EPI_ISL_17703606	LESP Nuevo Leon	Instituto de Diagnostico y Referencia Epidemiologicos (INDRE)	Abril Rodríguez-Maldonado; Claudia Wong-Arámbula; Felipe Arguijo-Perez; Helios Cárdenas-Hernández; Carmen Castro-Méndez; Lidia García-Torres; Ruth Madera-Sandoval; América Mandujano-Martínez; Nancy Martínez-Velázquez; Mireya Mederos-Michel; Angélica Pedraza-Meléndez; Joaquín Quiroz-Mercado; Daniel Regalado-Santiago; Silvia Rivero-Arredondo; Erika Sierra-Atanacio; Fernando González-Domínguez; Lucia Hernández-Rivas, Irma López-Martínez; Ernesto Ramírez-González; Maribel González-Villa
EPI_ISL_17703607, EPI_ISL_17703608, EPI_ISL_17703609, EPI_ISL_17703610	LESP Mexico City	Instituto de Diagnostico y Referencia Epidemiologicos (INDRE)	Abril Rodríguez-Maldonado; Claudia Wong-Arámbula; Felipe Arguijo-Perez; Helios Cárdenas-Hernández; Carmen Castro-Méndez; Lidia García-Torres; Ruth Madera-Sandoval; América Mandujano-Martínez; Nancy Martínez-Velázquez; Mireya Mederos-Michel; Angélica Pedraza-Meléndez; Joaquín Quiroz-Mercado; Daniel Regalado-Santiago; Silvia Rivero-Arredondo; Erika Sierra-Atanacio; Fernando González-Domínguez; Lucia Hernández-Rivas, Irma López-Martínez; Ernesto Ramírez-González; Maribel González-Villa
EPI_ISL_17703611	LESP Tlaxcala	Instituto de Diagnostico y Referencia Epidemiologicos (INDRE)	Abril Rodríguez-Maldonado; Claudia Wong-Arámbula; Felipe Arguijo-Perez; Helios Cárdenas-Hernández; Carmen Castro-Méndez; Lidia García-Torres; Ruth Madera-Sandoval; América Mandujano-Martínez; Nancy Martínez-Velázquez; Mireya Mederos-Michel; Angélica Pedraza-Meléndez; Joaquín Quiroz-Mercado; Daniel Regalado-Santiago; Silvia Rivero-Arredondo; Erika Sierra-Atanacio; Fernando González-Domínguez; Lucia Hernández-Rivas, Irma López-Martínez; Ernesto Ramírez-González; Maribel González-Villa
EPI_ISL_17703612	LESP Nuevo Leon	Instituto de Diagnostico y Referencia Epidemiologicos (INDRE)	Abril Rodríguez-Maldonado; Claudia Wong-Arámbula; Felipe Arguijo-Perez; Helios Cárdenas-Hernández; Carmen Castro-Méndez; Lidia García-Torres; Ruth Madera-Sandoval; América Mandujano-Martínez; Nancy Martínez-Velázquez; Mireya Mederos-Michel; Angélica Pedraza-Meléndez; Joaquín Quiroz-Mercado; Daniel Regalado-Santiago; Silvia Rivero-Arredondo; Erika Sierra-Atanacio; Fernando González-Domínguez; Lucia Hernández-Rivas, Irma López-Martínez; Ernesto Ramírez-González; Maribel González-Villa
EPI_ISL_17703744	Parasitology Laboratory, Institute of Tropical Medicine of Sao Paulo, School of Medicine, University of Sao Paulo	Parasitology Laboratory	Raissa Heloisa de Araujo Eliodoro, Ingra Claro Morales, Ester Cerdeira Sabino
EPI_ISL_17703745	Parasitology Laboratory, Institute of Tropical Medicine of Sao Paulo, School of Medicine, University of Sao Paulo	Parasitology Laboratory	Raissa Heloisa de Araujo Eliodoro, Ingra Morales Claro, Ester Cerdeira Sabino
EPI_ISL_17703746	Parasitology Laboratory, Institute of Tropical Medicine of Sao Paulo, School of Medicine, University of Sao Paulo	Parasitology Laboratory	Raissa Heloisa de Araujo Eliodoro, Ingra Claro Morales, Ester Cerdeira Sabino
EPI_ISL_17736865, EPI_ISL_17736866, EPI_ISL_17736868, EPI_ISL_17736869, EPI_ISL_17736870, EPI_ISL_17736871, EPI_ISL_17736872, EPI_ISL_17736873, EPI_ISL_17736874, EPI_ISL_17736875, EPI_ISL_17736876, EPI_ISL_17736879, EPI_ISL_17736880, EPI_ISL_17736881, EPI_ISL_17736882, EPI_ISL_17736883, EPI_ISL_17736884, EPI_ISL_17736885, EPI_ISL_17736886, EPI_ISL_17736887, EPI_ISL_17736888, EPI_ISL_17736889, EPI_ISL_17736890, EPI_ISL_17736891			
see above	Charité Universitätsmedizin Berlin, Institute for Virology/Laboratory Berlin	Charité Universitätsmedizin Berlin, Institute for Virology	Terry C. Jones, Julia Melchert, Barbara Mühlemann, Talitha Veith, Jörn Beheim-Schwarzbach, Julia Tesch, Marie Luisa Schmidt, Felix Walper, Tobias Bleicker, Caroline Isner, Frieder Pfäfflin, Ricardo Niklas Werner, Victor M. Corman, Christian Drosten
EPI_ISL_17737466, EPI_ISL_17737467, EPI_ISL_17737468, EPI_ISL_17737469, EPI_ISL_17737470, EPI_ISL_17737471, EPI_ISL_17737472, EPI_ISL_17737473, EPI_ISL_17737474, EPI_ISL_17737476, EPI_ISL_17737478, EPI_ISL_17737479, EPI_ISL_17737480, EPI_ISL_17737481, EPI_ISL_17737482, EPI_ISL_17737484, EPI_ISL_17737485, EPI_ISL_17737488, EPI_ISL_17737490, EPI_ISL_17737491, EPI_ISL_17737492, EPI_ISL_17737493, EPI_ISL_17737495, EPI_ISL_17737496, EPI_ISL_17737497, EPI_ISL_17737500, EPI_ISL_17737501, EPI_ISL_17737502, EPI_ISL_17737503, EPI_ISL_17737504, EPI_ISL_17737505, EPI_ISL_17737506, EPI_ISL_17737507, EPI_ISL_17737508, EPI_ISL_17737509, EPI_ISL_17737511, EPI_ISL_17737512, EPI_ISL_17737513, EPI_ISL_17737514, EPI_ISL_17737515, EPI_ISL_17737516, EPI_ISL_17737517, EPI_ISL_17737518, EPI_ISL_17737519, EPI_ISL_17737520, EPI_ISL_17737521, EPI_ISL_17737522, EPI_ISL_17737523, EPI_ISL_17737524, EPI_ISL_17737525, EPI_ISL_17737526, EPI_ISL_17737527, EPI_ISL_17737528, EPI_ISL_17737530, EPI_ISL_17737531, EPI_ISL_17737532, EPI_ISL_17737533, EPI_ISL_17737534, EPI_ISL_17737535, EPI_ISL_17737536, EPI_ISL_17737537, EPI_ISL_17737538, EPI_ISL_17737539, EPI_ISL_17737540, EPI_ISL_17737542, EPI_ISL_17737543, EPI_ISL_17737547, EPI_ISL_17737548, EPI_ISL_17737550, EPI_ISL_17737553			
see above	Public Health Ontario	Public Health Ontario	Isabel S, Eshaghi A, Duvvuri VR, Gubbay JB, Cronin K, Li A, Hasso M, Clark ST, Hopkins JP, Patel SN, Braukmann TWA
EPI_ISL_17793220	Microbiology, Immunology and Transplantation, KU Leuven	Microbiology, Immunology and Transplantation, KU Leuven	Vanmechelen,B., Wawina-Bokalanga,T., Logist,A.-S., Bloemen,M. and Maes,P.
EPI_ISL_17793221, EPI_ISL_17793226	Microbiology, Immunology and Transplantation, KU Leuven	Microbiology, Immunology and Transplantation, KU Leuven	Wawina-Bokalanga,T., Vanmechelen,B., Logist,A.-S., Bloemen,M. and Maes,P.
EPI_ISL_17793227	Microbiology, Immunology and Transplantation, KU Leuven	Microbiology, Immunology and Transplantation, KU Leuven	Vanmechelen,B., Wawina-Bokalanga,T., Logist,A.-S., Bloemen,M. and Maes,P.
EPI_ISL_17793230	Microbiology, Immunology and Transplantation, KU Leuven	Microbiology, Immunology and Transplantation, KU Leuven	Wawina-Bokalanga,T., Vanmechelen,B., Logist,A.-S., Sinnesael,R., Ysebaert,L., Verlinden,J., Van Holm,B., Bloemen,M. and Maes,P.
EPI_ISL_17793231	Microbiology, Immunology and Transplantation, KU Leuven	Microbiology, Immunology and Transplantation, KU Leuven	Vanmechelen,B., Wawina-Bokalanga,T., Logist,A.-S., Bloemen,M. and Maes,P.
EPI_ISL_17793232	Microbiology, Immunology and Transplantation, KU Leuven	Microbiology, Immunology and Transplantation, KU Leuven	Wawina-Bokalanga,T., Vanmechelen,B., Logist,A.-S., Bloemen,M. and Maes,P.
EPI_ISL_17793234	Microbiology, Immunology and Transplantation, KU Leuven	Microbiology, Immunology and Transplantation, KU Leuven	Vanmechelen,B., Wawina-Bokalanga,T., Logist,A.-S., Bloemen,M. and Maes,P.
EPI_ISL_17793235, EPI_ISL_17793241	Microbiology, Immunology and Transplantation, KU Leuven	Microbiology, Immunology and Transplantation, KU Leuven	Wawina-Bokalanga,T., Vanmechelen,B., Logist,A.-S., Bloemen,M. and Maes,P.
EPI_ISL_17793243, EPI_ISL_17793250	Microbiology, Immunology and Transplantation, KU Leuven	Microbiology, Immunology and Transplantation, KU Leuven	Vanmechelen,B., Wawina-Bokalanga,T., Logist,A.-S., Bloemen,M. and Maes,P.
EPI_ISL_17793251	Microbiology, Immunology and Transplantation, KU Leuven	Microbiology, Immunology and Transplantation, KU Leuven	Wawina-Bokalanga,T., Vanmechelen,B., Logist,A.-S., Bloemen,M. and Maes,P.
EPI_ISL_17793255	Microbiology, Immunology and Transplantation, KU Leuven	Microbiology, Immunology and Transplantation, KU Leuven	Wawina-Bokalanga,T., Vanmechelen,B., Logist,A.-S., Sinnesael,R., Ysebaert,L., Verlinden,J., Van Holm,B., Bloemen,M. and Maes,P.
EPI_ISL_17793256	Microbiology, Immunology and Transplantation, KU Leuven	Microbiology, Immunology and Transplantation, KU Leuven	Vanmechelen,B., Wawina-Bokalanga,T., Logist,A.-S., Bloemen,M. and Maes,P.
EPI_ISL_17793257, EPI_ISL_17793259, EPI_ISL_17793260	Microbiology, Immunology and Transplantation, KU Leuven	Microbiology, Immunology and Transplantation, KU Leuven	Wawina-Bokalanga,T., Vanmechelen,B., Logist,A.-S., Bloemen,M. and Maes,P.
EPI_ISL_17793263, EPI_ISL_17793264, EPI_ISL_17793265	Microbiology, Immunology and Transplantation, KU Leuven	Microbiology, Immunology and Transplantation, KU Leuven	Vanmechelen,B., Wawina-Bokalanga,T., Logist,A.-S., Bloemen,M. and Maes,P.
EPI_ISL_17793271	Microbiology, Immunology and Transplantation, KU Leuven	Microbiology, Immunology and Transplantation, KU Leuven	Wawina-Bokalanga,T., Vanmechelen,B., Logist,A.-S., Bloemen,M., Van Holm,B. and Maes,P.
EPI_ISL_17793273	Microbiology, Immunology and Transplantation, KU Leuven	Microbiology, Immunology and Transplantation, KU Leuven	Wawina-Bokalanga,T., Vanmechelen,B., Logist,A.-S., Bloemen,M. and Maes,P.
EPI_ISL_17793278	Microbiology, Immunology and Transplantation, KU Leuven	Microbiology, Immunology and Transplantation, KU Leuven	Wawina-Bokalanga,T., Mechelen,B., Logist,A.-S., Bloemen,M. and Maes,P.

EPI_ISL_17793280, EPI_ISL_17793286	Microbiology, Immunology and Transplantation, KU Leuven	Microbiology, Immunology and Transplantation, KU Leuven	Vanmechelen,B., Wawina-Bokalanga,T., Logist,A.-S., Bloemen,M. and Maes,P.
EPI_ISL_17793287	Microbiology, Immunology and Transplantation, KU Leuven	Microbiology, Immunology and Transplantation, KU Leuven	Vanmechelen,B., Wawona-Bokalanga,T., Logist,A.-S., Bloemen,M. and Maes,P.
EPI_ISL_17793290	Microbiology, Immunology and Transplantation, KU Leuven	Microbiology, Immunology and Transplantation, KU Leuven	Vanmechelen,B., Wawina-Bokalanga,T., Logist,A.-S., Bloemen,M. and Maes,P.
EPI_ISL_17793295, EPI_ISL_17797718, EPI_ISL_17797720, EPI_ISL_17797725, EPI_ISL_17797729, EPI_ISL_17797730, EPI_ISL_17797736	Microbiology, Immunology and Transplantation, KU Leuven	Microbiology, Immunology and Transplantation, KU Leuven	Wawina-Bokalanga,T., Vanmechelen,B., Logist,A.-S., Bloemen,M. and Maes,P.
EPI_ISL_17797740	Microbiology, Immunology and Transplantation, KU Leuven	Microbiology, Immunology and Transplantation, KU Leuven	Vanmechelen,B., Wawina-Bokalanga,T., Logist,A.-S., Bloemen,M. and Maes,P.
EPI_ISL_17797742, EPI_ISL_17797746	Microbiology, Immunology and Transplantation, KU Leuven	Microbiology, Immunology and Transplantation, KU Leuven	Wawina-Bokalanga,T., Vanmechelen,B., Logist,A.-S., Bloemen,M. and Maes,P.
EPI_ISL_17797750	Microbiology, Immunology and Transplantation, KU Leuven	Microbiology, Immunology and Transplantation, KU Leuven	Vanmechelen,B., Wawina-Bokalanga,T., Logist,A.-S., Van Holm,B., Bloemen,M. and Maes,P.
EPI_ISL_17809521	Hangzhou Center for Disease Control and Prevention	Hangzhou Center for Disease Control and Prevention	Lijiao Ao , Jun Li , Yue Yu
EPI_ISL_17817239, EPI_ISL_17817240, EPI_ISL_17817241	Tokyo Metropolitan Institute of Public Health	Tokyo Metropolitan Institute of Public Health	Fumi Kasuya, Wakaba Okada, Ryota Kumagai, Sachiko Harada, Arisa Amano, Michiya Hasegawa, Mami Nagashima, Kenji Sadamasu
EPI_ISL_17821096, EPI_ISL_17821097, EPI_ISL_17821098	ACL Laboratories	RIPHL at Rush University Medical Center	Stefan Green, Kevin Kunstman, Hannah Barbian, Felix Araujo Perez, Edith Perez, Sofiya Bobrovska, Alyse Kittner, Cecilia Chau, Giancarlo Balangue, Lok Yiu Ashley Wu
EPI_ISL_17821099, EPI_ISL_17821100, EPI_ISL_17821101	Quest Diagnostics	RIPHL at Rush University Medical Center	Stefan Green, Kevin Kunstman, Hannah Barbian, Felix Araujo Perez, Edith Perez, Sofiya Bobrovska, Alyse Kittner, Cecilia Chau, Giancarlo Balangue, Lok Yiu Ashley Wu
EPI_ISL_17834476	California Department of Public Health	California Department of Public Health	Kath, C., Haw, M., Espinosa, A., and Hacker, J.
EPI_ISL_17960864, EPI_ISL_17960865, EPI_ISL_17960866	Laboratorio Nacional de Salud Pública Dr. Defilló	Laboratorio Nacional de Salud Pública Dr. Defilló	Isaac Miguel Sánchez, Carlos Vergara Castillo, Edwin Féliz, Anny Peña, Pedro Martinez, Yeny E. Lara Perez, Robinson Agramonte
EPI_ISL_17972012	California Department of Public Health (CDPH)	California Department of Public Health (CDPH)	Kath,C., Haw,M., Espinosa,A. and Hacker,J.
EPI_ISL_17988352, EPI_ISL_17988365, EPI_ISL_17988370, EPI_ISL_17988372	Laboratorio Central de Salud Publica	Laboratorio Central de Salud Publica	Cynthia Vazquez, Vagner Fonseca, Andrea Gomez de la Fuente, Sandra Gonzalez, Fatima Fleitas, Mauricio Lima, Natalia R. Guimaraes, Felipe C. M. Iani, Analia Rojas, Tania Alfonso, Cesar Cantero, Julio Barrios, Shirley Villalba, Maria Jose Ortega, Juan Torales, Maria Liz Gamarra, Carolina Aquino, Jairo Mendez Rico, Luiz Carlos Junior Alcantara, Marta Giovanetti
EPI_ISL_18064642, EPI_ISL_18064643, EPI_ISL_18064644, EPI_ISL_18064645	Laboratory of Microbiology and Virology, Ospedale Amedeo di Savoia, ASL "Città di Torino"	Laboratory of Microbiology and Virology, Ospedale Amedeo di Savoia, ASL "Città di Torino"	Francesco Cerutti, Tiziano Allice, Maria Grazia Milia, Gabriella Gregori, Elisa Burdino, Sara Monteleone, Marisa Cazzadore, Valeria Ghisetti
EPI_ISL_18128768	Bichat-Claude Bernard Hospital, Paris France	Institut Pasteur	Aurelia Kwasiborski, Véronique Hourdel, Charlotte Balière, Damien Hoinard, Quentin Grassin, Maxence Feher, Clémentine De La Porte Des Vaux, Mélanie Cresta, Jessica Vanhomwegen, Jean-Claude Manuguerra, Christophe Batéjat, Valérie Caro
EPI_ISL_18137801, EPI_ISL_18137803, EPI_ISL_18137814, EPI_ISL_18137815, EPI_ISL_18137816, EPI_ISL_18137817	Northwestern Medicine	RIPHL at Rush University Medical Center	Stefan Green, Kevin Kunstman, Hannah Barbian, Sofiya Bobrovska, Felix Araujo Perez, Edith Perez, Cecilia Chau, Giancarlo Balangue, Lok Yiu Ashley Wu, Trisha Jeon, Marisol Dominguez, Latifah Boyd, Lacy Simons
EPI_ISL_18147335	Korea Disease Control and Prevention Agency	Korea Disease Control and Prevention Agency	Chung,Y.-S., Yi,H., Choi,M.-M., Kim,J.-W., Lee,M., Lee,S., Sim,G., Lee,J.H., Shin,H. and Choi,C.
EPI_ISL_18161271, EPI_ISL_18161303, EPI_ISL_18161304	Quest Diagnostics Nichols Institute	Los Angeles County Public Health Laboratories	J. Garrigues et al.
EPI_ISL_18213374, EPI_ISL_18213375	Institute for Hepatology,Shenzhen Third People's Hospital	Institute for Hepatology,Shenzhen Third People's Hospital	Lin Cheng,Zheng Zhang
EPI_ISL_18241788, EPI_ISL_18241789	Unidade de Genômica - UFRJ	Unidade de Genômica - UFRJ	Carolina Moreira Voloch, Filipe Romero Rebello Moreira, Diana Mariani, Rafael Mello Galliez, Debora Souza Faffe, Terezinha Marta Pereira Pinto Castiñeiras, Clarissa Damaso, Amílcar Tanuri.
EPI_ISL_18285962, EPI_ISL_18285963	Quest Diagnostics Nichols Institute	Los Angeles County Public Health Laboratories	J. Garrigues et al.
EPI_ISL_18285971	Cedars-Sinai Medical Center	Los Angeles County Public Health Laboratories	J. Garrigues et al.
EPI_ISL_18308395, EPI_ISL_18308397	National Virus Reference Laboratory	National Virus Reference Laboratory	Gabriel Gonzalez, Michael Carr, Emer O'Byrne, Weronika Banka, Brian Keogan, Jonathan Dean, Daniel Hare, Cillian F De Gascun
EPI_ISL_18323780, EPI_ISL_18323784	California Department of Public Health	California Department of Public Health	Kath, C., Haw, M., Espinosa, A., and Hacker, J.
EPI_ISL_18386999, EPI_ISL_18387001, EPI_ISL_18387005	NC - Los Angeles County Public Health Laboratories	NC - Los Angeles County Public Health Laboratories	Garrigues,J.M. and Green,N.M.
EPI_ISL_18399136, EPI_ISL_18399142, EPI_ISL_18399144, EPI_ISL_18399145	California Department of Public Health	California Department of Public Health	Kath, C., Haw, M., Espinosa, A., and Hacker, J.
EPI_ISL_18436040	PKC Mampang Prapatan	National Institute of Health Research and Development	Fajar Nur Sulistyahadi, Arie Ardiansyah Nugraha, Hana Apsari Pawestri, Kartika Dewi Puspa, Herna, Subangkit, IGM Wirabrata
EPI_ISL_18436041	PKC Jatinegara	National Institute of Health Research and Development	Fajar Nur Sulistyahadi, Arie Ardiansyah Nugraha, Hana Apsari Pawestri, Kartika Dewi Puspa, Herna, Subangkit, IGM Wirabrata
EPI_ISL_18452334, EPI_ISL_18452347	California Department of Public Health	California Department of Public Health	Kath, C., Haw, M., Espinosa, A., and Hacker, J.
EPI_ISL_18463161	RSUD Kembangan	National Institute of Health Research and Development	Hana Apsari Pawestri, Arie Ardiansyah Nugraha, Fajar Nur Sulistyahadi, Hartanti Dian Ikawati, Kartika Dewi Puspa, Markus Evan Anggia, Subangkit, Nelis Imaningsih, IGM Wirabrata
EPI_ISL_18467794	Eka Hospital BSD	National Institute of Health Research and Development	Fajar Nur Sulistyahadi, Hana Apsari Pawestri, Arie Ardiansyah Nugraha, Hartanti Dian Ikawati, Kartika Dewi Puspa, Subangkit, IGM Wirabrata
EPI_ISL_18467795, EPI_ISL_18467796	PKM Kembangan	National Institute of Health Research and Development	Hana Apsari Pawestri, Arie Ardiansyah Nugraha, Fajar Nur Sulistyahadi, Hartanti Dian Ikawati, Kartika Dewi Puspa, Subangkit, IGM Wirabrata
EPI_ISL_18467797	PKC Cengkareng	National Institute of Health Research and Development	Arie Ardiansyah Nugraha, Fajar Nur Sulistyahadi, Hartanti Dian Ikawati, Kartika Dewi Puspa, Hana Apsari Pawestri, Subangkit, IGM Wirabrata
EPI_ISL_18467798	PKC Grogol Petamburan	National Institute of Health Research and Development	Fajar Nur Sulistyahadi, Hana Apsari Pawestri, Arie Ardiansyah Nugraha, Hartanti Dian Ikawati, Kartika Dewi Puspa, Subangkit, IGM Wirabrata
EPI_ISL_18467799	PKC Setiabudi	National Institute of Health Research and Development	Hana Apsari Pawestri, Arie Ardiansyah Nugraha, Fajar Nur Sulistyahadi, Hartanti Dian Ikawati, Kartika Dewi Puspa, Subangkit, IGM Wirabrata
EPI_ISL_18567806, EPI_ISL_18567807	Southern Nevada Public Health Laboratory	Southern Nevada Public Health Laboratory	Horng-Yuan Kan
EPI_ISL_18702209	Center for Vectors and Infectious Diseases Research (CEVDI), National Health Institute Doutor Ricardo Jorge, IP (INSA)	Center for Vectors and Infectious Diseases Research (CEVDI), National Health Institute Doutor Ricardo Jorge, IP (INSA)	Isidro,J., Borges,V., Pinto,M., Sobral,D., Santos,J., Nunes,A., Mixao,V., Ferreira,R., Santos,D., Duarte,S., Vieira,L., Borrego,M., Nuncio,S., Lopes de Carvalho,I., Pelerito,A., Cordeiro,R. and Gomes,J.P.
EPI_ISL_18723947	DCHHS Sexual Health Clinic	Dallas County Health & Human Services Public Health Laboratory	Kabir, Farruk; Plaisance, Erin; Stringer, Joey; Short, Luke.
EPI_ISL_18737443, EPI_ISL_18737446, EPI_ISL_18737448, EPI_ISL_18737524	Parkland Health and Hospital System	Dallas County Health & Human Services Public Health Laboratory	Kabir, Farruk; Plaisance, Erin; Stringer, Joey; Short, Luke.
EPI_ISL_18744048	National Medical Center	National Medical Center	Jun-sun Park, Hongsoon Yim, Jihye Um, Hyang Su Kim, BumSik Chin, Jaehyun Jeon, Yeonjae Kim, Min-Kyung Kim
EPI_ISL_18747002, EPI_ISL_18747025	Parkland Health and Hospital System	Dallas County Health & Human Services Public Health Laboratory	Kabir, Farruk; Plaisance, Erin; Stringer, Joey; Short, Luke.
EPI_ISL_18755970, EPI_ISL_18755972, EPI_ISL_18755977, EPI_ISL_18755979	Erasmus Medical Center Department of Virology	Erasmus Medical Center Department of Virology	Leonard Schuele, Marjan Boter, Babs Verstrepen, Richard Molenkamp, Marion Koopmans, Bas Oude Munnink
EPI_ISL_18781788, EPI_ISL_18781791, EPI_ISL_18781792, EPI_ISL_18781793, EPI_ISL_18781811, EPI_ISL_18781833, EPI_ISL_18781836, EPI_ISL_18781849, EPI_ISL_18781923, EPI_ISL_18781924, EPI_ISL_18781925, EPI_ISL_18781928, EPI_ISL_18781929, EPI_ISL_18786340, EPI_ISL_18786342, EPI_ISL_18786347, EPI_ISL_18786349, EPI_ISL_18786350, EPI_ISL_18786351, EPI_ISL_18786357, EPI_ISL_18786358, EPI_ISL_18959312, EPI_ISL_18959314, EPI_ISL_18959318, EPI_ISL_18959321, EPI_ISL_18959324			
see above	California Department of Public Health	California Department of Public Health	Kath, C., Haw, M., Espinosa, A., and Hacker, J.
EPI_ISL_18993197	Los Angeles County Public Health Laboratory	Los Angeles County Public Health Laboratories	J. Garrigues et. al.
EPI_ISL_18993200, EPI_ISL_18993203	Quest Diagnostics Nichols Institute	Los Angeles County Public Health Laboratories	J. Garrigues et. al.
EPI_ISL_18993206	Quest Diagnostics West Hills	Los Angeles County Public Health Laboratories	J. Garrigues et. al.
EPI_ISL_19012435	Laboratorio de Enterovirus, Instituto Oswaldo Cruz, Fiocruz	Oswaldo Cruz Foundation Laboratory of Respiratory Virus and Measles	Paola Resende, Elisa Cavalcante Pereira, Bruna Mendonça da Silva, Jéssica Graça Macedo de Carvalho, Larissa Macedo Pinto, Victor Guimaraes, Marilda Siqueira, Renan da Silva Faustino, Marilia Santini, Edson Elias da Silva on behalf of the Fiocruz Genomic Surveillance Network

EPI_ISL_19016746	Quest Diagnostics	RIPHL at Rush University Medical Center	Stefan Green, Kevin Kunstman, Hannah Barbian, Sofiya Bobrovska, Felix Araujo Perez, Erin Newcomer
EPI_ISL_19016819	Quest Diagnostics	Regional Innovative Public Health Laboratory (RIPHL) at Rush University Medical Center	Stefan Green, Kevin Kunstman, Hannah Barbian, Sofiya Bobrovska, Felix Araujo Perez, Erin Newcomer
EPI_ISL_19031631, EPI_ISL_19031636, EPI_ISL_19031637	Quest Diagnostics	RIPHL at Rush University Medical Center	Stefan Green, Kevin Kunstman, Hannah Barbian, Sofiya Bobrovska, Felix Araujo Perez, Erin Newcomer, Alyse Kitttner
EPI_ISL_19058871	Guangdong Provincial Center for Disease Control and Prevention, Institute of Pathogenic Microbiology	Guangdong Provincial Center for Disease Control and Prevention, Institute of Pathogenic Microbiology	Li,B., Zhao,W. and Shen,C.
EPI_ISL_19131278, EPI_ISL_19131279	Laboratory Medicine and Pathology, University of Washington	Laboratory Medicine and Pathology, University of Washington	Roychoudhury,P., Xie,X., Sereewit,J., Ellis,S. and Greninger,A.
EPI_ISL_19139151	QUEST DIAGNOSTICS WEST HILLS	Los Angeles County Public Health Laboratories	N. Heibeck et al.
EPI_ISL_19143450, EPI_ISL_19143462, EPI_ISL_19143467	California Department of Public Health	California Department of Public Health	Kath, C., Haw, M., Espinosa, A., and Hacker, J.
EPI_ISL_19151801	Tokyo Metropolitan Institute of Public Health	Tokyo Metropolitan Institute of Public Health	Wakaba Okada, Ryota Kumagai, Sachiko Harada, Yu Yaoita, Arisa Amano, Kumiko Takahashi, Mami Nagashima, Kenji Sadamasu
EPI_ISL_19158945, EPI_ISL_19158948, EPI_ISL_19158949	Northwestern Medicine	RIPHL at Rush University Medical Center	Stefan Green, Kevin Kunstman, Hannah Barbian, Sofiya Bobrovska, Felix Araujo Perez, Erin Newcomer, Alyse Kitttner
EPI_ISL_19170460, EPI_ISL_19170468, EPI_ISL_19170472, EPI_ISL_19170475, EPI_ISL_19170478, EPI_ISL_19170479	California Department of Public Health	California Department of Public Health	Kath, C., Haw, M., Espinosa, A., and Hacker, J.
EPI_ISL_19196359	Quest Diagnostic Nichols Institute	Los Angeles County Public Health Laboratories	S. McCann et. al.
EPI_ISL_19205402, EPI_ISL_19205405	Laboratorio de Enterovirus, Instituto Oswaldo Cruz, Fiocruz	Instituto Oswaldo Cruz FIOCRUZ - Laboratory of Respiratory Viruses and Measles (LVRS)	Paola Resende, Elisa Cavalcante Pereira, Bruna Mendonça da Silva, Jéssica Graça Macedo de Carvalho, Larissa Macedo Pinto, Victor Guimaraes, Luciana Appolinario, Alice Sampaio, Marilda Siqueira, Renan da Silva Faustino, Marilia Santini, Edson Elias da Silva on behalf of the Fiocruz Genomic Surveillance Network
EPI_ISL_19230668	Virologia, INEI- ANLIS Dr. Carlos G. Malbran	Virologia, INEI- ANLIS Dr. Carlos G. Malbran	Lewis,A., Josiowicz,A., Poklepovich,T., Mallou,F., Cuba,F., Haim,M. and Cisterna,D.
EPI_ISL_19243972, EPI_ISL_19243973	Laboratory Medicine and Pathology, University of Washington	Laboratory Medicine and Pathology, University of Washington	Roychoudhury,P., Xie,X., Sereewit,J., Ellis,S. and Greninger,A.
EPI_ISL_19256209	Nigeria Centre for Disease Control and Prevention	Institute of Ecology and Evolution, University of Edinburgh	Parker,E., Omah,I.F., Varilly,P., Magee,A., Ayinla,A.O., Sijuwola,A.E., Ahmed,M.I., Ope-ewe,O.O., Ogunsanya,O.A., Olono,A., Eromon,P., Tomkins-Tinch,C.H., Otieno,J.R., Akanbi,O., Egwuenu,A., Ehiakhamen,O., Chukwu,C., Suleiman,K., Akinpelu,A., Ahmad,A., Imam,K.I., Ojedele,R., Oripenaye,V., Ikeata,K., Adelakun,S., Olajumoke,B., Djuicy,D.D., Messanga Essengue,L.L., Moumbeket Yifomnjou,M.H., Zeller,M., Gangavarapu,K., O'Toole,A., Park,D.J., Mboowa,G., Tessema,S.K., Tebeje,Y.K., Folarin,O., Happi,A., Lemey,P., Suchard,M.A., Andersen,K.G., Sabeti,P., Rambaut,A., Njoum,R., Ihekweazu,C., Jide,I., Adetifa,I. and Happi,C.T.
EPI_ISL_19295761, EPI_ISL_19295762, EPI_ISL_19295763, EPI_ISL_19295765, EPI_ISL_19295767, EPI_ISL_19295768, EPI_ISL_19295770	California Department of Public Health	California Department of Public Health	Kath, C., Haw, M., Espinosa, A., and Hacker, J.
